# Transcriptome analysis identifies key gene *LiMYB305* involved in monoterpene biosynthesis in *Lilium* ‘Siberia’

**DOI:** 10.3389/fpls.2022.1021576

**Published:** 2022-11-07

**Authors:** Yun-Yao Yang, Bo Ma, Ying-Ying Li, Ming-Zheng Han, Jing Wu, Xiao-Feng Zhou, Ji Tian, Wen-He Wang, Ping-Sheng Leng, Zeng-Hui Hu

**Affiliations:** ^1^ Beijing Advanced Innovation Center for Tree Breeding by Molecular Design, College of Landscape Architecture, Beijing University of Agriculture, Beijing, China; ^2^ Beijing Key Laboratory of Development and Quality Control of Ornamental Crops, Department of Ornamental Horticulture, China Agricultural University, Beijing, China

**Keywords:** *Lilium* ‘siberia’, flower fragrance, monoterpenes, RNA-Seq, LiMYB305

## Abstract

*Lilium* is a popular cut flower that is highly favored by consumers due to its snowy white color and strong fragrance, which originates from the release of monoterpenes. However, the underlying molecular mechanism of monoterpene synthesis remains poorly understood. In this study, the content of three main monoterpenes (linalool, ocimene, and myrcene) was examined in *Lilium* ‘Siberia’, and RNA sequencing of the 11 stages of flower development was conducted. The biosynthesis of the three monoterpenes increased with flower development, reaching their peak levels at the full flowering stage. Transcriptome data revealed 257,140 unigenes, with an average size of 794 bp, from which 43,934 differentially expressed genes were identified and enriched in the KEGG pathways partly involved in plant hormone signal transduction and monoterpenoid biosynthesis. Furthermore, the essential factor *LiMYB305* was identified by WGCNA after the release of the flower fragrance. The transient silencing of *LiMYB305* in petals using VIGS technology showed that the mRNA expression levels of *LiLiS*, *LiOcS*, and *LiMyS* were significantly downregulated and that the release of linalool, ocimene, and myrcene had decreased significantly. Y1H, LUC, and EMSA experiments revealed that LiMYB305 directly bound and activated the *LiOcS* promoter to increase the synthesis of monoterpenes. Taken together, these results provide insight into the molecular mechanism of monoterpene synthesis and provide valuable information to investigate the formation of the flower fragrance in *Lilium*.

## Introduction

Plants emit a series of volatile organic compounds (VOCs) to protect against pathogens, parasites, and herbivores, as well as to attract pollinators to facilitate successful pollination (Kessler and Baldwin, 2001; Dudareva et al., 2013). The floral fragrance is typically composed of a series of VOCs, including terpenoids, phenylpropanoids/benzenoids, and fatty acids and their derivatives, which are involved in plant-plant or plant-other interactions. To date, 1,700 floral VOCs have been identified in 1,000 seed plants (Knudsen et al., 2006). Among them, terpenes occupy a pivotal position.

*Lilium* is a well-known bulb flower with very high ornamental and commercial value worldwide. As an important horticultural crop, *Lilium* is not only widely used in gardens, but also comprises an indispensable proportion of the world’s fresh-cut flower market, due to its elegant color, beautiful shape, and unique fragrance. The fragrance of *Lilium* is an essential factor affecting its ornamental and commercial value. Although *Lilium* has a long history of breeding and many varieties have been cultivated (Abbas et al., 2019), its fragrance was ignored for a long time. However, the fragrance and its biosynthesis have attracted significant attention in *Lilium* more recently. Large differences in flower fragrance have been reported among *Lilium* species and cultivars (Zhang et al., 2013; Du et al., 2019), and both unscented and scented types exist, which serve as an extremely important genetic resource (Hu et al., 2017 **)**. In this study, we revealed that monoterpenes are the key aromatic compounds in *Lilium*. The release of monoterpenes accounts for more than 80% of the total fragrance released by scented *Lilium*, which is almost undetectable in unscented *Lilium* (Zhang et al., 2013). Moreover, among the monoterpenes, linalool, ocimene, and myrcene are the predominant components in scented *Lilium* (Hu et al., 2017; Du et al., 2019). Thus, understanding the regulatory mechanisms of monoterpene biosynthesis is crucial for breeding flower fragrance to improve the ornamental and commercial value of *Lilium*.

Terpenes are synthesized through the 2-C-methyl-D-erythritol-4-phosphate (MEP) pathway, which contributes to the production of monoterpenes in the plastid, and by the mevalonate (MVA) pathway, which intervenes in the synthesis of sesquiterpenes or triterpenes in the cytoplasm (Tholl et al., 2005). As genes encoding upstream enzymes in the MEP pathway, *LiDXS* and *LiDXR* have been cloned from *Lilium*, and their expression levels are consistent with the amounts of monoterpenes released (Hu et al., 2017; Zhang et al., 2018). Flowers of *LiDXR* transgenic tobacco lines have high monoterpene content (Zhang et al., 2018). In addition, TPSs, as the end-stage enzymes in the MEP and MVA pathways, directly participate in the synthesis of terpenes. We have reported that TPS mRNA levels change to coincide with monoterpene release, and their mRNA levels are higher in scented *Lilium* than in unscented *Lilium* (Hu et al., 2017). More than that, LoTPS1 and LoTPS3 promote the production of monoterpenes (Abbas et al., 2019) and are regulated by hexokinase (*LoHXK*) and fructokinase (*LoFRK*) (Abbas et al., 2021a). Although some genes in the monoterpene biosynthesis pathway in *Lilium* have been identified, the regulatory mechanisms are largely unknown.

Transcriptional regulation plays an important role in terpene synthesis. In the model plant *Arabidopsis thaliana*, the release of sesquiterpene is inhibited by the *myb21* mutation (Reeves et al., 2012), and AtMYC2 regulates the expression of *AtTPS11* and *AtTPS21* (Hong et al., 2012). A recent study talked about that MYB21 interacted with MYC2 to regulate the expression of *TPS*s, further affecting the production and the release of terpenes in *F. hybrida* and *A*. *thaliana* (Yang et al., 2020). Ding et al. (2020) reported the potential roles of OfWRKYs, which contain plant zinc cluster domains, in regulating the synthesis of aromatic compounds in sweet *Osmanthus fragrans*. Abbas et al. (2021b) determined that the HcMYB affected the regulatory mechanism of terpenoid biosynthesis in *Hedychium coronarium*. Thus, transcription factors (TFs) may be involved in terpene synthesis in *Lilium* (Hu et al., 2017; Shi et al., 2018). Nevertheless, the transcriptional regulation of genes involved in monoterpene synthesis in *Lilium* is largely unknown.

A lack of *Lilium* genomic information has limited research on the molecular mechanism of flower fragrance production. RNA-seq has allowed for investigations of the mechanism of flower fragrance production in non-model plants without a genome. *Lilium* ‘Siberia’, a hybrid of the oriental lily with a strong fragrance, was used in this study. To obtain insight into the transcriptional regulation of the monoterpene biosynthetic pathway, 11 flowering stages were studied to identify the three main monoterpenes, and RNA-seq was performed. Based on this information, candidate genes were identified by weighted correlation network analysis (WGCNA), and their functions were verified by virus-induced gene silencing (VIGS). Our results offer a valuable resource for understanding the regulatory mechanism of *Lilium* monoterpene synthesis, thereby providing the basis for fragrance genetic engineering to improve the ornamental and commercial value of *Lilium*.

## Results

### Transcriptome analysis of petals during development

**Figure 1:**
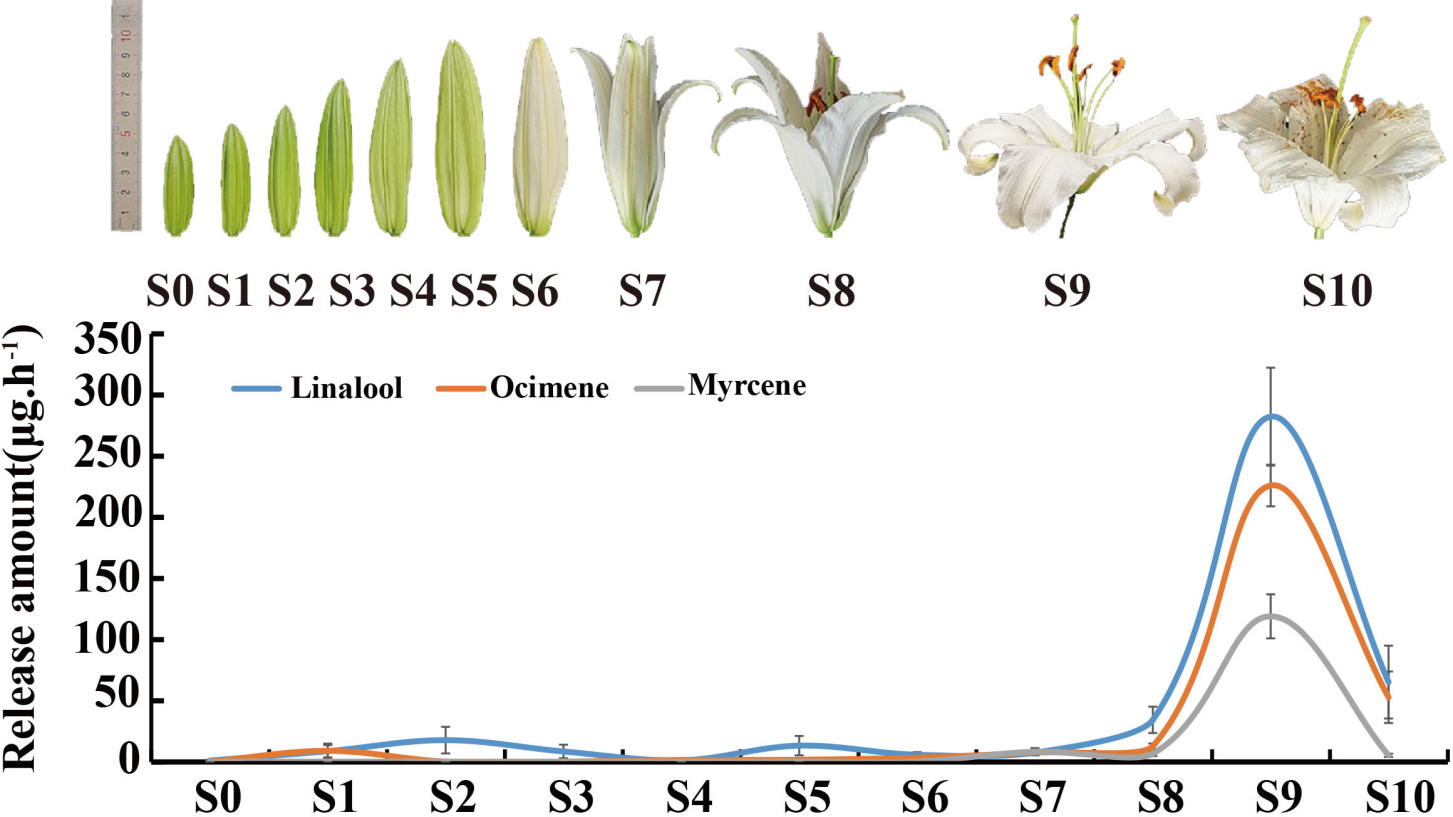
The relative release amounts of volatile components from *Lilium* ‘Siberia’. S0-S5: 5–10 cm; S6: the bud color changing to white; S7: tepals splitting slightly; S8: tepals semi-opening; S9: tepals fully opened; S10: petals decaying.

To analyze the dynamic changes of ‘Siberia’ fragrance synthesis, blooming ‘Siberia’ was divided into 11 periods (**Figure 1**), and three main monoterpenes were identified. The trend in the release of the three main monoterpenes was similar to that reported by Hu et al. (2017), reaching its peak during the blooming period and then declining. The highest monoterpene release rate was 280 μg·h^-1^ of linalool, which was 1.25- and 2.37-fold higher than that of ocimene and myrcene, respectively. Linalool was detected when the flower buds were young (S0), and the amount released increased gradually during flower development. However, a trace of myrcene was detected when development reached S7 (**Figure 1**).

To further explore the molecular mechanisms of fragrance production in *Lilium* ‘Siberia’, cDNA libraries of the 11 developmental periods were prepared for transcriptomic analysis (SRA: SRP352614). After removing the low-quality reads, 480.82 Gb of clean data were obtained (**Supplementary Table 1**). Based on the high-quality reads, the Q30 values of the sequences were > 91.67%, and GC content was 42.88% (**Supplementary Table 2** **)**, indicating that the reads could be used for further analysis. Additional information on the transcriptome data is shown in **Supplementary Tables 1**, **2**. After trimming and assembly, 257,140 unigenes were generated, with an average length of 794 bp (**Supplementary Table 2**). Of these, 147,039 (57%) unigenes were shorter than 500 bp, 103,999 (41%) were 500-3,000 bp, and 6,102 were longer than 3,500 bp (**Supplementary Figure 1A**).

As shown in **Supplementary Figure 1B**, 72,897 unigenes were annotated in six protein databases, including the NCBI Non-Redundant (NR, 67,899 unigenes, 26.41%), Swiss-Prot (41,700, 16.22%), Pfam (32,107, 12.49%), Cluster of Orthologous Groups (COG, 8,937, 3.48%), Gene Ontology (GO, 47,469, 18.46%), and Kyoto Encyclopedia of Genes and Genomes (KEGG, 26,412, 10.27%) databases (**Supplementary Figure 1B**). Approximately 71.65% of the unigenes were not annotated, indicating that a large number of new genes remain to be mined. A large number of unigenes in *Lilium* ‘Siberia’ had a high ratio of matched sequences in other plant species. The highest ratio of unigenes was homologous to those of *Asparagus officinalis* (7,267, 10.70%), followed by *Elaeis guineensis* (6,454, 9.51%), and *Phoenix dactylifera* (5,033, 7.41%) **(** **Supplementary Figure 1C** **)**. The COG classifications of the annotated unigenes were determined to assess the effectiveness of the annotation process, and the cluster for “translation, ribosomal structure, and biogenesis” comprised the largest group, followed by “general functional prediction only” and “post-translational modifications, protein turnover, and chaperones” (**Supplementary Figure 2A**). GO classifications were determined to categorize the predicted unigenes in the three main categories of biological processes, cellular components, and molecular functions (**Supplementary Figure 2B**).

### Comparison of the differentially expressed genes (DEGs) from different developmental stages

We compared adjacent stages to identify DEGs during flower development. A total of 43,934 DEGs were identified with FDR < 0.05 and |log2(fold-change)| ≥ 1 (**Supplementary Figure 3**). Of these genes, the S9/S10 DEGs were the most abundant (11,033 upregulated and 15,989 downregulated), followed by S7/S8 (7,160 upregulated and 3,989 downregulated) and S8/S9 (5,797 upregulated and 2,135 downregulated) (**Supplementary Figure 3**).

**Figure 2:**
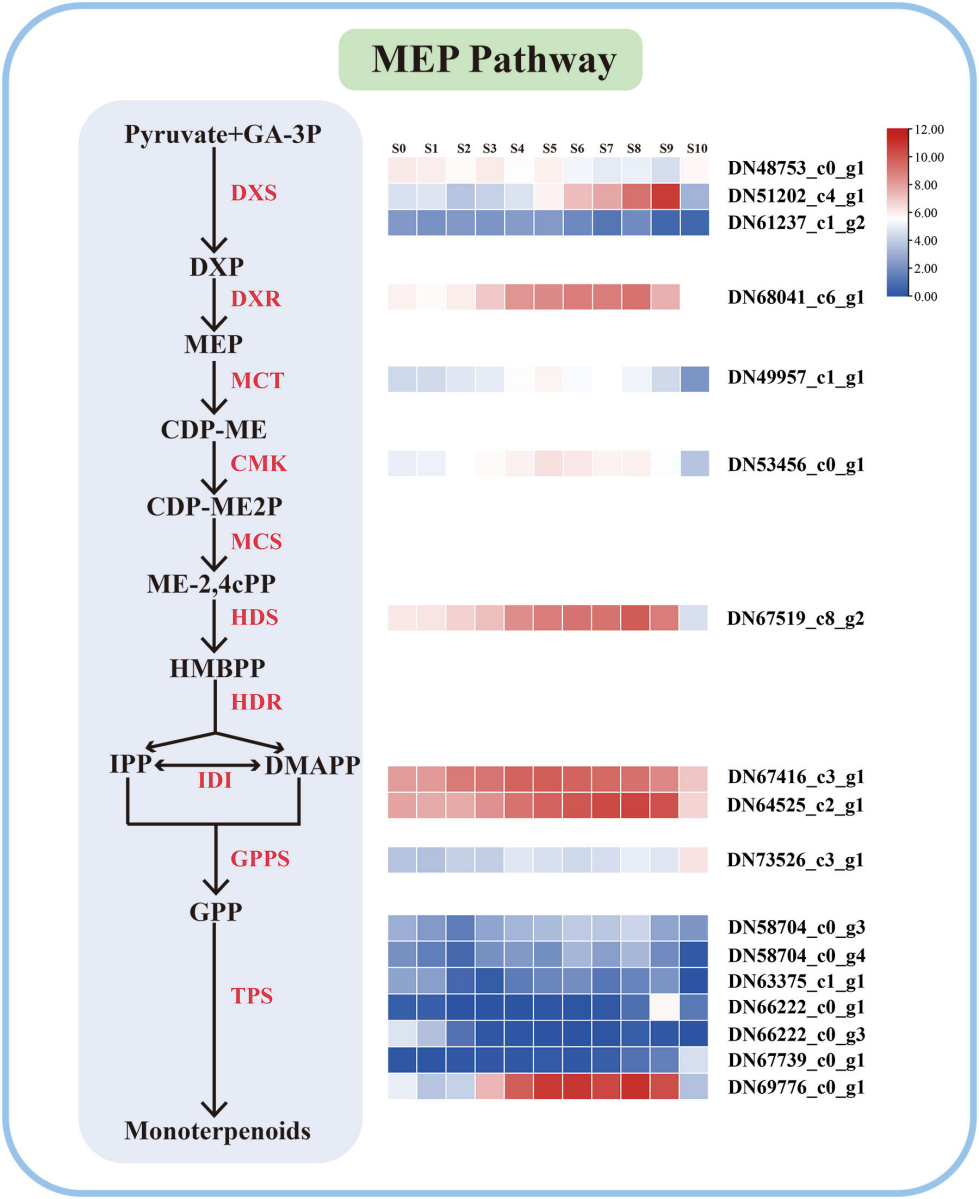
Expression profiles of the genes encoding enzymes involved in monoterpene biosynthesis. The change in bar color from blue to red indicates an expression change from low to high.

Further analysis of these DEGs using the KEGG annotations provided extra information on the enriched biological pathways, including “plant hormone signal transduction”, “phenylpropanoid biosynthesis”, “diterpenoid biosynthesis”, “carotenoid biosynthesis”, “flavonoid biosynthesis”, “fatty acid elongation”, “monoterpenoid biosynthesis”, and “terpenoid backbone biosynthesis” (**Supplementary Table 2**; **Supplementary Figure 4** **)**. These biological processes are all closely related to the biosynthesis of VOCs. A total of 17 unigenes in the ‘Siberia’ transcriptome were identified in the MEP pathway based on functional annotations, most of which had similar expression patterns to the release of monoterpenoids (**Figure 2**). For example, the expression pattern of the unigenes TRINITY_66222_c0_g1 and TRINITY_69776_co_g1, predicted linalool synthase (LiLiS) and ocimene synthase (LiOcS), respectively, and was consistent with the monoterpenoid components (**Figure 2**).

### Identification of WGCNA modules related to the production of flower fragrance

A total of 1,224 TFs were recognized in 34 families, among which the MYB (182), AP2/ERF (115), bHLH (81), NAC (75), and C3H (72) families were ranked in the top five (**Supplementary Figure 5**; **Supplementary Table 3** **)**.

**Figure 3:**
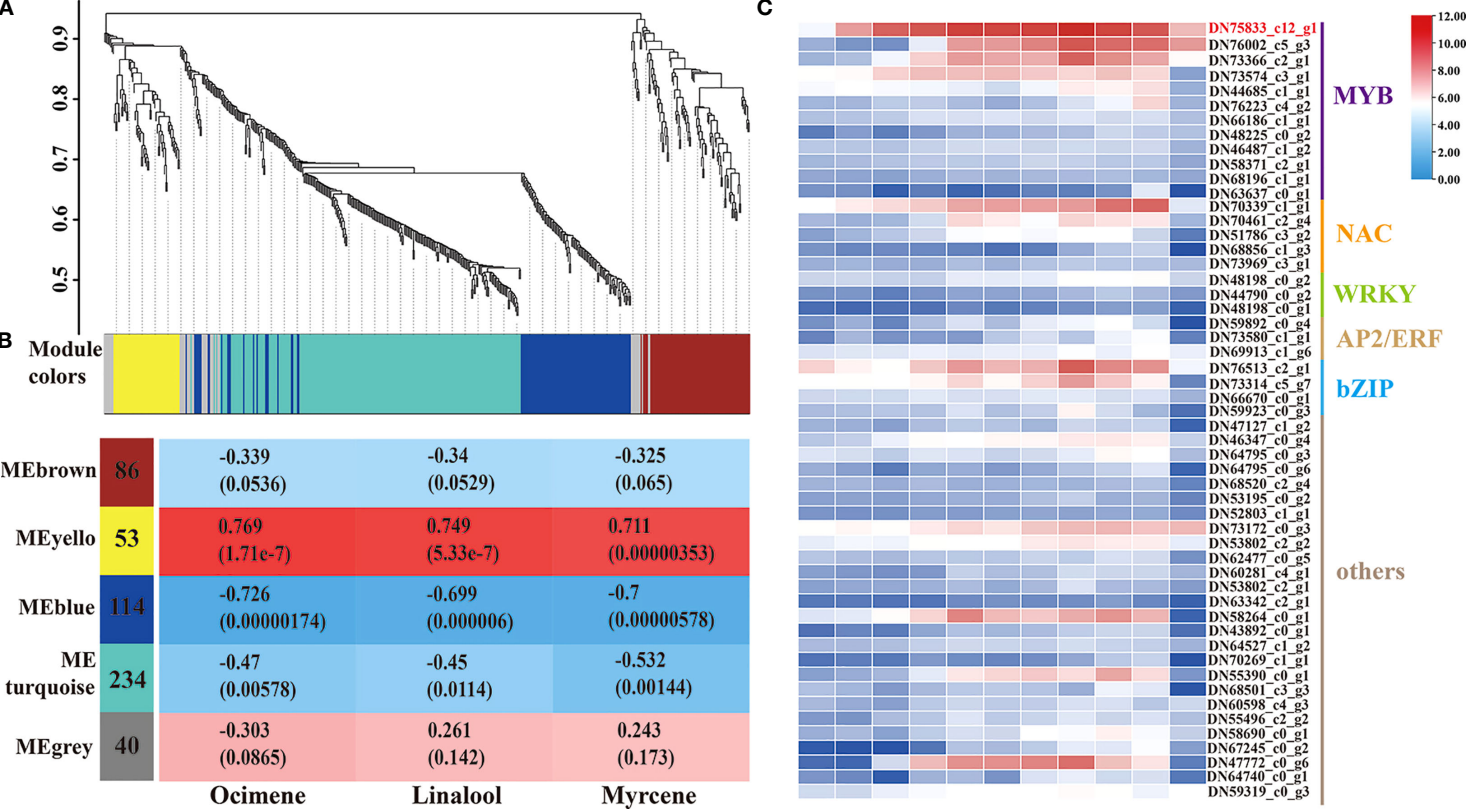
All TFs related to the three main fragrance compounds were identified by WGCNA. **(A)** Dendrogram plot with color annotation. **(B)** Nodule-monoterpenes weight correlations and corresponding *P*-values. **(C)** Heat-map comparison and TF categories in the MEyellow module. The change in the bar color from blue to red indicates a change in expression from low to high.

WGCNA was used to construct a potential regulatory network of TFs controlling the release of the three monoterpenes during flower development. All TFs were included in this analysis, and five distinct modules were established, which were labeled blue, brown, grey, turquoise, and yellow, respectively (**Figure 3A**; **Supplementary Table 4**). The MEyellow module (53 genes) had the highest correlations with ocimene, linalool, and myrcene, with correlation coefficients of 0.769, 0749, and 0.711, respectively (**Figure 3B**). As shown in **Figure 3C**, 53 TFs were identified, including members of the MYB, NAC, WRKY, AP2/ERF, bZIP, and LOB families, among which the MYB family received the most attention. Surprisingly, the unigene sequence (TRINITY_DN75833_c12_g1) belonging to the MYB family with the highest expression and the significant differences between developmental stages attracted our attention (**Supplementary Table 5**). After blasting the sequence against the NCBI database, LiMYB305 was assigned to this sequence, which prompted us to investigate whether it affected monoterpene production.

### *LiMYB305* cloning, expression, and subcellular localization

**Figure 4:**
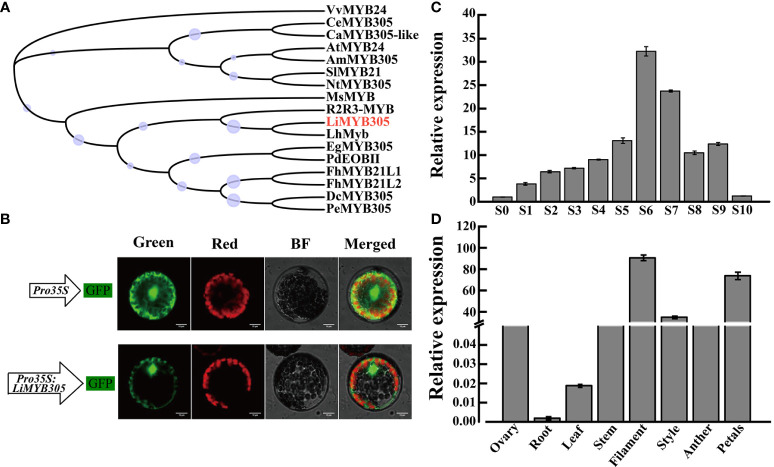
Characterization of *Lilium* ‘Siberia’ LiMYB305. **(A)** Phylogenetic tree of the LiMYB305 protein. LiMYB305 is marked in red. Bootstrap values are shown as a percentage of 1,000 replicates. The purple circle pattern represents the bootstrap, and the larger the circle, the larger the bootstrap. DcMYB305 (XP_020690104.1), PeMYB305 (XP_020572046.1), EgMYB305 (XP_010924268.1), PdEOBII; (XP_008800726.2), R2R3-MYB (AMO43680.1), LhMyb (BAB40790.1), AtMYB24 (AAM63674.1), VvMYB24 (NP_001268062.1), AmMYB305 (P81391.1), NtMYB305 (XP_016506729.1), SIMYB21 (NP_001351943.1), CeMYB305 (XP_027154721.1), CaMYB305-Like (XP_027073189.1), MsMYB (AQR58379.1) FhMYBL1 (QNC43969.1), and FhMYBL2 (QNC43970.1). **(B)** Subcellular localization of LiMYB305. Green, GFP fluorescence detected in the green channel; Red, chlorophyll fluorescence detected in the red channel; Merged, merged green and red channel images; BF, bright-field image. Bars = 10 μm. **(C)** Expression profiles of *LiMYB305* during the 11 developmental stages. **(D)** Expression profiles of *LiMYB305* in eight tissues.

The full-length *LiMYB305* cDNA sequences were cloned from full blooming petals of ‘Siberia’. The open reading frame (ORF) of *LiMYB305* was 555 bp, which encoded a protein of 184 amino acids with a molecular weight of 21.28 kD and a theoretical pI of 5.0. The amino acid analysis revealed that LiMYB305 was an R2R3-MYB type protein comprised of two conserved SANT domains of 15-62 and 68-111 amino acids, respectively (**Supplementary Figure 6**), and the 3D structure simulation is shown in **Supplementary Figure 7**. To further confirm that LiMYB305 was in the R2R3-MYB family, a more detailed phylogenetic tree based on multiple protein sequences was generated with LiMYB305 and some well-studied MYB proteins in lilies and other species. This phylogenetic tree demonstrated that LiMYB305 was closely clustered with LhMyb (**Figure 4A**).

The pBWA(V)HS-LiMYB305-GLosgfp construct and the empty plasmid, which served as the control, were transiently expressed in protoplasts isolated from *A. thaliana* and observed under a laser scanning confocal microscope to determine the subcellular localization of LiMYB305 protein. As shown in **Figure 4B**, the green fluorescence signal of the construct was observed in the nucleus and cytoplasm, while the green fluorescence signal of the empty plasmid was detected throughout the cell. To determine whether *LiMYB305* was involved in fragrance production, its expression patterns were evaluated in *Lilium* ‘Siberia’ at different flowering stages **(** **Figures 4C** **, D**). The quantitative real-time-polymerase chain reaction (qRT-PCR) results indicated an initial increase during S6, a decrease during flower opening, and a slight increase during full flowering (**Figure 4C**). Moreover, the *LiMYB305* expression level in floral tissues was much higher than that in roots, stems, or leaves where there was negligible expression. In addition, the expression level in filaments and petals was significantly higher than that in the ovary, followed by the styles, which were 90, 74, and 35-fold higher than that in the ovary, respectively (**Figure 4D**).

### Functional analysis of LiMYB305 during monoterpene production

**Figure 5:**
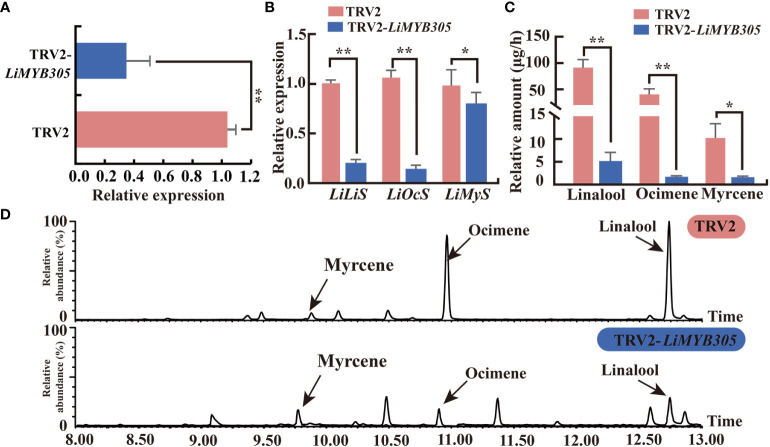
Effect of silencing *LiMYB305* on three main monoterpenoids and gene expression levels. **(A)** qRT-PCR results of the *LiMYB305* mRNA levels in TRV2-*LiMYB305* petals compared with control pTRV2 petals. **(B)** qRT-PCR results of *LiLiS*, *LiOcS*, and *LiMyS* mRNA levels in petals of *LiMYB305*-silenced plants compared with control plants. **(C)** Release of the three main monoterpenoids in petals of *LiMYB305* silenced plants compared with control plants. **(D)** Total ion current (TIC) chromatograms of the flower fragrance compounds emitted from *Lilium* ‘Siberia’. The peaks were myrcene, ocimene, and linalool, respectively. The first column is the TIC of ‘Siberia’ infected with pNC-TRV2 (control), and the second column is the TIC of ‘Siberia’ infected with pNC-TRV2-*LiMYB305.* *P < 0.05, **P < 0.01. Data are presented as mean ± SD, n = 3.

To investigate whether *LiMYB305* participates in the production of monoterpenes in *Lilium* ‘Siberia’, the VIGS method was used to reduce its expression in fully opened *Lilium* flowers. The recombinant vector TRV2-*LiMYB305*, which contained 200 bp from *LiMYB305*, was generated. *LiMYB305* expression was significantly lower in the *LiMYB305*-silenced plants than in the TRV control plants (79% decrease in the *LiMYB305* transcript, **Figure 5A**). Consistent with this pattern, the silenced plants also presented significantly lower expression of *LiLiS* and *LiOcS* than the TRV control discs, which decreased by 79.5% and 84.5%, respectively (**Figure 5B**). The gas chromatography-mass spectrometry analysis revealed that silencing *LiMYB305* led to significant decreases (94.45%, and 95.97%) in the amounts of linalool and ocimene released, respectively (**Figure 5C**). Likewise, the decreased expression of *LiMYB305* affected the expression of *LiMyS* and the synthesis of myrcene (**Figures 5B** **, C**). As expected, the abundance of the total ion current (TIC) of TRV2-*LiMYB305*-silenced plants was characteristically sparse, as opposed to control TRV2-infected petals (**Figure 5D**).

### Regulatory role of LiMYB305 on *LiOcS*

**Figure 6:**
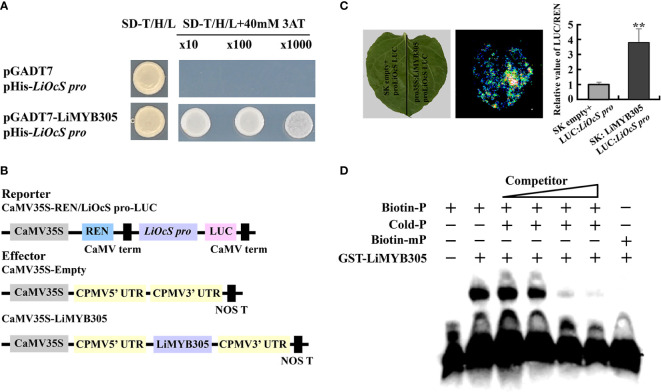
Interaction between LiMYB305 and the *LiOcS* promoter. **(A)** Analysis of LiMYB305 binding to the *LiOcS* promoter in the yeast one-hybrid system. The empty prey vector (AD) was used as the negative control. **(B)** Schematic representation of the constructed dual LUC reporter system. **(C)** Live imaging (left) and quantitative analysis (right) of transcriptional repression of the *LiOcS* promoter by LiMYB305. The LUC : LiOcS pro construct was co-infiltrated with SK : LiMYB305 or the SK empty vector in *N. benthamiana* leaves. Experiments were independently repeated three times. A representative image of an *N. benthamiana* leaf 3 days after infiltration is shown. Mean ± SD are shown from three replicates (n = 3). **(D)** EMSA of LiMYB305 binding to the *LiOcS* promoter. **P < 0.01.

A *cis*-element (CAGTTA), which has been confirmed to bind to AtMYB21 in *Arabidopsis*, was discovered on the *LiOcS* promoter, between −72 and −78 bp upstream of “ATG” (**Supplementary Figure 8**). Therefore, yeast-one-hybrid (Y1H) assays were carried out to determine whether LiMYB305 is bound to the *LiOcS* promoter. As shown in **Figure 6A**, LiMYB305 directly bound the *LiOcS* promoter (**Figure 6A**). To verify the Y1H assays, the *LiOcS* promoter was isolated and constructed with the firefly LUC reporter (*ProLiOcS :* LUC) to confirm whether LiMYB305 affected transactivation (**Figure 6B**). The reporter constructs, along with an effector construct consisting of LiMYB305 driven by the *35S* promoter (*Pro35S*:*LiMYB305*) and *Pro35S*:*REN* as an internal control, were co-infiltrated into *N. benthamiana* leaves. Live imaging and quantitative analysis demonstrated that co-infiltration of LiMYB305 with ProLiOcS : LUC significantly increased the expression of the LUC reporter, indicating that LiMYB305 may activate the expression of *LiOcS* (**Figure 6C**). To confirm these results, the direct binding of LiMYB305 to the cis-element (CAGTTA) was reconstructed by electrophoretic mobility shift assay (EMSA) *in vitro*. The results show that GST-LiMYB305 specifically recognized the biotin -labeled probe, but the binding activity disappeared when the cis-element was mutated (**Figure 6D**). Taken together, these results indicate the specific binding of LiMYB305 to the *LiOcS* promoter.

## Discussion

Flower fragrance is an important characteristic of *Lilium* that affects its ornamental and commercial value. Monoterpenes are the most prominent compounds in the floral fragrance of *Lilium* (Hu et al., 2017). A better understanding of monoterpene biosynthesis and its regulatory mechanism has provided new insight into the genetic engineering of fragrance and monoterpene production while considering the commercial significance of *Lilium*. In this study, the development of lily petals was divided into 11 periods, and changes in the main monoterpenoids were observed, which enriched the four periods examined by Hu et al. (2017). The levels of monoterpenoids increased to their highest levels at the full flower blooming stage and then decreased during flower decay, consistent with the results of Hu et al. (2017). In addition, the weak release of linalool and ocimene was captured at the young flower bud stage (**Figure 1**). Emissions of linalool and the production of linalool oxides help *Arabidopsis* reduce the attractiveness of its flowers and protect against pests (Boachon et al., 2015). A similar hypothesis proposes that monoterpenes have an important defensive role against pests in *Pinus armandi* (Pham et al., 2014). Therefore, we speculate that the weak release of monoterpenes at the young flower bud stage may be related to protection from insect pests.

To explore the release mechanism of flower fragrance in more detail, transcriptome sequencing was carried out for 11 time periods. A total of 257,140 unigenes were identified, which exceeded the 124,233 unigenes identified by Hu et al. (2017) and the 118,665 unigenes identified by Shi et al. (2018) in ‘Siberia’, providing data to analyze the molecular mechanisms in the absence of a genome background in *Lilium*. A total of 43,934 DEGs were identified during the 11 periods. By analyzing the different genes in the MEP pathway, the expression trends were the same as those of the release of flower fragrance, which first increased and then decreased. Of these genes, *DXS*, *DXR*, and *MCS* performed the best. Zhang et al. (2018) overexpressed *LiDXS* and *LiDXR* in tobacco and showed that they significantly increased the terpenoid content of tobacco plants. Similarly, we found a significant difference in the MEP pathway in LiTPS2 (TRINITY_69776_co_g1), which has been reported to catalyze the production of ocimene and linalool. Ectopic expression of LiTPS2 in transgenic tobacco plants significantly increases the release of monoterpenes compared with wild-type plants (Zhang et al., 2020). The KEGG analysis of all DEGs suggested that “plant hormone signal transduction” and “monoterpenoid biosynthesis” were the main enriched pathways, including pathways that encoded proteins related to the synthesis of jasmonic acid (JA), auxin, gibberellin (GA), and ethylene (**Supplementary Table 7**). Muhlemann et al. (2014) pointed out that ethylene significantly affected the release of VOCs in *Petunia*. Furthermore, the ethylene response factor RhERF6 has been identified in *Petunia*, which competes for binding of the c-myb domains in EOBI with the promoters of genes related to the production of flower fragrance, thereby negatively regulating the synthesis of VOCs in petunia flowers (Liu et al., 2018). In addition, JA, GA, and abscisic acid have been reported to affect the synthesis of VOCs **(** Kegge et al., 2013; Jasmin et al., 2017; Wu et al., 2018 **)**. We observed previously that spraying JA significantly increases the release of monoterpenes from ‘Siberia’ (Wu et al., 2018). The genes enriched in this pathway provide new insight to explore the mechanism of monoterpenoid release from multiple perspectives.

R2R3-MYB, as a potential regulator of the monoterpene pathway, was identified from the TF dataset based on the WGCNA, isolated by PCR, and designated as *LiMYB305*. *LiMYB305* had high homology with LhMyb (BAB40790.1) in the phylogenetic tree, which was related to anthocyanin biosynthesis in Asiatic hybrid lily. MYB305 knockdown reduces the expression of nectaries and flavonoid biosynthetic genes in the floral nectary (Liu et al., 2009). Subsequently, Liu and Thornburg (2012) reported that MYB305 may also function in the tobacco nectary maturation program by controlling the expression of starch metabolic genes. *OsMYB305* overexpression inhibits cellulose biosynthesis under low nitrogen conditions, thereby releasing carbohydrates for nitrate uptake and assimilation, and promoting rice growth (Wang et al., 2020). In *Lilium longiflorum*, LIMYB305 represses *LIHSC70* promoter activity under normal conditions, but activates promoter activity under heat stress, suggesting that LIMYB305 may need a high temperature to facilitate thermotolerance (Wu et al., 2021). Subsequently, a phylogenetic tree was constructed using the known amino acid sequence of *Arabidopsis* R2R3-MYB (**Supplementary Figure 9**). The results showed that LiMYB305 belongs to subgroup 19 together with AtMYB21, AtMYB24, and AtMYB57 (Dubos et al., 2010). Research on *Arabidopsis* has shown that these three MYBs are involved in stamen development through the JA signaling pathway, but they have functional redundancy (Cheng et al., 2009; Song et al., 2011; Qi et al., 2015). The *atmyb24* and *atmyb57* genes result in no apparent phenotype in wild-type plants, but *atmyb21* causes infertility. A significant decline in fertility is observed in double and triple mutants, and the triple mutation is more serious and causes defects in pollen maturation, anther dehiscence, and filament elongation (Cheng et al., 2009; Song et al., 2011). AtMYB21/24 has been demonstrated to directly regulate the expression of terpene synthase genes, and the effect on linalool has been confirmed (Yang et al., 2020). Snapdragon AmMYB24 interacts with AmCRY1 to activate *AmOCS* by binding to the MYBCOREATCYCB1 motif, thereby increasing the synthesis of ocimene (Han et al., 2022). Therefore, we speculated that LiMYB305 directly binds and regulates terpene synthase.

In this study, LiMYB305 had a potential regulatory role in terpene synthesis, presenting its multi-functionality. As *Lilium* is a bulb flower that is not suitable for stable transformation, VIGS silencing in ‘Siberia’ was utilized to investigate the function of LiMYB305 in regulating monoterpene biosynthesis. The results of monoterpene release and gene expression revealed that LiMYB305 was a monoterpene-related regulator. Subsequently, the *LiOcS* promoter was cloned successfully and further analysis showed that there was a MYBCORE cis-element, which has been confirmed to be specifically bound by FhMYB21 and ATMYB21/24 in *F. hybrida* and *Arabidopsis*, respectively. Our Y1H and dual-luciferase reporter assays demonstrated that *LiOcS* was a direct target of LiMYB305. The EMSA results showed that MYBCORE was the direct binding site. The *LiOcS* sequence was consistent with that of *LoTPS1*, which catalyzes GPP to produce linalool and ocimene (Abbas et al., 2019). Thus, the synthesis of linalool and ocimene was inhibited when *LiMYB305* expression decreased. In addition, many studies have shown that AtMYB21, AtMYB24, and AtMYB57 are involved in regulating JA. Yang et al. (2020) pointed out that MYC2 interacted with FhMYB21 and participates in the regulation of monoterpene synthesis. Combined with our findings that many hormones related to DEGs were enriched, and that spraying MeJA significantly affects the synthesis of flower fragrance (Wu et al., 2018), we boldly speculated that LiMYB305 might also be involved in the JA pathway to affect the production of monoterpenes. But the regulatory network is diverse and complex, which requires us to further study.

## Conclusions

In conclusion, a wealth of transcriptome data was generated, which made up for the lack of a genome. *LiMYB305* was involved in the regulation of monoterpene synthesis and may be a good candidate for engineering the synthesis of monoterpenes. This study provides insight into monoterpenes and useful resources for genetic engineering to produce more commercially important *Lilium* plants.

## Materials and methods

### Plant material and tissue collection

‘Siberia’ bulbs were imported from Holland and cultured in a greenhouse with suitable growth conditions at the Science Park, Beijing University of Agriculture (116°3’14” E, 40°0’95” E). Various floral developmental stages were selected for transcriptome sequencing, including S0–S5: 5–10 cm; S6: bud color changes to white; S7: tepals split slightly; S8: semi-opened tepals; S9: tepals fully-opened; and S10: petal decay. Different tissues (roots, stems, leaves, styles, ovaries, anthers, filaments, and petals) were collected at S9 for qRT-PCR. All of the samples from three biological replicates were collected and immediately frozen at −80°C for further analysis.

### Detection and analysis of VOCs

The VOCs emitted by ‘Siberia’ were collected using the dynamic headspace sampling method as described by Hu et al. (2017). Briefly, an individual flower was placed in a Reynolds oven bag (16 × 17.5 in) to gather the emitted VOCs. A stainless-steel tube (containing Tenax-GR) was used to collect the VOCs for 20 min. The floral VOCs were analyzed by automated thermal desorption-gas chromatography/mass spectrometry, and the compounds were identified by searching the NIST08 and WILEY libraries using TurboMass 5.4.2 software.

### RNA extraction, library preparation, and sequencing

Total RNA was extracted using the TransZol Up Plus RNA kit (TransGen Biotech, Beijing, China) according to the manufacturer’s protocol. The quality and quantity of the purified RNA were evaluated by 1% agarose gel electrophoresis and NanoDrop ND-1000 UV/Visible spectrophotometry (Thermo Fisher Scientific, Waltham, MA, USA). High-quality RNA samples were preserved to construct the cDNA library and for Illumina sequencing.

The libraries were constructed and sequenced at Shanghai Majorbio Bio-pharm Technology Co., Ltd. (Shanghai, China). RNA-seq transcriptome libraries were prepared using the Illumina TruSeq RNA Sample Preparation kit (San Diego, CA) according to the manufacturer’s instructions. Approximately 200–300 bp of cDNA target fragments, as detected by 2% agarose electrophoresis, were selected to construct the library after PCR amplification. After quantification by TBS380, the libraries were sequenced on an Illumina HiSeq Xten/NovaSeq 6000 sequencer for PE 150 of sequencing read length.

### DEG analysis, KEGG enrichment, and identification of co-expression modules

To determine the DEGs between two adjacent samples, the expression level of each transcript was calculated based on the transcript per million reads (TPM) method. Gene abundance was evaluated with the RSEM program (http://deweylab.biostat.wisc.edu/rsem/). The differential expression analysis was performed using DESeq^2^ with a Q value ≤ 0.05. DEGs with |log_2_FC| ≥ 1 and a Q value ≤ 0.05 were significant DEGs. In addition, the statistical enrichment of the DEGs in the KEGG pathways was carried out using the KOBAS program (http://kobas.cbi.pku.edu.cn/home.do). WGCNA was performed on the free online Majorbio Cloud Platform.

### Gene cloning, sequence analysis, and expression analyses

The full-length cDNA sequence of *LiMYB305* was obtained from the transcriptome data. The PCR products were ligated into the pEASYT1-Blunt vector (TransGen Biotech) and sequenced. A phylogenetic tree was constructed using the neighbor-joining method based on 1,000 bootstrap replicates in the MEGA7 program. A gene expression analysis was performed by qRT-PCR with three independent biological replicates for each sample. The SYBR Green qPCR Mix (TaKaRa, Ohtsu, Japan) was used to measure the expression levels on a Bio-Rad CFX96 Real-Time PCR system (BIO-RAD Laboratories, Hercules, CA, USA) according to the manufacturer’s instructions. The results were compared to the *LiActin* internal control and calculated using the 2^−ΔΔCT^ method. All primers are shown in **Supplementary Table 7**.

### Subcellular localization of the LiMYB305 proteins

The coding sequences of *LiMYB305* with the termination codons removed were subcloned into the pBWA(V)HS-GLosgfp vector by the *BsaI*/*Eco31I* restriction enzymes using T4 DNA ligase (TransGen Biotech). The constructs and empty plasmids were transformed into protoplasts isolated from *A. thaliana*, which were visualized under a laser scanning confocal microscope (Leica, Jena, Germany). All primers are shown in **Supplementary Table 7**.

### Functional verification of LiMYB305

A 200-bp fragment of the coding region was selected and cloned into the pNC-TRV2 vector to silence the *LiMYB305* gene in *Lilium* ‘Siberia’. The *Agrobacterium* cell strain GV3101 containing the TRV1 and pNC-TRV2 derivatives (1:1, OD600 = 1.0) was harvested and suspended in infiltration buffer (10 mM MgCl_2_, 200 mM acetosyringone, and 10 mM MES, pH 5.6). A mixture of *Agrobacterium* cultures was injected into the dorsal petals with 1 mL of bacterial suspension per petal using a 1 ml needleless syringe. All primers are shown in **Supplementary Table 7**.

### Promoter cloning and yeast one-hybrid assays

The *LiOcS* promoter (812bp) were amplified using the genomic DNA of ‘Siberia’ by HiTail-PCR following the protocol described as the study of Liu and Chen (2007). The Y1H assay was performed as described in the Yeast Protocol Manual (Clontech Laboratories, Palo Alto, CA, USA). The *LiMYB305* ORF was inserted into the pGADT7 vector, and the *LiOcS* (812 bp) promoter sequence was cloned and inserted into the pHis2 vector. The pGADT7-LiMYB305 and pHis-*LiOcSpro* combination were co-transformed into yeast strain Y187, while the co-transformation of empty pGADT7 vector and the pHis-*LiOcSpro* construct was used as a negative control. All of these strains were plated on SD/-Trp/-Leu/-His/+ 40 mM 3AT to verify the interaction results.

### Dual-luciferase reporter assays

The *LiOcS* promoter sequence was inserted into the pGreenII 0800-Luc vector, and co- transformed with pGreenII 62SK-LiMYB305 or the empty pGreenII 62-SK vector into *N. benthamiana* leaves using *A. tumefaciens* strain GV3101 (pSoup-p19). Two days after transformation, luciferase images were taken using a CCD camera (Andor Technology, Belfast, UK), and firefly luciferase and Renilla luciferase activities were determined using a dual-luciferase reporter assay kit (Promega, Madison, WI, USA) according to manufacturer’s standard protocol. Three biological replicates were used for each treatment.

### Electrophoretic mobility shift assay

Expression of the GST-LiMYB305 fusion protein was induced in 250 mL cultures of transformed *E. coli* BL21 cells using isopropylthio-β-galactoside at a final concentration of 0.4 mM. The EMSA was performed using a Light Shift chemiluminescent EMSA kit (Thermo Fisher) according to the manufacturer’s instructions.

## Data availability statement

The data presented in the study are deposited in the NCBI repository,accession number SRP352614.

## Author contributions

P-SL and Z-HH conceived, designed the experiments, and revised the manuscript. Y-YY, BM, and Y-YL performed the experiments and revised the manuscript. M-ZH, JW, X-FZ, JT, and W-HW analyzed the data. All the authors read and approved the final manuscript.

## Funding

This work was supported by the National Key R&D Program of China (2018YFD1000406), the Joint Project of Beijing Municipal Education Commission and Beijing Municipal Natural Science Foundation (KZ201810020028), and the Construction of Beijing Science and Technology Innovation and Service Capacity in Top Subjects (CEFF-PXM2019_014207_000032).

## Conflict of interest

The authors declare that the research was conducted in the absence of any commercial or financial relationships that could be construed as a potential conflict of interest.

## Publisher’s note

All claims expressed in this article are solely those of the authors and do not necessarily represent those of their affiliated organizations, or those of the publisher, the editors and the reviewers. Any product that may be evaluated in this article, or claim that may be made by its manufacturer, is not guaranteed or endorsed by the publisher.
