# Supplementary material for: Transcriptome analysis identifies key gene LiMYB305 involved in monoterpene biosynthesis in Lilium ‘Siberia’
Source: Front Plant Sci. 2022 Nov 7;13:1021576. doi: 10.3389/fpls.2022.1021576 (PMC9677127; doi:10.3389/fpls.2022.1021576)
Supplement: Supplementary file 1 [file DataSheet_1.docx]

Supplementary Material

## Supplementary Figures


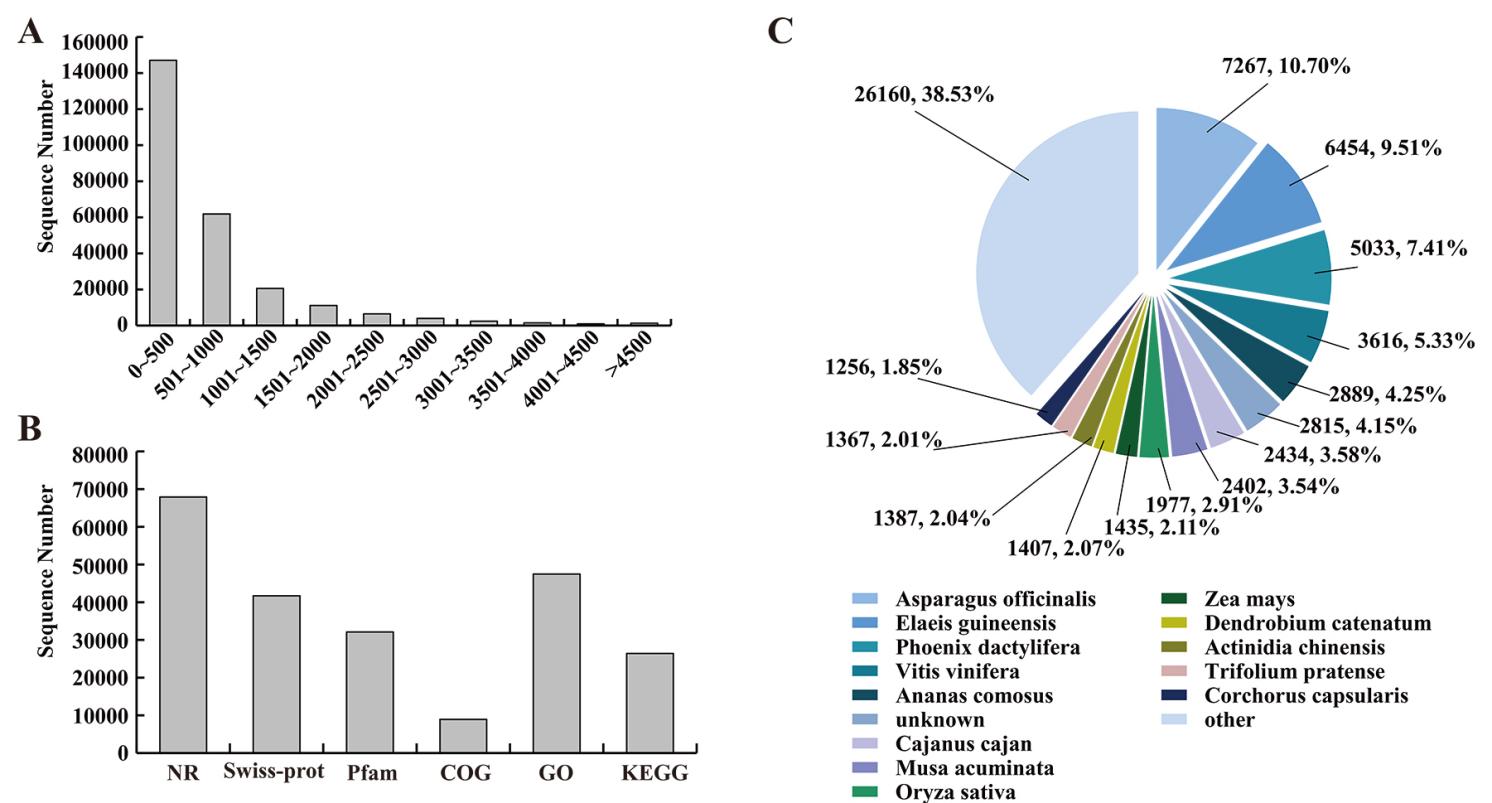


**Supplementary Figure 1** Expression profiles of RNA-seq data during the stages of flower development in *Lilium* 'Seberia'. A. sequence length distribution in 11 flower developmental stages. B. annotation analysis of unigenes. C. NR annotated species distribution.


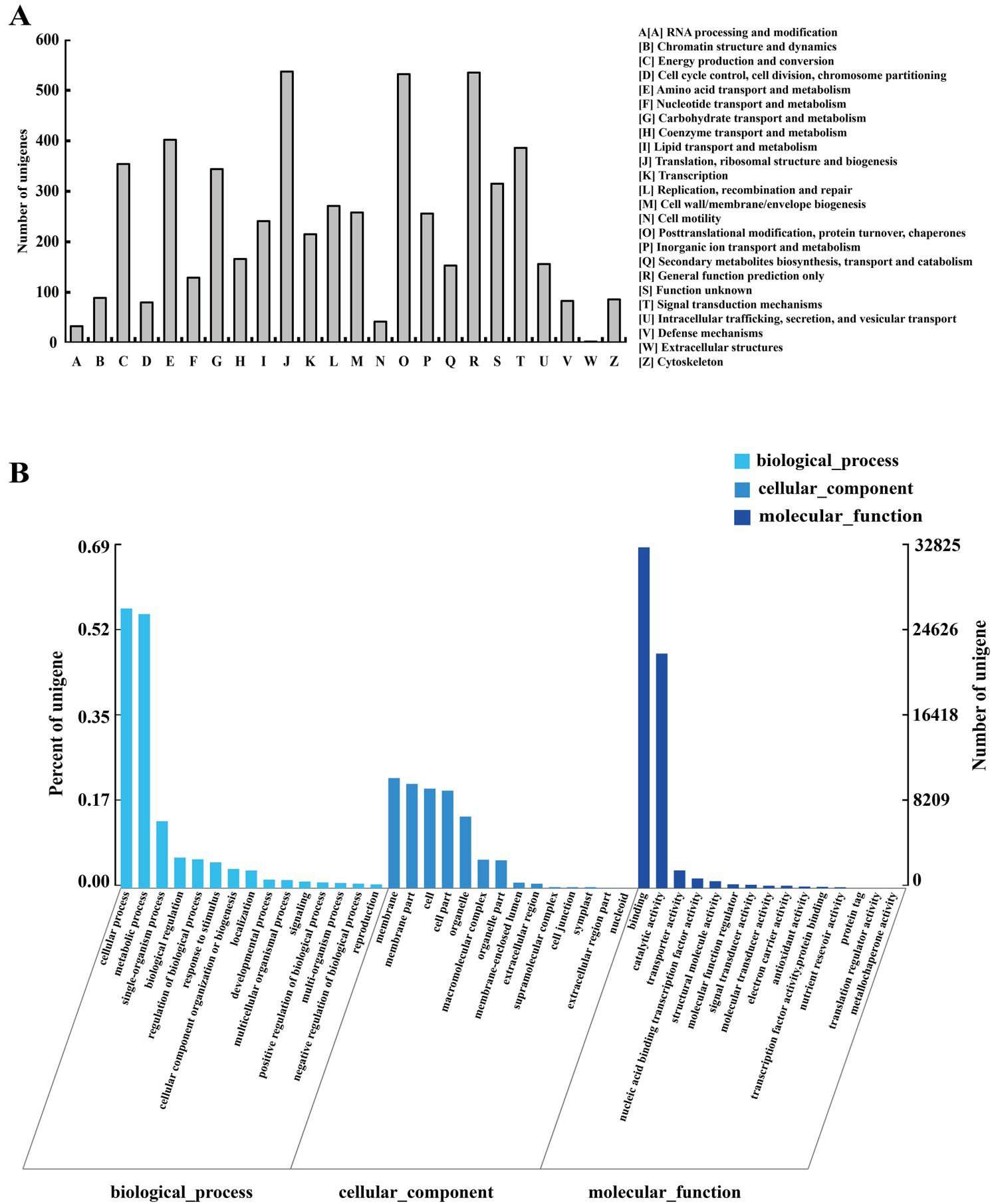


**Supplementary Figure 2** Cluster of orthologous groups (COG) functional classification and histogram presentation of Gene Ontology (GO) classifications of unigenes.


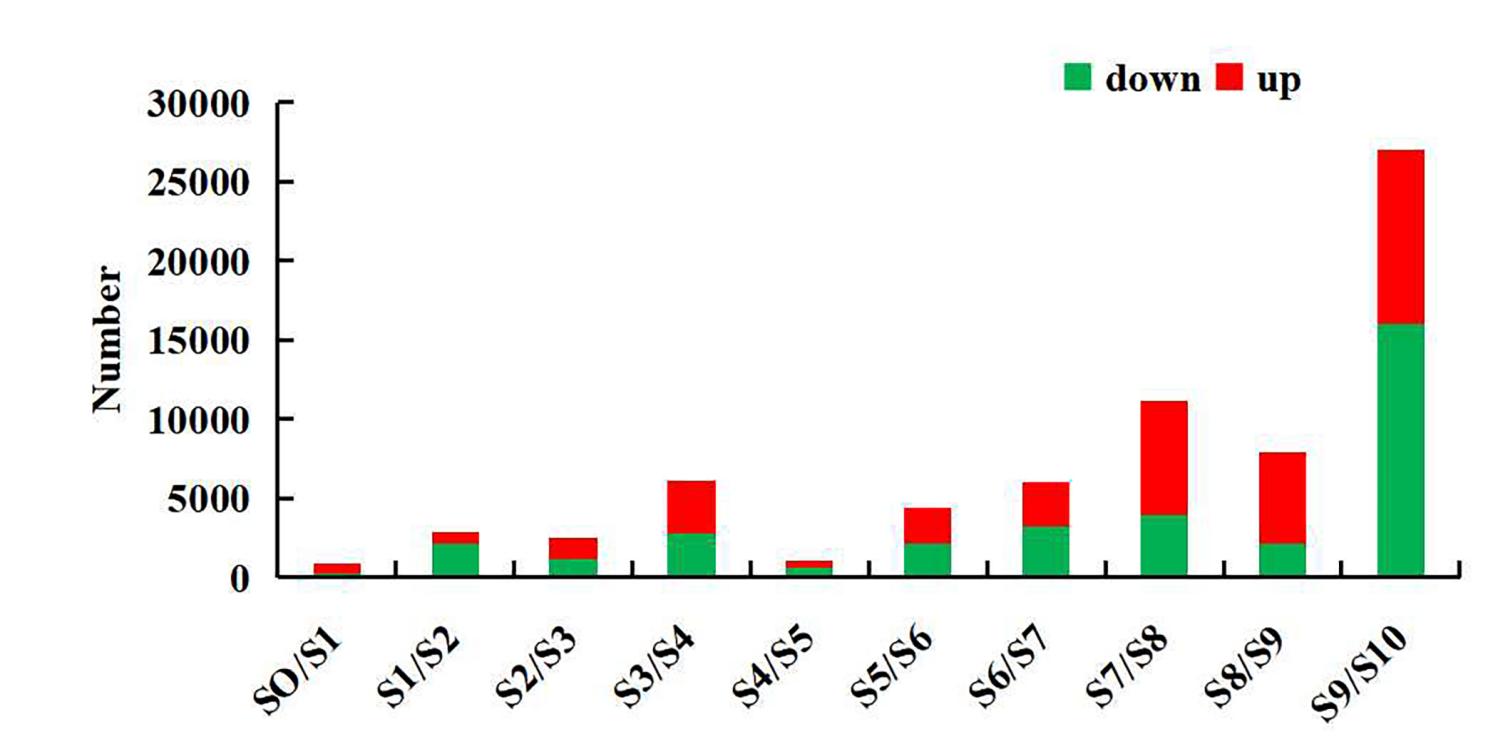


**Supplementary Figure 3** DEGs analyses of 11 development stages. Red indicates an up-regulated gene, and green indicates a down-regulated gene.


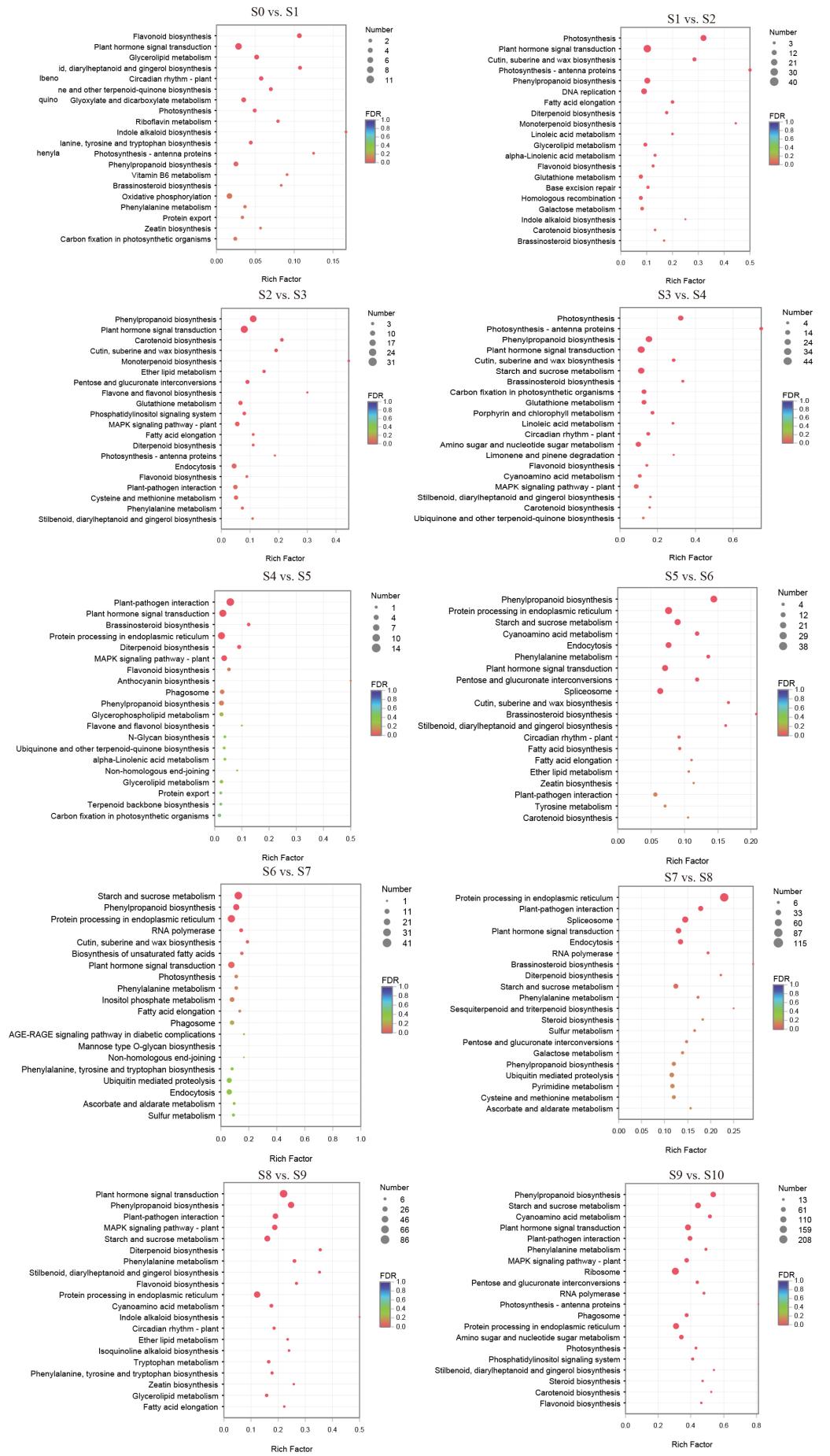


**Supplementary Figure 4**  KEGG analysis of DEGs identified from every two adjacent stages between developmental stages.

**
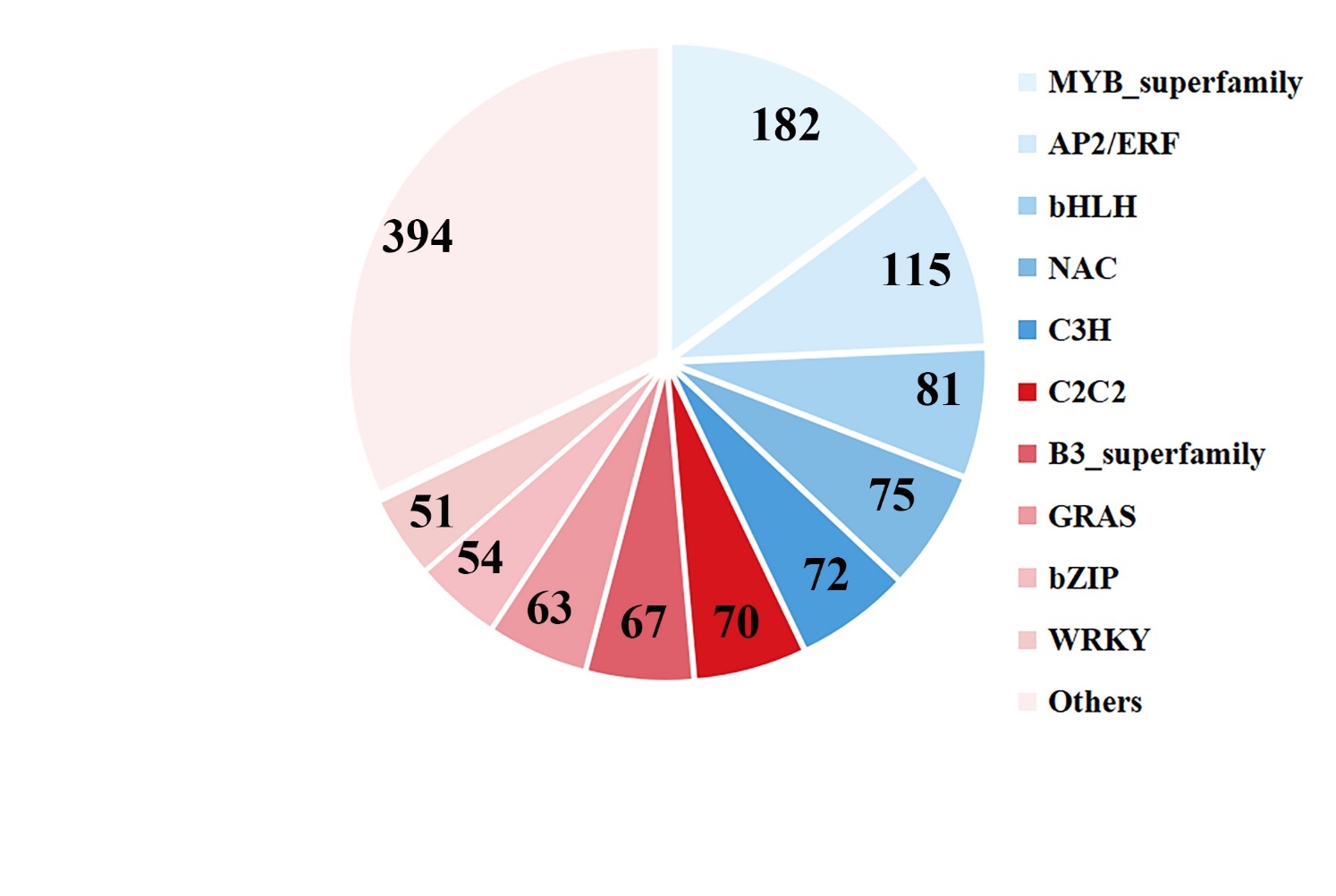
**

**Supplementary Figure 5** Numbers of top ten predicted TFs.


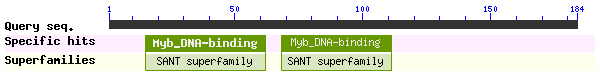


**Supplementary Figure 6.** Characterization of ‘Siberia’ LlMYB305 protein. LlMYB305 is an R2R3-MYB protein with two conserved SANT domains between 15-62 and 68-111 amino acids, respectively.


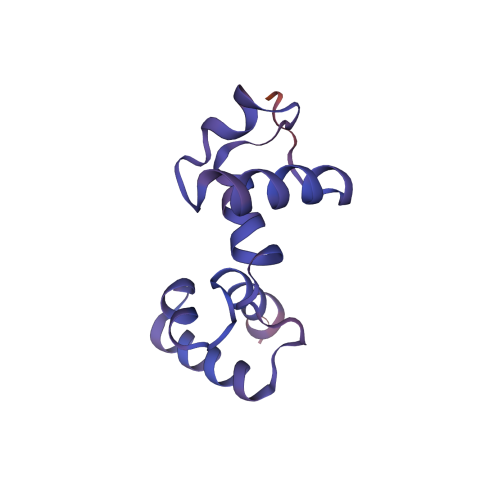


**Supplementary Figure 7** 3D structure simulation of LiMYB305 by using ExPASy.


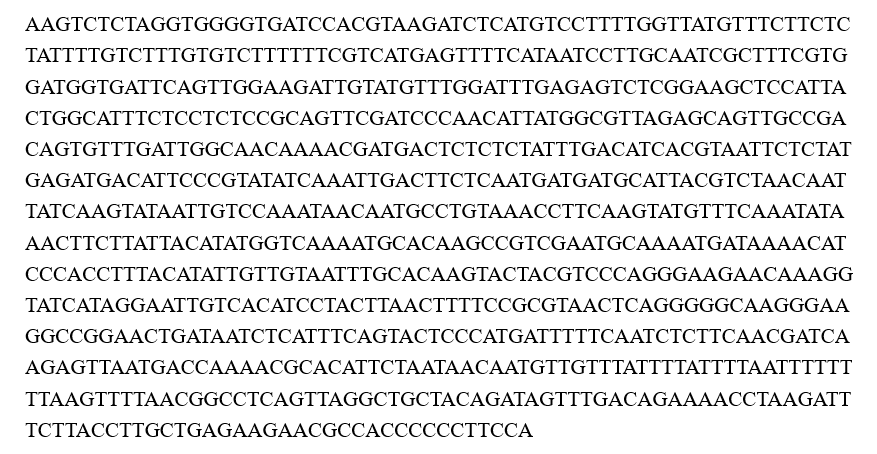


**Supplementary Figure 8** Promoter sequence of *LiOcS.*


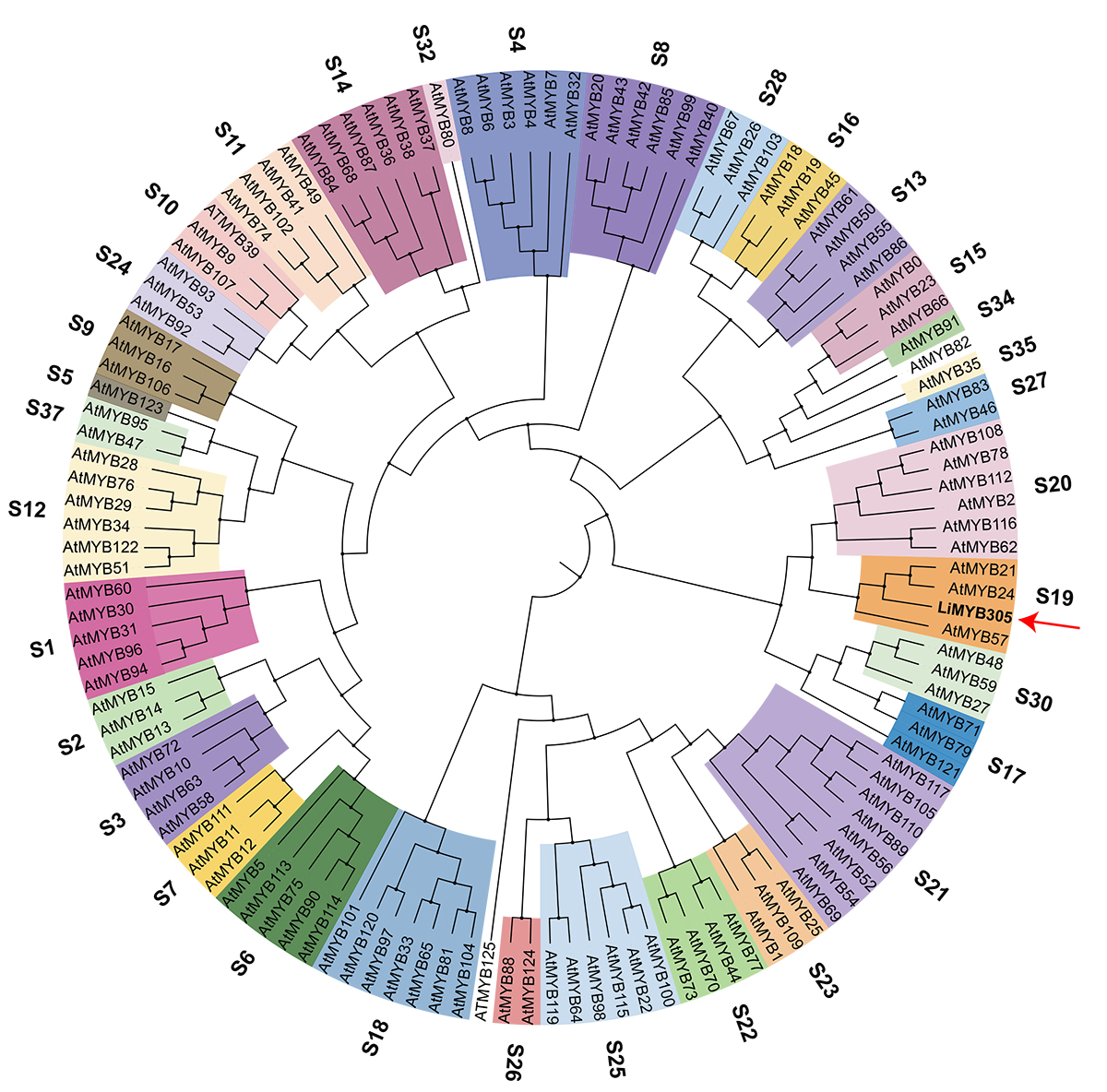


**Supplementary Figure 9** Phylogenetic relationship of LiMYB305 with all R2R3-MYB proteins from *Arabidopsis*. The Phylogenetic tree was constructed by iTOL online tool (https://itol.embl.de/tree/) with the default parameters. The amino acid sequences of the *Arabidopsis* MYBs were downloaded from TAIR (<https://www.arabidopsis.org/>).

**Supplementary Table 1.** RNA sequencing data and corresponding quality control

| **Sample** | **Raw reads** | **Raw bases** | **Clean reads** | **Clean bases** | **Error rate(%)** | **Q30**  **(%)** | **Mapped ratio** |
| --- | --- | --- | --- | --- | --- | --- | --- |
| S0_1 | 108,791,500 | 16,427,516,500 | 107,260,628 | 16,055,577,618 | 0.025 | 94.04 | 71.18% |
| S0_2 | 89,068,412 | 13,449,330,212 | 87,905,814 | 13,164,814,139 | 0.025 | 94.14 | 70.81% |
| S0_3 | 99,091,390 | 14,962,799,890 | 97,598,336 | 14,582,408,375 | 0.0254 | 93.7 | 71.22% |
| S1_1 | 94,671,222 | 14,295,354,522 | 93,277,668 | 13,966,379,492 | 0.025 | 94.08 | 71.57% |
| S1_2 | 86,488,396 | 13,059,747,796 | 85,122,276 | 12,732,255,540 | 0.0251 | 94.04 | 71.10% |
| S1_3 | 108,014,726 | 16,310,223,626 | 106,653,728 | 15,953,756,908 | 0.0253 | 93.77 | 70.96% |
| S2_1 | 99,773,108 | 15,065,739,308 | 98,159,486 | 14,684,632,761 | 0.0256 | 93.54 | 70.47% |
| S2_2 | 105,107,630 | 15,871,252,130 | 103,614,260 | 15,498,808,442 | 0.025 | 94.09 | 80.42% |
| S2_3 | 97,675,822 | 14,749,049,122 | 96,478,926 | 14,445,166,611 | 0.0252 | 93.91 | 71.98% |
| S3_1 | 110,249,340 | 16,647,650,340 | 108,725,396 | 16,265,620,608 | 0.0252 | 93.89 | 71.67% |
| S3_2 | 89,611,384 | 13,531,318,984 | 88,303,756 | 13,228,171,833 | 0.0258 | 93.35 | 70.59% |
| S3_3 | 95,184,424 | 14,372,848,024 | 93,930,536 | 14,060,159,907 | 0.0248 | 94.28 | 71.64% |
| S4_1 | 107,204,552 | 16,187,887,352 | 105,883,062 | 15,848,596,291 | 0.0254 | 93.72 | 72.16% |
| S4_2 | 98,657,108 | 14,897,223,308 | 97,489,704 | 14,602,048,329 | 0.0249 | 94.18 | 71.60% |
| S4_3 | 104,914,152 | 15,842,036,952 | 103,355,802 | 15,458,373,908 | 0.025 | 94.14 | 67.81% |
| S5_1 | 100,512,600 | 15,177,402,600 | 99,266,850 | 14,851,075,201 | 0.0249 | 94.18 | 70.95% |
| S5_2 | 96,344,978 | 14,548,091,678 | 94,926,594 | 14,203,786,101 | 0.025 | 94.08 | 71.73% |
| S5_3 | 98,830,520 | 14,923,408,520 | 97,435,580 | 14,574,040,623 | 0.0253 | 93.84 | 72.63% |
| S6_1 | 103,071,268 | 15,563,761,468 | 101,723,122 | 15,211,961,395 | 0.025 | 94.09 | 71.60% |
| S6_2 | 101,140,418 | 15,272,203,118 | 99,769,442 | 14,929,499,576 | 0.0251 | 94.01 | 71.31% |
| S6_3 | 93,706,908 | 14,149,743,108 | 91,939,570 | 13,723,907,873 | 0.0253 | 93.83 | 68.07% |
| S7_1 | 95,496,642 | 14,419,992,942 | 93,849,598 | 13,976,,777291 | 0.0254 | 93.68 | 68.37% |
| S7_2 | 106,535,190 | 16,086,813,690 | 105,020,132 | 15,700,326,089 | 0.025 | 94.13 | 70.89% |
| S7_3 | 96,268,740 | 14,536,579,740 | 94,367,310 | 14,063,352,535 | 0.0276 | 91.67 | 69.36% |
| S8_1 | 106,803,876 | 16,127,385,276 | 105,545,244 | 15,762,698,138 | 0.0252 | 93.89 | 73.44% |
| S8_2 | 93,310,102 | 14,089,825,402 | 91,827,520 | 13,718,373,602 | 0.0254 | 93.67 | 68.85% |
| S8_3 | 103,399,080 | 15,613,261,080 | 101,848,224 | 15,190,676,139 | 0.0253 | 93.81 | 70.19% |
| S9_1 | 88,335,762 | 13,338,700,062 | 87,051,552 | 13,017,599,141 | 0.0255 | 93.64 | 72.57% |
| S9_2 | 105,777,850 | 15,972,455,350 | 104,264,538 | 15,550,048,622 | 0.0247 | 94.38 | 66.76% |
| S9_3 | 99,885,758 | 15,082,749,458 | 98,526,362 | 14,722,803,074 | 0.0248 | 94.26 | 69.73% |
| S10_1 | 91,946,926 | 13,883,985,826 | 90,685,774 | 13,574,050,589 | 0.0251 | 94.03 | 71.01% |
| S10_2 | 94,115,198 | 14,211,394,898 | 92,757,626 | 13,868,087,052 | 0.0252 | 93.93 | 71.65% |
| S10_3 | 92,658,294 | 13,991,402,394 | 91,161,558 | 13,629,346,286 | 0.0254 | 93.7 | 71.42% |

**Supplementary Table 2.** Summary of the *Lilium* 'Siberia' transcriptome

| **Type** | **Resource** |
| --- | --- |
| Total transcripts number | 373,921 |
| Total unigenes number | 257,140 |
| Total sequence base | 296,830,607 |
| Largest | 20,898 |
| Smallest | 201 |
| Average length | 794 |
| N50 | 1,172 |
| E90N50 | 1,832 |
| GC percent | 42.88 |
| Mean mapped reads | 3601 |
| TransRate score | 0.28 |

**Supplementary Table 3.** KEGG analysis of all DEGs

| **Num** | **Pathway id** | **Description** | **Database** | **Ratio_in_study** | **Ratio_in_pop** | **Pvalue_uncorrected** | **Pvalue_corrected** | **First Category** | **Second Category** |
| --- | --- | --- | --- | --- | --- | --- | --- | --- | --- |
| 246 | map04075 | Plant hormone signal transduction | KEGG PATHWAY | 246/8804 | 392/26412 | 3.91612E-33 | 5.01263E-31 | Environmental Information Processing | Signal transduction |
| 169 | map00940 | Phenylpropanoid biosynthesis | KEGG PATHWAY | 169/8804 | 243/26412 | 5.81162E-31 | 3.71944E-29 | Metabolism | Biosynthesis of other secondary metabolites |
| 186 | map00500 | Starch and sucrose metabolism | KEGG PATHWAY | 186/8804 | 324/26412 | 3.63159E-19 | 1.54948E-17 | Metabolism | Carbohydrate metabolism |
| 250 | map04141 | Protein processing in endoplasmic reticulum | KEGG PATHWAY | 250/8804 | 501/26412 | 8.41012E-15 | 2.69124E-13 | Genetic Information Processing | Folding, sorting and degradation |
| 139 | map04626 | Plant-pathogen interaction | KEGG PATHWAY | 139/8804 | 248/26412 | 1.41921E-13 | 3.63317E-12 | Organismal Systems | Environmental adaptation |
| 128 | map04016 | MAPK signaling pathway - plant | KEGG PATHWAY | 128/8804 | 235/26412 | 1.88522E-11 | 4.02181E-10 | Environmental Information Processing | Signal transduction |
| 76 | map00460 | Cyanoamino acid metabolism | KEGG PATHWAY | 76/8804 | 126/26412 | 4.73744E-10 | 8.66274E-09 | Metabolism | Metabolism of other amino acids |
| 35 | map00904 | Diterpenoid biosynthesis | KEGG PATHWAY | 35/8804 | 45/26412 | 1.23829E-09 | 1.98126E-08 | Metabolism | Metabolism of terpenoids and polyketides |
| 52 | map00195 | Photosynthesis | KEGG PATHWAY | 52/8804 | 81/26412 | 1.27632E-08 | 1.81521E-07 | Metabolism | Energy metabolism |
| 127 | map00520 | Amino sugar and nucleotide sugar metabolism | KEGG PATHWAY | 127/8804 | 263/26412 | 3.2807E-07 | 4.19929E-06 | Metabolism | Carbohydrate metabolism |
| 28 | map00906 | Carotenoid biosynthesis | KEGG PATHWAY | 28/8804 | 38/26412 | 4.22452E-07 | 4.91581E-06 | Metabolism | Metabolism of terpenoids and polyketides |
| 30 | map00073 | Cutin, suberine and wax biosynthesis | KEGG PATHWAY | 30/8804 | 42/26412 | 4.99518E-07 | 4.91833E-06 | Metabolism | Lipid metabolism |
| 49 | map00360 | Phenylalanine metabolism | KEGG PATHWAY | 49/8804 | 81/26412 | 4.90168E-07 | 5.22846E-06 | Metabolism | Amino acid metabolism |
| 107 | map00270 | Cysteine and methionine metabolism | KEGG PATHWAY | 107/8804 | 217/26412 | 7.61944E-07 | 6.96634E-06 | Metabolism | Amino acid metabolism |
| 61 | map00040 | Pentose and glucuronate interconversions | KEGG PATHWAY | 61/8804 | 109/26412 | 9.71979E-07 | 8.29422E-06 | Metabolism | Carbohydrate metabolism |
| 91 | map04145 | Phagosome | KEGG PATHWAY | 91/8804 | 180/26412 | 1.28125E-06 | 1.025E-05 | Cellular Processes | Transport and catabolism |
| 46 | map03020 | RNA polymerase | KEGG PATHWAY | 46/8804 | 77/26412 | 1.79265E-06 | 1.34976E-05 | Genetic Information Processing | Transcription |
| 125 | map00240 | Pyrimidine metabolism | KEGG PATHWAY | 125/8804 | 267/26412 | 3.05516E-06 | 2.17256E-05 | Metabolism | Nucleotide metabolism |
| 26 | map00945 | Stilbenoid, diarylheptanoid and gingerol biosynthesis | KEGG PATHWAY | 26/8804 | 37/26412 | 4.77336E-06 | 3.21573E-05 | Metabolism | Biosynthesis of other secondary metabolites |
| 55 | map04070 | Phosphatidylinositol signaling system | KEGG PATHWAY | 55/8804 | 100/26412 | 6.63046E-06 | 4.24349E-05 | Environmental Information Processing | Signal transduction |
| 35 | map00941 | Flavonoid biosynthesis | KEGG PATHWAY | 35/8804 | 56/26412 | 7.36697E-06 | 4.49034E-05 | Metabolism | Biosynthesis of other secondary metabolites |
| 61 | map00561 | Glycerolipid metabolism | KEGG PATHWAY | 61/8804 | 115/26412 | 1.01229E-05 | 5.88969E-05 | Metabolism | Lipid metabolism |
| 14 | map00196 | Photosynthesis - antenna proteins | KEGG PATHWAY | 14/8804 | 16/26412 | 1.18465E-05 | 6.59284E-05 | Metabolism | Energy metabolism |
| 19 | map00591 | Linoleic acid metabolism | KEGG PATHWAY | 19/8804 | 25/26412 | 1.55021E-05 | 7.93706E-05 | Metabolism | Lipid metabolism |
| 33 | map00592 | alpha-Linolenic acid metabolism | KEGG PATHWAY | 33/8804 | 53/26412 | 1.50234E-05 | 8.01249E-05 | Metabolism | Lipid metabolism |
| 80 | map00564 | Glycerophospholipid metabolism | KEGG PATHWAY | 80/8804 | 162/26412 | 1.65975E-05 | 8.17107E-05 | Metabolism | Lipid metabolism |
| 30 | map00565 | Ether lipid metabolism | KEGG PATHWAY | 30/8804 | 47/26412 | 1.80953E-05 | 8.57851E-05 | Metabolism | Lipid metabolism |
| 29 | map00062 | Fatty acid elongation | KEGG PATHWAY | 29/8804 | 45/26412 | 1.90324E-05 | 8.70051E-05 | Metabolism | Lipid metabolism |
| 48 | map04712 | Circadian rhythm - plant | KEGG PATHWAY | 48/8804 | 87/26412 | 2.24051E-05 | 9.88914E-05 | Organismal Systems | Environmental adaptation |
| 104 | map00620 | Pyruvate metabolism | KEGG PATHWAY | 104/8804 | 224/26412 | 3.03029E-05 | 0.000129293 | Metabolism | Carbohydrate metabolism |
| 33 | map00100 | Steroid biosynthesis | KEGG PATHWAY | 33/8804 | 55/26412 | 4.47409E-05 | 0.000184737 | Metabolism | Lipid metabolism |
| 37 | map00053 | Ascorbate and aldarate metabolism | KEGG PATHWAY | 37/8804 | 64/26412 | 4.94488E-05 | 0.000197795 | Metabolism | Carbohydrate metabolism |
| 59 | map00330 | Arginine and proline metabolism | KEGG PATHWAY | 59/8804 | 115/26412 | 5.10627E-05 | 0.000198061 | Metabolism | Amino acid metabolism |
| 148 | map04144 | Endocytosis | KEGG PATHWAY | 148/8804 | 342/26412 | 7.32342E-05 | 0.000275705 | Cellular Processes | Transport and catabolism |
| 28 | map00950 | Isoquinoline alkaloid biosynthesis | KEGG PATHWAY | 28/8804 | 46/26412 | 0.000117453 | 0.000429541 | Metabolism | Biosynthesis of other secondary metabolites |
| 77 | map00710 | Carbon fixation in photosynthetic organisms | KEGG PATHWAY | 77/8804 | 164/26412 | 0.000196765 | 0.000699609 | Metabolism | Energy metabolism |
| 45 | map00061 | Fatty acid biosynthesis | KEGG PATHWAY | 45/8804 | 86/26412 | 0.000212436 | 0.000734912 | Metabolism | Lipid metabolism |
| 41 | map03410 | Base excision repair | KEGG PATHWAY | 41/8804 | 77/26412 | 0.000245138 | 0.000825729 | Genetic Information Processing | Replication and repair |
| 77 | map00480 | Glutathione metabolism | KEGG PATHWAY | 77/8804 | 166/26412 | 0.000314826 | 0.001033275 | Metabolism | Metabolism of other amino acids |
| 32 | map00130 | Ubiquinone and other terpenoid-quinone biosynthesis | KEGG PATHWAY | 32/8804 | 57/26412 | 0.000329486 | 0.001054354 | Metabolism | Metabolism of cofactors and vitamins |
| 46 | map00400 | Phenylalanine, tyrosine and tryptophan biosynthesis | KEGG PATHWAY | 46/8804 | 90/26412 | 0.000365942 | 0.001142453 | Metabolism | Amino acid metabolism |
| 16 | map00905 | Brassinosteroid biosynthesis | KEGG PATHWAY | 16/8804 | 24/26412 | 0.000855048 | 0.002605861 | Metabolism | Metabolism of terpenoids and polyketides |
| 8 | map00902 | Monoterpenoid biosynthesis | KEGG PATHWAY | 8/8804 | 9/26412 | 0.0009635 | 0.002868094 | Metabolism | Metabolism of terpenoids and polyketides |
| 29 | map01040 | Biosynthesis of unsaturated fatty acids | KEGG PATHWAY | 29/8804 | 53/26412 | 0.001082282 | 0.003148457 | Metabolism | Lipid metabolism |
| 47 | map00350 | Tyrosine metabolism | KEGG PATHWAY | 47/8804 | 98/26412 | 0.00185755 | 0.005283699 | Metabolism | Amino acid metabolism |
| 60 | map00051 | Fructose and mannose metabolism | KEGG PATHWAY | 60/8804 | 132/26412 | 0.00246313 | 0.006853928 | Metabolism | Carbohydrate metabolism |
| 67 | map04146 | Peroxisome | KEGG PATHWAY | 67/8804 | 152/26412 | 0.003645969 | 0.009929448 | Cellular Processes | Transport and catabolism |
| 28 | map00510 | N-Glycan biosynthesis | KEGG PATHWAY | 28/8804 | 54/26412 | 0.003750264 | 0.010000705 | Metabolism | Glycan biosynthesis and metabolism |
| 55 | map00052 | Galactose metabolism | KEGG PATHWAY | 55/8804 | 122/26412 | 0.004496023 | 0.011744713 | Metabolism | Carbohydrate metabolism |
| 41 | map00410 | beta-Alanine metabolism | KEGG PATHWAY | 41/8804 | 87/26412 | 0.005168971 | 0.013232565 | Metabolism | Metabolism of other amino acids |
| 27 | map00450 | Selenocompound metabolism | KEGG PATHWAY | 27/8804 | 53/26412 | 0.005984541 | 0.015020024 | Metabolism | Metabolism of other amino acids |
| 19 | map00908 | Zeatin biosynthesis | KEGG PATHWAY | 19/8804 | 35/26412 | 0.008498551 | 0.020524802 | Metabolism | Metabolism of terpenoids and polyketides |
| 41 | map00900 | Terpenoid backbone biosynthesis | KEGG PATHWAY | 41/8804 | 89/26412 | 0.008360576 | 0.02057988 | Metabolism | Metabolism of terpenoids and polyketides |
| 121 | map04120 | Ubiquitin mediated proteolysis | KEGG PATHWAY | 121/8804 | 303/26412 | 0.009066493 | 0.021490945 | Genetic Information Processing | Folding, sorting and degradation |
| 21 | map00600 | Sphingolipid metabolism | KEGG PATHWAY | 21/8804 | 40/26412 | 0.009504506 | 0.022119578 | Metabolism | Lipid metabolism |
| 169 | map03040 | Spliceosome | KEGG PATHWAY | 169/8804 | 437/26412 | 0.010317666 | 0.023583236 | Genetic Information Processing | Transcription |
| 11 | map04933 | AGE-RAGE signaling pathway in diabetic complications | KEGG PATHWAY | 11/8804 | 18/26412 | 0.014405166 | 0.032348444 | Human Diseases | Endocrine and metabolic disease |
| 9 | map00903 | Limonene and pinene degradation | KEGG PATHWAY | 9/8804 | 14/26412 | 0.017408633 | 0.038419053 | Metabolism | Metabolism of terpenoids and polyketides |
| 21 | map00960 | Tropane, piperidine and pyridine alkaloid biosynthesis | KEGG PATHWAY | 21/8804 | 42/26412 | 0.018595036 | 0.040341774 | Metabolism | Biosynthesis of other secondary metabolites |
| 7 | map00944 | Flavone and flavonol biosynthesis | KEGG PATHWAY | 7/8804 | 10/26412 | 0.019642249 | 0.041903465 | Metabolism | Biosynthesis of other secondary metabolites |
| 15 | map00660 | C5-Branched dibasic acid metabolism | KEGG PATHWAY | 15/8804 | 28/26412 | 0.021504583 | 0.045124371 | Metabolism | Carbohydrate metabolism |
| 178 | map00190 | Oxidative phosphorylation | KEGG PATHWAY | 178/8804 | 471/26412 | 0.02237075 | 0.046184775 | Metabolism | Energy metabolism |
| 49 | map00380 | Tryptophan metabolism | KEGG PATHWAY | 49/8804 | 115/26412 | 0.023405327 | 0.047553681 | Metabolism | Amino acid metabolism |
| 251 | map03010 | Ribosome | KEGG PATHWAY | 251/8804 | 679/26412 | 0.023782548 | 0.047565096 | Genetic Information Processing | Translation |
| 47 | map00071 | Fatty acid degradation | KEGG PATHWAY | 47/8804 | 110/26412 | 0.024659739 | 0.048560717 | Metabolism | Lipid metabolism |
| 34 | map00220 | Arginine biosynthesis | KEGG PATHWAY | 34/8804 | 76/26412 | 0.025075971 | 0.048632187 | Metabolism | Amino acid metabolism |
| 13 | map00909 | Sesquiterpenoid and triterpenoid biosynthesis | KEGG PATHWAY | 13/8804 | 24/26412 | 0.028383225 | 0.054224668 | Metabolism | Metabolism of terpenoids and polyketides |
| 50 | map00250 | Alanine, aspartate and glutamate metabolism | KEGG PATHWAY | 50/8804 | 119/26412 | 0.029200831 | 0.054966269 | Metabolism | Amino acid metabolism |
| 25 | map00290 | Valine, leucine and isoleucine biosynthesis | KEGG PATHWAY | 25/8804 | 54/26412 | 0.032343237 | 0.059999049 | Metabolism | Amino acid metabolism |
| 21 | map00511 | Other glycan degradation | KEGG PATHWAY | 21/8804 | 44/26412 | 0.033304471 | 0.060899603 | Metabolism | Glycan biosynthesis and metabolism |
| 33 | map00860 | Porphyrin and chlorophyll metabolism | KEGG PATHWAY | 33/8804 | 75/26412 | 0.034845233 | 0.062819575 | Metabolism | Metabolism of cofactors and vitamins |
| 53 | map00030 | Pentose phosphate pathway | KEGG PATHWAY | 53/8804 | 129/26412 | 0.039218893 | 0.069722477 | Metabolism | Carbohydrate metabolism |
| 84 | map03018 | RNA degradation | KEGG PATHWAY | 84/8804 | 215/26412 | 0.044069858 | 0.077273175 | Genetic Information Processing | Folding, sorting and degradation |
| 72 | map00020 | Citrate cycle (TCA cycle) | KEGG PATHWAY | 72/8804 | 182/26412 | 0.045050149 | 0.077924582 | Metabolism | Carbohydrate metabolism |
| 9 | map00603 | Glycosphingolipid biosynthesis - globo and isoglobo series | KEGG PATHWAY | 9/8804 | 16/26412 | 0.049910315 | 0.085180272 | Metabolism | Glycan biosynthesis and metabolism |
| 31 | map00310 | Lysine degradation | KEGG PATHWAY | 31/8804 | 73/26412 | 0.064505585 | 0.108640985 | Metabolism | Amino acid metabolism |
| 7 | map00901 | Indole alkaloid biosynthesis | KEGG PATHWAY | 7/8804 | 12/26412 | 0.066403397 | 0.110384868 | Metabolism | Biosynthesis of other secondary metabolites |
| 21 | map04130 | SNARE interactions in vesicular transport | KEGG PATHWAY | 21/8804 | 47/26412 | 0.069400479 | 0.113887966 | Genetic Information Processing | Folding, sorting and degradation |
| 16 | map00780 | Biotin metabolism | KEGG PATHWAY | 16/8804 | 35/26412 | 0.086601943 | 0.140317072 | Metabolism | Metabolism of cofactors and vitamins |
| 5 | map00966 | Glucosinolate biosynthesis | KEGG PATHWAY | 5/8804 | 8/26412 | 0.087911591 | 0.140658546 | Metabolism | Biosynthesis of other secondary metabolites |
| 17 | map00740 | Riboflavin metabolism | KEGG PATHWAY | 17/8804 | 38/26412 | 0.095229479 | 0.15048609 | Metabolism | Metabolism of cofactors and vitamins |
| 15 | map00300 | Lysine biosynthesis | KEGG PATHWAY | 15/8804 | 33/26412 | 0.099761457 | 0.155725202 | Metabolism | Amino acid metabolism |
| 32 | map00920 | Sulfur metabolism | KEGG PATHWAY | 32/8804 | 79/26412 | 0.109494246 | 0.168858596 | Metabolism | Energy metabolism |
| 3 | map00965 | Betalain biosynthesis | KEGG PATHWAY | 3/8804 | 4/26412 | 0.111094282 | 0.169286526 | Metabolism | Biosynthesis of other secondary metabolites |
| 145 | map00010 | Glycolysis / Gluconeogenesis | KEGG PATHWAY | 145/8804 | 400/26412 | 0.116781371 | 0.175859006 | Metabolism | Carbohydrate metabolism |
| 16 | map00531 | Glycosaminoglycan degradation | KEGG PATHWAY | 16/8804 | 37/26412 | 0.135202569 | 0.20123173 | Metabolism | Glycan biosynthesis and metabolism |
| 10 | map00750 | Vitamin B6 metabolism | KEGG PATHWAY | 10/8804 | 22/26412 | 0.163049318 | 0.239888652 | Metabolism | Metabolism of cofactors and vitamins |
| 11 | map00261 | Monobactam biosynthesis | KEGG PATHWAY | 11/8804 | 25/26412 | 0.177877604 | 0.25582397 | Metabolism | Biosynthesis of other secondary metabolites |
| 6 | map00604 | Glycosphingolipid biosynthesis - ganglio series | KEGG PATHWAY | 6/8804 | 12/26412 | 0.177671886 | 0.258431834 | Metabolism | Glycan biosynthesis and metabolism |
| 23 | map00760 | Nicotinate and nicotinamide metabolism | KEGG PATHWAY | 23/8804 | 58/26412 | 0.187701916 | 0.266953836 | Metabolism | Metabolism of cofactors and vitamins |
| 66 | map00260 | Glycine, serine and threonine metabolism | KEGG PATHWAY | 66/8804 | 180/26412 | 0.190880279 | 0.268490942 | Metabolism | Amino acid metabolism |
| 63 | map00630 | Glyoxylate and dicarboxylate metabolism | KEGG PATHWAY | 63/8804 | 172/26412 | 0.200165884 | 0.278491665 | Metabolism | Carbohydrate metabolism |
| 56 | map03440 | Homologous recombination | KEGG PATHWAY | 56/8804 | 155/26412 | 0.254540607 | 0.350335459 | Genetic Information Processing | Replication and repair |
| 51 | map00280 | Valine, leucine and isoleucine degradation | KEGG PATHWAY | 51/8804 | 141/26412 | 0.263456844 | 0.354973432 | Metabolism | Amino acid metabolism |
| 70 | map00562 | Inositol phosphate metabolism | KEGG PATHWAY | 70/8804 | 196/26412 | 0.261560286 | 0.356167197 | Metabolism | Carbohydrate metabolism |
| 155 | map03013 | RNA transport | KEGG PATHWAY | 155/8804 | 446/26412 | 0.276113328 | 0.368151105 | Genetic Information Processing | Translation |
| 8 | map04122 | Sulfur relay system | KEGG PATHWAY | 8/8804 | 19/26412 | 0.279276117 | 0.368529309 | Genetic Information Processing | Folding, sorting and degradation |
| 33 | map03060 | Protein export | KEGG PATHWAY | 33/8804 | 90/26412 | 0.284968398 | 0.372203621 | Genetic Information Processing | Folding, sorting and degradation |
| 22 | map00910 | Nitrogen metabolism | KEGG PATHWAY | 22/8804 | 59/26412 | 0.3023036 | 0.39085718 | Metabolism | Energy metabolism |
| 19 | map00770 | Pantothenate and CoA biosynthesis | KEGG PATHWAY | 19/8804 | 51/26412 | 0.323232362 | 0.413737423 | Metabolism | Metabolism of cofactors and vitamins |
| 1 | map00515 | Mannose type O-glycan biosynthesis | KEGG PATHWAY | 1/8804 | 1/26412 | 0.333333333 | 0.422442244 | Metabolism | Glycan biosynthesis and metabolism |
| 17 | map00670 | One carbon pool by folate | KEGG PATHWAY | 17/8804 | 46/26412 | 0.352061171 | 0.441802254 | Metabolism | Metabolism of cofactors and vitamins |
| 20 | map03022 | Basal transcription factors | KEGG PATHWAY | 20/8804 | 55/26412 | 0.364011272 | 0.448013873 | Genetic Information Processing | Transcription |
| 5 | map03450 | Non-homologous end-joining | KEGG PATHWAY | 5/8804 | 12/26412 | 0.368461222 | 0.449171776 | Genetic Information Processing | Replication and repair |
| 107 | map03015 | mRNA surveillance pathway | KEGG PATHWAY | 107/8804 | 311/26412 | 0.363711106 | 0.451990501 | Genetic Information Processing | Translation |
| 6 | map00440 | Phosphonate and phosphinate metabolism | KEGG PATHWAY | 6/8804 | 15/26412 | 0.381607866 | 0.460809499 | Metabolism | Metabolism of other amino acids |
| 7 | map00430 | Taurine and hypotaurine metabolism | KEGG PATHWAY | 7/8804 | 18/26412 | 0.391467347 | 0.468297387 | Metabolism | Metabolism of other amino acids |
| 34 | map03430 | Mismatch repair | KEGG PATHWAY | 34/8804 | 97/26412 | 0.396382565 | 0.469786744 | Genetic Information Processing | Replication and repair |
| 15 | map00340 | Histidine metabolism | KEGG PATHWAY | 15/8804 | 42/26412 | 0.428071803 | 0.502689824 | Metabolism | Amino acid metabolism |
| 91 | map03030 | DNA replication | KEGG PATHWAY | 91/8804 | 269/26412 | 0.454102355 | 0.528410013 | Genetic Information Processing | Replication and repair |
| 50 | map00970 | Aminoacyl-tRNA biosynthesis | KEGG PATHWAY | 50/8804 | 147/26412 | 0.461257285 | 0.531900292 | Genetic Information Processing | Translation |
| 77 | map03008 | Ribosome biogenesis in eukaryotes | KEGG PATHWAY | 77/8804 | 228/26412 | 0.468833764 | 0.535810016 | Genetic Information Processing | Translation |
| 1 | map00943 | Isoflavonoid biosynthesis | KEGG PATHWAY | 1/8804 | 2/26412 | 0.55556397 | 0.629311399 | Metabolism | Biosynthesis of other secondary metabolites |
| 1 | map00942 | Anthocyanin biosynthesis | KEGG PATHWAY | 1/8804 | 2/26412 | 0.55556397 | 0.629311399 | Metabolism | Biosynthesis of other secondary metabolites |
| 20 | map00730 | Thiamine metabolism | KEGG PATHWAY | 20/8804 | 61/26412 | 0.583891653 | 0.649896796 | Metabolism | Metabolism of cofactors and vitamins |
| 37 | map03420 | Nucleotide excision repair | KEGG PATHWAY | 37/8804 | 115/26412 | 0.638204367 | 0.704225508 | Genetic Information Processing | Replication and repair |
| 11 | map00590 | Arachidonic acid metabolism | KEGG PATHWAY | 11/8804 | 35/26412 | 0.655761682 | 0.71741449 | Metabolism | Lipid metabolism |
| 15 | map00790 | Folate biosynthesis | KEGG PATHWAY | 15/8804 | 48/26412 | 0.671983298 | 0.728931035 | Metabolism | Metabolism of cofactors and vitamins |
| 36 | map00640 | Propanoate metabolism | KEGG PATHWAY | 36/8804 | 115/26412 | 0.710167256 | 0.763877385 | Metabolism | Carbohydrate metabolism |
| 7 | map00563 | Glycosylphosphatidylinositol (GPI)-anchor biosynthesis | KEGG PATHWAY | 7/8804 | 25/26412 | 0.778579054 | 0.823620818 | Metabolism | Glycan biosynthesis and metabolism |
| 41 | map03050 | Proteasome | KEGG PATHWAY | 41/8804 | 134/26412 | 0.776706511 | 0.828486945 | Genetic Information Processing | Folding, sorting and degradation |
| 5 | map00072 | Synthesis and degradation of ketone bodies | KEGG PATHWAY | 5/8804 | 19/26412 | 0.812156506 | 0.852098629 | Metabolism | Lipid metabolism |
| 3 | map00514 | Other types of O-glycan biosynthesis | KEGG PATHWAY | 3/8804 | 12/26412 | 0.818937552 | 0.852227697 | Metabolism | Glycan biosynthesis and metabolism |
| 2 | map01502 | Vancomycin resistance | KEGG PATHWAY | 2/8804 | 9/26412 | 0.856976702 | 0.884621112 | Human Diseases | Drug resistance: antimicrobial |
| 17 | map00650 | Butanoate metabolism | KEGG PATHWAY | 17/8804 | 64/26412 | 0.902297757 | 0.923952903 | Metabolism | Carbohydrate metabolism |
| 108 | map02010 | ABC transporters | KEGG PATHWAY | 108/8804 | 687/26412 | 1 | 1 | Environmental Information Processing | Membrane transport |
| 572 | map00230 | Purine metabolism | KEGG PATHWAY | 572/8804 | 4913/26412 | 1 | 1 | Metabolism | Nucleotide metabolism |
| 39 | map00785 | Lipoic acid metabolism | KEGG PATHWAY | 39/8804 | 282/26412 | 1 | 1 | Metabolism | Metabolism of cofactors and vitamins |

**Supplementary Table 4.** Details of transcription factor analysis

| **Gene_id** | **PF ID** | **DNA domain** | **Description** | **Family** | **E-value** | **Score** |
| --- | --- | --- | --- | --- | --- | --- |
| TRINITY_DN53991_c3_g2 | PF00320 | GATA-zf | GATA zinc finger | C2C2 | 6.7E-13 | 47.9 |
| TRINITY_DN73139_c0_g4 | PF00320 | GATA-zf | GATA zinc finger | C2C2 | 1.3E-15 | 56.5 |
| TRINITY_DN73111_c0_g1 | PF00320 | GATA-zf | GATA zinc finger | C2C2 | 4.6E-14 | 51.6 |
| TRINITY_DN63282_c4_g1 | PF00320 | GATA-zf | GATA zinc finger | C2C2 | 5.1E-17 | 61.1 |
| TRINITY_DN63282_c4_g2 | PF00320 | GATA-zf | GATA zinc finger | C2C2 | 4.8E-16 | 57.9 |
| TRINITY_DN10903_c0_g1 | PF00320 | GATA-zf | GATA zinc finger | C2C2 | 0.000000022 | 33.4 |
| TRINITY_DN67755_c0_g1 | PF00320 | GATA-zf | GATA zinc finger | C2C2 | 1.4E-15 | 56.4 |
| TRINITY_DN63368_c3_g1 | PF00320 | GATA-zf | GATA zinc finger | C2C2 | 7.2E-17 | 60.6 |
| TRINITY_DN50434_c0_g3 | PF00320 | GATA-zf | GATA zinc finger | C2C2 | 4.4E-17 | 61.3 |
| TRINITY_DN43167_c0_g1 | PF00320 | GATA-zf | GATA zinc finger | C2C2 | 1.3E-18 | 66.1 |
| TRINITY_DN48855_c2_g3 | PF00320 | GATA-zf | GATA zinc finger | C2C2 | 2.6E-13 | 49.2 |
| TRINITY_DN48855_c2_g1 | PF00320 | GATA-zf | GATA zinc finger | C2C2 | 6E-13 | 48 |
| TRINITY_DN48865_c5_g1 | PF00320 | GATA-zf | GATA zinc finger | C2C2 | 1.2E-10 | 40.7 |
| TRINITY_DN71216_c4_g3 | PF00320 | GATA-zf | GATA zinc finger | C2C2 | 0.000000011 | 34.4 |
| TRINITY_DN71216_c4_g3 | PF03101 | FAR1 | FAR1 DNA-binding domain | FAR1 | 5E-22 | 78.3 |
| TRINITY_DN65286_c4_g2 | PF00320 | GATA-zf | GATA zinc finger | C2C2 | 5.4E-12 | 45 |
| TRINITY_DN65286_c4_g2 | PF03101 | FAR1 | FAR1 DNA-binding domain | FAR1 | 3.3E-25 | 88.5 |
| TRINITY_DN63875_c8_g1 | PF00320 | GATA-zf | GATA zinc finger | C2C2 | 2E-17 | 62.3 |
| TRINITY_DN53121_c5_g1 | PF00320 | GATA-zf | GATA zinc finger | C2C2 | 4.6E-11 | 42 |
| TRINITY_DN92172_c0_g1 | PF02365 | NAM | No apical meristem (NAM) protein | NAC | 0.0000016 | 28.7 |
| TRINITY_DN73799_c0_g1 | PF02365 | NAM | No apical meristem (NAM) protein | NAC | 0.0001 | 22.9 |
| TRINITY_DN47923_c0_g1 | PF02365 | NAM | No apical meristem (NAM) protein | NAC | 1.7E-15 | 57.5 |
| TRINITY_DN47923_c0_g1 | PF02365 | NAM | No apical meristem (NAM) protein | NAC | 0.00000078 | 29.7 |
| TRINITY_DN47923_c0_g3 | PF02365 | NAM | No apical meristem (NAM) protein | NAC | 1.7E-15 | 57.5 |
| TRINITY_DN47923_c0_g3 | PF02365 | NAM | No apical meristem (NAM) protein | NAC | 0.00000078 | 29.7 |
| TRINITY_DN44727_c0_g1 | PF02365 | NAM | No apical meristem (NAM) protein | NAC | 5.7E-22 | 78.2 |
| TRINITY_DN44727_c0_g2 | PF02365 | NAM | No apical meristem (NAM) protein | NAC | 8.4E-23 | 80.9 |
| TRINITY_DN44727_c0_g3 | PF02365 | NAM | No apical meristem (NAM) protein | NAC | 2E-20 | 73.3 |
| TRINITY_DN54160_c5_g3 | PF02365 | NAM | No apical meristem (NAM) protein | NAC | 2.8E-12 | 47.2 |
| TRINITY_DN70245_c0_g1 | PF02365 | NAM | No apical meristem (NAM) protein | NAC | 4.2E-27 | 94.7 |
| TRINITY_DN70245_c0_g2 | PF02365 | NAM | No apical meristem (NAM) protein | NAC | 1.1E-27 | 96.6 |
| TRINITY_DN70245_c0_g4 | PF02365 | NAM | No apical meristem (NAM) protein | NAC | 4.4E-23 | 81.8 |
| TRINITY_DN59983_c5_g3 | PF02365 | NAM | No apical meristem (NAM) protein | NAC | 2.4E-12 | 47.4 |
| TRINITY_DN63377_c0_g3 | PF02365 | NAM | No apical meristem (NAM) protein | NAC | 5E-13 | 49.6 |
| TRINITY_DN63377_c0_g3 | PF03101 | FAR1 | FAR1 DNA-binding domain | FAR1 | 0.00033 | 21.2 |
| TRINITY_DN51242_c1_g1 | PF02365 | NAM | No apical meristem (NAM) protein | NAC | 8.2E-28 | 97 |
| TRINITY_DN48294_c3_g1 | PF02365 | NAM | No apical meristem (NAM) protein | NAC | 4.9E-29 | 100.9 |
| TRINITY_DN43905_c0_g1 | PF02365 | NAM | No apical meristem (NAM) protein | NAC | 1.2E-27 | 96.5 |
| TRINITY_DN44570_c0_g1 | PF02365 | NAM | No apical meristem (NAM) protein | NAC | 0.0000089 | 26.3 |
| TRINITY_DN44570_c0_g1 | PF02365 | NAM | No apical meristem (NAM) protein | NAC | 0.000000096 | 32.6 |
| TRINITY_DN50025_c0_g1 | PF02365 | NAM | No apical meristem (NAM) protein | NAC | 1.1E-15 | 58 |
| TRINITY_DN50025_c0_g2 | PF02365 | NAM | No apical meristem (NAM) protein | NAC | 4E-28 | 97.9 |
| TRINITY_DN50025_c1_g1 | PF02365 | NAM | No apical meristem (NAM) protein | NAC | 1.1E-29 | 103 |
| TRINITY_DN70461_c2_g4 | PF02365 | NAM | No apical meristem (NAM) protein | NAC | 2E-17 | 63.7 |
| TRINITY_DN42270_c0_g2 | PF02365 | NAM | No apical meristem (NAM) protein | NAC | 7.5E-22 | 77.9 |
| TRINITY_DN42270_c1_g2 | PF02365 | NAM | No apical meristem (NAM) protein | NAC | 0.000000002 | 38 |
| TRINITY_DN47087_c3_g8 | PF02365 | NAM | No apical meristem (NAM) protein | NAC | 0.00000005 | 33.6 |
| TRINITY_DN47087_c3_g2 | PF02365 | NAM | No apical meristem (NAM) protein | NAC | 1.2E-25 | 90 |
| TRINITY_DN68856_c1_g2 | PF02365 | NAM | No apical meristem (NAM) protein | NAC | 1.3E-11 | 45.1 |
| TRINITY_DN68856_c1_g3 | PF02365 | NAM | No apical meristem (NAM) protein | NAC | 1.5E-29 | 102.6 |
| TRINITY_DN68856_c1_g1 | PF02365 | NAM | No apical meristem (NAM) protein | NAC | 0.00000006 | 33.3 |
| TRINITY_DN68856_c1_g1 | PF02365 | NAM | No apical meristem (NAM) protein | NAC | 0.000023 | 25 |
| TRINITY_DN65251_c2_g1 | PF02365 | NAM | No apical meristem (NAM) protein | NAC | 1.9E-22 | 79.8 |
| TRINITY_DN65251_c3_g1 | PF02365 | NAM | No apical meristem (NAM) protein | NAC | 9.6E-20 | 71.1 |
| TRINITY_DN65251_c3_g2 | PF02365 | NAM | No apical meristem (NAM) protein | NAC | 2.9E-29 | 101.6 |
| TRINITY_DN50577_c1_g1 | PF02365 | NAM | No apical meristem (NAM) protein | NAC | 5.4E-28 | 97.5 |
| TRINITY_DN50577_c1_g2 | PF02365 | NAM | No apical meristem (NAM) protein | NAC | 5.2E-27 | 94.4 |
| TRINITY_DN50577_c1_g3 | PF02365 | NAM | No apical meristem (NAM) protein | NAC | 2.1E-29 | 102.1 |
| TRINITY_DN51786_c1_g2 | PF02365 | NAM | No apical meristem (NAM) protein | NAC | 7.7E-23 | 81 |
| TRINITY_DN51786_c2_g1 | PF02365 | NAM | No apical meristem (NAM) protein | NAC | 1E-29 | 103.1 |
| TRINITY_DN51786_c3_g4 | PF02365 | NAM | No apical meristem (NAM) protein | NAC | 9.3E-27 | 93.6 |
| TRINITY_DN51786_c3_g1 | PF02365 | NAM | No apical meristem (NAM) protein | NAC | 6.4E-27 | 94.1 |
| TRINITY_DN51786_c3_g2 | PF02365 | NAM | No apical meristem (NAM) protein | NAC | 2.5E-09 | 37.7 |
| TRINITY_DN51786_c3_g2 | PF02365 | NAM | No apical meristem (NAM) protein | NAC | 0.0000014 | 28.9 |
| TRINITY_DN70339_c1_g1 | PF02365 | NAM | No apical meristem (NAM) protein | NAC | 6.9E-30 | 103.6 |
| TRINITY_DN49461_c0_g1 | PF02365 | NAM | No apical meristem (NAM) protein | NAC | 3.8E-29 | 101.2 |
| TRINITY_DN49461_c0_g2 | PF02365 | NAM | No apical meristem (NAM) protein | NAC | 0.000014 | 25.7 |
| TRINITY_DN49461_c0_g2 | PF02365 | NAM | No apical meristem (NAM) protein | NAC | 0.00065 | 20.4 |
| TRINITY_DN49461_c0_g3 | PF02365 | NAM | No apical meristem (NAM) protein | NAC | 9.2E-28 | 96.8 |
| TRINITY_DN49461_c0_g4 | PF02365 | NAM | No apical meristem (NAM) protein | NAC | 1.7E-17 | 63.9 |
| TRINITY_DN49461_c0_g6 | PF02365 | NAM | No apical meristem (NAM) protein | NAC | 0.00000092 | 29.5 |
| TRINITY_DN89508_c0_g1 | PF02365 | NAM | No apical meristem (NAM) protein | NAC | 5.9E-15 | 55.8 |
| TRINITY_DN44307_c0_g1 | PF02365 | NAM | No apical meristem (NAM) protein | NAC | 1.4E-24 | 86.6 |
| TRINITY_DN12127_c0_g1 | PF02365 | NAM | No apical meristem (NAM) protein | NAC | 1.2E-33 | 115.7 |
| TRINITY_DN52249_c4_g2 | PF02365 | NAM | No apical meristem (NAM) protein | NAC | 4E-11 | 43.5 |
| TRINITY_DN42936_c0_g1 | PF02365 | NAM | No apical meristem (NAM) protein | NAC | 1.2E-25 | 90 |
| TRINITY_DN42936_c0_g2 | PF02365 | NAM | No apical meristem (NAM) protein | NAC | 9.6E-26 | 90.3 |
| TRINITY_DN49786_c3_g1 | PF02365 | NAM | No apical meristem (NAM) protein | NAC | 5.8E-26 | 91 |
| TRINITY_DN49786_c3_g8 | PF02365 | NAM | No apical meristem (NAM) protein | NAC | 3.5E-21 | 75.7 |
| TRINITY_DN44118_c1_g1 | PF02365 | NAM | No apical meristem (NAM) protein | NAC | 2.5E-16 | 60.2 |
| TRINITY_DN50949_c0_g1 | PF02365 | NAM | No apical meristem (NAM) protein | NAC | 1.8E-25 | 89.4 |
| TRINITY_DN55467_c3_g1 | PF02365 | NAM | No apical meristem (NAM) protein | NAC | 5.4E-30 | 104 |
| TRINITY_DN73969_c3_g1 | PF02365 | NAM | No apical meristem (NAM) protein | NAC | 1.9E-23 | 83 |
| TRINITY_DN34623_c0_g2 | PF02365 | NAM | No apical meristem (NAM) protein | NAC | 2E-28 | 99 |
| TRINITY_DN46690_c0_g1 | PF02365 | NAM | No apical meristem (NAM) protein | NAC | 1.6E-28 | 99.3 |
| TRINITY_DN49389_c1_g1 | PF02365 | NAM | No apical meristem (NAM) protein | NAC | 3.6E-16 | 59.6 |
| TRINITY_DN49389_c1_g2 | PF02365 | NAM | No apical meristem (NAM) protein | NAC | 1.2E-20 | 74 |
| TRINITY_DN76242_c0_g1 | PF02365 | NAM | No apical meristem (NAM) protein | NAC | 0.00000002 | 34.8 |
| TRINITY_DN44436_c0_g1 | PF02365 | NAM | No apical meristem (NAM) protein | NAC | 0.000000015 | 35.3 |
| TRINITY_DN49135_c0_g1 | PF02365 | NAM | No apical meristem (NAM) protein | NAC | 0.000003 | 27.8 |
| TRINITY_DN65554_c1_g5 | PF02365 | NAM | No apical meristem (NAM) protein | NAC | 5.3E-29 | 100.8 |
| TRINITY_DN65554_c1_g2 | PF02365 | NAM | No apical meristem (NAM) protein | NAC | 6.8E-26 | 90.8 |
| TRINITY_DN65554_c1_g6 | PF02365 | NAM | No apical meristem (NAM) protein | NAC | 3.3E-30 | 104.6 |
| TRINITY_DN65554_c1_g8 | PF02365 | NAM | No apical meristem (NAM) protein | NAC | 7.9E-25 | 87.4 |
| TRINITY_DN65554_c1_g10 | PF02365 | NAM | No apical meristem (NAM) protein | NAC | 4E-13 | 49.9 |
| TRINITY_DN60721_c2_g1 | PF00319 | SRF-TF | SRF-type transcription factor (DNA-binding and dimerisation domain) | MADS | 6.1E-21 | 73.5 |
| TRINITY_DN54716_c0_g2 | PF00319 | SRF-TF | SRF-type transcription factor (DNA-binding and dimerisation domain) | MADS | 2.8E-25 | 87.4 |
| TRINITY_DN63855_c1_g1 | PF00319 | SRF-TF | SRF-type transcription factor (DNA-binding and dimerisation domain) | MADS | 4.6E-26 | 89.9 |
| TRINITY_DN65149_c0_g2 | PF00319 | SRF-TF | SRF-type transcription factor (DNA-binding and dimerisation domain) | MADS | 1.2E-25 | 88.6 |
| TRINITY_DN71037_c4_g1 | PF00319 | SRF-TF | SRF-type transcription factor (DNA-binding and dimerisation domain) | MADS | 2.5E-26 | 90.7 |
| TRINITY_DN47958_c1_g3 | PF00319 | SRF-TF | SRF-type transcription factor (DNA-binding and dimerisation domain) | MADS | 2.2E-25 | 87.7 |
| TRINITY_DN71942_c0_g2 | PF00319 | SRF-TF | SRF-type transcription factor (DNA-binding and dimerisation domain) | MADS | 2.8E-25 | 87.4 |
| TRINITY_DN71942_c0_g1 | PF00319 | SRF-TF | SRF-type transcription factor (DNA-binding and dimerisation domain) | MADS | 3.7E-27 | 93.4 |
| TRINITY_DN41652_c0_g1 | PF00319 | SRF-TF | SRF-type transcription factor (DNA-binding and dimerisation domain) | MADS | 1.2E-26 | 91.8 |
| TRINITY_DN72068_c4_g2 | PF00319 | SRF-TF | SRF-type transcription factor (DNA-binding and dimerisation domain) | MADS | 2.4E-25 | 87.6 |
| TRINITY_DN64503_c1_g1 | PF00319 | SRF-TF | SRF-type transcription factor (DNA-binding and dimerisation domain) | MADS | 7.5E-28 | 95.6 |
| TRINITY_DN64503_c2_g1 | PF00319 | SRF-TF | SRF-type transcription factor (DNA-binding and dimerisation domain) | MADS | 1.2E-26 | 91.7 |
| TRINITY_DN82280_c0_g1 | PF00319 | SRF-TF | SRF-type transcription factor (DNA-binding and dimerisation domain) | MADS | 1.2E-11 | 43.8 |
| TRINITY_DN73752_c1_g1 | PF00319 | SRF-TF | SRF-type transcription factor (DNA-binding and dimerisation domain) | MADS | 6.9E-21 | 73.3 |
| TRINITY_DN54856_c1_g1 | PF00319 | SRF-TF | SRF-type transcription factor (DNA-binding and dimerisation domain) | MADS | 1.1E-09 | 37.5 |
| TRINITY_DN54856_c1_g13 | PF00319 | SRF-TF | SRF-type transcription factor (DNA-binding and dimerisation domain) | MADS | 7.3E-10 | 38 |
| TRINITY_DN56139_c1_g3 | PF00319 | SRF-TF | SRF-type transcription factor (DNA-binding and dimerisation domain) | MADS | 2.9E-25 | 87.3 |
| TRINITY_DN56139_c2_g2 | PF00319 | SRF-TF | SRF-type transcription factor (DNA-binding and dimerisation domain) | MADS | 1.5E-25 | 88.3 |
| TRINITY_DN55522_c1_g2 | PF00319 | SRF-TF | SRF-type transcription factor (DNA-binding and dimerisation domain) | MADS | 1.1E-26 | 91.8 |
| TRINITY_DN74160_c1_g3 | PF00319 | SRF-TF | SRF-type transcription factor (DNA-binding and dimerisation domain) | MADS | 2.6E-10 | 39.5 |
| TRINITY_DN70817_c0_g5 | PF00319 | SRF-TF | SRF-type transcription factor (DNA-binding and dimerisation domain) | MADS | 1.3E-14 | 53.2 |
| TRINITY_DN70817_c0_g2 | PF00319 | SRF-TF | SRF-type transcription factor (DNA-binding and dimerisation domain) | MADS | 0.00000027 | 29.8 |
| TRINITY_DN68075_c6_g1 | PF00319 | SRF-TF | SRF-type transcription factor (DNA-binding and dimerisation domain) | MADS | 1.3E-23 | 82.1 |
| TRINITY_DN49299_c1_g4 | PF00319 | SRF-TF | SRF-type transcription factor (DNA-binding and dimerisation domain) | MADS | 4.3E-25 | 86.8 |
| TRINITY_DN91440_c0_g1 | PF00319 | SRF-TF | SRF-type transcription factor (DNA-binding and dimerisation domain) | MADS | 9.3E-11 | 40.9 |
| TRINITY_DN51486_c1_g2 | PF00319 | SRF-TF | SRF-type transcription factor (DNA-binding and dimerisation domain) | MADS | 4.3E-23 | 80.4 |
| TRINITY_DN70097_c3_g1 | PF00319 | SRF-TF | SRF-type transcription factor (DNA-binding and dimerisation domain) | MADS | 5.9E-09 | 35.1 |
| TRINITY_DN59335_c0_g3 | PF00319 | SRF-TF | SRF-type transcription factor (DNA-binding and dimerisation domain) | MADS | 1.3E-12 | 46.8 |
| TRINITY_DN74712_c1_g4 | PF00319 | SRF-TF | SRF-type transcription factor (DNA-binding and dimerisation domain) | MADS | 0.000000022 | 33.3 |
| TRINITY_DN60638_c1_g2 | PF00319 | SRF-TF | SRF-type transcription factor (DNA-binding and dimerisation domain) | MADS | 2.3E-26 | 90.9 |
| TRINITY_DN60638_c1_g5 | PF00319 | SRF-TF | SRF-type transcription factor (DNA-binding and dimerisation domain) | MADS | 1.2E-26 | 91.7 |
| TRINITY_DN60638_c2_g1 | PF00319 | SRF-TF | SRF-type transcription factor (DNA-binding and dimerisation domain) | MADS | 1.2E-26 | 91.8 |
| TRINITY_DN51869_c1_g1 | PF00319 | SRF-TF | SRF-type transcription factor (DNA-binding and dimerisation domain) | MADS | 8.8E-23 | 79.4 |
| TRINITY_DN60728_c0_g1 | PF03514 | GRAS | GRAS domain family | GRAS | 0.000015 | 24 |
| TRINITY_DN76489_c1_g1 | PF03514 | GRAS | GRAS domain family | GRAS | 2.7E-14 | 52.8 |
| TRINITY_DN76489_c1_g2 | PF03514 | GRAS | GRAS domain family | GRAS | 1.8E-22 | 79.7 |
| TRINITY_DN76489_c1_g2 | PF03514 | GRAS | GRAS domain family | GRAS | 6.6E-23 | 81.1 |
| TRINITY_DN74973_c3_g1 | PF03514 | GRAS | GRAS domain family | GRAS | 1.1E-121 | 406.2 |
| TRINITY_DN50952_c1_g11 | PF03514 | GRAS | GRAS domain family | GRAS | 6.4E-99 | 331.3 |
| TRINITY_DN41952_c0_g1 | PF03514 | GRAS | GRAS domain family | GRAS | 4.4E-11 | 42.2 |
| TRINITY_DN41952_c0_g2 | PF03514 | GRAS | GRAS domain family | GRAS | 1.6E-82 | 277.3 |
| TRINITY_DN53959_c0_g2 | PF03514 | GRAS | GRAS domain family | GRAS | 9.3E-136 | 452.5 |
| TRINITY_DN66761_c1_g6 | PF03514 | GRAS | GRAS domain family | GRAS | 2.1E-17 | 63 |
| TRINITY_DN68500_c0_g1 | PF03514 | GRAS | GRAS domain family | GRAS | 4E-51 | 174 |
| TRINITY_DN68500_c0_g2 | PF03514 | GRAS | GRAS domain family | GRAS | 0.000000022 | 33.3 |
| TRINITY_DN79624_c0_g1 | PF03514 | GRAS | GRAS domain family | GRAS | 4.7E-44 | 150.7 |
| TRINITY_DN55358_c1_g11 | PF03514 | GRAS | GRAS domain family | GRAS | 8.9E-98 | 327.5 |
| TRINITY_DN42110_c0_g1 | PF03514 | GRAS | GRAS domain family | GRAS | 1.4E-71 | 241.3 |
| TRINITY_DN60043_c5_g2 | PF03514 | GRAS | GRAS domain family | GRAS | 2.2E-136 | 454.6 |
| TRINITY_DN67978_c1_g3 | PF03514 | GRAS | GRAS domain family | GRAS | 7.5E-103 | 344.2 |
| TRINITY_DN64795_c0_g1 | PF03514 | GRAS | GRAS domain family | GRAS | 3E-88 | 296.2 |
| TRINITY_DN64795_c0_g6 | PF03514 | GRAS | GRAS domain family | GRAS | 9.8E-89 | 297.8 |
| TRINITY_DN64795_c0_g3 | PF03514 | GRAS | GRAS domain family | GRAS | 1E-95 | 320.7 |
| TRINITY_DN66474_c0_g2 | PF03514 | GRAS | GRAS domain family | GRAS | 0.000083 | 21.5 |
| TRINITY_DN67273_c1_g1 | PF03514 | GRAS | GRAS domain family | GRAS | 2.2E-130 | 434.8 |
| TRINITY_DN67273_c1_g2 | PF03514 | GRAS | GRAS domain family | GRAS | 0.000011 | 24.5 |
| TRINITY_DN40143_c0_g1 | PF03514 | GRAS | GRAS domain family | GRAS | 1.8E-115 | 385.7 |
| TRINITY_DN42587_c0_g1 | PF03514 | GRAS | GRAS domain family | GRAS | 1E-88 | 297.7 |
| TRINITY_DN70069_c1_g1 | PF03514 | GRAS | GRAS domain family | GRAS | 1.7E-56 | 191.7 |
| TRINITY_DN70069_c1_g3 | PF03514 | GRAS | GRAS domain family | GRAS | 1.1E-31 | 110 |
| TRINITY_DN74368_c0_g1 | PF03514 | GRAS | GRAS domain family | GRAS | 7.9E-23 | 80.9 |
| TRINITY_DN74368_c0_g1 | PF03514 | GRAS | GRAS domain family | GRAS | 0.000000085 | 31.4 |
| TRINITY_DN74368_c0_g1 | PF03514 | GRAS | GRAS domain family | GRAS | 5.6E-13 | 48.4 |
| TRINITY_DN74368_c0_g1 | PF03514 | GRAS | GRAS domain family | GRAS | 0.0000057 | 25.4 |
| TRINITY_DN57732_c3_g1 | PF03514 | GRAS | GRAS domain family | GRAS | 3.1E-67 | 227 |
| TRINITY_DN46347_c0_g4 | PF03514 | GRAS | GRAS domain family | GRAS | 2.5E-96 | 322.8 |
| TRINITY_DN49028_c1_g5 | PF03514 | GRAS | GRAS domain family | GRAS | 9.1E-23 | 80.7 |
| TRINITY_DN49028_c1_g6 | PF03514 | GRAS | GRAS domain family | GRAS | 0.0000044 | 25.8 |
| TRINITY_DN49028_c1_g4 | PF03514 | GRAS | GRAS domain family | GRAS | 3.9E-41 | 141.1 |
| TRINITY_DN49028_c1_g8 | PF03514 | GRAS | GRAS domain family | GRAS | 2.3E-15 | 56.3 |
| TRINITY_DN49028_c1_g8 | PF03514 | GRAS | GRAS domain family | GRAS | 3E-12 | 46 |
| TRINITY_DN42322_c0_g1 | PF03514 | GRAS | GRAS domain family | GRAS | 0.000000011 | 34.3 |
| TRINITY_DN23141_c0_g1 | PF03514 | GRAS | GRAS domain family | GRAS | 3.8E-135 | 450.5 |
| TRINITY_DN40858_c0_g1 | PF03514 | GRAS | GRAS domain family | GRAS | 3.5E-48 | 164.3 |
| TRINITY_DN47489_c0_g1 | PF03514 | GRAS | GRAS domain family | GRAS | 5.8E-116 | 387.4 |
| TRINITY_DN62017_c1_g2 | PF03514 | GRAS | GRAS domain family | GRAS | 1E-10 | 41 |
| TRINITY_DN62017_c1_g2 | PF03514 | GRAS | GRAS domain family | GRAS | 3.9E-10 | 39.1 |
| TRINITY_DN59818_c2_g1 | PF03514 | GRAS | GRAS domain family | GRAS | 3E-120 | 401.4 |
| TRINITY_DN91432_c0_g1 | PF03514 | GRAS | GRAS domain family | GRAS | 2.4E-108 | 362.3 |
| TRINITY_DN44890_c0_g1 | PF03514 | GRAS | GRAS domain family | GRAS | 7E-117 | 390.4 |
| TRINITY_DN44448_c0_g1 | PF03514 | GRAS | GRAS domain family | GRAS | 5.1E-38 | 130.9 |
| TRINITY_DN45020_c0_g1 | PF03514 | GRAS | GRAS domain family | GRAS | 7.3E-45 | 153.4 |
| TRINITY_DN45020_c0_g1 | PF03514 | GRAS | GRAS domain family | GRAS | 6.9E-21 | 74.5 |
| TRINITY_DN68019_c0_g1 | PF03514 | GRAS | GRAS domain family | GRAS | 7E-15 | 54.7 |
| TRINITY_DN92593_c0_g1 | PF03514 | GRAS | GRAS domain family | GRAS | 0.00000018 | 30.3 |
| TRINITY_DN21428_c0_g1 | PF03514 | GRAS | GRAS domain family | GRAS | 2.8E-18 | 65.9 |
| TRINITY_DN69563_c0_g2 | PF03514 | GRAS | GRAS domain family | GRAS | 2E-61 | 207.9 |
| TRINITY_DN47613_c0_g1 | PF03514 | GRAS | GRAS domain family | GRAS | 0.000000055 | 32 |
| TRINITY_DN74193_c2_g3 | PF03514 | GRAS | GRAS domain family | GRAS | 4E-116 | 387.9 |
| TRINITY_DN74193_c2_g4 | PF03514 | GRAS | GRAS domain family | GRAS | 3.5E-54 | 184.1 |
| TRINITY_DN74193_c2_g1 | PF03514 | GRAS | GRAS domain family | GRAS | 2.4E-109 | 365.6 |
| TRINITY_DN64180_c4_g2 | PF03514 | GRAS | GRAS domain family | GRAS | 0.000082 | 21.6 |
| TRINITY_DN76526_c5_g3 | PF03514 | GRAS | GRAS domain family | GRAS | 0.0000015 | 27.3 |
| TRINITY_DN71588_c1_g7 | PF03514 | GRAS | GRAS domain family | GRAS | 2.5E-54 | 184.5 |
| TRINITY_DN29268_c0_g1 | PF03514 | GRAS | GRAS domain family | GRAS | 9.8E-58 | 195.7 |
| TRINITY_DN29268_c0_g2 | PF03514 | GRAS | GRAS domain family | GRAS | 1.6E-61 | 208.2 |
| TRINITY_DN60754_c0_g1 | PF00170 | bZIP_1 | bZIP transcription factor | bZIP | 0.000000028 | 33.6 |
| TRINITY_DN59349_c0_g4 | PF00170 | bZIP_1 | bZIP transcription factor | bZIP | 0.000089 | 22.4 |
| TRINITY_DN59349_c0_g4 | PF08879 | WRC | WRC | GRF | 0.000000009 | 34.8 |
| TRINITY_DN59349_c1_g1 | PF00170 | bZIP_1 | bZIP transcription factor | bZIP | 0.00022 | 21.1 |
| TRINITY_DN46171_c2_g6 | PF00170 | bZIP_1 | bZIP transcription factor | bZIP | 4.8E-13 | 48.9 |
| TRINITY_DN59211_c3_g2 | PF00170 | bZIP_1 | bZIP transcription factor | bZIP | 1.4E-09 | 37.7 |
| TRINITY_DN48599_c0_g2 | PF00170 | bZIP_1 | bZIP transcription factor | bZIP | 0.00021 | 21.2 |
| TRINITY_DN66414_c0_g5 | PF00170 | bZIP_1 | bZIP transcription factor | bZIP | 0.0000056 | 26.2 |
| TRINITY_DN38241_c0_g1 | PF00170 | bZIP_1 | bZIP transcription factor | bZIP | 9.3E-11 | 41.5 |
| TRINITY_DN56066_c0_g1 | PF00170 | bZIP_1 | bZIP transcription factor | bZIP | 8E-10 | 38.5 |
| TRINITY_DN69670_c0_g1 | PF00170 | bZIP_1 | bZIP transcription factor | bZIP | 0.00000023 | 30.7 |
| TRINITY_DN59923_c0_g3 | PF00170 | bZIP_1 | bZIP transcription factor | bZIP | 6.1E-11 | 42.1 |
| TRINITY_DN49188_c7_g2 | PF00170 | bZIP_1 | bZIP transcription factor | bZIP | 0.000000018 | 34.2 |
| TRINITY_DN48344_c2_g1 | PF00170 | bZIP_1 | bZIP transcription factor | bZIP | 0.00000019 | 31 |
| TRINITY_DN66670_c0_g1 | PF00170 | bZIP_1 | bZIP transcription factor | bZIP | 2.7E-13 | 49.7 |
| TRINITY_DN66606_c1_g1 | PF00170 | bZIP_1 | bZIP transcription factor | bZIP | 4.1E-19 | 68.3 |
| TRINITY_DN51727_c1_g2 | PF00170 | bZIP_1 | bZIP transcription factor | bZIP | 9.7E-13 | 47.9 |
| TRINITY_DN67846_c3_g1 | PF00170 | bZIP_1 | bZIP transcription factor | bZIP | 7.4E-09 | 35.4 |
| TRINITY_DN63189_c0_g1 | PF00170 | bZIP_1 | bZIP transcription factor | bZIP | 0.000000005 | 36 |
| TRINITY_DN72651_c2_g1 | PF00170 | bZIP_1 | bZIP transcription factor | bZIP | 0.0000012 | 28.3 |
| TRINITY_DN72407_c0_g1 | PF00170 | bZIP_1 | bZIP transcription factor | bZIP | 1.5E-10 | 40.9 |
| TRINITY_DN68800_c1_g1 | PF00170 | bZIP_1 | bZIP transcription factor | bZIP | 1.1E-13 | 50.9 |
| TRINITY_DN73280_c3_g1 | PF00170 | bZIP_1 | bZIP transcription factor | bZIP | 3.6E-12 | 46.1 |
| TRINITY_DN68560_c0_g1 | PF00170 | bZIP_1 | bZIP transcription factor | bZIP | 9.6E-14 | 51.1 |
| TRINITY_DN12178_c0_g1 | PF00170 | bZIP_1 | bZIP transcription factor | bZIP | 5.9E-16 | 58.2 |
| TRINITY_DN74143_c1_g4 | PF00170 | bZIP_1 | bZIP transcription factor | bZIP | 3.2E-09 | 36.6 |
| TRINITY_DN68432_c4_g1 | PF00170 | bZIP_1 | bZIP transcription factor | bZIP | 6.8E-17 | 61.2 |
| TRINITY_DN43565_c0_g1 | PF00170 | bZIP_1 | bZIP transcription factor | bZIP | 0.00058 | 19.8 |
| TRINITY_DN57695_c1_g2 | PF00170 | bZIP_1 | bZIP transcription factor | bZIP | 6.9E-09 | 35.5 |
| TRINITY_DN57617_c1_g5 | PF00170 | bZIP_1 | bZIP transcription factor | bZIP | 0.000000017 | 34.3 |
| TRINITY_DN52830_c0_g2 | PF00170 | bZIP_1 | bZIP transcription factor | bZIP | 0.000000089 | 32 |
| TRINITY_DN52830_c0_g1 | PF00170 | bZIP_1 | bZIP transcription factor | bZIP | 0.000000084 | 32.1 |
| TRINITY_DN62034_c1_g2 | PF00170 | bZIP_1 | bZIP transcription factor | bZIP | 3.9E-18 | 65.2 |
| TRINITY_DN62034_c1_g3 | PF00170 | bZIP_1 | bZIP transcription factor | bZIP | 5E-18 | 64.8 |
| TRINITY_DN33643_c0_g1 | PF00170 | bZIP_1 | bZIP transcription factor | bZIP | 0.000000064 | 32.5 |
| TRINITY_DN55033_c3_g1 | PF00170 | bZIP_1 | bZIP transcription factor | bZIP | 5.5E-09 | 35.9 |
| TRINITY_DN39172_c0_g1 | PF00170 | bZIP_1 | bZIP transcription factor | bZIP | 0.000000013 | 34.6 |
| TRINITY_DN49234_c2_g2 | PF00170 | bZIP_1 | bZIP transcription factor | bZIP | 4.9E-09 | 36 |
| TRINITY_DN63742_c0_g1 | PF00170 | bZIP_1 | bZIP transcription factor | bZIP | 1.6E-15 | 56.8 |
| TRINITY_DN52442_c1_g2 | PF00170 | bZIP_1 | bZIP transcription factor | bZIP | 0.000082 | 22.5 |
| TRINITY_DN61420_c2_g1 | PF00170 | bZIP_1 | bZIP transcription factor | bZIP | 0.000000063 | 32.5 |
| TRINITY_DN61022_c0_g1 | PF00170 | bZIP_1 | bZIP transcription factor | bZIP | 0.000000072 | 32.3 |
| TRINITY_DN70062_c1_g3 | PF00170 | bZIP_1 | bZIP transcription factor | bZIP | 3.4E-13 | 49.3 |
| TRINITY_DN73314_c5_g7 | PF00170 | bZIP_1 | bZIP transcription factor | bZIP | 1.3E-10 | 41.1 |
| TRINITY_DN76513_c2_g1 | PF00170 | bZIP_1 | bZIP transcription factor | bZIP | 1E-10 | 41.4 |
| TRINITY_DN48142_c4_g1 | PF00170 | bZIP_1 | bZIP transcription factor | bZIP | 7.3E-14 | 51.5 |
| TRINITY_DN48142_c4_g4 | PF00170 | bZIP_1 | bZIP transcription factor | bZIP | 1.4E-13 | 50.5 |
| TRINITY_DN56122_c2_g1 | PF00170 | bZIP_1 | bZIP transcription factor | bZIP | 0.00003 | 23.9 |
| TRINITY_DN34164_c1_g1 | PF00170 | bZIP_1 | bZIP transcription factor | bZIP | 0.000000048 | 32.8 |
| TRINITY_DN37922_c0_g1 | PF00170 | bZIP_1 | bZIP transcription factor | bZIP | 0.000021 | 24.4 |
| TRINITY_DN55705_c1_g1 | PF00170 | bZIP_1 | bZIP transcription factor | bZIP | 2.3E-13 | 49.9 |
| TRINITY_DN56966_c2_g2 | PF00170 | bZIP_1 | bZIP transcription factor | bZIP | 9.7E-22 | 76.7 |
| TRINITY_DN56966_c2_g3 | PF00170 | bZIP_1 | bZIP transcription factor | bZIP | 0.000000013 | 34.6 |
| TRINITY_DN56966_c2_g1 | PF00170 | bZIP_1 | bZIP transcription factor | bZIP | 2.1E-18 | 66 |
| TRINITY_DN69151_c0_g1 | PF00170 | bZIP_1 | bZIP transcription factor | bZIP | 1.2E-09 | 38 |
| TRINITY_DN72801_c1_g1 | PF02319 | E2F_TDP | E2F/DP family winged-helix DNA-binding domain | E2F/DP | 9E-24 | 83.2 |
| TRINITY_DN41957_c0_g1 | PF02319 | E2F_TDP | E2F/DP family winged-helix DNA-binding domain | E2F/DP | 2E-21 | 75.7 |
| TRINITY_DN36760_c0_g1 | PF02319 | E2F_TDP | E2F/DP family winged-helix DNA-binding domain | E2F/DP | 1.3E-24 | 85.9 |
| TRINITY_DN36760_c0_g1 | PF02319 | E2F_TDP | E2F/DP family winged-helix DNA-binding domain | E2F/DP | 2.3E-11 | 43.4 |
| TRINITY_DN72467_c0_g1 | PF02319 | E2F_TDP | E2F/DP family winged-helix DNA-binding domain | E2F/DP | 2.5E-17 | 62.5 |
| TRINITY_DN68116_c0_g3 | PF02319 | E2F_TDP | E2F/DP family winged-helix DNA-binding domain | E2F/DP | 4.4E-13 | 48.9 |
| TRINITY_DN48655_c0_g1 | PF02319 | E2F_TDP | E2F/DP family winged-helix DNA-binding domain | E2F/DP | 2.6E-23 | 81.7 |
| TRINITY_DN48655_c1_g1 | PF02319 | E2F_TDP | E2F/DP family winged-helix DNA-binding domain | E2F/DP | 9.7E-26 | 89.5 |
| TRINITY_DN48643_c0_g2 | PF02319 | E2F_TDP | E2F/DP family winged-helix DNA-binding domain | E2F/DP | 1E-41 | 140.7 |
| TRINITY_DN75569_c1_g2 | PF02319 | E2F_TDP | E2F/DP family winged-helix DNA-binding domain | E2F/DP | 6.6E-25 | 86.8 |
| TRINITY_DN71750_c3_g4 | PF04873 | EIN3 | Ethylene insensitive 3 | EIL | 6.5E-125 | 415.4 |
| TRINITY_DN69050_c0_g1 | PF04873 | EIN3 | Ethylene insensitive 3 | EIL | 6.5E-88 | 294.2 |
| TRINITY_DN69050_c0_g5 | PF04873 | EIN3 | Ethylene insensitive 3 | EIL | 1.2E-15 | 57.4 |
| TRINITY_DN69963_c0_g1 | PF04873 | EIN3 | Ethylene insensitive 3 | EIL | 1.5E-128 | 427.3 |
| TRINITY_DN45078_c0_g1 | PF04873 | EIN3 | Ethylene insensitive 3 | EIL | 8.7E-127 | 421.5 |
| TRINITY_DN48225_c0_g2 | PF00249 | Myb_dna_bind | Myb-like DNA-binding domain | MYB_superfamily | 0.00000067 | 29.2 |
| TRINITY_DN48225_c0_g3 | PF00249 | Myb_dna_bind | Myb-like DNA-binding domain | MYB_superfamily | 0.00000094 | 28.8 |
| TRINITY_DN48225_c0_g6 | PF00249 | Myb_dna_bind | Myb-like DNA-binding domain | MYB_superfamily | 0.000000039 | 33.2 |
| TRINITY_DN48225_c0_g5 | PF00249 | Myb_dna_bind | Myb-like DNA-binding domain | MYB_superfamily | 0.00000017 | 31.1 |
| TRINITY_DN48225_c0_g7 | PF00249 | Myb_dna_bind | Myb-like DNA-binding domain | MYB_superfamily | 0.000000025 | 33.8 |
| TRINITY_DN62029_c0_g4 | PF00249 | Myb_dna_bind | Myb-like DNA-binding domain | MYB_superfamily | 0.00000012 | 31.6 |
| TRINITY_DN62029_c0_g3 | PF00249 | Myb_dna_bind | Myb-like DNA-binding domain | MYB_superfamily | 0.00000013 | 31.5 |
| TRINITY_DN63994_c0_g2 | PF00249 | Myb_dna_bind | Myb-like DNA-binding domain | MYB_superfamily | 0.000019 | 24.6 |
| TRINITY_DN16677_c0_g1 | PF00249 | Myb_dna_bind | Myb-like DNA-binding domain | MYB_superfamily | 0.00000001 | 35 |
| TRINITY_DN39763_c0_g1 | PF00249 | Myb_dna_bind | Myb-like DNA-binding domain | MYB_superfamily | 2.3E-49 | 165.1 |
| TRINITY_DN53493_c0_g1 | PF00249 | Myb_dna_bind | Myb-like DNA-binding domain | MYB_superfamily | 4.6E-22 | 77.8 |
| TRINITY_DN68762_c0_g1 | PF00249 | Myb_dna_bind | Myb-like DNA-binding domain | MYB_superfamily | 1.1E-13 | 50.9 |
| TRINITY_DN60157_c2_g1 | PF00249 | Myb_dna_bind | Myb-like DNA-binding domain | MYB_superfamily | 0.00075 | 19.5 |
| TRINITY_DN47988_c7_g10 | PF00249 | Myb_dna_bind | Myb-like DNA-binding domain | MYB_superfamily | 0.000000011 | 34.9 |
| TRINITY_DN47988_c7_g8 | PF00249 | Myb_dna_bind | Myb-like DNA-binding domain | MYB_superfamily | 4.4E-09 | 36.2 |
| TRINITY_DN56295_c2_g1 | PF00249 | Myb_dna_bind | Myb-like DNA-binding domain | MYB_superfamily | 2.7E-35 | 120.1 |
| TRINITY_DN56295_c2_g3 | PF00249 | Myb_dna_bind | Myb-like DNA-binding domain | MYB_superfamily | 9.1E-31 | 105.6 |
| TRINITY_DN71703_c0_g4 | PF00249 | Myb_dna_bind | Myb-like DNA-binding domain | MYB_superfamily | 9.9E-23 | 79.9 |
| TRINITY_DN60694_c0_g2 | PF00249 | Myb_dna_bind | Myb-like DNA-binding domain | MYB_superfamily | 9.4E-19 | 67.2 |
| TRINITY_DN60694_c0_g6 | PF00249 | Myb_dna_bind | Myb-like DNA-binding domain | MYB_superfamily | 0.000054 | 23.1 |
| TRINITY_DN60694_c0_g3 | PF00249 | Myb_dna_bind | Myb-like DNA-binding domain | MYB_superfamily | 0.000000039 | 33.2 |
| TRINITY_DN60694_c0_g3 | PF00249 | Myb_dna_bind | Myb-like DNA-binding domain | MYB_superfamily | 0.00000002 | 34.1 |
| TRINITY_DN60694_c0_g3 | PF00249 | Myb_dna_bind | Myb-like DNA-binding domain | MYB_superfamily | 0.000000014 | 34.6 |
| TRINITY_DN60694_c0_g4 | PF00249 | Myb_dna_bind | Myb-like DNA-binding domain | MYB_superfamily | 1.3E-20 | 73.1 |
| TRINITY_DN62882_c0_g3 | PF00249 | Myb_dna_bind | Myb-like DNA-binding domain | MYB_superfamily | 3E-34 | 116.8 |
| TRINITY_DN62882_c0_g4 | PF00249 | Myb_dna_bind | Myb-like DNA-binding domain | MYB_superfamily | 1.1E-20 | 73.4 |
| TRINITY_DN62882_c0_g5 | PF00249 | Myb_dna_bind | Myb-like DNA-binding domain | MYB_superfamily | 0.000000025 | 33.8 |
| TRINITY_DN62882_c0_g6 | PF00249 | Myb_dna_bind | Myb-like DNA-binding domain | MYB_superfamily | 6.4E-22 | 77.3 |
| TRINITY_DN62882_c1_g1 | PF00249 | Myb_dna_bind | Myb-like DNA-binding domain | MYB_superfamily | 0.00008 | 22.6 |
| TRINITY_DN62882_c1_g1 | PF00249 | Myb_dna_bind | Myb-like DNA-binding domain | MYB_superfamily | 0.000000005 | 36.1 |
| TRINITY_DN62882_c1_g3 | PF00249 | Myb_dna_bind | Myb-like DNA-binding domain | MYB_superfamily | 3.2E-29 | 100.7 |
| TRINITY_DN62882_c1_g4 | PF00249 | Myb_dna_bind | Myb-like DNA-binding domain | MYB_superfamily | 7.1E-31 | 106 |
| TRINITY_DN65496_c3_g1 | PF00249 | Myb_dna_bind | Myb-like DNA-binding domain | MYB_superfamily | 6.2E-11 | 42.2 |
| TRINITY_DN62738_c5_g3 | PF00249 | Myb_dna_bind | Myb-like DNA-binding domain | MYB_superfamily | 5.6E-17 | 61.5 |
| TRINITY_DN45811_c3_g2 | PF00249 | Myb_dna_bind | Myb-like DNA-binding domain | MYB_superfamily | 0.000000024 | 33.9 |
| TRINITY_DN45811_c3_g1 | PF00249 | Myb_dna_bind | Myb-like DNA-binding domain | MYB_superfamily | 6.7E-09 | 35.6 |
| TRINITY_DN72321_c0_g2 | PF00249 | Myb_dna_bind | Myb-like DNA-binding domain | MYB_superfamily | 1.1E-15 | 57.4 |
| TRINITY_DN69674_c3_g1 | PF00249 | Myb_dna_bind | Myb-like DNA-binding domain | MYB_superfamily | 1.1E-32 | 111.7 |
| TRINITY_DN50641_c1_g1 | PF00249 | Myb_dna_bind | Myb-like DNA-binding domain | MYB_superfamily | 8.7E-12 | 44.9 |
| TRINITY_DN67796_c1_g1 | PF00249 | Myb_dna_bind | Myb-like DNA-binding domain | MYB_superfamily | 0.000000064 | 32.5 |
| TRINITY_DN67799_c0_g2 | PF00249 | Myb_dna_bind | Myb-like DNA-binding domain | MYB_superfamily | 1.8E-10 | 40.7 |
| TRINITY_DN51008_c5_g1 | PF00249 | Myb_dna_bind | Myb-like DNA-binding domain | MYB_superfamily | 0.00000053 | 29.6 |
| TRINITY_DN41882_c0_g1 | PF00249 | Myb_dna_bind | Myb-like DNA-binding domain | MYB_superfamily | 1.1E-09 | 38.2 |
| TRINITY_DN53960_c5_g1 | PF00249 | Myb_dna_bind | Myb-like DNA-binding domain | MYB_superfamily | 3.2E-13 | 49.5 |
| TRINITY_DN48347_c1_g1 | PF00249 | Myb_dna_bind | Myb-like DNA-binding domain | MYB_superfamily | 0.00011 | 22.1 |
| TRINITY_DN73223_c6_g4 | PF00249 | Myb_dna_bind | Myb-like DNA-binding domain | MYB_superfamily | 8.7E-20 | 70.5 |
| TRINITY_DN57900_c5_g1 | PF00249 | Myb_dna_bind | Myb-like DNA-binding domain | MYB_superfamily | 3.8E-50 | 167.7 |
| TRINITY_DN75574_c1_g1 | PF00249 | Myb_dna_bind | Myb-like DNA-binding domain | MYB_superfamily | 0.0000031 | 27.1 |
| TRINITY_DN43426_c0_g1 | PF00249 | Myb_dna_bind | Myb-like DNA-binding domain | MYB_superfamily | 3.2E-17 | 62.3 |
| TRINITY_DN43426_c0_g2 | PF00249 | Myb_dna_bind | Myb-like DNA-binding domain | MYB_superfamily | 8.7E-31 | 105.7 |
| TRINITY_DN43426_c0_g3 | PF00249 | Myb_dna_bind | Myb-like DNA-binding domain | MYB_superfamily | 8E-33 | 112.2 |
| TRINITY_DN52089_c3_g2 | PF00249 | Myb_dna_bind | Myb-like DNA-binding domain | MYB_superfamily | 0.0003 | 20.8 |
| TRINITY_DN65018_c0_g1 | PF00249 | Myb_dna_bind | Myb-like DNA-binding domain | MYB_superfamily | 3.7E-54 | 180.5 |
| TRINITY_DN65018_c1_g1 | PF00249 | Myb_dna_bind | Myb-like DNA-binding domain | MYB_superfamily | 9.9E-52 | 172.8 |
| TRINITY_DN45922_c0_g1 | PF00249 | Myb_dna_bind | Myb-like DNA-binding domain | MYB_superfamily | 4.3E-28 | 97.1 |
| TRINITY_DN45950_c0_g2 | PF00249 | Myb_dna_bind | Myb-like DNA-binding domain | MYB_superfamily | 6.8E-24 | 83.6 |
| TRINITY_DN65386_c2_g3 | PF00249 | Myb_dna_bind | Myb-like DNA-binding domain | MYB_superfamily | 0.000053 | 23.2 |
| TRINITY_DN65386_c3_g2 | PF00249 | Myb_dna_bind | Myb-like DNA-binding domain | MYB_superfamily | 0.0000054 | 26.3 |
| TRINITY_DN65386_c3_g3 | PF00249 | Myb_dna_bind | Myb-like DNA-binding domain | MYB_superfamily | 0.0000063 | 26.1 |
| TRINITY_DN76002_c5_g3 | PF00249 | Myb_dna_bind | Myb-like DNA-binding domain | MYB_superfamily | 4E-13 | 49.2 |
| TRINITY_DN40720_c0_g1 | PF00249 | Myb_dna_bind | Myb-like DNA-binding domain | MYB_superfamily | 1.9E-33 | 114.2 |
| TRINITY_DN47880_c0_g2 | PF00249 | Myb_dna_bind | Myb-like DNA-binding domain | MYB_superfamily | 1.6E-32 | 111.2 |
| TRINITY_DN47880_c0_g3 | PF00249 | Myb_dna_bind | Myb-like DNA-binding domain | MYB_superfamily | 9.6E-33 | 112 |
| TRINITY_DN47880_c0_g5 | PF00249 | Myb_dna_bind | Myb-like DNA-binding domain | MYB_superfamily | 0.000000043 | 33.1 |
| TRINITY_DN44732_c0_g1 | PF00249 | Myb_dna_bind | Myb-like DNA-binding domain | MYB_superfamily | 1.3E-29 | 101.9 |
| TRINITY_DN67862_c0_g1 | PF00249 | Myb_dna_bind | Myb-like DNA-binding domain | MYB_superfamily | 5.9E-10 | 39 |
| TRINITY_DN42132_c0_g2 | PF00249 | Myb_dna_bind | Myb-like DNA-binding domain | MYB_superfamily | 4.4E-49 | 164.3 |
| TRINITY_DN72224_c1_g1 | PF00249 | Myb_dna_bind | Myb-like DNA-binding domain | MYB_superfamily | 6.4E-33 | 112.5 |
| TRINITY_DN72224_c2_g3 | PF00249 | Myb_dna_bind | Myb-like DNA-binding domain | MYB_superfamily | 7.6E-14 | 51.5 |
| TRINITY_DN72224_c2_g4 | PF00249 | Myb_dna_bind | Myb-like DNA-binding domain | MYB_superfamily | 3.7E-31 | 106.9 |
| TRINITY_DN66566_c0_g6 | PF00249 | Myb_dna_bind | Myb-like DNA-binding domain | MYB_superfamily | 0.000016 | 24.9 |
| TRINITY_DN29750_c0_g1 | PF00249 | Myb_dna_bind | Myb-like DNA-binding domain | MYB_superfamily | 0.000000026 | 33.7 |
| TRINITY_DN9522_c0_g1 | PF00249 | Myb_dna_bind | Myb-like DNA-binding domain | MYB_superfamily | 5.8E-15 | 55 |
| TRINITY_DN53737_c0_g1 | PF00249 | Myb_dna_bind | Myb-like DNA-binding domain | MYB_superfamily | 4.7E-29 | 100.1 |
| TRINITY_DN44685_c1_g1 | PF00249 | Myb_dna_bind | Myb-like DNA-binding domain | MYB_superfamily | 6.4E-10 | 38.9 |
| TRINITY_DN53508_c2_g6 | PF00249 | Myb_dna_bind | Myb-like DNA-binding domain | MYB_superfamily | 6.1E-11 | 42.2 |
| TRINITY_DN87702_c0_g1 | PF00249 | Myb_dna_bind | Myb-like DNA-binding domain | MYB_superfamily | 3.2E-10 | 39.9 |
| TRINITY_DN60579_c1_g2 | PF00249 | Myb_dna_bind | Myb-like DNA-binding domain | MYB_superfamily | 3.6E-13 | 49.3 |
| TRINITY_DN60560_c2_g1 | PF00249 | Myb_dna_bind | Myb-like DNA-binding domain | MYB_superfamily | 9E-16 | 57.6 |
| TRINITY_DN76223_c4_g2 | PF00249 | Myb_dna_bind | Myb-like DNA-binding domain | MYB_superfamily | 0.000000049 | 32.9 |
| TRINITY_DN21863_c0_g1 | PF00249 | Myb_dna_bind | Myb-like DNA-binding domain | MYB_superfamily | 0.0000031 | 27.1 |
| TRINITY_DN58242_c1_g1 | PF00249 | Myb_dna_bind | Myb-like DNA-binding domain | MYB_superfamily | 0.00000011 | 31.8 |
| TRINITY_DN47309_c1_g3 | PF00249 | Myb_dna_bind | Myb-like DNA-binding domain | MYB_superfamily | 0.00000029 | 30.4 |
| TRINITY_DN47309_c1_g6 | PF00249 | Myb_dna_bind | Myb-like DNA-binding domain | MYB_superfamily | 0.00000048 | 29.7 |
| TRINITY_DN47309_c1_g2 | PF00249 | Myb_dna_bind | Myb-like DNA-binding domain | MYB_superfamily | 0.00000042 | 29.9 |
| TRINITY_DN49628_c0_g3 | PF00249 | Myb_dna_bind | Myb-like DNA-binding domain | MYB_superfamily | 6.2E-12 | 45.3 |
| TRINITY_DN69160_c0_g1 | PF00249 | Myb_dna_bind | Myb-like DNA-binding domain | MYB_superfamily | 0.000097 | 22.3 |
| TRINITY_DN64707_c0_g1 | PF00249 | Myb_dna_bind | Myb-like DNA-binding domain | MYB_superfamily | 2.4E-16 | 59.5 |
| TRINITY_DN47007_c3_g7 | PF00249 | Myb_dna_bind | Myb-like DNA-binding domain | MYB_superfamily | 0.000000015 | 34.5 |
| TRINITY_DN58371_c2_g1 | PF00249 | Myb_dna_bind | Myb-like DNA-binding domain | MYB_superfamily | 2.8E-09 | 36.8 |
| TRINITY_DN53009_c0_g2 | PF00249 | Myb_dna_bind | Myb-like DNA-binding domain | MYB_superfamily | 5.5E-12 | 45.5 |
| TRINITY_DN75331_c2_g7 | PF00249 | Myb_dna_bind | Myb-like DNA-binding domain | MYB_superfamily | 2.2E-09 | 37.2 |
| TRINITY_DN68196_c1_g1 | PF00249 | Myb_dna_bind | Myb-like DNA-binding domain | MYB_superfamily | 0.000042 | 23.5 |
| TRINITY_DN74205_c0_g1 | PF00249 | Myb_dna_bind | Myb-like DNA-binding domain | MYB_superfamily | 0.000000099 | 31.9 |
| TRINITY_DN68581_c6_g3 | PF00249 | Myb_dna_bind | Myb-like DNA-binding domain | MYB_superfamily | 6.5E-09 | 35.7 |
| TRINITY_DN49422_c1_g2 | PF00249 | Myb_dna_bind | Myb-like DNA-binding domain | MYB_superfamily | 8.9E-10 | 38.4 |
| TRINITY_DN42509_c0_g1 | PF00249 | Myb_dna_bind | Myb-like DNA-binding domain | MYB_superfamily | 5.2E-09 | 36 |
| TRINITY_DN42555_c0_g3 | PF00249 | Myb_dna_bind | Myb-like DNA-binding domain | MYB_superfamily | 0.00000045 | 29.8 |
| TRINITY_DN51873_c0_g1 | PF00249 | Myb_dna_bind | Myb-like DNA-binding domain | MYB_superfamily | 0.00037 | 20.5 |
| TRINITY_DN48528_c2_g2 | PF00249 | Myb_dna_bind | Myb-like DNA-binding domain | MYB_superfamily | 0.000000013 | 34.7 |
| TRINITY_DN19038_c0_g2 | PF00249 | Myb_dna_bind | Myb-like DNA-binding domain | MYB_superfamily | 4.8E-21 | 74.5 |
| TRINITY_DN50455_c1_g1 | PF00249 | Myb_dna_bind | Myb-like DNA-binding domain | MYB_superfamily | 2.1E-17 | 62.9 |
| TRINITY_DN50455_c1_g1 | PF00249 | Myb_dna_bind | Myb-like DNA-binding domain | MYB_superfamily | 0.0000011 | 28.5 |
| TRINITY_DN50438_c0_g1 | PF00249 | Myb_dna_bind | Myb-like DNA-binding domain | MYB_superfamily | 9.1E-09 | 35.2 |
| TRINITY_DN5796_c0_g1 | PF00249 | Myb_dna_bind | Myb-like DNA-binding domain | MYB_superfamily | 0.000000013 | 34.7 |
| TRINITY_DN75833_c12_g1 | PF00249 | Myb_dna_bind | Myb-like DNA-binding domain | MYB_superfamily | 2.3E-33 | 114 |
| TRINITY_DN42908_c0_g1 | PF00249 | Myb_dna_bind | Myb-like DNA-binding domain | MYB_superfamily | 2.8E-09 | 36.8 |
| TRINITY_DN62481_c0_g1 | PF00249 | Myb_dna_bind | Myb-like DNA-binding domain | MYB_superfamily | 8.7E-27 | 92.9 |
| TRINITY_DN60612_c2_g2 | PF00249 | Myb_dna_bind | Myb-like DNA-binding domain | MYB_superfamily | 0.0000026 | 27.3 |
| TRINITY_DN73366_c1_g2 | PF00249 | Myb_dna_bind | Myb-like DNA-binding domain | MYB_superfamily | 2.3E-34 | 117.2 |
| TRINITY_DN73366_c2_g1 | PF00249 | Myb_dna_bind | Myb-like DNA-binding domain | MYB_superfamily | 2.3E-34 | 117.2 |
| TRINITY_DN56768_c2_g4 | PF00249 | Myb_dna_bind | Myb-like DNA-binding domain | MYB_superfamily | 1.3E-22 | 79.5 |
| TRINITY_DN56768_c2_g2 | PF00249 | Myb_dna_bind | Myb-like DNA-binding domain | MYB_superfamily | 1.3E-12 | 47.5 |
| TRINITY_DN56768_c2_g5 | PF00249 | Myb_dna_bind | Myb-like DNA-binding domain | MYB_superfamily | 1.9E-32 | 111 |
| TRINITY_DN44112_c0_g2 | PF00249 | Myb_dna_bind | Myb-like DNA-binding domain | MYB_superfamily | 0.00000024 | 30.6 |
| TRINITY_DN44112_c0_g1 | PF00249 | Myb_dna_bind | Myb-like DNA-binding domain | MYB_superfamily | 0.0000003 | 30.3 |
| TRINITY_DN54469_c0_g2 | PF00249 | Myb_dna_bind | Myb-like DNA-binding domain | MYB_superfamily | 9.4E-09 | 35.2 |
| TRINITY_DN59551_c1_g2 | PF00249 | Myb_dna_bind | Myb-like DNA-binding domain | MYB_superfamily | 0.0000049 | 26.5 |
| TRINITY_DN55961_c1_g2 | PF00249 | Myb_dna_bind | Myb-like DNA-binding domain | MYB_superfamily | 0.00000041 | 29.9 |
| TRINITY_DN73968_c1_g6 | PF00249 | Myb_dna_bind | Myb-like DNA-binding domain | MYB_superfamily | 7.7E-18 | 64.2 |
| TRINITY_DN63637_c0_g1 | PF00249 | Myb_dna_bind | Myb-like DNA-binding domain | MYB_superfamily | 6.1E-10 | 39 |
| TRINITY_DN70684_c8_g1 | PF00249 | Myb_dna_bind | Myb-like DNA-binding domain | MYB_superfamily | 0.00000014 | 31.5 |
| TRINITY_DN72124_c1_g5 | PF00249 | Myb_dna_bind | Myb-like DNA-binding domain | MYB_superfamily | 3.3E-50 | 167.9 |
| TRINITY_DN40079_c0_g1 | PF00249 | Myb_dna_bind | Myb-like DNA-binding domain | MYB_superfamily | 5.4E-30 | 103.2 |
| TRINITY_DN62831_c0_g1 | PF00249 | Myb_dna_bind | Myb-like DNA-binding domain | MYB_superfamily | 0.00038 | 20.4 |
| TRINITY_DN4829_c0_g1 | PF00249 | Myb_dna_bind | Myb-like DNA-binding domain | MYB_superfamily | 1.2E-32 | 111.7 |
| TRINITY_DN55930_c2_g2 | PF00249 | Myb_dna_bind | Myb-like DNA-binding domain | MYB_superfamily | 0.0000015 | 28.1 |
| TRINITY_DN55930_c2_g3 | PF00249 | Myb_dna_bind | Myb-like DNA-binding domain | MYB_superfamily | 7.5E-11 | 41.9 |
| TRINITY_DN76008_c8_g1 | PF00249 | Myb_dna_bind | Myb-like DNA-binding domain | MYB_superfamily | 2E-25 | 88.5 |
| TRINITY_DN76008_c8_g2 | PF00249 | Myb_dna_bind | Myb-like DNA-binding domain | MYB_superfamily | 9.1E-35 | 118.4 |
| TRINITY_DN55482_c0_g1 | PF00249 | Myb_dna_bind | Myb-like DNA-binding domain | MYB_superfamily | 1.8E-09 | 37.4 |
| TRINITY_DN55482_c1_g2 | PF00249 | Myb_dna_bind | Myb-like DNA-binding domain | MYB_superfamily | 2.8E-10 | 40 |
| TRINITY_DN71156_c3_g2 | PF00249 | Myb_dna_bind | Myb-like DNA-binding domain | MYB_superfamily | 3.2E-19 | 68.7 |
| TRINITY_DN66830_c6_g2 | PF00249 | Myb_dna_bind | Myb-like DNA-binding domain | MYB_superfamily | 2.1E-09 | 37.2 |
| TRINITY_DN60784_c3_g2 | PF00249 | Myb_dna_bind | Myb-like DNA-binding domain | MYB_superfamily | 0.000000055 | 32.7 |
| TRINITY_DN42660_c0_g1 | PF00249 | Myb_dna_bind | Myb-like DNA-binding domain | MYB_superfamily | 5E-31 | 106.5 |
| TRINITY_DN66186_c1_g1 | PF00249 | Myb_dna_bind | Myb-like DNA-binding domain | MYB_superfamily | 0.000000012 | 34.8 |
| TRINITY_DN62078_c1_g2 | PF00249 | Myb_dna_bind | Myb-like DNA-binding domain | MYB_superfamily | 6.5E-34 | 115.7 |
| TRINITY_DN62078_c2_g2 | PF00249 | Myb_dna_bind | Myb-like DNA-binding domain | MYB_superfamily | 0.0000021 | 27.7 |
| TRINITY_DN62099_c2_g1 | PF00249 | Myb_dna_bind | Myb-like DNA-binding domain | MYB_superfamily | 0.00000086 | 28.9 |
| TRINITY_DN70669_c7_g1 | PF00249 | Myb_dna_bind | Myb-like DNA-binding domain | MYB_superfamily | 0.0000044 | 26.6 |
| TRINITY_DN68105_c0_g2 | PF00249 | Myb_dna_bind | Myb-like DNA-binding domain | MYB_superfamily | 0.00013 | 21.9 |
| TRINITY_DN71279_c0_g1 | PF00249 | Myb_dna_bind | Myb-like DNA-binding domain | MYB_superfamily | 0.000051 | 23.2 |
| TRINITY_DN46451_c4_g2 | PF00249 | Myb_dna_bind | Myb-like DNA-binding domain | MYB_superfamily | 0.000000027 | 33.7 |
| TRINITY_DN44961_c0_g1 | PF00249 | Myb_dna_bind | Myb-like DNA-binding domain | MYB_superfamily | 8.1E-12 | 45 |
| TRINITY_DN64290_c3_g2 | PF00249 | Myb_dna_bind | Myb-like DNA-binding domain | MYB_superfamily | 2.2E-19 | 69.2 |
| TRINITY_DN64290_c3_g4 | PF00249 | Myb_dna_bind | Myb-like DNA-binding domain | MYB_superfamily | 1.2E-26 | 92.5 |
| TRINITY_DN50381_c3_g1 | PF00249 | Myb_dna_bind | Myb-like DNA-binding domain | MYB_superfamily | 7.7E-09 | 35.4 |
| TRINITY_DN73574_c3_g1 | PF00249 | Myb_dna_bind | Myb-like DNA-binding domain | MYB_superfamily | 0.00001 | 25.4 |
| TRINITY_DN59706_c0_g2 | PF00249 | Myb_dna_bind | Myb-like DNA-binding domain | MYB_superfamily | 3.4E-31 | 107 |
| TRINITY_DN91980_c0_g1 | PF00249 | Myb_dna_bind | Myb-like DNA-binding domain | MYB_superfamily | 4.7E-29 | 100.2 |
| TRINITY_DN46487_c0_g1 | PF00249 | Myb_dna_bind | Myb-like DNA-binding domain | MYB_superfamily | 1.6E-09 | 37.6 |
| TRINITY_DN46487_c1_g2 | PF00249 | Myb_dna_bind | Myb-like DNA-binding domain | MYB_superfamily | 0.000000094 | 32 |
| TRINITY_DN62113_c0_g1 | PF00249 | Myb_dna_bind | Myb-like DNA-binding domain | MYB_superfamily | 0.00000027 | 30.5 |
| TRINITY_DN62113_c0_g1 | PF00249 | Myb_dna_bind | Myb-like DNA-binding domain | MYB_superfamily | 0.0000072 | 25.9 |
| TRINITY_DN62113_c0_g1 | PF00249 | Myb_dna_bind | Myb-like DNA-binding domain | MYB_superfamily | 1.7E-10 | 40.7 |
| TRINITY_DN48186_c0_g1 | PF00249 | Myb_dna_bind | Myb-like DNA-binding domain | MYB_superfamily | 1E-28 | 99 |
| TRINITY_DN28777_c0_g1 | PF00249 | Myb_dna_bind | Myb-like DNA-binding domain | MYB_superfamily | 1.9E-22 | 79 |
| TRINITY_DN56646_c2_g5 | PF00249 | Myb_dna_bind | Myb-like DNA-binding domain | MYB_superfamily | 3.7E-34 | 116.5 |
| TRINITY_DN56646_c2_g7 | PF00249 | Myb_dna_bind | Myb-like DNA-binding domain | MYB_superfamily | 5.3E-30 | 103.2 |
| TRINITY_DN56646_c2_g9 | PF00249 | Myb_dna_bind | Myb-like DNA-binding domain | MYB_superfamily | 0.00000013 | 31.5 |
| TRINITY_DN56646_c2_g10 | PF00249 | Myb_dna_bind | Myb-like DNA-binding domain | MYB_superfamily | 5.3E-29 | 100 |
| TRINITY_DN54228_c1_g3 | PF00249 | Myb_dna_bind | Myb-like DNA-binding domain | MYB_superfamily | 0.00076 | 19.4 |
| TRINITY_DN58423_c2_g3 | PF00249 | Myb_dna_bind | Myb-like DNA-binding domain | MYB_superfamily | 0.0000001 | 31.8 |
| TRINITY_DN52106_c0_g3 | PF00249 | Myb_dna_bind | Myb-like DNA-binding domain | MYB_superfamily | 0.0000003 | 30.4 |
| TRINITY_DN75239_c4_g1 | PF00249 | Myb_dna_bind | Myb-like DNA-binding domain | MYB_superfamily | 0.000089 | 22.4 |
| TRINITY_DN53884_c2_g2 | PF00249 | Myb_dna_bind | Myb-like DNA-binding domain | MYB_superfamily | 0.0000051 | 26.4 |
| TRINITY_DN49789_c0_g4 | PF00249 | Myb_dna_bind | Myb-like DNA-binding domain | MYB_superfamily | 2.9E-10 | 40 |
| TRINITY_DN73261_c3_g1 | PF00249 | Myb_dna_bind | Myb-like DNA-binding domain | MYB_superfamily | 2.8E-13 | 49.6 |
| TRINITY_DN51168_c5_g1 | PF00249 | Myb_dna_bind | Myb-like DNA-binding domain | MYB_superfamily | 0.000000029 | 33.6 |
| TRINITY_DN47468_c3_g2 | PF00249 | Myb_dna_bind | Myb-like DNA-binding domain | MYB_superfamily | 7.1E-09 | 35.6 |
| TRINITY_DN45166_c0_g2 | PF00249 | Myb_dna_bind | Myb-like DNA-binding domain | MYB_superfamily | 1.5E-22 | 79.3 |
| TRINITY_DN45166_c0_g1 | PF00249 | Myb_dna_bind | Myb-like DNA-binding domain | MYB_superfamily | 0.00000032 | 30.2 |
| TRINITY_DN45166_c0_g1 | PF00249 | Myb_dna_bind | Myb-like DNA-binding domain | MYB_superfamily | 0.000027 | 24.1 |
| TRINITY_DN73788_c3_g4 | PF00249 | Myb_dna_bind | Myb-like DNA-binding domain | MYB_superfamily | 4.8E-33 | 112.9 |
| TRINITY_DN73788_c3_g5 | PF00249 | Myb_dna_bind | Myb-like DNA-binding domain | MYB_superfamily | 2E-20 | 72.6 |
| TRINITY_DN52814_c1_g1 | PF00249 | Myb_dna_bind | Myb-like DNA-binding domain | MYB_superfamily | 8.6E-09 | 35.3 |
| TRINITY_DN46300_c1_g1 | PF00249 | Myb_dna_bind | Myb-like DNA-binding domain | MYB_superfamily | 0.00000099 | 28.7 |
| TRINITY_DN46300_c1_g2 | PF00249 | Myb_dna_bind | Myb-like DNA-binding domain | MYB_superfamily | 0.000000011 | 35 |
| TRINITY_DN77130_c0_g1 | PF00249 | Myb_dna_bind | Myb-like DNA-binding domain | MYB_superfamily | 3E-33 | 113.6 |
| TRINITY_DN89728_c0_g1 | PF00249 | Myb_dna_bind | Myb-like DNA-binding domain | MYB_superfamily | 8.2E-12 | 45 |
| TRINITY_DN75304_c4_g1 | PF00249 | Myb_dna_bind | Myb-like DNA-binding domain | MYB_superfamily | 0.00000021 | 30.8 |
| TRINITY_DN66148_c0_g1 | PF02362 | B3 | B3 DNA binding domain | B3_superfamily | 4.2E-20 | 71.4 |
| TRINITY_DN57170_c0_g1 | PF02362 | B3 | B3 DNA binding domain | B3_superfamily | 1.9E-28 | 98.1 |
| TRINITY_DN43446_c0_g1 | PF02362 | B3 | B3 DNA binding domain | B3_superfamily | 6.2E-22 | 77.2 |
| TRINITY_DN66770_c5_g3 | PF02362 | B3 | B3 DNA binding domain | B3_superfamily | 2.2E-22 | 78.7 |
| TRINITY_DN66770_c5_g1 | PF02362 | B3 | B3 DNA binding domain | B3_superfamily | 1.9E-17 | 62.8 |
| TRINITY_DN47735_c4_g1 | PF02362 | B3 | B3 DNA binding domain | B3_superfamily | 1.4E-11 | 44 |
| TRINITY_DN53475_c0_g2 | PF02362 | B3 | B3 DNA binding domain | B3_superfamily | 6.4E-16 | 57.9 |
| TRINITY_DN56007_c2_g2 | PF02362 | B3 | B3 DNA binding domain | B3_superfamily | 1.7E-15 | 56.6 |
| TRINITY_DN45611_c2_g1 | PF02362 | B3 | B3 DNA binding domain | B3_superfamily | 2E-23 | 82.1 |
| TRINITY_DN74260_c1_g1 | PF02362 | B3 | B3 DNA binding domain | B3_superfamily | 0.000011 | 25.1 |
| TRINITY_DN79585_c0_g1 | PF02362 | B3 | B3 DNA binding domain | B3_superfamily | 1.5E-17 | 63.2 |
| TRINITY_DN89161_c0_g1 | PF02362 | B3 | B3 DNA binding domain | B3_superfamily | 0.000098 | 22 |
| TRINITY_DN69295_c0_g3 | PF02362 | B3 | B3 DNA binding domain | B3_superfamily | 1.9E-21 | 75.7 |
| TRINITY_DN73480_c0_g1 | PF02362 | B3 | B3 DNA binding domain | B3_superfamily | 6E-15 | 54.8 |
| TRINITY_DN50211_c3_g1 | PF02362 | B3 | B3 DNA binding domain | B3_superfamily | 3.7E-15 | 55.5 |
| TRINITY_DN58494_c2_g1 | PF02362 | B3 | B3 DNA binding domain | B3_superfamily | 8.9E-26 | 89.6 |
| TRINITY_DN58494_c2_g4 | PF02362 | B3 | B3 DNA binding domain | B3_superfamily | 3.2E-25 | 87.8 |
| TRINITY_DN58494_c2_g4 | PF00847 | AP2 | AP2 domain | AP2/ERF | 0.00000022 | 30.9 |
| TRINITY_DN58494_c2_g8 | PF02362 | B3 | B3 DNA binding domain | B3_superfamily | 6.4E-26 | 90.1 |
| TRINITY_DN64706_c1_g2 | PF02362 | B3 | B3 DNA binding domain | B3_superfamily | 1.2E-16 | 60.3 |
| TRINITY_DN67245_c0_g2 | PF02362 | B3 | B3 DNA binding domain | B3_superfamily | 3.5E-09 | 36.3 |
| TRINITY_DN67245_c0_g1 | PF02362 | B3 | B3 DNA binding domain | B3_superfamily | 2E-13 | 50 |
| TRINITY_DN57384_c1_g3 | PF02362 | B3 | B3 DNA binding domain | B3_superfamily | 8.5E-23 | 80 |
| TRINITY_DN63021_c2_g2 | PF02362 | B3 | B3 DNA binding domain | B3_superfamily | 6.8E-24 | 83.5 |
| TRINITY_DN63021_c2_g1 | PF02362 | B3 | B3 DNA binding domain | B3_superfamily | 1.2E-22 | 79.6 |
| TRINITY_DN51929_c3_g2 | PF02362 | B3 | B3 DNA binding domain | B3_superfamily | 0.00039 | 20.1 |
| TRINITY_DN51929_c3_g1 | PF02362 | B3 | B3 DNA binding domain | B3_superfamily | 3.7E-22 | 78 |
| TRINITY_DN65424_c1_g1 | PF02362 | B3 | B3 DNA binding domain | B3_superfamily | 7.4E-20 | 70.6 |
| TRINITY_DN74093_c1_g1 | PF02362 | B3 | B3 DNA binding domain | B3_superfamily | 0.00000046 | 29.5 |
| TRINITY_DN74093_c1_g1 | PF02362 | B3 | B3 DNA binding domain | B3_superfamily | 0.000011 | 25.2 |
| TRINITY_DN52625_c0_g2 | PF02362 | B3 | B3 DNA binding domain | B3_superfamily | 1.7E-21 | 75.8 |
| TRINITY_DN53486_c1_g4 | PF02362 | B3 | B3 DNA binding domain | B3_superfamily | 2E-22 | 78.8 |
| TRINITY_DN70858_c0_g3 | PF02362 | B3 | B3 DNA binding domain | B3_superfamily | 1.5E-12 | 47.2 |
| TRINITY_DN70858_c0_g5 | PF02362 | B3 | B3 DNA binding domain | B3_superfamily | 3.9E-09 | 36.2 |
| TRINITY_DN70858_c0_g6 | PF02362 | B3 | B3 DNA binding domain | B3_superfamily | 0.00000057 | 29.2 |
| TRINITY_DN55049_c4_g2 | PF02362 | B3 | B3 DNA binding domain | B3_superfamily | 2.7E-32 | 110.5 |
| TRINITY_DN54094_c6_g2 | PF02362 | B3 | B3 DNA binding domain | B3_superfamily | 3.1E-35 | 120 |
| TRINITY_DN54094_c6_g4 | PF02362 | B3 | B3 DNA binding domain | B3_superfamily | 2.2E-34 | 117.2 |
| TRINITY_DN54094_c6_g5 | PF02362 | B3 | B3 DNA binding domain | B3_superfamily | 6.3E-36 | 122.2 |
| TRINITY_DN54094_c6_g1 | PF02362 | B3 | B3 DNA binding domain | B3_superfamily | 6.3E-37 | 125.4 |
| TRINITY_DN54094_c6_g6 | PF02362 | B3 | B3 DNA binding domain | B3_superfamily | 6.4E-36 | 122.2 |
| TRINITY_DN54094_c6_g7 | PF02362 | B3 | B3 DNA binding domain | B3_superfamily | 3.9E-36 | 122.9 |
| TRINITY_DN54094_c6_g10 | PF02362 | B3 | B3 DNA binding domain | B3_superfamily | 3E-20 | 71.8 |
| TRINITY_DN47948_c4_g1 | PF02362 | B3 | B3 DNA binding domain | B3_superfamily | 1.7E-28 | 98.3 |
| TRINITY_DN67657_c5_g4 | PF02362 | B3 | B3 DNA binding domain | B3_superfamily | 6.7E-18 | 64.3 |
| TRINITY_DN43762_c0_g1 | PF02362 | B3 | B3 DNA binding domain | B3_superfamily | 3.3E-12 | 46 |
| TRINITY_DN61784_c2_g1 | PF02362 | B3 | B3 DNA binding domain | B3_superfamily | 5.8E-36 | 122.3 |
| TRINITY_DN61784_c2_g2 | PF02362 | B3 | B3 DNA binding domain | B3_superfamily | 9.8E-13 | 47.7 |
| TRINITY_DN74058_c0_g2 | PF02362 | B3 | B3 DNA binding domain | B3_superfamily | 2.4E-23 | 81.8 |
| TRINITY_DN54256_c0_g2 | PF02362 | B3 | B3 DNA binding domain | B3_superfamily | 2E-21 | 75.6 |
| TRINITY_DN37129_c0_g1 | PF02362 | B3 | B3 DNA binding domain | B3_superfamily | 1.2E-19 | 69.9 |
| TRINITY_DN58480_c2_g3 | PF02362 | B3 | B3 DNA binding domain | B3_superfamily | 8.1E-13 | 48 |
| TRINITY_DN58480_c2_g5 | PF02362 | B3 | B3 DNA binding domain | B3_superfamily | 7.1E-16 | 57.8 |
| TRINITY_DN58480_c2_g4 | PF02362 | B3 | B3 DNA binding domain | B3_superfamily | 8.8E-23 | 80 |
| TRINITY_DN58480_c2_g2 | PF02362 | B3 | B3 DNA binding domain | B3_superfamily | 1.7E-25 | 88.7 |
| TRINITY_DN63366_c1_g1 | PF02362 | B3 | B3 DNA binding domain | B3_superfamily | 7.5E-31 | 105.9 |
| TRINITY_DN63366_c1_g2 | PF02362 | B3 | B3 DNA binding domain | B3_superfamily | 7.7E-30 | 102.6 |
| TRINITY_DN63366_c1_g3 | PF02362 | B3 | B3 DNA binding domain | B3_superfamily | 7.6E-30 | 102.7 |
| TRINITY_DN56107_c1_g5 | PF02362 | B3 | B3 DNA binding domain | B3_superfamily | 0.00000006 | 32.4 |
| TRINITY_DN53830_c0_g1 | PF02362 | B3 | B3 DNA binding domain | B3_superfamily | 4.6E-35 | 119.4 |
| TRINITY_DN56591_c1_g2 | PF02362 | B3 | B3 DNA binding domain | B3_superfamily | 1.7E-12 | 46.9 |
| TRINITY_DN56591_c1_g1 | PF02362 | B3 | B3 DNA binding domain | B3_superfamily | 3.3E-24 | 84.5 |
| TRINITY_DN56591_c1_g3 | PF02362 | B3 | B3 DNA binding domain | B3_superfamily | 1.6E-27 | 95.2 |
| TRINITY_DN56591_c1_g3 | PF00847 | AP2 | AP2 domain | AP2/ERF | 5.3E-09 | 36.1 |
| TRINITY_DN56591_c1_g4 | PF02362 | B3 | B3 DNA binding domain | B3_superfamily | 1.3E-25 | 89.1 |
| TRINITY_DN56591_c1_g5 | PF02362 | B3 | B3 DNA binding domain | B3_superfamily | 1.6E-26 | 92 |
| TRINITY_DN56591_c1_g6 | PF02362 | B3 | B3 DNA binding domain | B3_superfamily | 6.5E-16 | 57.9 |
| TRINITY_DN44020_c0_g1 | PF02362 | B3 | B3 DNA binding domain | B3_superfamily | 0.00000098 | 28.5 |
| TRINITY_DN44020_c0_g1 | PF02362 | B3 | B3 DNA binding domain | B3_superfamily | 0.0000021 | 27.4 |
| TRINITY_DN47534_c0_g1 | PF00010 | HLH | Helix-loop-helix DNA-binding domain | bHLH | 0.00000011 | 31.5 |
| TRINITY_DN72370_c1_g2 | PF00010 | HLH | Helix-loop-helix DNA-binding domain | bHLH | 0.00000015 | 31.1 |
| TRINITY_DN53250_c1_g2 | PF00010 | HLH | Helix-loop-helix DNA-binding domain | bHLH | 3.2E-15 | 55.7 |
| TRINITY_DN65905_c0_g3 | PF00010 | HLH | Helix-loop-helix DNA-binding domain | bHLH | 0.000029 | 23.8 |
| TRINITY_DN71084_c0_g1 | PF00010 | HLH | Helix-loop-helix DNA-binding domain | bHLH | 4E-11 | 42.6 |
| TRINITY_DN59682_c3_g4 | PF00010 | HLH | Helix-loop-helix DNA-binding domain | bHLH | 0.000000038 | 33 |
| TRINITY_DN60908_c0_g1 | PF00010 | HLH | Helix-loop-helix DNA-binding domain | bHLH | 0.000000043 | 32.8 |
| TRINITY_DN56792_c1_g4 | PF00010 | HLH | Helix-loop-helix DNA-binding domain | bHLH | 0.000000028 | 33.5 |
| TRINITY_DN52332_c1_g5 | PF00010 | HLH | Helix-loop-helix DNA-binding domain | bHLH | 0.000000001 | 38.1 |
| TRINITY_DN54873_c4_g1 | PF00010 | HLH | Helix-loop-helix DNA-binding domain | bHLH | 5.7E-10 | 38.9 |
| TRINITY_DN59676_c2_g2 | PF00010 | HLH | Helix-loop-helix DNA-binding domain | bHLH | 0.0000027 | 27.1 |
| TRINITY_DN66940_c0_g1 | PF00010 | HLH | Helix-loop-helix DNA-binding domain | bHLH | 0.000000001 | 38 |
| TRINITY_DN66940_c0_g2 | PF00010 | HLH | Helix-loop-helix DNA-binding domain | bHLH | 0.000013 | 24.9 |
| TRINITY_DN58576_c2_g2 | PF00010 | HLH | Helix-loop-helix DNA-binding domain | bHLH | 1.9E-10 | 40.4 |
| TRINITY_DN61349_c4_g3 | PF00010 | HLH | Helix-loop-helix DNA-binding domain | bHLH | 3.1E-10 | 39.7 |
| TRINITY_DN52033_c5_g1 | PF00010 | HLH | Helix-loop-helix DNA-binding domain | bHLH | 0.0000034 | 26.8 |
| TRINITY_DN64784_c0_g1 | PF00010 | HLH | Helix-loop-helix DNA-binding domain | bHLH | 0.00000047 | 29.5 |
| TRINITY_DN48751_c3_g1 | PF00010 | HLH | Helix-loop-helix DNA-binding domain | bHLH | 0.000000069 | 32.2 |
| TRINITY_DN48751_c3_g2 | PF00010 | HLH | Helix-loop-helix DNA-binding domain | bHLH | 0.000000033 | 33.3 |
| TRINITY_DN36993_c0_g1 | PF00010 | HLH | Helix-loop-helix DNA-binding domain | bHLH | 9.8E-14 | 50.9 |
| TRINITY_DN67837_c0_g5 | PF00010 | HLH | Helix-loop-helix DNA-binding domain | bHLH | 5.3E-10 | 39 |
| TRINITY_DN67837_c0_g1 | PF00010 | HLH | Helix-loop-helix DNA-binding domain | bHLH | 0.00000015 | 31.1 |
| TRINITY_DN82245_c0_g1 | PF00010 | HLH | Helix-loop-helix DNA-binding domain | bHLH | 7.3E-13 | 48.1 |
| TRINITY_DN44632_c0_g1 | PF00010 | HLH | Helix-loop-helix DNA-binding domain | bHLH | 1.5E-10 | 40.8 |
| TRINITY_DN24083_c0_g1 | PF00010 | HLH | Helix-loop-helix DNA-binding domain | bHLH | 1.7E-09 | 37.4 |
| TRINITY_DN60070_c0_g1 | PF00010 | HLH | Helix-loop-helix DNA-binding domain | bHLH | 2E-12 | 46.7 |
| TRINITY_DN60053_c0_g1 | PF00010 | HLH | Helix-loop-helix DNA-binding domain | bHLH | 6.4E-09 | 35.5 |
| TRINITY_DN42064_c0_g1 | PF00010 | HLH | Helix-loop-helix DNA-binding domain | bHLH | 0.0000015 | 27.9 |
| TRINITY_DN62824_c0_g1 | PF00010 | HLH | Helix-loop-helix DNA-binding domain | bHLH | 0.000011 | 25.1 |
| TRINITY_DN62824_c1_g2 | PF00010 | HLH | Helix-loop-helix DNA-binding domain | bHLH | 0.000092 | 22.2 |
| TRINITY_DN50282_c2_g7 | PF00010 | HLH | Helix-loop-helix DNA-binding domain | bHLH | 9.1E-09 | 35 |
| TRINITY_DN59460_c0_g3 | PF00010 | HLH | Helix-loop-helix DNA-binding domain | bHLH | 0.000000012 | 34.7 |
| TRINITY_DN54948_c0_g1 | PF00010 | HLH | Helix-loop-helix DNA-binding domain | bHLH | 2.3E-09 | 36.9 |
| TRINITY_DN60522_c1_g1 | PF00010 | HLH | Helix-loop-helix DNA-binding domain | bHLH | 1.9E-13 | 50 |
| TRINITY_DN69064_c0_g2 | PF00010 | HLH | Helix-loop-helix DNA-binding domain | bHLH | 0.000019 | 24.4 |
| TRINITY_DN72443_c1_g2 | PF00010 | HLH | Helix-loop-helix DNA-binding domain | bHLH | 0.000000058 | 32.4 |
| TRINITY_DN46621_c0_g1 | PF00010 | HLH | Helix-loop-helix DNA-binding domain | bHLH | 0.00000001 | 34.9 |
| TRINITY_DN67960_c0_g1 | PF00010 | HLH | Helix-loop-helix DNA-binding domain | bHLH | 0.000045 | 23.2 |
| TRINITY_DN72442_c0_g5 | PF00010 | HLH | Helix-loop-helix DNA-binding domain | bHLH | 0.0000049 | 26.3 |
| TRINITY_DN72442_c0_g2 | PF00010 | HLH | Helix-loop-helix DNA-binding domain | bHLH | 9E-10 | 38.2 |
| TRINITY_DN72442_c0_g1 | PF00010 | HLH | Helix-loop-helix DNA-binding domain | bHLH | 0.000096 | 22.1 |
| TRINITY_DN28203_c0_g1 | PF00010 | HLH | Helix-loop-helix DNA-binding domain | bHLH | 0.000000081 | 32 |
| TRINITY_DN60736_c4_g1 | PF00010 | HLH | Helix-loop-helix DNA-binding domain | bHLH | 0.0000011 | 28.3 |
| TRINITY_DN70320_c5_g6 | PF00010 | HLH | Helix-loop-helix DNA-binding domain | bHLH | 3.1E-15 | 55.7 |
| TRINITY_DN46760_c3_g1 | PF00010 | HLH | Helix-loop-helix DNA-binding domain | bHLH | 0.0000028 | 27 |
| TRINITY_DN44182_c0_g3 | PF00010 | HLH | Helix-loop-helix DNA-binding domain | bHLH | 0.00000072 | 28.9 |
| TRINITY_DN44182_c0_g1 | PF00010 | HLH | Helix-loop-helix DNA-binding domain | bHLH | 2.2E-09 | 37 |
| TRINITY_DN9482_c0_g1 | PF00010 | HLH | Helix-loop-helix DNA-binding domain | bHLH | 0.0000014 | 28 |
| TRINITY_DN67197_c0_g1 | PF00010 | HLH | Helix-loop-helix DNA-binding domain | bHLH | 0.00018 | 21.3 |
| TRINITY_DN43567_c0_g1 | PF00010 | HLH | Helix-loop-helix DNA-binding domain | bHLH | 0.00037 | 20.3 |
| TRINITY_DN57780_c2_g1 | PF00010 | HLH | Helix-loop-helix DNA-binding domain | bHLH | 6.8E-10 | 38.6 |
| TRINITY_DN75474_c2_g1 | PF00010 | HLH | Helix-loop-helix DNA-binding domain | bHLH | 0.0000048 | 26.3 |
| TRINITY_DN45963_c0_g1 | PF00010 | HLH | Helix-loop-helix DNA-binding domain | bHLH | 0.00015 | 21.5 |
| TRINITY_DN48444_c0_g1 | PF00010 | HLH | Helix-loop-helix DNA-binding domain | bHLH | 4.2E-09 | 36.1 |
| TRINITY_DN4851_c0_g1 | PF00010 | HLH | Helix-loop-helix DNA-binding domain | bHLH | 3.1E-11 | 42.9 |
| TRINITY_DN64612_c0_g1 | PF00010 | HLH | Helix-loop-helix DNA-binding domain | bHLH | 0.0000002 | 30.7 |
| TRINITY_DN55923_c0_g3 | PF00010 | HLH | Helix-loop-helix DNA-binding domain | bHLH | 0.0000051 | 26.2 |
| TRINITY_DN55923_c0_g2 | PF00010 | HLH | Helix-loop-helix DNA-binding domain | bHLH | 0.0000003 | 30.2 |
| TRINITY_DN55923_c0_g1 | PF00010 | HLH | Helix-loop-helix DNA-binding domain | bHLH | 0.00000094 | 28.6 |
| TRINITY_DN44964_c0_g1 | PF00010 | HLH | Helix-loop-helix DNA-binding domain | bHLH | 1.5E-15 | 56.7 |
| TRINITY_DN45791_c0_g1 | PF00010 | HLH | Helix-loop-helix DNA-binding domain | bHLH | 0.000000098 | 31.7 |
| TRINITY_DN45780_c0_g1 | PF00010 | HLH | Helix-loop-helix DNA-binding domain | bHLH | 0.00026 | 20.8 |
| TRINITY_DN47961_c0_g1 | PF00010 | HLH | Helix-loop-helix DNA-binding domain | bHLH | 0.00051 | 19.8 |
| TRINITY_DN61123_c0_g1 | PF00010 | HLH | Helix-loop-helix DNA-binding domain | bHLH | 1.3E-13 | 50.5 |
| TRINITY_DN7275_c0_g1 | PF00010 | HLH | Helix-loop-helix DNA-binding domain | bHLH | 2.3E-11 | 43.3 |
| TRINITY_DN46451_c5_g1 | PF00010 | HLH | Helix-loop-helix DNA-binding domain | bHLH | 1.8E-11 | 43.7 |
| TRINITY_DN64276_c0_g1 | PF00010 | HLH | Helix-loop-helix DNA-binding domain | bHLH | 1.3E-10 | 41 |
| TRINITY_DN59356_c0_g1 | PF00010 | HLH | Helix-loop-helix DNA-binding domain | bHLH | 0.000000072 | 32.1 |
| TRINITY_DN18041_c0_g1 | PF00010 | HLH | Helix-loop-helix DNA-binding domain | bHLH | 0.00007 | 22.6 |
| TRINITY_DN45001_c0_g1 | PF00010 | HLH | Helix-loop-helix DNA-binding domain | bHLH | 0.000025 | 24 |
| TRINITY_DN60299_c0_g1 | PF00010 | HLH | Helix-loop-helix DNA-binding domain | bHLH | 0.000076 | 22.5 |
| TRINITY_DN60299_c0_g2 | PF00010 | HLH | Helix-loop-helix DNA-binding domain | bHLH | 3.8E-14 | 52.2 |
| TRINITY_DN70417_c0_g1 | PF00010 | HLH | Helix-loop-helix DNA-binding domain | bHLH | 0.000011 | 25.1 |
| TRINITY_DN70417_c0_g2 | PF00010 | HLH | Helix-loop-helix DNA-binding domain | bHLH | 1.5E-09 | 37.5 |
| TRINITY_DN54605_c2_g6 | PF00010 | HLH | Helix-loop-helix DNA-binding domain | bHLH | 1.5E-09 | 37.6 |
| TRINITY_DN52006_c1_g1 | PF00010 | HLH | Helix-loop-helix DNA-binding domain | bHLH | 0.0000032 | 26.9 |
| TRINITY_DN57578_c0_g1 | PF00010 | HLH | Helix-loop-helix DNA-binding domain | bHLH | 5.4E-09 | 35.7 |
| TRINITY_DN41440_c0_g1 | PF00010 | HLH | Helix-loop-helix DNA-binding domain | bHLH | 0.0000042 | 26.5 |
| TRINITY_DN52314_c3_g1 | PF00010 | HLH | Helix-loop-helix DNA-binding domain | bHLH | 5.4E-09 | 35.8 |
| TRINITY_DN49505_c0_g1 | PF00010 | HLH | Helix-loop-helix DNA-binding domain | bHLH | 0.000000099 | 31.7 |
| TRINITY_DN69020_c3_g1 | PF00010 | HLH | Helix-loop-helix DNA-binding domain | bHLH | 1.4E-15 | 56.9 |
| TRINITY_DN64552_c3_g4 | PF03195 | DUF260 | Protein of unknown function DUF260 | LOB | 3.5E-39 | 133 |
| TRINITY_DN54835_c0_g1 | PF03195 | DUF260 | Protein of unknown function DUF260 | LOB | 5E-37 | 126.1 |
| TRINITY_DN54835_c0_g3 | PF03195 | DUF260 | Protein of unknown function DUF260 | LOB | 1.3E-37 | 128 |
| TRINITY_DN54835_c0_g6 | PF03195 | DUF260 | Protein of unknown function DUF260 | LOB | 6.9E-19 | 67.9 |
| TRINITY_DN66014_c0_g3 | PF03195 | DUF260 | Protein of unknown function DUF260 | LOB | 3.1E-22 | 78.7 |
| TRINITY_DN66014_c0_g2 | PF03195 | DUF260 | Protein of unknown function DUF260 | LOB | 3.6E-12 | 46.4 |
| TRINITY_DN66014_c0_g4 | PF03195 | DUF260 | Protein of unknown function DUF260 | LOB | 1.1E-16 | 60.9 |
| TRINITY_DN66014_c0_g1 | PF03195 | DUF260 | Protein of unknown function DUF260 | LOB | 6.1E-09 | 36.1 |
| TRINITY_DN1599_c0_g1 | PF03195 | DUF260 | Protein of unknown function DUF260 | LOB | 1E-38 | 131.6 |
| TRINITY_DN43449_c0_g1 | PF03195 | DUF260 | Protein of unknown function DUF260 | LOB | 1.8E-41 | 140.4 |
| TRINITY_DN36746_c0_g1 | PF03195 | DUF260 | Protein of unknown function DUF260 | LOB | 4E-40 | 136.1 |
| TRINITY_DN36746_c0_g2 | PF03195 | DUF260 | Protein of unknown function DUF260 | LOB | 1.5E-40 | 137.5 |
| TRINITY_DN64740_c0_g1 | PF03195 | DUF260 | Protein of unknown function DUF260 | LOB | 1.1E-22 | 80.2 |
| TRINITY_DN68445_c1_g4 | PF03195 | DUF260 | Protein of unknown function DUF260 | LOB | 4.7E-25 | 87.7 |
| TRINITY_DN35482_c0_g1 | PF03195 | DUF260 | Protein of unknown function DUF260 | LOB | 3.6E-42 | 142.6 |
| TRINITY_DN45431_c0_g1 | PF03195 | DUF260 | Protein of unknown function DUF260 | LOB | 5.2E-18 | 65.1 |
| TRINITY_DN51993_c3_g2 | PF03195 | DUF260 | Protein of unknown function DUF260 | LOB | 1.7E-39 | 134.1 |
| TRINITY_DN51993_c3_g3 | PF03195 | DUF260 | Protein of unknown function DUF260 | LOB | 3.6E-27 | 94.5 |
| TRINITY_DN51993_c3_g1 | PF03195 | DUF260 | Protein of unknown function DUF260 | LOB | 9.8E-44 | 147.7 |
| TRINITY_DN44146_c0_g1 | PF03195 | DUF260 | Protein of unknown function DUF260 | LOB | 3.3E-44 | 149.2 |
| TRINITY_DN82502_c0_g1 | PF03195 | DUF260 | Protein of unknown function DUF260 | LOB | 1.5E-44 | 150.3 |
| TRINITY_DN65929_c0_g2 | PF03195 | DUF260 | Protein of unknown function DUF260 | LOB | 1.2E-22 | 80 |
| TRINITY_DN65929_c0_g1 | PF03195 | DUF260 | Protein of unknown function DUF260 | LOB | 6.8E-19 | 68 |
| TRINITY_DN43128_c0_g1 | PF03195 | DUF260 | Protein of unknown function DUF260 | LOB | 2.8E-44 | 149.4 |
| TRINITY_DN79142_c0_g1 | PF03195 | DUF260 | Protein of unknown function DUF260 | LOB | 2.9E-10 | 40.3 |
| TRINITY_DN42801_c0_g1 | PF03195 | DUF260 | Protein of unknown function DUF260 | LOB | 1.1E-37 | 128.3 |
| TRINITY_DN23844_c0_g1 | PF03195 | DUF260 | Protein of unknown function DUF260 | LOB | 9.7E-31 | 106 |
| TRINITY_DN53998_c0_g3 | PF03195 | DUF260 | Protein of unknown function DUF260 | LOB | 7.8E-41 | 138.4 |
| TRINITY_DN43369_c0_g1 | PF03195 | DUF260 | Protein of unknown function DUF260 | LOB | 1E-38 | 131.5 |
| TRINITY_DN51125_c0_g1 | PF03195 | DUF260 | Protein of unknown function DUF260 | LOB | 4.6E-28 | 97.4 |
| TRINITY_DN44095_c0_g1 | PF03195 | DUF260 | Protein of unknown function DUF260 | LOB | 2.4E-35 | 120.7 |
| TRINITY_DN64527_c1_g2 | PF00096 | zf-C2H2 | Zinc finger, C2H2 type | C2H2 | 6E-21 | 73.5 |
| TRINITY_DN64527_c1_g3 | PF00096 | zf-C2H2 | Zinc finger, C2H2 type | C2H2 | 1.4E-15 | 56.7 |
| TRINITY_DN64531_c1_g1 | PF00096 | zf-C2H2 | Zinc finger, C2H2 type | C2H2 | 3.7E-19 | 67.9 |
| TRINITY_DN57584_c3_g2 | PF00096 | zf-C2H2 | Zinc finger, C2H2 type | C2H2 | 0.00023 | 21.3 |
| TRINITY_DN41188_c0_g1 | PF00096 | zf-C2H2 | Zinc finger, C2H2 type | C2H2 | 0.00000062 | 29.4 |
| TRINITY_DN83029_c0_g1 | PF00096 | zf-C2H2 | Zinc finger, C2H2 type | C2H2 | 0.00096 | 19.4 |
| TRINITY_DN72802_c2_g2 | PF00096 | zf-C2H2 | Zinc finger, C2H2 type | C2H2 | 0.0002 | 21.5 |
| TRINITY_DN72802_c2_g1 | PF00096 | zf-C2H2 | Zinc finger, C2H2 type | C2H2 | 0.0002 | 21.5 |
| TRINITY_DN72802_c3_g1 | PF00096 | zf-C2H2 | Zinc finger, C2H2 type | C2H2 | 0.00036 | 20.7 |
| TRINITY_DN80050_c0_g1 | PF00096 | zf-C2H2 | Zinc finger, C2H2 type | C2H2 | 0.0000099 | 25.6 |
| TRINITY_DN71919_c0_g6 | PF00096 | zf-C2H2 | Zinc finger, C2H2 type | C2H2 | 0.00000086 | 28.9 |
| TRINITY_DN56430_c1_g4 | PF00096 | zf-C2H2 | Zinc finger, C2H2 type | C2H2 | 0.0000021 | 27.8 |
| TRINITY_DN58264_c0_g1 | PF00096 | zf-C2H2 | Zinc finger, C2H2 type | C2H2 | 1.2E-10 | 41.1 |
| TRINITY_DN70269_c1_g1 | PF00096 | zf-C2H2 | Zinc finger, C2H2 type | C2H2 | 0.00046 | 20.4 |
| TRINITY_DN14409_c0_g1 | PF00096 | zf-C2H2 | Zinc finger, C2H2 type | C2H2 | 1.5E-12 | 47.1 |
| TRINITY_DN67824_c1_g5 | PF00096 | zf-C2H2 | Zinc finger, C2H2 type | C2H2 | 0.0000018 | 27.9 |
| TRINITY_DN41655_c0_g1 | PF00096 | zf-C2H2 | Zinc finger, C2H2 type | C2H2 | 0.0004 | 20.6 |
| TRINITY_DN75931_c1_g1 | PF00096 | zf-C2H2 | Zinc finger, C2H2 type | C2H2 | 0.000008 | 25.9 |
| TRINITY_DN43892_c0_g1 | PF00096 | zf-C2H2 | Zinc finger, C2H2 type | C2H2 | 0.0000031 | 27.2 |
| TRINITY_DN79473_c0_g1 | PF00096 | zf-C2H2 | Zinc finger, C2H2 type | C2H2 | 9.8E-13 | 47.7 |
| TRINITY_DN72786_c1_g1 | PF00096 | zf-C2H2 | Zinc finger, C2H2 type | C2H2 | 0.00025 | 21.2 |
| TRINITY_DN66228_c0_g3 | PF00096 | zf-C2H2 | Zinc finger, C2H2 type | C2H2 | 0.000049 | 23.4 |
| TRINITY_DN66228_c0_g1 | PF00096 | zf-C2H2 | Zinc finger, C2H2 type | C2H2 | 2.2E-16 | 59.1 |
| TRINITY_DN66228_c0_g4 | PF00096 | zf-C2H2 | Zinc finger, C2H2 type | C2H2 | 0.000052 | 23.4 |
| TRINITY_DN58080_c1_g5 | PF00096 | zf-C2H2 | Zinc finger, C2H2 type | C2H2 | 7.2E-09 | 35.5 |
| TRINITY_DN53708_c1_g2 | PF00096 | zf-C2H2 | Zinc finger, C2H2 type | C2H2 | 2.5E-14 | 52.7 |
| TRINITY_DN44349_c0_g1 | PF00096 | zf-C2H2 | Zinc finger, C2H2 type | C2H2 | 0.0000017 | 28 |
| TRINITY_DN15686_c0_g1 | PF00096 | zf-C2H2 | Zinc finger, C2H2 type | C2H2 | 2.4E-09 | 37 |
| TRINITY_DN53211_c3_g1 | PF00096 | zf-C2H2 | Zinc finger, C2H2 type | C2H2 | 0.00006 | 23.1 |
| TRINITY_DN53211_c3_g5 | PF00096 | zf-C2H2 | Zinc finger, C2H2 type | C2H2 | 0.00000033 | 30.3 |
| TRINITY_DN55453_c1_g2 | PF00096 | zf-C2H2 | Zinc finger, C2H2 type | C2H2 | 0.000093 | 22.5 |
| TRINITY_DN59829_c1_g4 | PF00096 | zf-C2H2 | Zinc finger, C2H2 type | C2H2 | 0.00075 | 19.7 |
| TRINITY_DN71180_c0_g1 | PF00096 | zf-C2H2 | Zinc finger, C2H2 type | C2H2 | 0.000079 | 22.8 |
| TRINITY_DN44899_c0_g1 | PF00096 | zf-C2H2 | Zinc finger, C2H2 type | C2H2 | 0.00083 | 19.6 |
| TRINITY_DN44899_c0_g2 | PF00096 | zf-C2H2 | Zinc finger, C2H2 type | C2H2 | 0.000076 | 22.8 |
| TRINITY_DN44899_c0_g3 | PF00096 | zf-C2H2 | Zinc finger, C2H2 type | C2H2 | 0.00002 | 24.7 |
| TRINITY_DN44899_c0_g4 | PF00096 | zf-C2H2 | Zinc finger, C2H2 type | C2H2 | 0.0005 | 20.2 |
| TRINITY_DN75176_c1_g1 | PF00096 | zf-C2H2 | Zinc finger, C2H2 type | C2H2 | 0.00094 | 19.4 |
| TRINITY_DN75176_c1_g1 | PF00096 | zf-C2H2 | Zinc finger, C2H2 type | C2H2 | 0.0000078 | 25.9 |
| TRINITY_DN82956_c0_g1 | PF00096 | zf-C2H2 | Zinc finger, C2H2 type | C2H2 | 0.000000017 | 34.3 |
| TRINITY_DN74764_c0_g1 | PF00096 | zf-C2H2 | Zinc finger, C2H2 type | C2H2 | 0.000011 | 25.5 |
| TRINITY_DN81219_c0_g1 | PF00096 | zf-C2H2 | Zinc finger, C2H2 type | C2H2 | 3.5E-18 | 64.8 |
| TRINITY_DN51333_c0_g4 | PF00096 | zf-C2H2 | Zinc finger, C2H2 type | C2H2 | 0.000077 | 22.8 |
| TRINITY_DN51333_c0_g5 | PF00096 | zf-C2H2 | Zinc finger, C2H2 type | C2H2 | 0.00048 | 20.3 |
| TRINITY_DN51333_c0_g6 | PF00096 | zf-C2H2 | Zinc finger, C2H2 type | C2H2 | 0.00079 | 19.6 |
| TRINITY_DN51333_c0_g8 | PF00096 | zf-C2H2 | Zinc finger, C2H2 type | C2H2 | 0.00022 | 21.4 |
| TRINITY_DN56430_c1_g1 | PF00096 | zf-C2H2 | Zinc finger, C2H2 type | C2H2 | 0.000022 | 24.5 |
| TRINITY_DN56430_c1_g3 | PF00096 | zf-C2H2 | Zinc finger, C2H2 type | C2H2 | 0.0000012 | 28.5 |
| TRINITY_DN73900_c0_g2 | PF00096 | zf-C2H2 | Zinc finger, C2H2 type | C2H2 | 0.0000039 | 26.9 |
| TRINITY_DN72355_c1_g4 | PF02042 | RWP-RK | RWP-RK domain | Nin-like | 3.2E-14 | 52.5 |
| TRINITY_DN61268_c0_g2 | PF02042 | RWP-RK | RWP-RK domain | Nin-like | 1.5E-11 | 43.9 |
| TRINITY_DN44718_c0_g1 | PF02042 | RWP-RK | RWP-RK domain | Nin-like | 2.1E-18 | 65.9 |
| TRINITY_DN56628_c0_g1 | PF02042 | RWP-RK | RWP-RK domain | Nin-like | 6.4E-20 | 70.7 |
| TRINITY_DN47854_c4_g10 | PF02042 | RWP-RK | RWP-RK domain | Nin-like | 1.6E-26 | 91.9 |
| TRINITY_DN74104_c3_g1 | PF02042 | RWP-RK | RWP-RK domain | Nin-like | 4.2E-26 | 90.5 |
| TRINITY_DN56987_c2_g1 | PF03101 | FAR1 | FAR1 DNA-binding domain | FAR1 | 9.3E-39 | 131.9 |
| TRINITY_DN72450_c1_g6 | PF03101 | FAR1 | FAR1 DNA-binding domain | FAR1 | 1.6E-21 | 76.7 |
| TRINITY_DN72450_c1_g1 | PF03101 | FAR1 | FAR1 DNA-binding domain | FAR1 | 3.2E-10 | 40.5 |
| TRINITY_DN67665_c3_g3 | PF03101 | FAR1 | FAR1 DNA-binding domain | FAR1 | 4.9E-49 | 164.9 |
| TRINITY_DN74508_c0_g4 | PF03101 | FAR1 | FAR1 DNA-binding domain | FAR1 | 5.3E-23 | 81.4 |
| TRINITY_DN74508_c1_g2 | PF03101 | FAR1 | FAR1 DNA-binding domain | FAR1 | 1.4E-49 | 166.6 |
| TRINITY_DN72304_c1_g3 | PF03101 | FAR1 | FAR1 DNA-binding domain | FAR1 | 3.2E-36 | 123.8 |
| TRINITY_DN54160_c4_g1 | PF03101 | FAR1 | FAR1 DNA-binding domain | FAR1 | 0.0000028 | 27.8 |
| TRINITY_DN54160_c5_g2 | PF03101 | FAR1 | FAR1 DNA-binding domain | FAR1 | 0.00000028 | 31 |
| TRINITY_DN69603_c0_g1 | PF03101 | FAR1 | FAR1 DNA-binding domain | FAR1 | 0.00000056 | 30.1 |
| TRINITY_DN56526_c1_g3 | PF03101 | FAR1 | FAR1 DNA-binding domain | FAR1 | 3.7E-15 | 56.3 |
| TRINITY_DN56526_c1_g1 | PF03101 | FAR1 | FAR1 DNA-binding domain | FAR1 | 1.7E-13 | 51 |
| TRINITY_DN41348_c0_g2 | PF03101 | FAR1 | FAR1 DNA-binding domain | FAR1 | 8.7E-09 | 35.9 |
| TRINITY_DN41348_c0_g3 | PF03101 | FAR1 | FAR1 DNA-binding domain | FAR1 | 0.00039 | 20.9 |
| TRINITY_DN72299_c3_g3 | PF03101 | FAR1 | FAR1 DNA-binding domain | FAR1 | 3.9E-47 | 158.8 |
| TRINITY_DN65034_c4_g1 | PF03101 | FAR1 | FAR1 DNA-binding domain | FAR1 | 1.2E-17 | 64.3 |
| TRINITY_DN71928_c0_g1 | PF03101 | FAR1 | FAR1 DNA-binding domain | FAR1 | 0.00002 | 25.1 |
| TRINITY_DN53713_c0_g2 | PF03101 | FAR1 | FAR1 DNA-binding domain | FAR1 | 2.8E-18 | 66.3 |
| TRINITY_DN65471_c0_g1 | PF03101 | FAR1 | FAR1 DNA-binding domain | FAR1 | 7.5E-16 | 58.5 |
| TRINITY_DN59473_c3_g2 | PF03101 | FAR1 | FAR1 DNA-binding domain | FAR1 | 4.4E-40 | 136.2 |
| TRINITY_DN46675_c1_g1 | PF03101 | FAR1 | FAR1 DNA-binding domain | FAR1 | 1.5E-15 | 57.6 |
| TRINITY_DN46675_c1_g2 | PF03101 | FAR1 | FAR1 DNA-binding domain | FAR1 | 0.0000025 | 28 |
| TRINITY_DN44301_c0_g1 | PF03101 | FAR1 | FAR1 DNA-binding domain | FAR1 | 9.5E-22 | 77.4 |
| TRINITY_DN51683_c1_g1 | PF03101 | FAR1 | FAR1 DNA-binding domain | FAR1 | 1E-17 | 64.5 |
| TRINITY_DN67221_c0_g2 | PF03101 | FAR1 | FAR1 DNA-binding domain | FAR1 | 3.3E-17 | 62.8 |
| TRINITY_DN67221_c0_g6 | PF03101 | FAR1 | FAR1 DNA-binding domain | FAR1 | 5.8E-21 | 74.9 |
| TRINITY_DN67221_c0_g7 | PF03101 | FAR1 | FAR1 DNA-binding domain | FAR1 | 2.6E-18 | 66.4 |
| TRINITY_DN67221_c0_g11 | PF03101 | FAR1 | FAR1 DNA-binding domain | FAR1 | 2.7E-18 | 66.3 |
| TRINITY_DN67221_c0_g3 | PF03101 | FAR1 | FAR1 DNA-binding domain | FAR1 | 2.2E-17 | 63.4 |
| TRINITY_DN67221_c0_g12 | PF03101 | FAR1 | FAR1 DNA-binding domain | FAR1 | 2.2E-16 | 60.2 |
| TRINITY_DN65732_c1_g5 | PF03101 | FAR1 | FAR1 DNA-binding domain | FAR1 | 1.6E-15 | 57.4 |
| TRINITY_DN40189_c0_g1 | PF03101 | FAR1 | FAR1 DNA-binding domain | FAR1 | 1.7E-20 | 73.4 |
| TRINITY_DN65663_c5_g1 | PF03101 | FAR1 | FAR1 DNA-binding domain | FAR1 | 8.8E-21 | 74.3 |
| TRINITY_DN65663_c6_g1 | PF03101 | FAR1 | FAR1 DNA-binding domain | FAR1 | 6.7E-22 | 77.9 |
| TRINITY_DN75832_c2_g3 | PF03101 | FAR1 | FAR1 DNA-binding domain | FAR1 | 9.6E-21 | 74.2 |
| TRINITY_DN53142_c2_g1 | PF03101 | FAR1 | FAR1 DNA-binding domain | FAR1 | 5.5E-31 | 107 |
| TRINITY_DN52768_c0_g1 | PF03101 | FAR1 | FAR1 DNA-binding domain | FAR1 | 8.5E-20 | 71.2 |
| TRINITY_DN53195_c0_g2 | PF03101 | FAR1 | FAR1 DNA-binding domain | FAR1 | 8.7E-41 | 138.4 |
| TRINITY_DN49855_c0_g2 | PF03101 | FAR1 | FAR1 DNA-binding domain | FAR1 | 0.00000016 | 31.8 |
| TRINITY_DN61426_c0_g1 | PF03101 | FAR1 | FAR1 DNA-binding domain | FAR1 | 5.9E-22 | 78.1 |
| TRINITY_DN57411_c0_g1 | PF03101 | FAR1 | FAR1 DNA-binding domain | FAR1 | 9.7E-42 | 141.5 |
| TRINITY_DN53874_c2_g5 | PF03101 | FAR1 | FAR1 DNA-binding domain | FAR1 | 9.2E-82 | 269.8 |
| TRINITY_DN46232_c0_g1 | PF03101 | FAR1 | FAR1 DNA-binding domain | FAR1 | 0.00006 | 23.6 |
| TRINITY_DN76366_c3_g2 | PF03101 | FAR1 | FAR1 DNA-binding domain | FAR1 | 0.00002 | 25.1 |
| TRINITY_DN52803_c1_g1 | PF03101 | FAR1 | FAR1 DNA-binding domain | FAR1 | 1.5E-23 | 83.2 |
| TRINITY_DN52803_c1_g5 | PF03101 | FAR1 | FAR1 DNA-binding domain | FAR1 | 0.0006 | 20.4 |
| TRINITY_DN60136_c2_g2 | PF03101 | FAR1 | FAR1 DNA-binding domain | FAR1 | 1.7E-15 | 57.3 |
| TRINITY_DN63986_c0_g1 | PF00847 | AP2 | AP2 domain | AP2/ERF | 2.6E-19 | 69.1 |
| TRINITY_DN76406_c2_g3 | PF00847 | AP2 | AP2 domain | AP2/ERF | 0.00011 | 22.3 |
| TRINITY_DN9821_c0_g1 | PF00847 | AP2 | AP2 domain | AP2/ERF | 1.3E-14 | 54.1 |
| TRINITY_DN60153_c2_g1 | PF00847 | AP2 | AP2 domain | AP2/ERF | 1.4E-11 | 44.4 |
| TRINITY_DN60153_c2_g3 | PF00847 | AP2 | AP2 domain | AP2/ERF | 0.00056 | 20 |
| TRINITY_DN60153_c2_g2 | PF00847 | AP2 | AP2 domain | AP2/ERF | 3.2E-11 | 43.2 |
| TRINITY_DN48538_c1_g1 | PF00847 | AP2 | AP2 domain | AP2/ERF | 2.2E-13 | 50.1 |
| TRINITY_DN67510_c1_g4 | PF00847 | AP2 | AP2 domain | AP2/ERF | 1E-10 | 41.6 |
| TRINITY_DN67510_c1_g1 | PF00847 | AP2 | AP2 domain | AP2/ERF | 3.2E-11 | 43.2 |
| TRINITY_DN91546_c0_g1 | PF00847 | AP2 | AP2 domain | AP2/ERF | 1.1E-12 | 47.8 |
| TRINITY_DN59892_c0_g4 | PF00847 | AP2 | AP2 domain | AP2/ERF | 4E-13 | 49.3 |
| TRINITY_DN59892_c0_g5 | PF00847 | AP2 | AP2 domain | AP2/ERF | 2.5E-14 | 53.1 |
| TRINITY_DN59892_c0_g6 | PF00847 | AP2 | AP2 domain | AP2/ERF | 6.9E-12 | 45.3 |
| TRINITY_DN59892_c0_g7 | PF00847 | AP2 | AP2 domain | AP2/ERF | 7E-13 | 48.5 |
| TRINITY_DN59892_c0_g1 | PF00847 | AP2 | AP2 domain | AP2/ERF | 0.0000091 | 25.7 |
| TRINITY_DN59892_c0_g2 | PF00847 | AP2 | AP2 domain | AP2/ERF | 8.4E-13 | 48.2 |
| TRINITY_DN59892_c0_g3 | PF00847 | AP2 | AP2 domain | AP2/ERF | 1.2E-12 | 47.8 |
| TRINITY_DN59892_c0_g8 | PF00847 | AP2 | AP2 domain | AP2/ERF | 3E-13 | 49.7 |
| TRINITY_DN59892_c0_g9 | PF00847 | AP2 | AP2 domain | AP2/ERF | 2.8E-13 | 49.7 |
| TRINITY_DN59892_c0_g10 | PF00847 | AP2 | AP2 domain | AP2/ERF | 4.3E-13 | 49.2 |
| TRINITY_DN56909_c2_g2 | PF00847 | AP2 | AP2 domain | AP2/ERF | 2E-12 | 47 |
| TRINITY_DN56909_c2_g1 | PF00847 | AP2 | AP2 domain | AP2/ERF | 4.9E-10 | 39.4 |
| TRINITY_DN45861_c0_g1 | PF00847 | AP2 | AP2 domain | AP2/ERF | 7.9E-13 | 48.3 |
| TRINITY_DN45861_c0_g3 | PF00847 | AP2 | AP2 domain | AP2/ERF | 2.9E-13 | 49.7 |
| TRINITY_DN45861_c0_g4 | PF00847 | AP2 | AP2 domain | AP2/ERF | 1.6E-13 | 50.6 |
| TRINITY_DN72316_c5_g2 | PF00847 | AP2 | AP2 domain | AP2/ERF | 1.1E-18 | 67.1 |
| TRINITY_DN72316_c5_g5 | PF00847 | AP2 | AP2 domain | AP2/ERF | 7.1E-16 | 58.1 |
| TRINITY_DN65606_c0_g2 | PF00847 | AP2 | AP2 domain | AP2/ERF | 0.00016 | 21.8 |
| TRINITY_DN56009_c2_g2 | PF00847 | AP2 | AP2 domain | AP2/ERF | 0.00024 | 21.1 |
| TRINITY_DN56009_c2_g2 | PF00847 | AP2 | AP2 domain | AP2/ERF | 0.00000038 | 30.1 |
| TRINITY_DN51774_c3_g2 | PF00847 | AP2 | AP2 domain | AP2/ERF | 7.9E-13 | 48.3 |
| TRINITY_DN66553_c4_g5 | PF00847 | AP2 | AP2 domain | AP2/ERF | 3.2E-12 | 46.4 |
| TRINITY_DN41378_c0_g2 | PF00847 | AP2 | AP2 domain | AP2/ERF | 5.3E-13 | 48.9 |
| TRINITY_DN40300_c0_g2 | PF00847 | AP2 | AP2 domain | AP2/ERF | 1.3E-12 | 47.6 |
| TRINITY_DN63390_c3_g1 | PF00847 | AP2 | AP2 domain | AP2/ERF | 0.000026 | 24.2 |
| TRINITY_DN45432_c0_g3 | PF00847 | AP2 | AP2 domain | AP2/ERF | 1.4E-13 | 50.7 |
| TRINITY_DN45432_c0_g4 | PF00847 | AP2 | AP2 domain | AP2/ERF | 1E-13 | 51.1 |
| TRINITY_DN45432_c0_g6 | PF00847 | AP2 | AP2 domain | AP2/ERF | 1.1E-11 | 44.7 |
| TRINITY_DN45432_c0_g5 | PF00847 | AP2 | AP2 domain | AP2/ERF | 6.7E-14 | 51.8 |
| TRINITY_DN71911_c7_g3 | PF00847 | AP2 | AP2 domain | AP2/ERF | 3E-11 | 43.3 |
| TRINITY_DN50138_c1_g6 | PF00847 | AP2 | AP2 domain | AP2/ERF | 2.6E-13 | 49.9 |
| TRINITY_DN45274_c0_g1 | PF00847 | AP2 | AP2 domain | AP2/ERF | 4.3E-11 | 42.8 |
| TRINITY_DN73481_c4_g10 | PF00847 | AP2 | AP2 domain | AP2/ERF | 1.3E-14 | 54.1 |
| TRINITY_DN44690_c0_g1 | PF00847 | AP2 | AP2 domain | AP2/ERF | 1E-13 | 51.1 |
| TRINITY_DN50057_c0_g3 | PF00847 | AP2 | AP2 domain | AP2/ERF | 0.0000059 | 26.3 |
| TRINITY_DN67854_c0_g2 | PF00847 | AP2 | AP2 domain | AP2/ERF | 0.00019 | 21.5 |
| TRINITY_DN45432_c0_g1 | PF00847 | AP2 | AP2 domain | AP2/ERF | 4.8E-12 | 45.8 |
| TRINITY_DN45432_c0_g2 | PF00847 | AP2 | AP2 domain | AP2/ERF | 2.6E-14 | 53 |
| TRINITY_DN69538_c0_g3 | PF00847 | AP2 | AP2 domain | AP2/ERF | 3.7E-13 | 49.4 |
| TRINITY_DN69538_c0_g5 | PF00847 | AP2 | AP2 domain | AP2/ERF | 2E-11 | 43.8 |
| TRINITY_DN69538_c0_g6 | PF00847 | AP2 | AP2 domain | AP2/ERF | 3.1E-13 | 49.6 |
| TRINITY_DN69538_c0_g4 | PF00847 | AP2 | AP2 domain | AP2/ERF | 2.6E-13 | 49.9 |
| TRINITY_DN69538_c0_g7 | PF00847 | AP2 | AP2 domain | AP2/ERF | 0.00000021 | 30.9 |
| TRINITY_DN88614_c0_g1 | PF00847 | AP2 | AP2 domain | AP2/ERF | 2.5E-12 | 46.7 |
| TRINITY_DN73580_c1_g1 | PF00847 | AP2 | AP2 domain | AP2/ERF | 1.3E-13 | 50.9 |
| TRINITY_DN18316_c0_g1 | PF00847 | AP2 | AP2 domain | AP2/ERF | 2.3E-11 | 43.6 |
| TRINITY_DN61823_c0_g1 | PF00847 | AP2 | AP2 domain | AP2/ERF | 8.7E-13 | 48.2 |
| TRINITY_DN61823_c1_g1 | PF00847 | AP2 | AP2 domain | AP2/ERF | 0.000000074 | 32.4 |
| TRINITY_DN61823_c1_g3 | PF00847 | AP2 | AP2 domain | AP2/ERF | 9E-13 | 48.1 |
| TRINITY_DN60979_c0_g1 | PF00847 | AP2 | AP2 domain | AP2/ERF | 1.2E-14 | 54.1 |
| TRINITY_DN45813_c0_g1 | PF00847 | AP2 | AP2 domain | AP2/ERF | 1.5E-12 | 47.4 |
| TRINITY_DN64528_c0_g1 | PF00847 | AP2 | AP2 domain | AP2/ERF | 3.4E-14 | 52.7 |
| TRINITY_DN64528_c0_g2 | PF00847 | AP2 | AP2 domain | AP2/ERF | 3E-13 | 49.7 |
| TRINITY_DN70717_c1_g2 | PF00847 | AP2 | AP2 domain | AP2/ERF | 1.8E-14 | 53.6 |
| TRINITY_DN70717_c1_g1 | PF00847 | AP2 | AP2 domain | AP2/ERF | 0.000058 | 23.1 |
| TRINITY_DN63073_c0_g2 | PF00847 | AP2 | AP2 domain | AP2/ERF | 2.9E-12 | 46.5 |
| TRINITY_DN36237_c0_g1 | PF00847 | AP2 | AP2 domain | AP2/ERF | 3.4E-12 | 46.3 |
| TRINITY_DN57395_c1_g5 | PF00847 | AP2 | AP2 domain | AP2/ERF | 0.000000093 | 32.1 |
| TRINITY_DN60442_c0_g1 | PF00847 | AP2 | AP2 domain | AP2/ERF | 1.7E-10 | 40.9 |
| TRINITY_DN41860_c0_g1 | PF00847 | AP2 | AP2 domain | AP2/ERF | 6E-15 | 55.1 |
| TRINITY_DN51950_c0_g1 | PF00847 | AP2 | AP2 domain | AP2/ERF | 3.1E-14 | 52.8 |
| TRINITY_DN42907_c0_g1 | PF00847 | AP2 | AP2 domain | AP2/ERF | 5.1E-10 | 39.3 |
| TRINITY_DN42907_c0_g2 | PF00847 | AP2 | AP2 domain | AP2/ERF | 4.7E-10 | 39.4 |
| TRINITY_DN9335_c0_g1 | PF00847 | AP2 | AP2 domain | AP2/ERF | 2E-14 | 53.4 |
| TRINITY_DN76945_c0_g1 | PF00847 | AP2 | AP2 domain | AP2/ERF | 3.8E-13 | 49.3 |
| TRINITY_DN10831_c0_g1 | PF00847 | AP2 | AP2 domain | AP2/ERF | 1.7E-11 | 44 |
| TRINITY_DN66257_c3_g2 | PF00847 | AP2 | AP2 domain | AP2/ERF | 6.4E-11 | 42.2 |
| TRINITY_DN66257_c3_g3 | PF00847 | AP2 | AP2 domain | AP2/ERF | 0.00015 | 21.8 |
| TRINITY_DN69913_c1_g6 | PF00847 | AP2 | AP2 domain | AP2/ERF | 7E-13 | 48.5 |
| TRINITY_DN28872_c0_g1 | PF00847 | AP2 | AP2 domain | AP2/ERF | 1.4E-09 | 37.9 |
| TRINITY_DN62080_c0_g1 | PF00847 | AP2 | AP2 domain | AP2/ERF | 1.2E-13 | 51 |
| TRINITY_DN62080_c0_g3 | PF00847 | AP2 | AP2 domain | AP2/ERF | 8E-13 | 48.3 |
| TRINITY_DN62080_c0_g5 | PF00847 | AP2 | AP2 domain | AP2/ERF | 2E-12 | 47 |
| TRINITY_DN42857_c0_g1 | PF00847 | AP2 | AP2 domain | AP2/ERF | 6.2E-11 | 42.3 |
| TRINITY_DN39153_c0_g1 | PF00847 | AP2 | AP2 domain | AP2/ERF | 5.6E-12 | 45.6 |
| TRINITY_DN67040_c3_g1 | PF00847 | AP2 | AP2 domain | AP2/ERF | 0.00000055 | 29.6 |
| TRINITY_DN67040_c3_g1 | PF00847 | AP2 | AP2 domain | AP2/ERF | 2.8E-12 | 46.6 |
| TRINITY_DN50492_c2_g2 | PF00847 | AP2 | AP2 domain | AP2/ERF | 2.6E-11 | 43.4 |
| TRINITY_DN25392_c0_g1 | PF00847 | AP2 | AP2 domain | AP2/ERF | 2.3E-11 | 43.7 |
| TRINITY_DN79095_c0_g1 | PF00847 | AP2 | AP2 domain | AP2/ERF | 6.8E-14 | 51.7 |
| TRINITY_DN44250_c0_g1 | PF00847 | AP2 | AP2 domain | AP2/ERF | 4.4E-11 | 42.7 |
| TRINITY_DN28025_c0_g1 | PF00847 | AP2 | AP2 domain | AP2/ERF | 3.3E-12 | 46.3 |
| TRINITY_DN91387_c0_g1 | PF00847 | AP2 | AP2 domain | AP2/ERF | 7.5E-14 | 51.6 |
| TRINITY_DN65897_c0_g1 | PF00847 | AP2 | AP2 domain | AP2/ERF | 1.8E-13 | 50.4 |
| TRINITY_DN65897_c1_g7 | PF00847 | AP2 | AP2 domain | AP2/ERF | 2.8E-12 | 46.5 |
| TRINITY_DN50826_c0_g1 | PF00847 | AP2 | AP2 domain | AP2/ERF | 0.000000001 | 38.4 |
| TRINITY_DN51872_c3_g2 | PF00847 | AP2 | AP2 domain | AP2/ERF | 1.1E-12 | 47.8 |
| TRINITY_DN39332_c0_g3 | PF00847 | AP2 | AP2 domain | AP2/ERF | 0.00000014 | 31.5 |
| TRINITY_DN47361_c1_g2 | PF00847 | AP2 | AP2 domain | AP2/ERF | 0.0000015 | 28.2 |
| TRINITY_DN47361_c1_g6 | PF00847 | AP2 | AP2 domain | AP2/ERF | 3.1E-12 | 46.4 |
| TRINITY_DN47361_c1_g1 | PF00847 | AP2 | AP2 domain | AP2/ERF | 7.6E-13 | 48.4 |
| TRINITY_DN48622_c0_g1 | PF00847 | AP2 | AP2 domain | AP2/ERF | 3.1E-13 | 49.6 |
| TRINITY_DN48622_c1_g3 | PF00847 | AP2 | AP2 domain | AP2/ERF | 0.00000019 | 31.1 |
| TRINITY_DN48622_c1_g2 | PF00847 | AP2 | AP2 domain | AP2/ERF | 2.9E-12 | 46.5 |
| TRINITY_DN48622_c1_g6 | PF00847 | AP2 | AP2 domain | AP2/ERF | 0.00029 | 20.9 |
| TRINITY_DN59227_c1_g3 | PF00847 | AP2 | AP2 domain | AP2/ERF | 1.3E-10 | 41.3 |
| TRINITY_DN44064_c1_g1 | PF00847 | AP2 | AP2 domain | AP2/ERF | 6.4E-13 | 48.6 |
| TRINITY_DN46856_c0_g2 | PF00847 | AP2 | AP2 domain | AP2/ERF | 1.9E-23 | 82.3 |
| TRINITY_DN49553_c1_g2 | PF00847 | AP2 | AP2 domain | AP2/ERF | 1.1E-11 | 44.6 |
| TRINITY_DN36381_c0_g1 | PF00847 | AP2 | AP2 domain | AP2/ERF | 4.6E-10 | 39.5 |
| TRINITY_DN41773_c0_g1 | PF00847 | AP2 | AP2 domain | AP2/ERF | 4.1E-13 | 49.2 |
| TRINITY_DN56909_c2_g3 | PF00847 | AP2 | AP2 domain | AP2/ERF | 2.6E-16 | 59.5 |
| TRINITY_DN56909_c2_g4 | PF00847 | AP2 | AP2 domain | AP2/ERF | 2.5E-16 | 59.5 |
| TRINITY_DN48895_c4_g1 | PF00642 | Zf-CCCH | Zinc finger C-x8-C-x5-C-x3-H type (and similar) | C3H | 7.2E-22 | 76.6 |
| TRINITY_DN48895_c4_g5 | PF00642 | Zf-CCCH | Zinc finger C-x8-C-x5-C-x3-H type (and similar) | C3H | 9.3E-09 | 34.9 |
| TRINITY_DN39769_c0_g1 | PF00642 | Zf-CCCH | Zinc finger C-x8-C-x5-C-x3-H type (and similar) | C3H | 1.9E-14 | 53 |
| TRINITY_DN52387_c8_g1 | PF00642 | Zf-CCCH | Zinc finger C-x8-C-x5-C-x3-H type (and similar) | C3H | 1.2E-46 | 155.7 |
| TRINITY_DN60976_c0_g2 | PF00642 | Zf-CCCH | Zinc finger C-x8-C-x5-C-x3-H type (and similar) | C3H | 0.00068 | 19.3 |
| TRINITY_DN38127_c0_g1 | PF00642 | Zf-CCCH | Zinc finger C-x8-C-x5-C-x3-H type (and similar) | C3H | 0.000000083 | 31.8 |
| TRINITY_DN67436_c3_g2 | PF00642 | Zf-CCCH | Zinc finger C-x8-C-x5-C-x3-H type (and similar) | C3H | 9.5E-18 | 63.5 |
| TRINITY_DN67436_c3_g4 | PF00642 | Zf-CCCH | Zinc finger C-x8-C-x5-C-x3-H type (and similar) | C3H | 1.6E-39 | 132.9 |
| TRINITY_DN72304_c1_g5 | PF00642 | Zf-CCCH | Zinc finger C-x8-C-x5-C-x3-H type (and similar) | C3H | 1.4E-09 | 37.5 |
| TRINITY_DN54084_c0_g1 | PF00642 | Zf-CCCH | Zinc finger C-x8-C-x5-C-x3-H type (and similar) | C3H | 0.000018 | 24.4 |
| TRINITY_DN71569_c1_g1 | PF00642 | Zf-CCCH | Zinc finger C-x8-C-x5-C-x3-H type (and similar) | C3H | 0.0000021 | 27.3 |
| TRINITY_DN48636_c0_g2 | PF00642 | Zf-CCCH | Zinc finger C-x8-C-x5-C-x3-H type (and similar) | C3H | 0.000086 | 22.2 |
| TRINITY_DN69637_c4_g1 | PF00642 | Zf-CCCH | Zinc finger C-x8-C-x5-C-x3-H type (and similar) | C3H | 0.00017 | 21.3 |
| TRINITY_DN46575_c0_g1 | PF00642 | Zf-CCCH | Zinc finger C-x8-C-x5-C-x3-H type (and similar) | C3H | 8.6E-17 | 60.5 |
| TRINITY_DN61301_c0_g2 | PF00642 | Zf-CCCH | Zinc finger C-x8-C-x5-C-x3-H type (and similar) | C3H | 2.6E-13 | 49.4 |
| TRINITY_DN61860_c0_g1 | PF00642 | Zf-CCCH | Zinc finger C-x8-C-x5-C-x3-H type (and similar) | C3H | 0.000001 | 28.4 |
| TRINITY_DN45519_c0_g2 | PF00642 | Zf-CCCH | Zinc finger C-x8-C-x5-C-x3-H type (and similar) | C3H | 7.5E-10 | 38.3 |
| TRINITY_DN49099_c0_g1 | PF00642 | Zf-CCCH | Zinc finger C-x8-C-x5-C-x3-H type (and similar) | C3H | 1.2E-19 | 69.5 |
| TRINITY_DN46865_c1_g6 | PF00642 | Zf-CCCH | Zinc finger C-x8-C-x5-C-x3-H type (and similar) | C3H | 1.6E-12 | 46.9 |
| TRINITY_DN51278_c0_g4 | PF00642 | Zf-CCCH | Zinc finger C-x8-C-x5-C-x3-H type (and similar) | C3H | 2.5E-09 | 36.7 |
| TRINITY_DN51278_c0_g2 | PF00642 | Zf-CCCH | Zinc finger C-x8-C-x5-C-x3-H type (and similar) | C3H | 1.8E-24 | 84.9 |
| TRINITY_DN51278_c0_g2 | PF00642 | Zf-CCCH | Zinc finger C-x8-C-x5-C-x3-H type (and similar) | C3H | 0.0000076 | 25.6 |
| TRINITY_DN51278_c0_g5 | PF00642 | Zf-CCCH | Zinc finger C-x8-C-x5-C-x3-H type (and similar) | C3H | 8.8E-31 | 105.1 |
| TRINITY_DN48325_c2_g1 | PF00642 | Zf-CCCH | Zinc finger C-x8-C-x5-C-x3-H type (and similar) | C3H | 1.3E-13 | 50.3 |
| TRINITY_DN72241_c1_g8 | PF00642 | Zf-CCCH | Zinc finger C-x8-C-x5-C-x3-H type (and similar) | C3H | 0.00000021 | 30.5 |
| TRINITY_DN60281_c4_g2 | PF00642 | Zf-CCCH | Zinc finger C-x8-C-x5-C-x3-H type (and similar) | C3H | 0.00038 | 20.2 |
| TRINITY_DN60281_c4_g1 | PF00642 | Zf-CCCH | Zinc finger C-x8-C-x5-C-x3-H type (and similar) | C3H | 0.0000099 | 25.2 |
| TRINITY_DN65907_c0_g8 | PF00642 | Zf-CCCH | Zinc finger C-x8-C-x5-C-x3-H type (and similar) | C3H | 0.000000021 | 33.7 |
| TRINITY_DN67954_c3_g1 | PF00642 | Zf-CCCH | Zinc finger C-x8-C-x5-C-x3-H type (and similar) | C3H | 7.9E-14 | 51 |
| TRINITY_DN58424_c0_g3 | PF00642 | Zf-CCCH | Zinc finger C-x8-C-x5-C-x3-H type (and similar) | C3H | 0.00048 | 19.8 |
| TRINITY_DN58424_c1_g1 | PF00642 | Zf-CCCH | Zinc finger C-x8-C-x5-C-x3-H type (and similar) | C3H | 3.9E-47 | 157.2 |
| TRINITY_DN75825_c1_g2 | PF00642 | Zf-CCCH | Zinc finger C-x8-C-x5-C-x3-H type (and similar) | C3H | 0.0000057 | 26 |
| TRINITY_DN73679_c4_g1 | PF00642 | Zf-CCCH | Zinc finger C-x8-C-x5-C-x3-H type (and similar) | C3H | 0.000014 | 24.7 |
| TRINITY_DN73172_c0_g3 | PF00642 | Zf-CCCH | Zinc finger C-x8-C-x5-C-x3-H type (and similar) | C3H | 2.7E-25 | 87.6 |
| TRINITY_DN73172_c0_g3 | PF00642 | Zf-CCCH | Zinc finger C-x8-C-x5-C-x3-H type (and similar) | C3H | 1.1E-16 | 60.1 |
| TRINITY_DN58352_c5_g3 | PF00642 | Zf-CCCH | Zinc finger C-x8-C-x5-C-x3-H type (and similar) | C3H | 0.00025 | 20.8 |
| TRINITY_DN66374_c0_g1 | PF00642 | Zf-CCCH | Zinc finger C-x8-C-x5-C-x3-H type (and similar) | C3H | 0.0000071 | 25.7 |
| TRINITY_DN64343_c2_g1 | PF00642 | Zf-CCCH | Zinc finger C-x8-C-x5-C-x3-H type (and similar) | C3H | 0.0002 | 21 |
| TRINITY_DN46776_c0_g1 | PF00642 | Zf-CCCH | Zinc finger C-x8-C-x5-C-x3-H type (and similar) | C3H | 0.000027 | 23.8 |
| TRINITY_DN64973_c0_g1 | PF00642 | Zf-CCCH | Zinc finger C-x8-C-x5-C-x3-H type (and similar) | C3H | 1.4E-14 | 53.4 |
| TRINITY_DN26096_c0_g1 | PF00642 | Zf-CCCH | Zinc finger C-x8-C-x5-C-x3-H type (and similar) | C3H | 0.0000011 | 28.2 |
| TRINITY_DN49923_c0_g1 | PF00642 | Zf-CCCH | Zinc finger C-x8-C-x5-C-x3-H type (and similar) | C3H | 0.00002 | 24.2 |
| TRINITY_DN44170_c0_g1 | PF00642 | Zf-CCCH | Zinc finger C-x8-C-x5-C-x3-H type (and similar) | C3H | 0.000000099 | 31.6 |
| TRINITY_DN56238_c0_g1 | PF00642 | Zf-CCCH | Zinc finger C-x8-C-x5-C-x3-H type (and similar) | C3H | 3.1E-15 | 55.5 |
| TRINITY_DN62477_c0_g3 | PF00642 | Zf-CCCH | Zinc finger C-x8-C-x5-C-x3-H type (and similar) | C3H | 0.0000093 | 25.3 |
| TRINITY_DN62477_c0_g5 | PF00642 | Zf-CCCH | Zinc finger C-x8-C-x5-C-x3-H type (and similar) | C3H | 0.000024 | 24 |
| TRINITY_DN53443_c5_g2 | PF00642 | Zf-CCCH | Zinc finger C-x8-C-x5-C-x3-H type (and similar) | C3H | 7.9E-10 | 38.3 |
| TRINITY_DN53627_c4_g1 | PF00642 | Zf-CCCH | Zinc finger C-x8-C-x5-C-x3-H type (and similar) | C3H | 4.8E-21 | 74 |
| TRINITY_DN75513_c1_g3 | PF00642 | Zf-CCCH | Zinc finger C-x8-C-x5-C-x3-H type (and similar) | C3H | 0.00001 | 25.1 |
| TRINITY_DN76660_c8_g1 | PF00642 | Zf-CCCH | Zinc finger C-x8-C-x5-C-x3-H type (and similar) | C3H | 0.00013 | 21.7 |
| TRINITY_DN54394_c4_g2 | PF00642 | Zf-CCCH | Zinc finger C-x8-C-x5-C-x3-H type (and similar) | C3H | 1.5E-33 | 113.9 |
| TRINITY_DN54394_c4_g3 | PF00642 | Zf-CCCH | Zinc finger C-x8-C-x5-C-x3-H type (and similar) | C3H | 0.00058 | 19.6 |
| TRINITY_DN42865_c0_g1 | PF00642 | Zf-CCCH | Zinc finger C-x8-C-x5-C-x3-H type (and similar) | C3H | 2.3E-16 | 59.1 |
| TRINITY_DN93713_c0_g1 | PF00642 | Zf-CCCH | Zinc finger C-x8-C-x5-C-x3-H type (and similar) | C3H | 0.0000051 | 26.1 |
| TRINITY_DN69514_c2_g1 | PF00642 | Zf-CCCH | Zinc finger C-x8-C-x5-C-x3-H type (and similar) | C3H | 7.5E-29 | 98.9 |
| TRINITY_DN69514_c2_g1 | PF00642 | Zf-CCCH | Zinc finger C-x8-C-x5-C-x3-H type (and similar) | C3H | 8.7E-18 | 63.6 |
| TRINITY_DN45774_c0_g1 | PF00642 | Zf-CCCH | Zinc finger C-x8-C-x5-C-x3-H type (and similar) | C3H | 0.00000014 | 31.1 |
| TRINITY_DN63762_c0_g3 | PF00642 | Zf-CCCH | Zinc finger C-x8-C-x5-C-x3-H type (and similar) | C3H | 0.000000022 | 33.7 |
| TRINITY_DN40935_c0_g1 | PF00642 | Zf-CCCH | Zinc finger C-x8-C-x5-C-x3-H type (and similar) | C3H | 1.2E-10 | 40.9 |
| TRINITY_DN70039_c0_g2 | PF00642 | Zf-CCCH | Zinc finger C-x8-C-x5-C-x3-H type (and similar) | C3H | 0.00000017 | 30.8 |
| TRINITY_DN50367_c0_g3 | PF00642 | Zf-CCCH | Zinc finger C-x8-C-x5-C-x3-H type (and similar) | C3H | 0.00045 | 19.9 |
| TRINITY_DN58239_c6_g1 | PF00642 | Zf-CCCH | Zinc finger C-x8-C-x5-C-x3-H type (and similar) | C3H | 0.000000026 | 33.4 |
| TRINITY_DN60207_c1_g2 | PF00642 | Zf-CCCH | Zinc finger C-x8-C-x5-C-x3-H type (and similar) | C3H | 9.1E-13 | 47.6 |
| TRINITY_DN65516_c5_g4 | PF00642 | Zf-CCCH | Zinc finger C-x8-C-x5-C-x3-H type (and similar) | C3H | 2.3E-45 | 151.5 |
| TRINITY_DN53802_c2_g1 | PF00642 | Zf-CCCH | Zinc finger C-x8-C-x5-C-x3-H type (and similar) | C3H | 1.6E-17 | 62.8 |
| TRINITY_DN53802_c2_g2 | PF00642 | Zf-CCCH | Zinc finger C-x8-C-x5-C-x3-H type (and similar) | C3H | 2.1E-18 | 65.6 |
| TRINITY_DN73986_c2_g2 | PF00642 | Zf-CCCH | Zinc finger C-x8-C-x5-C-x3-H type (and similar) | C3H | 6.2E-12 | 45 |
| TRINITY_DN73986_c2_g1 | PF00642 | Zf-CCCH | Zinc finger C-x8-C-x5-C-x3-H type (and similar) | C3H | 0.0000076 | 25.6 |
| TRINITY_DN56562_c4_g3 | PF00642 | Zf-CCCH | Zinc finger C-x8-C-x5-C-x3-H type (and similar) | C3H | 6.3E-40 | 134.2 |
| TRINITY_DN56562_c4_g1 | PF00642 | Zf-CCCH | Zinc finger C-x8-C-x5-C-x3-H type (and similar) | C3H | 4.9E-42 | 140.9 |
| TRINITY_DN63342_c2_g1 | PF00642 | Zf-CCCH | Zinc finger C-x8-C-x5-C-x3-H type (and similar) | C3H | 0.0000044 | 26.3 |
| TRINITY_DN75902_c3_g2 | PF00642 | Zf-CCCH | Zinc finger C-x8-C-x5-C-x3-H type (and similar) | C3H | 0.0000014 | 27.9 |
| TRINITY_DN28457_c0_g1 | PF00046 | homeobox | Homeobox domain | LBD (AS2/LOB) | 0.00000032 | 29.9 |
| TRINITY_DN52789_c0_g3 | PF00046 | homeobox | Homeobox domain | LBD (AS2/LOB) | 1.5E-18 | 66.2 |
| TRINITY_DN63854_c4_g1 | PF00046 | homeobox | Homeobox domain | LBD (AS2/LOB) | 0.00000016 | 30.9 |
| TRINITY_DN59889_c5_g1 | PF00046 | homeobox | Homeobox domain | LBD (AS2/LOB) | 2.4E-18 | 65.5 |
| TRINITY_DN59889_c5_g4 | PF00046 | homeobox | Homeobox domain | LBD (AS2/LOB) | 3.1E-09 | 36.4 |
| TRINITY_DN67151_c5_g1 | PF00046 | homeobox | Homeobox domain | LBD (AS2/LOB) | 1.8E-17 | 62.8 |
| TRINITY_DN60890_c1_g1 | PF00046 | homeobox | Homeobox domain | LBD (AS2/LOB) | 1.1E-19 | 69.9 |
| TRINITY_DN61510_c5_g6 | PF00046 | homeobox | Homeobox domain | LBD (AS2/LOB) | 0.00000018 | 30.7 |
| TRINITY_DN45525_c2_g2 | PF00046 | homeobox | Homeobox domain | LBD (AS2/LOB) | 8.8E-15 | 54.1 |
| TRINITY_DN45525_c2_g3 | PF00046 | homeobox | Homeobox domain | LBD (AS2/LOB) | 2.3E-17 | 62.4 |
| TRINITY_DN45525_c2_g1 | PF00046 | homeobox | Homeobox domain | LBD (AS2/LOB) | 1.8E-15 | 56.4 |
| TRINITY_DN45525_c2_g4 | PF00046 | homeobox | Homeobox domain | LBD (AS2/LOB) | 5E-16 | 58.1 |
| TRINITY_DN49427_c0_g10 | PF00046 | homeobox | Homeobox domain | LBD (AS2/LOB) | 0.000043 | 23.1 |
| TRINITY_DN42453_c0_g1 | PF00046 | homeobox | Homeobox domain | LBD (AS2/LOB) | 8.6E-19 | 67 |
| TRINITY_DN54686_c2_g2 | PF00046 | homeobox | Homeobox domain | LBD (AS2/LOB) | 3.2E-18 | 65.2 |
| TRINITY_DN56660_c1_g1 | PF00046 | homeobox | Homeobox domain | LBD (AS2/LOB) | 6.6E-09 | 35.3 |
| TRINITY_DN43391_c0_g1 | PF00046 | homeobox | Homeobox domain | LBD (AS2/LOB) | 2.5E-17 | 62.3 |
| TRINITY_DN55710_c3_g2 | PF00046 | homeobox | Homeobox domain | LBD (AS2/LOB) | 1.7E-15 | 56.4 |
| TRINITY_DN64279_c1_g7 | PF00046 | homeobox | Homeobox domain | LBD (AS2/LOB) | 0.000028 | 23.7 |
| TRINITY_DN45807_c0_g1 | PF00046 | homeobox | Homeobox domain | LBD (AS2/LOB) | 1.4E-09 | 37.5 |
| TRINITY_DN65102_c0_g1 | PF00046 | homeobox | Homeobox domain | LBD (AS2/LOB) | 7.5E-21 | 73.6 |
| TRINITY_DN65549_c0_g1 | PF00046 | homeobox | Homeobox domain | LBD (AS2/LOB) | 0.00026 | 20.6 |
| TRINITY_DN68857_c0_g1 | PF00046 | homeobox | Homeobox domain | LBD (AS2/LOB) | 2.7E-18 | 65.4 |
| TRINITY_DN73205_c6_g1 | PF00046 | homeobox | Homeobox domain | LBD (AS2/LOB) | 0.000018 | 24.3 |
| TRINITY_DN47457_c1_g2 | PF00046 | homeobox | Homeobox domain | LBD (AS2/LOB) | 0.0000017 | 27.6 |
| TRINITY_DN51725_c0_g1 | PF00046 | homeobox | Homeobox domain | LBD (AS2/LOB) | 0.00000027 | 30.2 |
| TRINITY_DN44388_c0_g1 | PF00046 | homeobox | Homeobox domain | LBD (AS2/LOB) | 4E-17 | 61.6 |
| TRINITY_DN44388_c0_g2 | PF00046 | homeobox | Homeobox domain | LBD (AS2/LOB) | 1.9E-18 | 65.9 |
| TRINITY_DN64703_c0_g1 | PF00046 | homeobox | Homeobox domain | LBD (AS2/LOB) | 4.8E-17 | 61.4 |
| TRINITY_DN64703_c0_g2 | PF00046 | homeobox | Homeobox domain | LBD (AS2/LOB) | 0.000016 | 24.5 |
| TRINITY_DN47127_c1_g3 | PF00046 | homeobox | Homeobox domain | LBD (AS2/LOB) | 3.8E-20 | 71.3 |
| TRINITY_DN47127_c1_g2 | PF00046 | homeobox | Homeobox domain | LBD (AS2/LOB) | 7.4E-19 | 67.2 |
| TRINITY_DN73119_c0_g2 | PF00046 | homeobox | Homeobox domain | LBD (AS2/LOB) | 8.2E-17 | 60.6 |
| TRINITY_DN73119_c0_g1 | PF00046 | homeobox | Homeobox domain | LBD (AS2/LOB) | 3.2E-15 | 55.5 |
| TRINITY_DN64602_c2_g1 | PF00046 | homeobox | Homeobox domain | LBD (AS2/LOB) | 2.1E-12 | 46.5 |
| TRINITY_DN75484_c1_g1 | PF00046 | homeobox | Homeobox domain | LBD (AS2/LOB) | 0.00000059 | 29.1 |
| TRINITY_DN55032_c0_g1 | PF00046 | homeobox | Homeobox domain | LBD (AS2/LOB) | 3.1E-16 | 58.8 |
| TRINITY_DN66948_c0_g1 | PF00046 | homeobox | Homeobox domain | LBD (AS2/LOB) | 1E-15 | 57.1 |
| TRINITY_DN71219_c5_g1 | PF00046 | homeobox | Homeobox domain | LBD (AS2/LOB) | 3.5E-19 | 68.2 |
| TRINITY_DN51494_c1_g3 | PF00046 | homeobox | Homeobox domain | LBD (AS2/LOB) | 0.00000058 | 29.1 |
| TRINITY_DN56491_c3_g2 | PF00046 | homeobox | Homeobox domain | LBD (AS2/LOB) | 0.00000012 | 31.3 |
| TRINITY_DN43984_c0_g1 | PF00046 | homeobox | Homeobox domain | LBD (AS2/LOB) | 9.3E-17 | 60.5 |
| TRINITY_DN52195_c0_g1 | PF00046 | homeobox | Homeobox domain | LBD (AS2/LOB) | 0.00000024 | 30.3 |
| TRINITY_DN75200_c3_g1 | PF00046 | homeobox | Homeobox domain | LBD (AS2/LOB) | 2E-10 | 40.2 |
| TRINITY_DN57544_c2_g2 | PF00046 | homeobox | Homeobox domain | LBD (AS2/LOB) | 1.1E-15 | 57 |
| TRINITY_DN73433_c1_g2 | PF00046 | homeobox | Homeobox domain | LBD (AS2/LOB) | 0.000001 | 28.3 |
| TRINITY_DN73211_c2_g2 | PF00046 | homeobox | Homeobox domain | LBD (AS2/LOB) | 3E-10 | 39.6 |
| TRINITY_DN73211_c2_g1 | PF00046 | homeobox | Homeobox domain | LBD (AS2/LOB) | 3.6E-16 | 58.6 |
| TRINITY_DN70968_c0_g2 | PF00046 | homeobox | Homeobox domain | LBD (AS2/LOB) | 0.000000062 | 32.2 |
| TRINITY_DN69602_c0_g1 | PF08879 | WRC | WRC | GRF | 3.3E-14 | 52.2 |
| TRINITY_DN69602_c1_g2 | PF08879 | WRC | WRC | GRF | 2.8E-11 | 42.9 |
| TRINITY_DN69602_c1_g1 | PF08879 | WRC | WRC | GRF | 8.6E-14 | 50.9 |
| TRINITY_DN71102_c1_g2 | PF08879 | WRC | WRC | GRF | 3.8E-11 | 42.4 |
| TRINITY_DN74511_c0_g2 | PF08879 | WRC | WRC | GRF | 4.6E-15 | 55 |
| TRINITY_DN48388_c1_g2 | PF08879 | WRC | WRC | GRF | 7.3E-20 | 70.3 |
| TRINITY_DN43868_c0_g1 | PF08879 | WRC | WRC | GRF | 1.2E-11 | 44 |
| TRINITY_DN43944_c0_g1 | PF08879 | WRC | WRC | GRF | 6.2E-14 | 51.4 |
| TRINITY_DN43323_c0_g1 | PF08879 | WRC | WRC | GRF | 1.9E-14 | 53 |
| TRINITY_DN73496_c0_g3 | PF08879 | WRC | WRC | GRF | 4.9E-11 | 42.1 |
| TRINITY_DN68598_c0_g2 | PF08879 | WRC | WRC | GRF | 1.4E-11 | 43.9 |
| TRINITY_DN58499_c0_g1 | PF08879 | WRC | WRC | GRF | 1E-13 | 50.7 |
| TRINITY_DN21587_c0_g1 | PF08879 | WRC | WRC | GRF | 2.4E-11 | 43.1 |
| TRINITY_DN51186_c3_g6 | PF08879 | WRC | WRC | GRF | 0.0001 | 21.9 |
| TRINITY_DN49263_c0_g1 | PF08879 | WRC | WRC | GRF | 7.2E-12 | 44.7 |
| TRINITY_DN49263_c0_g2 | PF08879 | WRC | WRC | GRF | 6.4E-15 | 54.5 |
| TRINITY_DN74757_c0_g1 | PF08879 | WRC | WRC | GRF | 4.6E-09 | 35.8 |
| TRINITY_DN68549_c1_g1 | PF08879 | WRC | WRC | GRF | 1.5E-09 | 37.4 |
| TRINITY_DN68549_c1_g3 | PF08879 | WRC | WRC | GRF | 4E-11 | 42.4 |
| TRINITY_DN44900_c0_g1 | PF08879 | WRC | WRC | GRF | 2.5E-14 | 52.6 |
| TRINITY_DN63596_c0_g2 | PF08879 | WRC | WRC | GRF | 0.0000035 | 26.5 |
| TRINITY_DN63596_c0_g1 | PF08879 | WRC | WRC | GRF | 2.3E-22 | 78.3 |
| TRINITY_DN65829_c1_g2 | PF08879 | WRC | WRC | GRF | 1.6E-12 | 46.8 |
| TRINITY_DN65829_c1_g3 | PF08879 | WRC | WRC | GRF | 6.4E-12 | 44.9 |
| TRINITY_DN47487_c2_g2 | PF08879 | WRC | WRC | GRF | 5.7E-21 | 73.9 |
| TRINITY_DN47487_c2_g3 | PF08879 | WRC | WRC | GRF | 4.3E-20 | 71.1 |
| TRINITY_DN75011_c3_g3 | PF05142 | DUF702 | Domain of unknown function (DUF702) | SRS | 5.3E-31 | 108 |
| TRINITY_DN75011_c3_g4 | PF05142 | DUF702 | Domain of unknown function (DUF702) | SRS | 3.5E-20 | 72.9 |
| TRINITY_DN65149_c1_g2 | PF05142 | DUF702 | Domain of unknown function (DUF702) | SRS | 0.00009 | 22.9 |
| TRINITY_DN50657_c0_g1 | PF05142 | DUF702 | Domain of unknown function (DUF702) | SRS | 8.7E-14 | 52.1 |
| TRINITY_DN38822_c0_g1 | PF05142 | DUF702 | Domain of unknown function (DUF702) | SRS | 8.7E-61 | 204.6 |
| TRINITY_DN75786_c1_g1 | PF05142 | DUF702 | Domain of unknown function (DUF702) | SRS | 7.5E-62 | 208.1 |
| TRINITY_DN49070_c0_g2 | PF03859 | CG1 | CG-1 domain | CAMTA | 0.000063 | 22.6 |
| TRINITY_DN70208_c1_g9 | PF03859 | CG1 | CG-1 domain | CAMTA | 1.8E-48 | 163.1 |
| TRINITY_DN53764_c0_g1 | PF03859 | CG1 | CG-1 domain | CAMTA | 1.2E-46 | 157.2 |
| TRINITY_DN58102_c2_g4 | PF03859 | CG1 | CG-1 domain | CAMTA | 2.3E-50 | 169.1 |
| TRINITY_DN75436_c3_g9 | PF03859 | CG1 | CG-1 domain | CAMTA | 8.8E-10 | 38.3 |
| TRINITY_DN65399_c2_g2 | PF03859 | CG1 | CG-1 domain | CAMTA | 4.6E-44 | 148.8 |
| TRINITY_DN45073_c0_g1 | PF00447 | HSF_dna_bind | HSF-type DNA-binding | HSF | 1.4E-15 | 57.5 |
| TRINITY_DN59675_c3_g2 | PF00447 | HSF_dna_bind | HSF-type DNA-binding | HSF | 9.5E-21 | 74.1 |
| TRINITY_DN59675_c3_g4 | PF00447 | HSF_dna_bind | HSF-type DNA-binding | HSF | 1.3E-29 | 102.5 |
| TRINITY_DN49177_c2_g1 | PF00447 | HSF_dna_bind | HSF-type DNA-binding | HSF | 2.1E-29 | 101.9 |
| TRINITY_DN49177_c2_g5 | PF00447 | HSF_dna_bind | HSF-type DNA-binding | HSF | 2.4E-16 | 60 |
| TRINITY_DN49177_c2_g4 | PF00447 | HSF_dna_bind | HSF-type DNA-binding | HSF | 2E-30 | 105.1 |
| TRINITY_DN49177_c2_g7 | PF00447 | HSF_dna_bind | HSF-type DNA-binding | HSF | 1.7E-29 | 102.1 |
| TRINITY_DN59282_c0_g2 | PF00447 | HSF_dna_bind | HSF-type DNA-binding | HSF | 1.3E-30 | 105.7 |
| TRINITY_DN59282_c0_g1 | PF00447 | HSF_dna_bind | HSF-type DNA-binding | HSF | 3.5E-31 | 107.5 |
| TRINITY_DN53654_c0_g2 | PF00447 | HSF_dna_bind | HSF-type DNA-binding | HSF | 1.2E-19 | 70.5 |
| TRINITY_DN53654_c0_g3 | PF00447 | HSF_dna_bind | HSF-type DNA-binding | HSF | 5.4E-15 | 55.6 |
| TRINITY_DN53654_c0_g4 | PF00447 | HSF_dna_bind | HSF-type DNA-binding | HSF | 3.9E-20 | 72.1 |
| TRINITY_DN61595_c2_g1 | PF00447 | HSF_dna_bind | HSF-type DNA-binding | HSF | 5.1E-32 | 110.2 |
| TRINITY_DN66926_c0_g4 | PF00447 | HSF_dna_bind | HSF-type DNA-binding | HSF | 5.2E-23 | 81.3 |
| TRINITY_DN66926_c0_g8 | PF00447 | HSF_dna_bind | HSF-type DNA-binding | HSF | 1.9E-25 | 89.1 |
| TRINITY_DN66926_c0_g9 | PF00447 | HSF_dna_bind | HSF-type DNA-binding | HSF | 4.9E-23 | 81.4 |
| TRINITY_DN66926_c0_g3 | PF00447 | HSF_dna_bind | HSF-type DNA-binding | HSF | 7.8E-27 | 93.6 |
| TRINITY_DN45466_c0_g1 | PF00447 | HSF_dna_bind | HSF-type DNA-binding | HSF | 7.4E-13 | 48.8 |
| TRINITY_DN58195_c1_g1 | PF00447 | HSF_dna_bind | HSF-type DNA-binding | HSF | 7.6E-31 | 106.5 |
| TRINITY_DN58195_c1_g3 | PF00447 | HSF_dna_bind | HSF-type DNA-binding | HSF | 1.6E-30 | 105.4 |
| TRINITY_DN58195_c1_g4 | PF00447 | HSF_dna_bind | HSF-type DNA-binding | HSF | 4.9E-32 | 110.3 |
| TRINITY_DN67126_c1_g1 | PF00447 | HSF_dna_bind | HSF-type DNA-binding | HSF | 4.8E-19 | 68.6 |
| TRINITY_DN67126_c1_g3 | PF00447 | HSF_dna_bind | HSF-type DNA-binding | HSF | 9.1E-25 | 87 |
| TRINITY_DN67126_c1_g4 | PF00447 | HSF_dna_bind | HSF-type DNA-binding | HSF | 4.7E-31 | 107.2 |
| TRINITY_DN44153_c0_g1 | PF00447 | HSF_dna_bind | HSF-type DNA-binding | HSF | 1.4E-12 | 47.9 |
| TRINITY_DN27874_c0_g1 | PF00447 | HSF_dna_bind | HSF-type DNA-binding | HSF | 4.5E-10 | 39.8 |
| TRINITY_DN37578_c0_g1 | PF00447 | HSF_dna_bind | HSF-type DNA-binding | HSF | 0.000053 | 23.6 |
| TRINITY_DN80151_c0_g1 | PF00447 | HSF_dna_bind | HSF-type DNA-binding | HSF | 0.00011 | 22.5 |
| TRINITY_DN71095_c3_g6 | PF00447 | HSF_dna_bind | HSF-type DNA-binding | HSF | 7.9E-30 | 103.2 |
| TRINITY_DN71095_c3_g10 | PF00447 | HSF_dna_bind | HSF-type DNA-binding | HSF | 2.4E-32 | 111.3 |
| TRINITY_DN71095_c3_g2 | PF00447 | HSF_dna_bind | HSF-type DNA-binding | HSF | 8.9E-27 | 93.4 |
| TRINITY_DN67247_c3_g6 | PF00447 | HSF_dna_bind | HSF-type DNA-binding | HSF | 4.6E-31 | 107.2 |
| TRINITY_DN67247_c3_g8 | PF00447 | HSF_dna_bind | HSF-type DNA-binding | HSF | 1.7E-31 | 108.6 |
| TRINITY_DN75423_c3_g1 | PF00447 | HSF_dna_bind | HSF-type DNA-binding | HSF | 8.3E-21 | 74.3 |
| TRINITY_DN59596_c0_g1 | PF03106 | WRKY | WRKY DNA -binding domain | WRKY | 7.4E-21 | 74.1 |
| TRINITY_DN59596_c0_g2 | PF03106 | WRKY | WRKY DNA -binding domain | WRKY | 1.5E-20 | 73.2 |
| TRINITY_DN71755_c1_g1 | PF03106 | WRKY | WRKY DNA -binding domain | WRKY | 0.00000014 | 31.6 |
| TRINITY_DN44790_c0_g1 | PF03106 | WRKY | WRKY DNA -binding domain | WRKY | 1.2E-26 | 92.7 |
| TRINITY_DN44790_c0_g2 | PF03106 | WRKY | WRKY DNA -binding domain | WRKY | 0.0000057 | 26.5 |
| TRINITY_DN44790_c0_g2 | PF03106 | WRKY | WRKY DNA -binding domain | WRKY | 9.3E-15 | 54.6 |
| TRINITY_DN28147_c0_g1 | PF03106 | WRKY | WRKY DNA -binding domain | WRKY | 0.00000056 | 29.7 |
| TRINITY_DN28147_c0_g1 | PF03106 | WRKY | WRKY DNA -binding domain | WRKY | 8.2E-09 | 35.6 |
| TRINITY_DN46929_c0_g2 | PF03106 | WRKY | WRKY DNA -binding domain | WRKY | 0.0000086 | 25.9 |
| TRINITY_DN46929_c0_g10 | PF03106 | WRKY | WRKY DNA -binding domain | WRKY | 4.6E-25 | 87.6 |
| TRINITY_DN41883_c0_g1 | PF03106 | WRKY | WRKY DNA -binding domain | WRKY | 1E-25 | 89.7 |
| TRINITY_DN48990_c2_g2 | PF03106 | WRKY | WRKY DNA -binding domain | WRKY | 6E-24 | 84 |
| TRINITY_DN48990_c2_g6 | PF03106 | WRKY | WRKY DNA -binding domain | WRKY | 1.6E-23 | 82.7 |
| TRINITY_DN64748_c1_g3 | PF03106 | WRKY | WRKY DNA -binding domain | WRKY | 1.1E-51 | 172.8 |
| TRINITY_DN64748_c1_g2 | PF03106 | WRKY | WRKY DNA -binding domain | WRKY | 4.8E-26 | 90.8 |
| TRINITY_DN23211_c0_g1 | PF03106 | WRKY | WRKY DNA -binding domain | WRKY | 8.7E-25 | 86.7 |
| TRINITY_DN43924_c0_g1 | PF03106 | WRKY | WRKY DNA -binding domain | WRKY | 6.3E-27 | 93.6 |
| TRINITY_DN49778_c0_g2 | PF03106 | WRKY | WRKY DNA -binding domain | WRKY | 0.000000034 | 33.6 |
| TRINITY_DN44662_c0_g1 | PF03106 | WRKY | WRKY DNA -binding domain | WRKY | 5.3E-27 | 93.8 |
| TRINITY_DN42240_c0_g1 | PF03106 | WRKY | WRKY DNA -binding domain | WRKY | 2.9E-25 | 88.3 |
| TRINITY_DN54361_c3_g3 | PF03106 | WRKY | WRKY DNA -binding domain | WRKY | 4E-17 | 62.2 |
| TRINITY_DN58036_c6_g2 | PF03106 | WRKY | WRKY DNA -binding domain | WRKY | 1.7E-26 | 92.2 |
| TRINITY_DN36204_c0_g1 | PF03106 | WRKY | WRKY DNA -binding domain | WRKY | 3E-26 | 91.4 |
| TRINITY_DN58322_c0_g1 | PF03106 | WRKY | WRKY DNA -binding domain | WRKY | 1.4E-24 | 86 |
| TRINITY_DN58322_c0_g2 | PF03106 | WRKY | WRKY DNA -binding domain | WRKY | 1.2E-22 | 79.9 |
| TRINITY_DN58322_c0_g4 | PF03106 | WRKY | WRKY DNA -binding domain | WRKY | 4.6E-25 | 87.6 |
| TRINITY_DN39825_c0_g1 | PF03106 | WRKY | WRKY DNA -binding domain | WRKY | 7.5E-27 | 93.3 |
| TRINITY_DN44378_c0_g2 | PF03106 | WRKY | WRKY DNA -binding domain | WRKY | 3E-26 | 91.4 |
| TRINITY_DN44378_c0_g3 | PF03106 | WRKY | WRKY DNA -binding domain | WRKY | 9.8E-26 | 89.8 |
| TRINITY_DN26994_c0_g1 | PF03106 | WRKY | WRKY DNA -binding domain | WRKY | 1.7E-23 | 82.6 |
| TRINITY_DN43205_c0_g1 | PF03106 | WRKY | WRKY DNA -binding domain | WRKY | 7E-27 | 93.4 |
| TRINITY_DN43217_c0_g1 | PF03106 | WRKY | WRKY DNA -binding domain | WRKY | 9.9E-24 | 83.3 |
| TRINITY_DN57705_c2_g1 | PF03106 | WRKY | WRKY DNA -binding domain | WRKY | 3.3E-27 | 94.5 |
| TRINITY_DN61619_c1_g8 | PF03106 | WRKY | WRKY DNA -binding domain | WRKY | 5.8E-51 | 170.6 |
| TRINITY_DN50987_c0_g1 | PF03106 | WRKY | WRKY DNA -binding domain | WRKY | 2.7E-21 | 75.5 |
| TRINITY_DN50987_c1_g1 | PF03106 | WRKY | WRKY DNA -binding domain | WRKY | 2.8E-23 | 81.9 |
| TRINITY_DN50987_c1_g2 | PF03106 | WRKY | WRKY DNA -binding domain | WRKY | 9.7E-21 | 73.8 |
| TRINITY_DN49869_c1_g3 | PF03106 | WRKY | WRKY DNA -binding domain | WRKY | 1.4E-22 | 79.7 |
| TRINITY_DN49869_c1_g2 | PF03106 | WRKY | WRKY DNA -binding domain | WRKY | 0.00000019 | 31.2 |
| TRINITY_DN49869_c1_g4 | PF03106 | WRKY | WRKY DNA -binding domain | WRKY | 2E-25 | 88.8 |
| TRINITY_DN50242_c0_g1 | PF03106 | WRKY | WRKY DNA -binding domain | WRKY | 1.6E-24 | 85.8 |
| TRINITY_DN38994_c0_g1 | PF03106 | WRKY | WRKY DNA -binding domain | WRKY | 1.6E-24 | 85.8 |
| TRINITY_DN66892_c1_g4 | PF03106 | WRKY | WRKY DNA -binding domain | WRKY | 3.2E-51 | 171.4 |
| TRINITY_DN39293_c0_g1 | PF03106 | WRKY | WRKY DNA -binding domain | WRKY | 6.1E-26 | 90.4 |
| TRINITY_DN39293_c0_g4 | PF03106 | WRKY | WRKY DNA -binding domain | WRKY | 1.1E-13 | 51.2 |
| TRINITY_DN38800_c0_g1 | PF03106 | WRKY | WRKY DNA -binding domain | WRKY | 3.9E-49 | 164.7 |
| TRINITY_DN44495_c0_g1 | PF03106 | WRKY | WRKY DNA -binding domain | WRKY | 7.1E-24 | 83.8 |
| TRINITY_DN44833_c0_g1 | PF03106 | WRKY | WRKY DNA -binding domain | WRKY | 0.00000041 | 30.1 |
| TRINITY_DN48198_c0_g1 | PF03106 | WRKY | WRKY DNA -binding domain | WRKY | 0.000000099 | 32.1 |
| TRINITY_DN48198_c0_g2 | PF03106 | WRKY | WRKY DNA -binding domain | WRKY | 2.4E-25 | 88.5 |
| TRINITY_DN65846_c0_g1 | PF03106 | WRKY | WRKY DNA -binding domain | WRKY | 1.1E-49 | 166.4 |
| TRINITY_DN65159_c2_g2 | PF02045 | CBFB_NFYA | CCAAT-binding transcription factor (CBF-B/NF-YA) subunit B | NF-Y | 2.1E-12 | 47.4 |
| TRINITY_DN65159_c2_g2 | PF02045 | CBFB_NFYA | CCAAT-binding transcription factor (CBF-B/NF-YA) subunit B | NF-Y | 6.6E-10 | 39.4 |
| TRINITY_DN67937_c1_g1 | PF02045 | CBFB_NFYA | CCAAT-binding transcription factor (CBF-B/NF-YA) subunit B | NF-Y | 3.3E-12 | 46.8 |
| TRINITY_DN60509_c1_g4 | PF02045 | CBFB_NFYA | CCAAT-binding transcription factor (CBF-B/NF-YA) subunit B | NF-Y | 5.1E-28 | 97.4 |
| TRINITY_DN73070_c0_g1 | PF02045 | CBFB_NFYA | CCAAT-binding transcription factor (CBF-B/NF-YA) subunit B | NF-Y | 6.9E-13 | 48.9 |
| TRINITY_DN44081_c0_g1 | PF02045 | CBFB_NFYA | CCAAT-binding transcription factor (CBF-B/NF-YA) subunit B | NF-Y | 1.5E-11 | 44.6 |
| TRINITY_DN70156_c2_g3 | PF04504 | DUF573 | Protein of unknown function, DUF573 | GeBP | 2.1E-29 | 101.9 |
| TRINITY_DN52023_c4_g2 | PF04504 | DUF573 | Protein of unknown function, DUF573 | GeBP | 0.0000075 | 26.4 |
| TRINITY_DN58218_c0_g3 | PF04504 | DUF573 | Protein of unknown function, DUF573 | GeBP | 0.000072 | 23.2 |
| TRINITY_DN58218_c0_g4 | PF04504 | DUF573 | Protein of unknown function, DUF573 | GeBP | 0.00000038 | 30.6 |
| TRINITY_DN58218_c0_g1 | PF04504 | DUF573 | Protein of unknown function, DUF573 | GeBP | 0.000031 | 24.4 |
| TRINITY_DN68520_c2_g4 | PF04504 | DUF573 | Protein of unknown function, DUF573 | GeBP | 1.3E-10 | 41.7 |
| TRINITY_DN26898_c0_g1 | PF04504 | DUF573 | Protein of unknown function, DUF573 | GeBP | 4E-13 | 49.7 |
| TRINITY_DN48196_c3_g1 | PF04504 | DUF573 | Protein of unknown function, DUF573 | GeBP | 0.00058 | 20.3 |
| TRINITY_DN56370_c0_g1 | PF04504 | DUF573 | Protein of unknown function, DUF573 | GeBP | 0.00058 | 20.3 |
| TRINITY_DN42894_c0_g1 | PF04504 | DUF573 | Protein of unknown function, DUF573 | GeBP | 1.2E-13 | 51.4 |
| TRINITY_DN68840_c0_g2 | PF04504 | DUF573 | Protein of unknown function, DUF573 | GeBP | 1.9E-31 | 108.4 |
| TRINITY_DN60742_c1_g3 | PF04504 | DUF573 | Protein of unknown function, DUF573 | GeBP | 1.1E-19 | 70.7 |
| TRINITY_DN44877_c0_g2 | PF04504 | DUF573 | Protein of unknown function, DUF573 | GeBP | 2.6E-25 | 88.8 |
| TRINITY_DN72285_c1_g2 | PF03634 | TCP | TCP family transcription factor | TCP | 1E-33 | 117 |
| TRINITY_DN72285_c1_g3 | PF03634 | TCP | TCP family transcription factor | TCP | 6.9E-24 | 85 |
| TRINITY_DN67161_c2_g2 | PF03634 | TCP | TCP family transcription factor | TCP | 2.1E-15 | 57.5 |
| TRINITY_DN64754_c5_g1 | PF03634 | TCP | TCP family transcription factor | TCP | 6.1E-33 | 114.4 |
| TRINITY_DN27843_c1_g1 | PF03634 | TCP | TCP family transcription factor | TCP | 2E-15 | 57.5 |
| TRINITY_DN72315_c1_g2 | PF03634 | TCP | TCP family transcription factor | TCP | 7.4E-37 | 127.2 |
| TRINITY_DN43733_c0_g1 | PF03634 | TCP | TCP family transcription factor | TCP | 2.9E-28 | 99.2 |
| TRINITY_DN64237_c2_g1 | PF03634 | TCP | TCP family transcription factor | TCP | 1E-41 | 142.9 |
| TRINITY_DN74646_c4_g5 | PF03634 | TCP | TCP family transcription factor | TCP | 5.5E-18 | 65.9 |
| TRINITY_DN74646_c4_g3 | PF03634 | TCP | TCP family transcription factor | TCP | 1.8E-31 | 109.7 |
| TRINITY_DN74646_c4_g6 | PF03634 | TCP | TCP family transcription factor | TCP | 3.3E-41 | 141.3 |
| TRINITY_DN58023_c2_g2 | PF03634 | TCP | TCP family transcription factor | TCP | 7.7E-31 | 107.6 |
| TRINITY_DN58023_c2_g4 | PF03634 | TCP | TCP family transcription factor | TCP | 2.1E-39 | 135.4 |
| TRINITY_DN58023_c2_g5 | PF03634 | TCP | TCP family transcription factor | TCP | 2.3E-29 | 102.8 |
| TRINITY_DN58023_c2_g8 | PF03634 | TCP | TCP family transcription factor | TCP | 6.9E-30 | 104.5 |
| TRINITY_DN58023_c2_g1 | PF03634 | TCP | TCP family transcription factor | TCP | 1.2E-21 | 77.8 |
| TRINITY_DN33856_c0_g1 | PF03634 | TCP | TCP family transcription factor | TCP | 2.3E-39 | 135.3 |
| TRINITY_DN55858_c3_g1 | PF06217 | GAGA_bind | GAGA binding protein-like family | BBR-BPC | 1.9E-66 | 225.2 |
| TRINITY_DN61808_c1_g1 | PF06217 | GAGA_bind | GAGA binding protein-like family | BBR-BPC | 2E-93 | 313.8 |
| TRINITY_DN61808_c1_g5 | PF06217 | GAGA_bind | GAGA binding protein-like family | BBR-BPC | 1.2E-11 | 45.4 |
| TRINITY_DN61808_c1_g2 | PF06217 | GAGA_bind | GAGA binding protein-like family | BBR-BPC | 2.1E-97 | 326.9 |
| TRINITY_DN54625_c1_g2 | PF06217 | GAGA_bind | GAGA binding protein-like family | BBR-BPC | 1.8E-81 | 274.5 |
| TRINITY_DN45177_c0_g1 | PF06217 | GAGA_bind | GAGA binding protein-like family | BBR-BPC | 3E-96 | 323.1 |
| TRINITY_DN44014_c0_g1 | PF06217 | GAGA_bind | GAGA binding protein-like family | BBR-BPC | 1.9E-90 | 304 |
| TRINITY_DN70513_c0_g1 | PF03110 | SBP | SBP domain | SBP | 3.2E-31 | 107.2 |
| TRINITY_DN70513_c1_g1 | PF03110 | SBP | SBP domain | SBP | 1.6E-17 | 63.4 |
| TRINITY_DN65610_c0_g3 | PF03110 | SBP | SBP domain | SBP | 0.0000014 | 28.3 |
| TRINITY_DN65610_c2_g1 | PF03110 | SBP | SBP domain | SBP | 6.2E-19 | 67.9 |
| TRINITY_DN71630_c1_g1 | PF03110 | SBP | SBP domain | SBP | 0.0000037 | 26.9 |
| TRINITY_DN44348_c0_g1 | PF03110 | SBP | SBP domain | SBP | 1.7E-17 | 63.3 |
| TRINITY_DN44348_c0_g2 | PF03110 | SBP | SBP domain | SBP | 0.000012 | 25.3 |
| TRINITY_DN42741_c0_g1 | PF03110 | SBP | SBP domain | SBP | 1.3E-17 | 63.7 |
| TRINITY_DN58351_c1_g2 | PF03110 | SBP | SBP domain | SBP | 1.2E-33 | 115 |
| TRINITY_DN58351_c1_g1 | PF03110 | SBP | SBP domain | SBP | 1.8E-32 | 111.2 |
| TRINITY_DN70452_c2_g4 | PF03110 | SBP | SBP domain | SBP | 1.2E-29 | 102.2 |
| TRINITY_DN50736_c4_g1 | PF03110 | SBP | SBP domain | SBP | 6.7E-32 | 109.4 |
| TRINITY_DN52466_c3_g2 | PF03110 | SBP | SBP domain | SBP | 2.1E-27 | 95 |
| TRINITY_DN46320_c3_g3 | PF03110 | SBP | SBP domain | SBP | 3.5E-31 | 107.1 |
| TRINITY_DN46320_c3_g2 | PF03110 | SBP | SBP domain | SBP | 2.5E-29 | 101.1 |
| TRINITY_DN59922_c0_g2 | PF01422 | Zf-NF-X1 | NF-X1 type zinc finger | NF-X1 | 0.00015 | 21.6 |
| TRINITY_DN59319_c0_g3 | PF01422 | Zf-NF-X1 | NF-X1 type zinc finger | NF-X1 | 2.4E-10 | 40.1 |
| TRINITY_DN40795_c0_g1 | PF00643 | zf-B_box | B-box zinc finger | C2C2 | 0.000000099 | 31.9 |
| TRINITY_DN69266_c4_g1 | PF00643 | zf-B_box | B-box zinc finger | C2C2 | 2.2E-14 | 53.2 |
| TRINITY_DN48387_c4_g3 | PF00643 | zf-B_box | B-box zinc finger | C2C2 | 0.00000074 | 29.1 |
| TRINITY_DN55390_c0_g1 | PF00643 | zf-B_box | B-box zinc finger | C2C2 | 0.000000002 | 37.3 |
| TRINITY_DN57437_c1_g7 | PF00643 | zf-B_box | B-box zinc finger | C2C2 | 7.9E-14 | 51.4 |
| TRINITY_DN68501_c3_g3 | PF00643 | zf-B_box | B-box zinc finger | C2C2 | 0.0000037 | 26.9 |
| TRINITY_DN42579_c0_g1 | PF00643 | zf-B_box | B-box zinc finger | C2C2 | 0.0000041 | 26.7 |
| TRINITY_DN42783_c0_g1 | PF00643 | zf-B_box | B-box zinc finger | C2C2 | 0.00000074 | 29.1 |
| TRINITY_DN56716_c0_g1 | PF00643 | zf-B_box | B-box zinc finger | C2C2 | 0.00064 | 19.7 |
| TRINITY_DN56716_c0_g2 | PF00643 | zf-B_box | B-box zinc finger | C2C2 | 0.00021 | 21.2 |
| TRINITY_DN50957_c5_g1 | PF00643 | zf-B_box | B-box zinc finger | C2C2 | 0.00076 | 19.5 |
| TRINITY_DN47002_c2_g1 | PF00643 | zf-B_box | B-box zinc finger | C2C2 | 6.7E-13 | 48.4 |
| TRINITY_DN49043_c0_g5 | PF00643 | zf-B_box | B-box zinc finger | C2C2 | 0.000025 | 24.2 |
| TRINITY_DN43190_c0_g1 | PF00643 | zf-B_box | B-box zinc finger | C2C2 | 0.000057 | 23.1 |
| TRINITY_DN60332_c2_g2 | PF00643 | zf-B_box | B-box zinc finger | C2C2 | 0.000092 | 22.4 |
| TRINITY_DN14502_c0_g1 | PF00643 | zf-B_box | B-box zinc finger | C2C2 | 7.3E-14 | 51.5 |
| TRINITY_DN50387_c5_g1 | PF00643 | zf-B_box | B-box zinc finger | C2C2 | 0.00014 | 21.8 |
| TRINITY_DN52528_c0_g1 | PF00643 | zf-B_box | B-box zinc finger | C2C2 | 0.00046 | 20.1 |
| TRINITY_DN44011_c0_g1 | PF00643 | zf-B_box | B-box zinc finger | C2C2 | 0.00000019 | 31 |
| TRINITY_DN44011_c0_g2 | PF00643 | zf-B_box | B-box zinc finger | C2C2 | 0.00000032 | 30.2 |
| TRINITY_DN52467_c6_g1 | PF00643 | zf-B_box | B-box zinc finger | C2C2 | 0.00000067 | 29.2 |
| TRINITY_DN74819_c1_g4 | PF03638 | TCR | Tesmin/TSO1-like CXC domain, cysteine-rich domain | CPP | 6.7E-21 | 74.2 |
| TRINITY_DN74819_c1_g1 | PF03638 | TCR | Tesmin/TSO1-like CXC domain, cysteine-rich domain | CPP | 7E-23 | 80.5 |
| TRINITY_DN39835_c0_g1 | PF03638 | TCR | Tesmin/TSO1-like CXC domain, cysteine-rich domain | CPP | 7.8E-25 | 86.8 |
| TRINITY_DN61689_c0_g7 | PF03638 | TCR | Tesmin/TSO1-like CXC domain, cysteine-rich domain | CPP | 8.2E-24 | 83.5 |
| TRINITY_DN52872_c4_g1 | PF03638 | TCR | Tesmin/TSO1-like CXC domain, cysteine-rich domain | CPP | 1.2E-22 | 79.7 |
| TRINITY_DN62316_c1_g7 | PF03638 | TCR | Tesmin/TSO1-like CXC domain, cysteine-rich domain | CPP | 0.00099 | 19.2 |
| TRINITY_DN55496_c2_g2 | PF02701 | Zf-Dof | Dof domain, zinc finger | C2C2 | 4.2E-31 | 106.7 |
| TRINITY_DN51761_c0_g2 | PF02701 | Zf-Dof | Dof domain, zinc finger | C2C2 | 1.3E-20 | 73.1 |
| TRINITY_DN51761_c0_g3 | PF02701 | Zf-Dof | Dof domain, zinc finger | C2C2 | 5.9E-32 | 109.5 |
| TRINITY_DN46650_c0_g5 | PF02701 | Zf-Dof | Dof domain, zinc finger | C2C2 | 3.2E-31 | 107.1 |
| TRINITY_DN46650_c0_g6 | PF02701 | Zf-Dof | Dof domain, zinc finger | C2C2 | 7.3E-33 | 112.4 |
| TRINITY_DN46650_c0_g3 | PF02701 | Zf-Dof | Dof domain, zinc finger | C2C2 | 2.5E-32 | 110.7 |
| TRINITY_DN46650_c0_g8 | PF02701 | Zf-Dof | Dof domain, zinc finger | C2C2 | 3.7E-32 | 110.1 |
| TRINITY_DN46650_c0_g1 | PF02701 | Zf-Dof | Dof domain, zinc finger | C2C2 | 1.1E-32 | 111.8 |
| TRINITY_DN46650_c0_g9 | PF02701 | Zf-Dof | Dof domain, zinc finger | C2C2 | 1.3E-33 | 114.8 |
| TRINITY_DN45464_c0_g1 | PF02701 | Zf-Dof | Dof domain, zinc finger | C2C2 | 3.3E-33 | 113.5 |
| TRINITY_DN45464_c0_g3 | PF02701 | Zf-Dof | Dof domain, zinc finger | C2C2 | 1E-32 | 111.9 |
| TRINITY_DN45464_c0_g2 | PF02701 | Zf-Dof | Dof domain, zinc finger | C2C2 | 3.2E-33 | 113.5 |
| TRINITY_DN60997_c3_g1 | PF02701 | Zf-Dof | Dof domain, zinc finger | C2C2 | 1.8E-31 | 107.9 |
| TRINITY_DN50531_c3_g5 | PF02701 | Zf-Dof | Dof domain, zinc finger | C2C2 | 6.6E-33 | 112.5 |
| TRINITY_DN74067_c0_g2 | PF02701 | Zf-Dof | Dof domain, zinc finger | C2C2 | 1E-32 | 111.9 |
| TRINITY_DN74067_c0_g1 | PF02701 | Zf-Dof | Dof domain, zinc finger | C2C2 | 6.4E-17 | 61.3 |
| TRINITY_DN24657_c0_g1 | PF02701 | Zf-Dof | Dof domain, zinc finger | C2C2 | 8.8E-32 | 108.9 |
| TRINITY_DN46762_c3_g1 | PF02701 | Zf-Dof | Dof domain, zinc finger | C2C2 | 8.3E-32 | 109 |
| TRINITY_DN69059_c1_g5 | PF02701 | Zf-Dof | Dof domain, zinc finger | C2C2 | 5.3E-21 | 74.4 |
| TRINITY_DN42192_c0_g1 | PF04690 | YABBY | YABBY protein | C2C2 | 2.2E-52 | 177.8 |
| TRINITY_DN42130_c0_g1 | PF04690 | YABBY | YABBY protein | C2C2 | 9.2E-30 | 104.2 |
| TRINITY_DN42130_c0_g1 | PF04690 | YABBY | YABBY protein | C2C2 | 2.8E-27 | 96.1 |
| TRINITY_DN55709_c0_g1 | PF04690 | YABBY | YABBY protein | C2C2 | 2.3E-64 | 216.7 |
| TRINITY_DN50143_c2_g2 | PF04689 | S1FA | DNA binding protein S1FA | S1Fa-like | 1.7E-38 | 130.4 |
| TRINITY_DN40244_c0_g2 | PF08536 | Whirly | Whirly transcription factor | Whirly | 1.1E-16 | 60.8 |
| TRINITY_DN40244_c0_g1 | PF08536 | Whirly | Whirly transcription factor | Whirly | 4.4E-59 | 198 |
| TRINITY_DN74136_c6_g1 | PF08536 | Whirly | Whirly transcription factor | Whirly | 0.00059 | 19.5 |
| TRINITY_DN65808_c4_g1 | PF08536 | Whirly | Whirly transcription factor | Whirly | 0.000046 | 23.1 |
| TRINITY_DN47772_c0_g4 | PF04770 | ZF-HD_dimer | ZF-HD protein dimerisation region | ZF-HD | 2.2E-28 | 98.2 |
| TRINITY_DN47772_c0_g5 | PF04770 | ZF-HD_dimer | ZF-HD protein dimerisation region | ZF-HD | 2.4E-30 | 104.5 |
| TRINITY_DN47772_c0_g3 | PF04770 | ZF-HD_dimer | ZF-HD protein dimerisation region | ZF-HD | 8.4E-30 | 102.7 |
| TRINITY_DN47772_c0_g6 | PF04770 | ZF-HD_dimer | ZF-HD protein dimerisation region | ZF-HD | 6.9E-30 | 103 |
| TRINITY_DN65693_c2_g1 | PF04770 | ZF-HD_dimer | ZF-HD protein dimerisation region | ZF-HD | 1.2E-27 | 95.8 |
| TRINITY_DN74316_c1_g1 | PF04770 | ZF-HD_dimer | ZF-HD protein dimerisation region | ZF-HD | 4.3E-24 | 84.5 |
| TRINITY_DN49113_c0_g4 | PF04770 | ZF-HD_dimer | ZF-HD protein dimerisation region | ZF-HD | 2.8E-29 | 101.1 |
| TRINITY_DN49113_c0_g5 | PF04770 | ZF-HD_dimer | ZF-HD protein dimerisation region | ZF-HD | 1.2E-29 | 102.2 |
| TRINITY_DN49113_c0_g6 | PF04770 | ZF-HD_dimer | ZF-HD protein dimerisation region | ZF-HD | 6.7E-29 | 99.9 |
| TRINITY_DN74189_c0_g6 | PF04770 | ZF-HD_dimer | ZF-HD protein dimerisation region | ZF-HD | 2.5E-25 | 88.4 |
| TRINITY_DN60598_c4_g2 | PF06943 | Zf-LSD1 | LSD1 zinc finger | C2C2 | 6E-26 | 89.8 |
| TRINITY_DN60598_c4_g3 | PF06943 | Zf-LSD1 | LSD1 zinc finger | C2C2 | 4.7E-35 | 118.9 |
| TRINITY_DN60984_c0_g1 | PF06943 | Zf-LSD1 | LSD1 zinc finger | C2C2 | 0.000063 | 22.6 |
| TRINITY_DN62461_c1_g13 | PF06943 | Zf-LSD1 | LSD1 zinc finger | C2C2 | 0.0002 | 21 |
| TRINITY_DN72308_c2_g1 | PF06943 | Zf-LSD1 | LSD1 zinc finger | C2C2 | 2.4E-19 | 68.7 |
| TRINITY_DN72308_c2_g3 | PF06943 | Zf-LSD1 | LSD1 zinc finger | C2C2 | 0.0006 | 19.5 |
| TRINITY_DN60467_c4_g1 | PF06943 | Zf-LSD1 | LSD1 zinc finger | C2C2 | 5.1E-26 | 90 |
| TRINITY_DN44901_c4_g2 | PF06943 | Zf-LSD1 | LSD1 zinc finger | C2C2 | 7E-11 | 41.6 |
| TRINITY_DN57490_c1_g1 | PF06943 | Zf-LSD1 | LSD1 zinc finger | C2C2 | 0.000000008 | 35.1 |
| TRINITY_DN48522_c6_g1 | PF05687 | DUF822 | BES1/BZR1 plant transcription factor, N-terminal | BES1 | 3E-45 | 154.2 |
| TRINITY_DN51670_c0_g1 | PF05687 | DUF822 | BES1/BZR1 plant transcription factor, N-terminal | BES1 | 7.2E-10 | 39.3 |
| TRINITY_DN51670_c0_g2 | PF05687 | DUF822 | BES1/BZR1 plant transcription factor, N-terminal | BES1 | 4.7E-60 | 202.2 |
| TRINITY_DN51670_c1_g5 | PF05687 | DUF822 | BES1/BZR1 plant transcription factor, N-terminal | BES1 | 1.5E-61 | 207.1 |
| TRINITY_DN58690_c0_g1 | PF05687 | DUF822 | BES1/BZR1 plant transcription factor, N-terminal | BES1 | 6.3E-35 | 120.7 |
| TRINITY_DN57072_c1_g2 | PF01698 | FLO_LFY | Floricaula / Leafy protein | LFY | 1.3E-182 | 607.1 |
| TRINITY_DN57072_c1_g5 | PF01698 | FLO_LFY | Floricaula / Leafy protein | LFY | 5.5E-09 | 35.6 |

**Supplementary Table 5.** Module Component Details

| **gene id** | **NR description** | **module** | **kME** |
| --- | --- | --- | --- |
| TRINITY_DN58494_c2_g1 | XP_008788762.1(PREDICTED: B3 domain-containing protein Os02g0683500-like [Phoenix dactylifera]) | blue | 0.61839 |
| TRINITY_DN47961_c0_g1 | AAD56411.1(bHLH transcription factor GBOF-1 [Tulipa gesneriana]) | blue | 0.6672 |
| TRINITY_DN48538_c1_g1 | XP_009397201.1(PREDICTED: ethylene-responsive transcription factor 12-like [Musa acuminata subsp. malaccensis]) | blue | 0.67438 |
| TRINITY_DN48655_c0_g1 | XP_020691884.1(transcription factor-like protein DPB isoform X1 [Dendrobium catenatum]) | blue | 0.50819 |
| TRINITY_DN49234_c2_g2 | XP_008782655.1(PREDICTED: transcription factor RF2b-like [Phoenix dactylifera]) | blue | 0.68865 |
| TRINITY_DN50381_c3_g1 | XP_009399863.1(PREDICTED: transcription factor PCL1-like [Musa acuminata subsp. malaccensis]) | blue | 0.40629 |
| TRINITY_DN52387_c8_g1 | XP_010929825.1(PREDICTED: zinc finger CCCH domain-containing protein ZFN-like isoform X1 [Elaeis guineensis]) | blue | 0.69782 |
| TRINITY_DN57695_c1_g2 | XP_009406251.1(PREDICTED: basic leucine zipper 61-like [Musa acuminata subsp. malaccensis]) | blue | 0.58343 |
| TRINITY_DN59349_c1_g1 |  | blue | 0.54913 |
| TRINITY_DN61808_c1_g2 | XP_008787890.1(PREDICTED: protein Barley B recombinant-like [Phoenix dactylifera]) | blue | 0.84836 |
| TRINITY_DN62113_c0_g1 | BAU68653.1(R2R3-MYB transcriptional factor, partial [Lilium hybrid division I]) | blue | 0.80861 |
| TRINITY_DN63282_c4_g1 | XP_008812058.1(PREDICTED: GATA transcription factor 2-like [Phoenix dactylifera]) | blue | 0.79935 |
| TRINITY_DN70062_c1_g3 | XP_010923489.1(PREDICTED: light-inducible protein CPRF2-like [Elaeis guineensis]) | blue | 0.4328 |
| TRINITY_DN56139_c2_g2 | AHY82574.1(MADS-box protein 7 [Lilium formosanum]) | blue | 0.5204 |
| TRINITY_DN54160_c4_g1 | XP_015901649.1(PREDICTED: uncharacterized protein LOC107434675 isoform X1 [Ziziphus jujuba]) | blue | 0.7345 |
| TRINITY_DN36237_c0_g1 | XP_020251321.1(ethylene-responsive transcription factor WIN1 [Asparagus officinalis]) | blue | 0.91444 |
| TRINITY_DN36993_c0_g1 | XP_010932049.1(PREDICTED: transcription factor SPATULA [Elaeis guineensis]) | blue | 0.93993 |
| TRINITY_DN38127_c0_g1 | XP_010938177.1(PREDICTED: zinc finger CCCH domain-containing protein 3 isoform X1 [Elaeis guineensis]) | blue | 0.84744 |
| TRINITY_DN42064_c0_g1 | XP_020274488.1(transcription factor ILI3-like [Asparagus officinalis]) | blue | 0.80908 |
| TRINITY_DN42130_c0_g1 | XP_008787013.1(PREDICTED: protein YABBY 4-like [Phoenix dactylifera]) | blue | 0.84518 |
| TRINITY_DN42741_c0_g1 | AQR58153.1(SPL2, partial [Tulipa gesneriana]) | blue | 0.87915 |
| TRINITY_DN43190_c0_g1 | XP_017696909.1(PREDICTED: B-box zinc finger protein 32-like [Phoenix dactylifera]) | blue | 0.60879 |
| TRINITY_DN44011_c0_g1 | XP_022775975.1(zinc finger protein CONSTANS-LIKE 16-like [Durio zibethinus]) | blue | 0.85098 |
| TRINITY_DN44153_c0_g1 | PSR93425.1(Heat stress transcription factor B-2a like [Actinidia chinensis var. chinensis]) | blue | 0.48904 |
| TRINITY_DN44348_c0_g1 | XP_018678966.1(PREDICTED: squamosa promoter-binding-like protein 10 isoform X1 [Musa acuminata subsp. malaccensis]) | blue | 0.92945 |
| TRINITY_DN44570_c0_g1 | XP_010909195.1(PREDICTED: NAC domain-containing protein 83 [Elaeis guineensis]) | blue | 0.88969 |
| TRINITY_DN44964_c0_g1 | XP_008811822.1(PREDICTED: transcription factor PIF4-like isoform X2 [Phoenix dactylifera]) | blue | 0.93153 |
| TRINITY_DN45166_c0_g2 | XP_009418273.1(PREDICTED: transcription factor DIVARICATA-like [Musa acuminata subsp. malaccensis]) | blue | 0.93861 |
| TRINITY_DN45177_c0_g1 | XP_010939970.1(PREDICTED: protein Barley B recombinant-like [Elaeis guineensis]) | blue | 0.90257 |
| TRINITY_DN45464_c0_g1 | XP_017700540.1(PREDICTED: dof zinc finger protein DOF5.1-like isoform X2 [Phoenix dactylifera]) | blue | 0.59535 |
| TRINITY_DN45464_c0_g2 | XP_008802760.1(PREDICTED: dof zinc finger protein DOF5.1-like isoform X1 [Phoenix dactylifera]) | blue | 0.85647 |
| TRINITY_DN45813_c0_g1 | XP_008803818.2(PREDICTED: ethylene-responsive transcription factor ERF038-like [Phoenix dactylifera]) | blue | 0.90304 |
| TRINITY_DN45950_c0_g2 | XP_010922322.1(PREDICTED: transcription factor AS1-like [Elaeis guineensis]) | blue | 0.83272 |
| TRINITY_DN46300_c1_g1 | XP_017700511.1(PREDICTED: myb-related protein 2-like isoform X1 [Phoenix dactylifera]) | blue | 0.41041 |
| TRINITY_DN46300_c1_g2 | XP_006585391.1(PREDICTED: myb family transcription factor APL-like isoform X1 [Glycine max]) | blue | 0.84623 |
| TRINITY_DN46621_c0_g1 | XP_008788501.1(PREDICTED: transcription factor bHLH106-like [Phoenix dactylifera]) | blue | 0.76313 |
| TRINITY_DN46650_c0_g8 | XP_018684472.1(PREDICTED: dof zinc finger protein DOF3.1-like [Musa acuminata subsp. malaccensis]) | blue | 0.54907 |
| TRINITY_DN46675_c1_g2 | XP_020258746.1(protein FAR1-RELATED SEQUENCE 5-like [Asparagus officinalis]) | blue | 0.82015 |
| TRINITY_DN47087_c3_g2 | PKU80315.1(Putative NAC domain-containing protein 94 [Dendrobium catenatum]) | blue | 0.91282 |
| TRINITY_DN47309_c1_g6 | XP_010942127.1(PREDICTED: two-component response regulator ORR22-like isoform X1 [Elaeis guineensis]) | blue | 0.86463 |
| TRINITY_DN47735_c4_g1 | XP_010919684.2(PREDICTED: B3 domain-containing protein Os03g0212300-like isoform X2 [Elaeis guineensis]) | blue | 0.79288 |
| TRINITY_DN48142_c4_g1 | XP_010922938.1(PREDICTED: ABSCISIC ACID-INSENSITIVE 5-like protein 2 [Elaeis guineensis]) | blue | 0.55592 |
| TRINITY_DN48186_c0_g1 | XP_010926223.1(PREDICTED: myb-like DNA-binding protein BAS1 isoform X1 [Elaeis guineensis]) | blue | 0.90104 |
| TRINITY_DN48655_c1_g1 | XP_010241817.1(PREDICTED: transcription factor-like protein DPB [Nelumbo nucifera]) | blue | 0.8786 |
| TRINITY_DN48855_c2_g1 | XP_010923284.1(PREDICTED: GATA transcription factor 18-like [Elaeis guineensis]) | blue | 0.83483 |
| TRINITY_DN49113_c0_g4 | XP_010930367.1(PREDICTED: zinc-finger homeodomain protein 4-like [Elaeis guineensis]) | blue | 0.96408 |
| TRINITY_DN50577_c1_g3 | XP_017696717.1(PREDICTED: NAC domain-containing protein 37-like [Phoenix dactylifera]) | blue | 0.9112 |
| TRINITY_DN50736_c4_g1 | XP_008789607.1(PREDICTED: squamosa promoter-binding-like protein 3 [Phoenix dactylifera]) | blue | 0.89947 |
| TRINITY_DN51869_c1_g1 | BAB91550.1(MADS-box transcription factor [Lilium regale]) | blue | 0.94253 |
| TRINITY_DN52314_c3_g1 | XP_009409645.2(PREDICTED: transcription factor bHLH30-like [Musa acuminata subsp. malaccensis]) | blue | 0.90426 |
| TRINITY_DN52332_c1_g5 | XP_008799483.1(PREDICTED: transcription factor bHLH35-like isoform X1 [Phoenix dactylifera]) | blue | 0.94135 |
| TRINITY_DN52814_c1_g1 | PKA55894.1(Telomere repeat-binding factor 4 [Apostasia shenzhenica]) | blue | 0.9275 |
| TRINITY_DN52872_c4_g1 | XP_010923540.1(PREDICTED: protein tesmin/TSO1-like CXC 3 [Elaeis guineensis]) | blue | 0.86141 |
| TRINITY_DN53211_c3_g1 | PIA64055.1(hypothetical protein AQUCO_00201389v1 [Aquilegia coerulea]) | blue | 0.81537 |
| TRINITY_DN53475_c0_g2 | XP_008786460.1(PREDICTED: auxin response factor 7-like [Phoenix dactylifera]) | blue | 0.91013 |
| TRINITY_DN53627_c4_g1 | XP_008805470.1(PREDICTED: zinc finger CCCH domain-containing protein 31-like [Phoenix dactylifera]) | blue | 0.87549 |
| TRINITY_DN54094_c6_g1 | XP_010924666.1(PREDICTED: B3 domain-containing protein Os01g0723500 [Elaeis guineensis]) | blue | 0.86148 |
| TRINITY_DN54094_c6_g6 | XP_010924666.1(PREDICTED: B3 domain-containing protein Os01g0723500 [Elaeis guineensis]) | blue | 0.65042 |
| TRINITY_DN55049_c4_g2 | XP_010942229.1(PREDICTED: putative B3 domain-containing protein Os06g0632500 [Elaeis guineensis]) | blue | 0.90835 |
| TRINITY_DN55482_c1_g2 | BAM71806.1(R2R3-MYB transcription factor [Gentiana triflora]) | blue | 0.88579 |
| TRINITY_DN55858_c3_g1 | PKA53235.1(Protein basic pentacysteine 7 [Apostasia shenzhenica]) | blue | 0.98147 |
| TRINITY_DN55923_c0_g2 | XP_010936746.2(PREDICTED: uncharacterized protein LOC105056299 [Elaeis guineensis]) | blue | 0.3559 |
| TRINITY_DN55930_c2_g2 | XP_011081287.1(transcription factor MYBS3 [Sesamum indicum]) | blue | 0.33763 |
| TRINITY_DN56007_c2_g2 | XP_010905576.1(PREDICTED: B3 domain-containing protein Os07g0563300-like isoform X1 [Elaeis guineensis]) | blue | 0.72701 |
| TRINITY_DN56295_c2_g1 | XP_009400270.1(PREDICTED: myb-related protein Hv1-like isoform X2 [Musa acuminata subsp. malaccensis]) | blue | 0.94991 |
| TRINITY_DN56646_c2_g9 | BAU68656.1(R2R3-MYB transcriptional factor, partial [Lilium hybrid division I]) | blue | 0.92562 |
| TRINITY_DN56716_c0_g1 | XP_010933331.1(PREDICTED: probable ADP-ribosylation factor GTPase-activating protein AGD14 isoform X2 [Elaeis guineensis]) | blue | 0.83609 |
| TRINITY_DN56909_c2_g1 | APY23909.1(APETALA 2-like protein, partial [Lilium formosanum x Lilium longiflorum]) | blue | 0.94203 |
| TRINITY_DN56909_c2_g4 | CBI21387.3(unnamed protein product, partial [Vitis vinifera]) | blue | 0.85586 |
| TRINITY_DN57072_c1_g2 | BBC44063.1(floricaula/leafy protein [Lilium formosanum x Lilium longiflorum]) | blue | 0.79817 |
| TRINITY_DN58023_c2_g2 | XP_010249637.1(PREDICTED: transcription factor TCP7 [Nelumbo nucifera]) | blue | 0.89554 |
| TRINITY_DN58351_c1_g1 | XP_008795752.1(PREDICTED: squamosa promoter-binding-like protein 14 isoform X2 [Phoenix dactylifera]) | blue | 0.84913 |
| TRINITY_DN58423_c2_g3 | XP_010919983.1(PREDICTED: probable transcription factor KAN2 isoform X1 [Elaeis guineensis]) | blue | 0.82851 |
| TRINITY_DN59676_c2_g2 | BAU29931.1(transcription factor basic helix-loop-helix [Lilium regale]) | blue | 0.62115 |
| TRINITY_DN59889_c5_g1 | XP_020100432.1(homeobox-leucine zipper protein ROC2-like [Ananas comosus]) | blue | 0.72998 |
| TRINITY_DN60467_c4_g1 | XP_009590862.1(PREDICTED: protein LOL1 isoform X1 [Nicotiana tomentosiformis]) | blue | 0.91473 |
| TRINITY_DN60522_c1_g1 | XP_010906815.1(PREDICTED: transcription factor PIF4-like isoform X2 [Elaeis guineensis]) | blue | 0.82157 |
| TRINITY_DN60560_c2_g1 | PKU76346.1(Myb-related protein Myb4 [Dendrobium catenatum]) | blue | 0.92715 |
| TRINITY_DN60612_c2_g2 | XP_017702270.1(PREDICTED: two-component response regulator ORR21-like isoform X1 [Phoenix dactylifera]) | blue | 0.67982 |
| TRINITY_DN60721_c2_g1 | XP_010939817.1(PREDICTED: agamous-like MADS-box protein AGL65 isoform X2 [Elaeis guineensis]) | blue | 0.59623 |
| TRINITY_DN61123_c0_g1 | XP_010904660.1(PREDICTED: transcription factor PIF4 isoform X7 [Elaeis guineensis]) | blue | 0.82149 |
| TRINITY_DN61808_c1_g1 | XP_010919161.1(PREDICTED: barley B recombinant-like protein D [Elaeis guineensis]) | blue | 0.71085 |
| TRINITY_DN61860_c0_g1 | XP_019706299.1(PREDICTED: zinc finger CCCH domain-containing protein 53-like isoform X2 [Elaeis guineensis]) | blue | 0.98249 |
| TRINITY_DN62078_c2_g2 | XP_010921536.1(PREDICTED: myb-related protein P [Elaeis guineensis]) | blue | 0.90029 |
| TRINITY_DN62080_c0_g5 | XP_020685544.1(ethylene-responsive transcription factor ERF061-like [Dendrobium catenatum]) | blue | 0.80635 |
| TRINITY_DN62316_c1_g7 | XP_017699905.1(PREDICTED: histone-lysine N-methyltransferase EZ1-like isoform X3 [Phoenix dactylifera]) | blue | 0.80097 |
| TRINITY_DN62882_c0_g4 | AIU39031.1(myb transcription factor 2, partial [Narcissus tazetta var. chinensis]) | blue | 0.8378 |
| TRINITY_DN63282_c4_g2 | XP_010910788.1(PREDICTED: GATA transcription factor 2-like [Elaeis guineensis]) | blue | 0.93832 |
| TRINITY_DN63368_c3_g1 | XP_009411369.1(PREDICTED: GATA transcription factor 1-like [Musa acuminata subsp. malaccensis]) | blue | 0.901 |
| TRINITY_DN63596_c0_g1 | XP_008780035.1(PREDICTED: growth-regulating factor 10-like [Phoenix dactylifera]) | blue | 0.84253 |
| TRINITY_DN63875_c8_g1 | XP_010915216.1(PREDICTED: putative GATA transcription factor 22 [Elaeis guineensis]) | blue | 0.82049 |
| TRINITY_DN64527_c1_g3 | XP_020517880.1(uncharacterized zinc finger protein At4g06634 isoform X2 [Amborella trichopoda]) | blue | 0.67303 |
| TRINITY_DN64552_c3_g4 | XP_019701995.1(PREDICTED: LOB domain-containing protein 36 [Elaeis guineensis]) | blue | 0.6265 |
| TRINITY_DN64973_c0_g1 | XP_010913902.1(PREDICTED: zinc finger CCCH domain-containing protein 31 [Elaeis guineensis]) | blue | 0.89433 |
| TRINITY_DN65102_c0_g1 | XP_019708739.1(PREDICTED: homeobox-leucine zipper protein ROC8-like [Elaeis guineensis]) | blue | 0.81063 |
| TRINITY_DN65554_c1_g6 | XP_020260871.1(NAC domain-containing protein 86-like [Asparagus officinalis]) | blue | 0.89616 |
| TRINITY_DN65610_c0_g3 | XP_010927740.1(PREDICTED: squamosa promoter-binding-like protein 16 [Elaeis guineensis]) | blue | 0.92641 |
| TRINITY_DN65829_c1_g2 | XP_009401828.1(PREDICTED: uncharacterized protein LOC103985746 isoform X1 [Musa acuminata subsp. malaccensis]) | blue | 0.92702 |
| TRINITY_DN65829_c1_g3 | XP_022880555.1(uncharacterized protein LOC111397810 [Olea europaea var. sylvestris]) | blue | 0.85261 |
| TRINITY_DN65846_c0_g1 | XP_008796561.1(PREDICTED: probable WRKY transcription factor 2 isoform X4 [Phoenix dactylifera]) | blue | 0.68189 |
| TRINITY_DN67273_c1_g1 | XP_010916517.1(PREDICTED: DELLA protein SLR1 isoform X1 [Elaeis guineensis]) | blue | 0.84974 |
| TRINITY_DN67657_c5_g4 | XP_020102157.1(B3 domain-containing protein Os05g0481400 [Ananas comosus]) | blue | 0.77053 |
| TRINITY_DN67755_c0_g1 | XP_020570577.1(GATA transcription factor 4-like [Phalaenopsis equestris]) | blue | 0.93897 |
| TRINITY_DN67854_c0_g2 | XP_010927010.1(PREDICTED: AP2-like ethylene-responsive transcription factor At2g41710 isoform X2 [Elaeis guineensis]) | blue | 0.87516 |
| TRINITY_DN68432_c4_g1 | PKA52554.1(G-box-binding factor 1 [Apostasia shenzhenica]) | blue | 0.60029 |
| TRINITY_DN69064_c0_g2 | XP_019707625.1(PREDICTED: transcription factor bHLH35 isoform X2 [Elaeis guineensis]) | blue | 0.85589 |
| TRINITY_DN69295_c0_g3 | XP_008811924.1(PREDICTED: auxin response factor 23-like [Phoenix dactylifera]) | blue | 0.92445 |
| TRINITY_DN69514_c2_g1 | XP_020265075.1(zinc finger CCCH domain-containing protein 8 [Asparagus officinalis]) | blue | 0.87082 |
| TRINITY_DN69563_c0_g2 | XP_019709953.1(PREDICTED: nodulation-signaling pathway 1 protein [Elaeis guineensis]) | blue | 0.79583 |
| TRINITY_DN69670_c0_g1 | XP_008804826.1(PREDICTED: F-box/LRR-repeat protein At1g67190-like [Phoenix dactylifera]) | blue | 0.92091 |
| TRINITY_DN70513_c1_g1 | XP_008801848.1(PREDICTED: squamosa promoter-binding-like protein 16 [Phoenix dactylifera]) | blue | 0.95121 |
| TRINITY_DN72801_c1_g1 | XP_017700050.1(PREDICTED: transcription factor E2FB-like [Phoenix dactylifera]) | blue | 0.63319 |
| TRINITY_DN73211_c2_g2 | XP_010923678.1(PREDICTED: homeobox-leucine zipper protein HOX32 [Elaeis guineensis]) | blue | 0.87282 |
| TRINITY_DN74189_c0_g6 | XP_010925945.1(PREDICTED: LOW QUALITY PROTEIN: zinc-finger homeodomain protein 6-like [Elaeis guineensis]) | blue | 0.90032 |
| TRINITY_DN42110_c0_g1 | XP_010905080.1(PREDICTED: scarecrow-like protein 15 [Elaeis guineensis]) | brown | 0.54426 |
| TRINITY_DN44901_c4_g2 | XP_009388277.1(PREDICTED: metacaspase-1 [Musa acuminata subsp. malaccensis]) | brown | 0.54271 |
| TRINITY_DN48751_c3_g1 | XP_010930149.1(PREDICTED: transcription factor bHLH129 isoform X1 [Elaeis guineensis]) | brown | 0.67272 |
| TRINITY_DN50138_c1_g6 | XP_017701969.1(PREDICTED: ethylene-responsive transcription factor ERF113-like isoform X2 [Phoenix dactylifera]) | brown | 0.64786 |
| TRINITY_DN51786_c3_g1 | ABB72843.1(NAC protein 1 splice variant 1 [Elaeis guineensis]) | brown | 0.59895 |
| TRINITY_DN54686_c2_g2 | XP_009412772.1(PREDICTED: homeobox-leucine zipper protein HOX6-like [Musa acuminata subsp. malaccensis]) | brown | 0.32828 |
| TRINITY_DN59829_c1_g4 | XP_010935821.1(PREDICTED: zinc finger protein ZAT5-like [Elaeis guineensis]) | brown | 0.38967 |
| TRINITY_DN60908_c0_g1 | XP_010919575.2(PREDICTED: transcription factor UNE10 [Elaeis guineensis]) | brown | 0.44643 |
| TRINITY_DN61619_c1_g8 | XP_020676729.1(probable WRKY transcription factor 3 [Dendrobium catenatum]) | brown | 0.42614 |
| TRINITY_DN69538_c0_g5 | XP_010915419.1(PREDICTED: dehydration-responsive element-binding protein 3-like [Elaeis guineensis]) | brown | 0.38709 |
| TRINITY_DN70245_c0_g2 | XP_008778535.1(PREDICTED: NAC domain-containing protein 21/22-like [Phoenix dactylifera]) | brown | 0.5153 |
| TRINITY_DN23141_c0_g1 | XP_009411742.1(PREDICTED: scarecrow-like protein 3 [Musa acuminata subsp. malaccensis]) | brown | 0.50982 |
| TRINITY_DN39835_c0_g1 | XP_020273429.1(protein tesmin/TSO1-like CXC 5 isoform X2 [Asparagus officinalis]) | brown | 0.50013 |
| TRINITY_DN41860_c0_g1 | XP_008811550.1(PREDICTED: pathogenesis-related genes transcriptional activator PTI5-like [Phoenix dactylifera]) | brown | 0.7963 |
| TRINITY_DN43446_c0_g1 | XP_020097643.1(auxin response factor 11-like [Ananas comosus]) | brown | 0.39457 |
| TRINITY_DN44662_c0_g1 | XP_010916103.2(PREDICTED: probable WRKY transcription factor 31 [Elaeis guineensis]) | brown | 0.82562 |
| TRINITY_DN44961_c0_g1 | XP_010919736.1(PREDICTED: protein REVEILLE 6 [Elaeis guineensis]) | brown | 0.90835 |
| TRINITY_DN45774_c0_g1 | KHN34067.1(Zinc finger CCCH domain-containing protein 38 [Glycine soja]) | brown | 0.54893 |
| TRINITY_DN49043_c0_g5 | XP_009420790.1(PREDICTED: B-box zinc finger protein 19-like isoform X2 [Musa acuminata subsp. malaccensis]) | brown | 0.82142 |
| TRINITY_DN51786_c2_g1 | XP_010921292.1(PREDICTED: NAC domain-containing protein 100-like [Elaeis guineensis]) | brown | 0.92415 |
| TRINITY_DN51786_c3_g4 | XP_010921289.1(PREDICTED: NAC transcription factor 47-like [Elaeis guineensis]) | brown | 0.83376 |
| TRINITY_DN52195_c0_g1 | XP_010932911.1(PREDICTED: BEL1-like homeodomain protein 7 [Elaeis guineensis]) | brown | 0.71359 |
| TRINITY_DN55033_c3_g1 | XP_002462662.1(transcription factor RF2a [Sorghum bicolor]) | brown | 0.53854 |
| TRINITY_DN56768_c2_g5 | XP_010943207.1(PREDICTED: transcription factor MYB44-like [Elaeis guineensis]) | brown | 0.49437 |
| TRINITY_DN57490_c1_g1 | PKA53303.1(Metacaspase-1 [Apostasia shenzhenica]) | brown | 0.53365 |
| TRINITY_DN57584_c3_g2 | XP_010937215.1(PREDICTED: zinc finger AN1 and C2H2 domain-containing stress-associated protein 16 [Elaeis guineensis]) | brown | 0.52368 |
| TRINITY_DN58195_c1_g4 | XP_008776669.1(PREDICTED: heat stress transcription factor A-4b-like [Phoenix dactylifera]) | brown | 0.79192 |
| TRINITY_DN59706_c0_g2 | XP_020241762.1(transcription factor GAMYB-like [Asparagus officinalis]) | brown | 0.92471 |
| TRINITY_DN60754_c0_g1 | XP_010921141.1(PREDICTED: transcription factor RF2b-like isoform X1 [Elaeis guineensis]) | brown | 0.73953 |
| TRINITY_DN61420_c2_g1 | PKA47010.1(Transcription factor VIP1 [Apostasia shenzhenica]) | brown | 0.81955 |
| TRINITY_DN62017_c1_g2 | XP_009417256.1(PREDICTED: chitin-inducible gibberellin-responsive protein 1-like [Musa acuminata subsp. malaccensis]) | brown | 0.76002 |
| TRINITY_DN63855_c1_g1 | XP_020091023.1(agamous-like MADS-box protein AGL104 [Ananas comosus]) | brown | 0.87484 |
| TRINITY_DN63986_c0_g1 | ONK62414.1(uncharacterized protein A4U43_C07F3620 [Asparagus officinalis]) | brown | 0.73689 |
| TRINITY_DN64343_c2_g1 | XP_009391353.1(PREDICTED: probable LRR receptor-like serine/threonine-protein kinase At1g67720 [Musa acuminata subsp. malaccensis]) | brown | 0.84998 |
| TRINITY_DN64707_c0_g1 | XP_008789086.1(PREDICTED: uncharacterized protein LOC103706673 isoform X1 [Phoenix dactylifera]) | brown | 0.87131 |
| TRINITY_DN64748_c1_g2 | ART33472.1(transcription factor WRKY33 [Lilium regale]) | brown | 0.89671 |
| TRINITY_DN66414_c0_g5 | XP_009411586.1(PREDICTED: bZIP transcription factor 60 [Musa acuminata subsp. malaccensis]) | brown | 0.61237 |
| TRINITY_DN70684_c8_g1 | XP_008781504.1(PREDICTED: uncharacterized protein LOC103701275 [Phoenix dactylifera]) | brown | 0.7059 |
| TRINITY_DN72407_c0_g1 | XP_009406251.1(PREDICTED: basic leucine zipper 61-like [Musa acuminata subsp. malaccensis]) | brown | 0.83923 |
| TRINITY_DN75484_c1_g1 | XP_008807227.1(PREDICTED: homeobox protein BEL1 homolog [Phoenix dactylifera]) | brown | 0.60209 |
| TRINITY_DN72224_c2_g4 | XP_010922970.1(PREDICTED: transcription factor GAMYB isoform X1 [Elaeis guineensis]) | brown | 0.4033 |
| TRINITY_DN43391_c0_g1 | XP_010905085.1(PREDICTED: homeobox-leucine zipper protein HOX12-like [Elaeis guineensis]) | brown | 0.50933 |
| TRINITY_DN47361_c1_g6 | XP_010909715.1(PREDICTED: ethylene-responsive transcription factor 4-like [Elaeis guineensis]) | brown | 0.63358 |
| TRINITY_DN47468_c3_g2 | XP_010914281.1(PREDICTED: myb family transcription factor APL isoform X1 [Elaeis guineensis]) | brown | 0.74577 |
| TRINITY_DN48636_c0_g2 | XP_008800633.1(PREDICTED: zinc finger CCCH domain-containing protein 18-like isoform X2 [Phoenix dactylifera]) | brown | 0.75492 |
| TRINITY_DN50434_c0_g3 | XP_009397634.1(PREDICTED: GATA transcription factor 5-like [Musa acuminata subsp. malaccensis]) | brown | 0.56015 |
| TRINITY_DN50492_c2_g2 | XP_010914176.1(PREDICTED: protein KAKU4 isoform X2 [Elaeis guineensis]) | brown | 0.52066 |
| TRINITY_DN52006_c1_g1 | XP_008788364.1(PREDICTED: transcription factor bHLH121 isoform X2 [Phoenix dactylifera]) | brown | 0.58446 |
| TRINITY_DN53960_c5_g1 | XP_020248378.1(probable transcription factor At5g61620 [Asparagus officinalis]) | brown | 0.61243 |
| TRINITY_DN54160_c5_g2 | PSS11743.1(PKS-NRPS hybrid synthetase [Actinidia chinensis var. chinensis]) | brown | 0.55669 |
| TRINITY_DN54856_c1_g1 | XP_019189701.1(PREDICTED: agamous-like MADS-box protein AGL82 [Ipomoea nil]) | brown | 0.89083 |
| TRINITY_DN57395_c1_g5 | XP_022019230.1(ethylene-responsive transcription factor 3-like, partial [Helianthus annuus]) | brown | 0.59451 |
| TRINITY_DN58036_c6_g2 | XP_010921433.1(PREDICTED: probable WRKY transcription factor 48 [Elaeis guineensis]) | brown | 0.65564 |
| TRINITY_DN58424_c1_g1 | XP_010921758.1(PREDICTED: zinc finger CCCH domain-containing protein 6 [Elaeis guineensis]) | brown | 0.80024 |
| TRINITY_DN58576_c2_g2 | XP_010913124.1(PREDICTED: transcription factor bHLH130-like isoform X2 [Elaeis guineensis]) | brown | 0.78323 |
| TRINITY_DN60207_c1_g2 | XP_008808204.1(PREDICTED: E3 ubiquitin-protein ligase makorin isoform X2 [Phoenix dactylifera]) | brown | 0.65805 |
| TRINITY_DN60299_c0_g1 | XP_017698135.1(PREDICTED: transcription factor BIM2 isoform X4 [Phoenix dactylifera]) | brown | 0.66011 |
| TRINITY_DN61268_c0_g2 | XP_010916090.1(PREDICTED: uncharacterized protein LOC105041008 [Elaeis guineensis]) | brown | 0.50888 |
| TRINITY_DN62034_c1_g3 | XP_008784547.1(PREDICTED: cytochrome P450 77A3-like [Phoenix dactylifera]) | brown | 0.65908 |
| TRINITY_DN64528_c0_g1 | XP_010930947.1(PREDICTED: ethylene-responsive transcription factor ABR1 [Elaeis guineensis]) | brown | 0.78942 |
| TRINITY_DN65251_c2_g1 | ACN29102.1(unknown [Zea mays]) | brown | 0.88336 |
| TRINITY_DN65399_c2_g2 | XP_008786234.1(PREDICTED: calmodulin-binding transcription activator 5-like isoform X1 [Phoenix dactylifera]) | brown | 0.75259 |
| TRINITY_DN65897_c0_g1 | XP_010932878.1(PREDICTED: LOW QUALITY PROTEIN: ethylene-responsive transcription factor 3 [Elaeis guineensis]) | brown | 0.79419 |
| TRINITY_DN67151_c5_g1 | XP_020250149.1(homeobox-leucine zipper protein HAT5-like [Asparagus officinalis]) | brown | 0.80401 |
| TRINITY_DN68445_c1_g4 | XP_008800648.1(PREDICTED: LOB domain-containing protein 38-like [Phoenix dactylifera]) | brown | 0.76527 |
| TRINITY_DN68856_c1_g1 | XP_018811837.1(PREDICTED: NAC domain-containing protein 83-like [Juglans regia]) | brown | 0.82087 |
| TRINITY_DN69020_c3_g1 | XP_010943317.1(PREDICTED: transcription factor BIM2 isoform X7 [Elaeis guineensis]) | brown | 0.54584 |
| TRINITY_DN69602_c1_g2 | XP_008801311.1(PREDICTED: uncharacterized protein LOC103715464 [Phoenix dactylifera]) | brown | 0.64539 |
| TRINITY_DN70513_c0_g1 | APY23910.1(squamosa promoter-binding-like protein 3, partial [Lilium formosanum x Lilium longiflorum]) | brown | 0.7823 |
| TRINITY_DN71156_c3_g2 | ATL77059.1(transcription factor MYB31, partial [Morus alba]) | brown | 0.67469 |
| TRINITY_DN71755_c1_g1 | OVA02525.1(DNA-binding WRKY [Macleaya cordata]) | brown | 0.90865 |
| TRINITY_DN71942_c0_g1 | AQR58148.1(SOC1-like 1 protein, partial [Tulipa gesneriana]) | brown | 0.57749 |
| TRINITY_DN72442_c0_g2 | OVA09662.1(Myc-type [Macleaya cordata]) | brown | 0.68019 |
| TRINITY_DN74819_c1_g4 | XP_020263972.1(protein tesmin/TSO1-like CXC 5, partial [Asparagus officinalis]) | brown | 0.59914 |
| TRINITY_DN75304_c4_g1 | XP_008784941.1(PREDICTED: uncharacterized protein LOC103703755 isoform X1 [Phoenix dactylifera]) | brown | 0.47281 |
| TRINITY_DN54716_c0_g2 | AQR58148.1(SOC1-like 1 protein, partial [Tulipa gesneriana]) | brown | 0.52853 |
| TRINITY_DN58424_c0_g3 | PON80308.1(Zinc finger, CCCH-type [Parasponia andersonii]) | brown | 0.30566 |
| TRINITY_DN73752_c1_g1 | GAY43377.1(hypothetical protein CUMW_074000 [Citrus unshiu]) | brown | 0.5389 |
| TRINITY_DN71750_c3_g4 | XP_008797493.1(PREDICTED: ETHYLENE INSENSITIVE 3-like 3 protein [Phoenix dactylifera]) | brown | 0.41161 |
| TRINITY_DN73070_c0_g1 | XP_008790783.1(PREDICTED: nuclear transcription factor Y subunit A-1 [Phoenix dactylifera]) | brown | 0.34348 |
| TRINITY_DN50057_c0_g3 | XP_010913077.1(PREDICTED: ethylene-responsive transcription factor-like protein At4g13040 isoform X2 [Elaeis guineensis]) | brown | 0.43459 |
| TRINITY_DN58499_c0_g1 | XP_009382156.1(PREDICTED: uncharacterized protein LOC103970204 isoform X2 [Musa acuminata subsp. malaccensis]) | brown | 0.59209 |
| TRINITY_DN56987_c2_g1 | XP_019708381.1(PREDICTED: protein FAR1-RELATED SEQUENCE 5-like isoform X1 [Elaeis guineensis]) | brown | 0.38997 |
| TRINITY_DN60299_c0_g2 | KMZ58270.1(Transcription factor BIM2 [Zostera marina]) | brown | 0.69721 |
| TRINITY_DN48387_c4_g3 | XP_010926187.1(PREDICTED: putative zinc finger protein CONSTANS-LIKE 11 isoform X2 [Elaeis guineensis]) | brown | 0.38069 |
| TRINITY_DN45078_c0_g1 | ASV46341.1(ethylene insensitive 3, partial [Lilium regale]) | brown | 0.40148 |
| TRINITY_DN50949_c0_g1 | PIA50965.1(hypothetical protein AQUCO_01100050v1 [Aquilegia coerulea]) | grey | 0.41161 |
| TRINITY_DN51278_c0_g2 | KQK87649.1(hypothetical protein SETIT_033903mg [Setaria italica]) | grey | -0.00698 |
| TRINITY_DN70669_c7_g1 | XP_010922889.1(PREDICTED: myb-related protein 2-like [Elaeis guineensis]) | grey | 0.3728 |
| TRINITY_DN48347_c1_g1 | XP_022734373.1(MYB-like transcription factor ETC1 [Durio zibethinus]) | grey | 0.68511 |
| TRINITY_DN64503_c1_g1 | BAM34482.1(MADS-box transcription factor [Alstroemeria ligtu subsp. ligtu]) | grey | 0.4986 |
| TRINITY_DN70069_c1_g1 | BAC77269.2(SCARECROW-like protein [Lilium longiflorum]) | grey | 0.47986 |
| TRINITY_DN75423_c3_g1 | AUX13103.1(heat stress transcription factor A3B [Lilium longiflorum]) | grey | 0.625 |
| TRINITY_DN62034_c1_g2 | XP_020676480.1(G-box-binding factor 1-like isoform X3 [Dendrobium catenatum]) | grey | -0.0758 |
| TRINITY_DN74819_c1_g1 | XP_008793061.1(PREDICTED: protein tesmin/TSO1-like CXC 5 isoform X1 [Phoenix dactylifera]) | grey | 0.00188 |
| TRINITY_DN51950_c0_g1 | XP_008785849.1(PREDICTED: ethylene-responsive transcription factor ERF110-like [Phoenix dactylifera]) | grey | 0.49042 |
| TRINITY_DN66228_c0_g1 | XP_010931909.1(PREDICTED: transcription factor IIIA [Elaeis guineensis]) | grey | 0.21696 |
| TRINITY_DN45791_c0_g1 | XP_018677740.1(PREDICTED: transcription factor FAMA [Musa acuminata subsp. malaccensis]) | grey | 0.0716 |
| TRINITY_DN75902_c3_g2 | XP_008786457.1(PREDICTED: zinc finger CCCH domain-containing protein 16 [Phoenix dactylifera]) | grey | -0.23133 |
| TRINITY_DN47087_c3_g8 | XP_020578930.1(NAC domain-containing protein 68-like [Phalaenopsis equestris]) | grey | 0.35751 |
| TRINITY_DN56491_c3_g2 | XP_020686609.1(BEL1-like homeodomain protein 1 [Dendrobium catenatum]) | grey | 0.55212 |
| TRINITY_DN60638_c1_g2 | APY23916.1(floral homeotic protein APETALA 1, partial [Lilium formosanum x Lilium longiflorum]) | grey | 0.37485 |
| TRINITY_DN70717_c1_g2 | XP_020693801.1(ethylene-responsive transcription factor 2-like [Dendrobium catenatum]) | grey | 0.45329 |
| TRINITY_DN74193_c2_g3 | BAC77269.2(SCARECROW-like protein [Lilium longiflorum]) | grey | 0.38045 |
| TRINITY_DN44307_c0_g1 | XP_010930661.1(PREDICTED: NAC domain-containing protein 104-like [Elaeis guineensis]) | grey | 0.70192 |
| TRINITY_DN53654_c0_g2 | ADM47610.1(heat shock transcription factor A2 [Lilium longiflorum]) | grey | 0.44156 |
| TRINITY_DN55482_c0_g1 | XP_020263794.1(myb-related protein Myb4-like [Asparagus officinalis]) | grey | 0.49242 |
| TRINITY_DN61301_c0_g2 | XP_020200959.1(zinc finger CCCH domain-containing protein 8 isoform X2 [Aegilops tauschii subsp. tauschii]) | grey | 0.71477 |
| TRINITY_DN61823_c1_g1 | XP_006418202.1(ethylene-responsive transcription factor 10 [Eutrema salsugineum]) | grey | 0.79438 |
| TRINITY_DN67846_c3_g1 | XP_009397218.1(PREDICTED: G-box-binding factor 1-like [Musa acuminata subsp. malaccensis]) | grey | 0.72729 |
| TRINITY_DN68105_c0_g2 | XP_008784600.1(PREDICTED: telomere repeat-binding protein 2 [Phoenix dactylifera]) | grey | 0.18529 |
| TRINITY_DN73679_c4_g1 | XP_010929994.1(PREDICTED: zinc finger CCCH domain-containing protein 65 isoform X2 [Elaeis guineensis]) | grey | 0.76092 |
| TRINITY_DN74104_c3_g1 | XP_008800674.1(PREDICTED: protein NLP2-like [Phoenix dactylifera]) | grey | 0.87245 |
| TRINITY_DN49263_c0_g1 | XP_020678302.1(uncharacterized protein LOC110096605 [Dendrobium catenatum]) | grey | -0.37863 |
| TRINITY_DN68762_c0_g1 | XP_010935334.1(PREDICTED: protein CCA1 [Elaeis guineensis]) | grey | 0.64923 |
| TRINITY_DN59282_c0_g2 | XP_008799550.1(PREDICTED: heat stress transcription factor B-2b [Phoenix dactylifera]) | grey | -0.26631 |
| TRINITY_DN72442_c0_g1 | CBI21285.3(unnamed protein product, partial [Vitis vinifera]) | grey | -0.49781 |
| TRINITY_DN54873_c4_g1 | XP_009403789.1(PREDICTED: transcription factor ICE1-like isoform X2 [Musa acuminata subsp. malaccensis]) | grey | -0.64478 |
| TRINITY_DN46929_c0_g10 | XP_010931380.1(PREDICTED: probable WRKY transcription factor 67 [Elaeis guineensis]) | grey | 0.63093 |
| TRINITY_DN56909_c2_g2 | XP_018685325.1(PREDICTED: AP2-like ethylene-responsive transcription factor TOE3 isoform X5 [Musa acuminata subsp. malaccensis]) | grey | 0.10316 |
| TRINITY_DN58218_c0_g1 | XP_009405762.1(PREDICTED: uncharacterized protein LOC103988834 isoform X1 [Musa acuminata subsp. malaccensis]) | grey | -0.10158 |
| TRINITY_DN75474_c2_g1 | ART33469.1(transcription factor MYC2 [Lilium regale]) | grey | 0.14919 |
| TRINITY_DN46762_c3_g1 | XP_010916555.1(PREDICTED: uncharacterized protein LOC105041327 [Elaeis guineensis]) | grey | 0.37363 |
| TRINITY_DN48751_c3_g2 | XP_020253420.1(transcription factor bHLH128-like isoform X1 [Asparagus officinalis]) | grey | 0.44468 |
| TRINITY_DN58195_c1_g1 | XP_008812463.1(PREDICTED: heat stress transcription factor A-1-like [Phoenix dactylifera]) | grey | -0.06904 |
| TRINITY_DN62477_c0_g3 | XP_020883942.1(zinc finger CCCH domain-containing protein 29 [Arabidopsis lyrata subsp. lyrata]) | grey | 0.73809 |
| TRINITY_DN40189_c0_g1 | XP_010914954.1(PREDICTED: protein FAR1-RELATED SEQUENCE 5 [Elaeis guineensis]) | turquoise | 0.88308 |
| TRINITY_DN40244_c0_g1 | XP_010915145.1(PREDICTED: single-stranded DNA-binding protein WHY1, chloroplastic isoform X1 [Elaeis guineensis]) | turquoise | 0.89034 |
| TRINITY_DN43128_c0_g1 | XP_010940136.1(PREDICTED: LOB domain-containing protein 25 [Elaeis guineensis]) | turquoise | 0.30414 |
| TRINITY_DN45519_c0_g2 | XP_010927126.1(PREDICTED: zinc finger CCCH domain-containing protein 46 [Elaeis guineensis]) | turquoise | 0.57451 |
| TRINITY_DN45611_c2_g1 | XP_019710206.1(PREDICTED: auxin response factor 15-like isoform X3 [Elaeis guineensis]) | turquoise | 0.85053 |
| TRINITY_DN46320_c3_g3 | XP_020268251.1(squamosa promoter-binding-like protein 15 isoform X2 [Asparagus officinalis]) | turquoise | 0.77776 |
| TRINITY_DN46575_c0_g1 | XP_010254574.1(PREDICTED: KH domain-containing protein HEN4-like [Nelumbo nucifera]) | turquoise | 0.72269 |
| TRINITY_DN46650_c0_g6 | XP_008806466.2(PREDICTED: dof zinc finger protein DOF1.4-like [Phoenix dactylifera]) | turquoise | 0.56023 |
| TRINITY_DN46650_c0_g9 | XP_010922502.1(PREDICTED: dof zinc finger protein DOF2.1-like [Elaeis guineensis]) | turquoise | 0.43686 |
| TRINITY_DN46675_c1_g1 | ONK76507.1(uncharacterized protein A4U43_C03F28830 [Asparagus officinalis]) | turquoise | 0.88463 |
| TRINITY_DN47127_c1_g3 | XP_019710982.1(PREDICTED: WUSCHEL-related homeobox 8 isoform X4 [Elaeis guineensis]) | turquoise | 0.91892 |
| TRINITY_DN47309_c1_g3 | XP_010942127.1(PREDICTED: two-component response regulator ORR22-like isoform X1 [Elaeis guineensis]) | turquoise | 0.84103 |
| TRINITY_DN47534_c0_g1 | XP_010936695.1(PREDICTED: transcription factor bHLH63 [Elaeis guineensis]) | turquoise | 0.93399 |
| TRINITY_DN47948_c4_g1 | XP_008810645.1(PREDICTED: B3 domain-containing protein Os01g0723500-like [Phoenix dactylifera]) | turquoise | 0.7329 |
| TRINITY_DN48225_c0_g7 | XP_010940077.1(PREDICTED: protein RADIALIS-like 3 [Elaeis guineensis]) | turquoise | 0.70481 |
| TRINITY_DN48522_c6_g1 | XP_008808793.1(PREDICTED: beta-amylase 8 isoform X2 [Phoenix dactylifera]) | turquoise | 0.86714 |
| TRINITY_DN48865_c5_g1 | XP_009390446.1(PREDICTED: GATA transcription factor 20 [Musa acuminata subsp. malaccensis]) | turquoise | 0.7708 |
| TRINITY_DN49099_c0_g1 | XP_008803514.1(PREDICTED: zinc finger CCCH domain-containing protein 57-like isoform X2 [Phoenix dactylifera]) | turquoise | 0.62053 |
| TRINITY_DN49263_c0_g2 | KYP70617.1(hypothetical protein KK1_009838 [Cajanus cajan]) | turquoise | 0.78699 |
| TRINITY_DN49389_c1_g1 | XP_019707314.1(PREDICTED: uncharacterized protein LOC105048021 isoform X2 [Elaeis guineensis]) | turquoise | 0.95189 |
| TRINITY_DN49422_c1_g2 | XP_010918102.1(PREDICTED: myb family transcription factor PHL11 [Elaeis guineensis]) | turquoise | 0.58187 |
| TRINITY_DN49427_c0_g10 | XP_010929108.1(PREDICTED: homeobox protein knotted-1-like 13 [Elaeis guineensis]) | turquoise | 0.93515 |
| TRINITY_DN49855_c0_g2 | XP_008785327.1(PREDICTED: protein FAR1-RELATED SEQUENCE 5-like [Phoenix dactylifera]) | turquoise | 0.85787 |
| TRINITY_DN49923_c0_g1 | XP_008781781.2(PREDICTED: LOW QUALITY PROTEIN: tRNA-dihydrouridine(47) synthase [NAD(P)(+)]-like [Phoenix dactylifera]) | turquoise | 0.96354 |
| TRINITY_DN51168_c5_g1 | XP_020114653.1(telomeric repeat-binding factor 2-like [Ananas comosus]) | turquoise | 0.74317 |
| TRINITY_DN51670_c1_g5 | XP_008795632.1(PREDICTED: protein BZR1 homolog 1 [Phoenix dactylifera]) | turquoise | 0.35281 |
| TRINITY_DN51873_c0_g1 | XP_017701520.1(PREDICTED: SWR1-complex protein 4 [Phoenix dactylifera]) | turquoise | 0.72344 |
| TRINITY_DN52625_c0_g2 | XP_010912055.1(PREDICTED: LOW QUALITY PROTEIN: auxin response factor 17-like [Elaeis guineensis]) | turquoise | 0.88778 |
| TRINITY_DN53142_c2_g1 | XP_010258819.1(PREDICTED: protein FAR1-RELATED SEQUENCE 5-like isoform X1 [Nelumbo nucifera]) | turquoise | 0.78406 |
| TRINITY_DN54228_c1_g3 | XP_010943521.1(PREDICTED: uncharacterized protein LOC105061234 [Elaeis guineensis]) | turquoise | 0.72479 |
| TRINITY_DN54625_c1_g2 | XP_010908697.1(PREDICTED: barley B recombinant-like protein D isoform X1 [Elaeis guineensis]) | turquoise | 0.93088 |
| TRINITY_DN55709_c0_g1 | XP_020255936.1(axial regulator YABBY 5-like isoform X1 [Asparagus officinalis]) | turquoise | 0.84302 |
| TRINITY_DN56107_c1_g5 | PPR98018.1(hypothetical protein GOBAR_AA22649 [Gossypium barbadense]) | turquoise | 0.83748 |
| TRINITY_DN56526_c1_g3 | XP_018852218.1(PREDICTED: protein FAR-RED IMPAIRED RESPONSE 1-like isoform X4 [Juglans regia]) | turquoise | 0.78183 |
| TRINITY_DN56660_c1_g1 | XP_010921790.1(PREDICTED: homeobox protein LUMINIDEPENDENS [Elaeis guineensis]) | turquoise | 0.44804 |
| TRINITY_DN57617_c1_g5 | XP_008788583.1(PREDICTED: basic leucine zipper 23-like [Phoenix dactylifera]) | turquoise | 0.79733 |
| TRINITY_DN58218_c0_g4 | PKA56613.1(Mediator-associated protein 1 [Apostasia shenzhenica]) | turquoise | 0.84983 |
| TRINITY_DN58239_c6_g1 | XP_010924983.1(PREDICTED: zinc finger CCCH domain-containing protein 7 [Elaeis guineensis]) | turquoise | 0.68952 |
| TRINITY_DN58242_c1_g1 | XP_010932445.1(PREDICTED: uncharacterized protein LOC105053107 isoform X5 [Elaeis guineensis]) | turquoise | 0.90457 |
| TRINITY_DN58480_c2_g4 | XP_020274674.1(putative B3 domain-containing protein Os04g0346900 [Asparagus officinalis]) | turquoise | 0.6561 |
| TRINITY_DN58494_c2_g8 | XP_010934908.1(PREDICTED: B3 domain-containing protein Os03g0120900-like [Elaeis guineensis]) | turquoise | 0.44158 |
| TRINITY_DN59551_c1_g2 | XP_008790654.1(PREDICTED: chromatin modification-related protein EAF1 B-like isoform X3 [Phoenix dactylifera]) | turquoise | 0.5982 |
| TRINITY_DN59818_c2_g1 | XP_009384356.1(PREDICTED: protein SCARECROW 2-like [Musa acuminata subsp. malaccensis]) | turquoise | 0.87172 |
| TRINITY_DN60136_c2_g2 | XP_010912762.1(PREDICTED: protein FAR1-RELATED SEQUENCE 6 isoform X2 [Elaeis guineensis]) | turquoise | 0.85943 |
| TRINITY_DN60281_c4_g2 | XP_008799098.1(PREDICTED: zinc finger CCCH domain-containing protein 45-like [Phoenix dactylifera]) | turquoise | 0.9126 |
| TRINITY_DN60997_c3_g1 | XP_010938141.1(PREDICTED: cyclic dof factor 3-like [Elaeis guineensis]) | turquoise | 0.79653 |
| TRINITY_DN61426_c0_g1 | XP_008787331.1(PREDICTED: LOW QUALITY PROTEIN: protein FAR-RED IMPAIRED RESPONSE 1-like [Phoenix dactylifera]) | turquoise | 0.87751 |
| TRINITY_DN61595_c2_g1 | XP_009419759.1(PREDICTED: heat stress transcription factor A-2c-like [Musa acuminata subsp. malaccensis]) | turquoise | 0.70921 |
| TRINITY_DN62080_c0_g1 | XP_010924856.1(PREDICTED: ethylene-responsive transcription factor ERF061 [Elaeis guineensis]) | turquoise | 0.30373 |
| TRINITY_DN62080_c0_g3 | XP_020685544.1(ethylene-responsive transcription factor ERF061-like [Dendrobium catenatum]) | turquoise | 0.55576 |
| TRINITY_DN62099_c2_g1 | XP_010914965.1(PREDICTED: myb family transcription factor EFM [Elaeis guineensis]) | turquoise | 0.38512 |
| TRINITY_DN62882_c1_g1 | XP_020102034.1(transcription factor MYB86-like [Ananas comosus]) | turquoise | 0.62541 |
| TRINITY_DN64279_c1_g7 | PHT56008.1(hypothetical protein CQW23_04494 [Capsicum baccatum]) | turquoise | 0.72211 |
| TRINITY_DN64612_c0_g1 | XP_008810758.1(PREDICTED: transcription factor bHLH82-like isoform X1 [Phoenix dactylifera]) | turquoise | 0.77948 |
| TRINITY_DN64784_c0_g1 | XP_010943353.1(PREDICTED: transcription factor bHLH49 isoform X2 [Elaeis guineensis]) | turquoise | 0.77244 |
| TRINITY_DN64795_c0_g1 | XP_020676049.1(scarecrow-like protein 27 [Dendrobium catenatum]) | turquoise | 0.64773 |
| TRINITY_DN65018_c1_g1 | XP_008793540.1(PREDICTED: uncharacterized protein LOC103709816 isoform X1 [Phoenix dactylifera]) | turquoise | 0.70737 |
| TRINITY_DN65159_c2_g2 | ONK65710.1(uncharacterized protein A4U43_C06F140 [Asparagus officinalis]) | turquoise | 0.8628 |
| TRINITY_DN65386_c2_g3 | XP_019704854.1(PREDICTED: protein ALWAYS EARLY 3-like isoform X2 [Elaeis guineensis]) | turquoise | 0.69664 |
| TRINITY_DN65386_c3_g2 | XP_010931795.1(PREDICTED: protein ALWAYS EARLY 2 isoform X2 [Elaeis guineensis]) | turquoise | 0.8683 |
| TRINITY_DN65424_c1_g1 | XP_010917437.2(PREDICTED: LOW QUALITY PROTEIN: auxin response factor 16 [Elaeis guineensis]) | turquoise | 0.71803 |
| TRINITY_DN65610_c2_g1 | XP_022966801.1(squamosa promoter-binding-like protein 12 isoform X2 [Cucurbita maxima]) | turquoise | 0.89456 |
| TRINITY_DN65663_c5_g1 | XP_020098686.1(protein FAR1-RELATED SEQUENCE 5-like [Ananas comosus]) | turquoise | 0.30207 |
| TRINITY_DN65663_c6_g1 | XP_008809265.1(PREDICTED: protein FAR1-RELATED SEQUENCE 5-like [Phoenix dactylifera]) | turquoise | 0.7733 |
| TRINITY_DN65732_c1_g5 | XP_010930280.1(PREDICTED: protein FAR1-RELATED SEQUENCE 6-like [Elaeis guineensis]) | turquoise | 0.8275 |
| TRINITY_DN66374_c0_g1 | XP_008791527.1(PREDICTED: zinc finger CCCH domain-containing protein 24 [Phoenix dactylifera]) | turquoise | 0.96927 |
| TRINITY_DN66474_c0_g2 | XP_017431439.1(PREDICTED: ATP-dependent zinc metalloprotease FTSH 2, chloroplastic-like [Vigna angularis]) | turquoise | 0.86524 |
| TRINITY_DN66606_c1_g1 | XP_010914262.1(PREDICTED: G-box-binding factor 3-like isoform X1 [Elaeis guineensis]) | turquoise | 0.71632 |
| TRINITY_DN66940_c0_g1 | XP_010942107.1(PREDICTED: transcription factor bHLH66 [Elaeis guineensis]) | turquoise | 0.59558 |
| TRINITY_DN67197_c0_g1 | XP_020572803.1(transcription factor ILI5 [Phalaenopsis equestris]) | turquoise | 0.74257 |
| TRINITY_DN67221_c0_g11 | XP_021813341.1(protein FAR1-RELATED SEQUENCE 5-like [Prunus avium]) | turquoise | 0.64346 |
| TRINITY_DN67221_c0_g2 | XP_021813341.1(protein FAR1-RELATED SEQUENCE 5-like [Prunus avium]) | turquoise | 0.95834 |
| TRINITY_DN67221_c0_g3 | XP_021806714.1(protein FAR1-RELATED SEQUENCE 5-like [Prunus avium]) | turquoise | 0.8885 |
| TRINITY_DN67221_c0_g6 | XP_023872091.1(protein FAR-RED IMPAIRED RESPONSE 1-like [Quercus suber]) | turquoise | 0.84134 |
| TRINITY_DN67665_c3_g3 | XP_010909612.1(PREDICTED: protein FAR1-RELATED SEQUENCE 5-like, partial [Elaeis guineensis]) | turquoise | 0.54734 |
| TRINITY_DN68598_c0_g2 | XP_010940350.1(PREDICTED: lysine-specific demethylase JMJ25 [Elaeis guineensis]) | turquoise | 0.73176 |
| TRINITY_DN69151_c0_g1 | XP_017634928.1(PREDICTED: bZIP transcription factor 11-like [Gossypium arboreum]) | turquoise | 0.34226 |
| TRINITY_DN69637_c4_g1 | XP_008777661.1(PREDICTED: zinc finger CCCH domain-containing protein 36-like isoform X1 [Phoenix dactylifera]) | turquoise | 0.74675 |
| TRINITY_DN69674_c3_g1 | BAU68652.1(R2R3-MYB transcriptional factor, partial [Lilium hybrid division I]) | turquoise | 0.4177 |
| TRINITY_DN69963_c0_g1 | XP_009404687.1(PREDICTED: ETHYLENE INSENSITIVE 3-like 1 protein [Musa acuminata subsp. malaccensis]) | turquoise | 0.74412 |
| TRINITY_DN70039_c0_g2 | XP_010906463.1(PREDICTED: zinc finger CCCH domain-containing protein 59 isoform X1 [Elaeis guineensis]) | turquoise | 0.94391 |
| TRINITY_DN70156_c2_g3 | XP_010914790.1(PREDICTED: probable transcription factor At3g04930 [Elaeis guineensis]) | turquoise | 0.66714 |
| TRINITY_DN70320_c5_g6 | XP_009410295.1(PREDICTED: transcription factor BIM2 isoform X1 [Musa acuminata subsp. malaccensis]) | turquoise | 0.69879 |
| TRINITY_DN70858_c0_g5 | XP_020248224.1(B3 domain-containing protein Os01g0905400-like [Asparagus officinalis]) | turquoise | 0.70257 |
| TRINITY_DN71216_c4_g3 | XP_009407252.1(PREDICTED: protein FAR1-RELATED SEQUENCE 5-like isoform X2 [Musa acuminata subsp. malaccensis]) | turquoise | 0.89077 |
| TRINITY_DN71279_c0_g1 | XP_010918748.1(PREDICTED: AT-rich interactive domain-containing protein 2 [Elaeis guineensis]) | turquoise | 0.88448 |
| TRINITY_DN71569_c1_g1 | XP_010937003.1(PREDICTED: LOW QUALITY PROTEIN: zinc finger CCCH domain-containing protein 24-like [Elaeis guineensis]) | turquoise | 0.5295 |
| TRINITY_DN71703_c0_g4 | XP_010914614.1(PREDICTED: cell division cycle 5-like protein [Elaeis guineensis]) | turquoise | 0.49194 |
| TRINITY_DN72299_c3_g3 | XP_010909461.1(PREDICTED: protein FAR1-RELATED SEQUENCE 5 isoform X1 [Elaeis guineensis]) | turquoise | 0.93165 |
| TRINITY_DN72304_c1_g3 | XP_010941615.1(PREDICTED: protein FAR-RED IMPAIRED RESPONSE 1-like [Elaeis guineensis]) | turquoise | 0.83277 |
| TRINITY_DN7275_c0_g1 | XP_010272971.1(PREDICTED: transcription factor bHLH96 [Nelumbo nucifera]) | turquoise | 0.70985 |
| TRINITY_DN72802_c2_g2 | XP_010915277.1(PREDICTED: protein indeterminate-domain 7 [Elaeis guineensis]) | turquoise | 0.85504 |
| TRINITY_DN72802_c3_g1 | XP_008795616.1(PREDICTED: protein indeterminate-domain 2-like [Phoenix dactylifera]) | turquoise | 0.83804 |
| TRINITY_DN73480_c0_g1 | XP_019709181.1(PREDICTED: putative B3 domain-containing protein Os03g0621600 isoform X1 [Elaeis guineensis]) | turquoise | 0.84272 |
| TRINITY_DN73496_c0_g3 | XP_010275976.1(PREDICTED: lysine-specific demethylase JMJ25-like isoform X3 [Nelumbo nucifera]) | turquoise | 0.82457 |
| TRINITY_DN73900_c0_g2 | XP_010932273.1(PREDICTED: lysine-specific demethylase JMJ705-like [Elaeis guineensis]) | turquoise | 0.8845 |
| TRINITY_DN74058_c0_g2 | XP_023920662.1(auxin response factor 6 [Quercus suber]) | turquoise | 0.89615 |
| TRINITY_DN74508_c0_g4 | XP_010258819.1(PREDICTED: protein FAR1-RELATED SEQUENCE 5-like isoform X1 [Nelumbo nucifera]) | turquoise | 0.6269 |
| TRINITY_DN74973_c3_g1 | APY23911.1(DELLA protein GAI, partial [Lilium formosanum x Lilium longiflorum]) | turquoise | 0.9439 |
| TRINITY_DN75513_c1_g3 | XP_010938389.1(PREDICTED: zinc finger CCCH domain-containing protein 4 [Elaeis guineensis]) | turquoise | 0.80676 |
| TRINITY_DN75569_c1_g2 | XP_017700050.1(PREDICTED: transcription factor E2FB-like [Phoenix dactylifera]) | turquoise | 0.84465 |
| TRINITY_DN76366_c3_g2 | XP_018852218.1(PREDICTED: protein FAR-RED IMPAIRED RESPONSE 1-like isoform X4 [Juglans regia]) | turquoise | 0.63671 |
| TRINITY_DN46690_c0_g1 | XP_008783552.1(PREDICTED: protein NTM1-like 9 [Phoenix dactylifera]) | turquoise | 0.6172 |
| TRINITY_DN52789_c0_g3 | XP_008789333.1(PREDICTED: homeobox-leucine zipper protein GLABRA 2 [Phoenix dactylifera]) | turquoise | 0.32182 |
| TRINITY_DN57411_c0_g1 | XP_008784021.1(PREDICTED: protein FAR1-RELATED SEQUENCE 5-like [Phoenix dactylifera]) | turquoise | 0.51106 |
| TRINITY_DN52768_c0_g1 | XP_008809265.1(PREDICTED: protein FAR1-RELATED SEQUENCE 5-like [Phoenix dactylifera]) | turquoise | 0.33255 |
| TRINITY_DN64602_c2_g1 | XP_009391328.1(PREDICTED: homeobox-leucine zipper protein HOX4-like [Musa acuminata subsp. malaccensis]) | turquoise | 0.42233 |
| TRINITY_DN74067_c0_g1 | XP_010943306.1(PREDICTED: dof zinc finger protein DOF5.4-like [Elaeis guineensis]) | turquoise | 0.50033 |
| TRINITY_DN72316_c5_g5 | XP_008808053.1(PREDICTED: AP2-like ethylene-responsive transcription factor AIL1 [Phoenix dactylifera]) | turquoise | 0.50368 |
| TRINITY_DN75832_c2_g3 | XP_018684782.1(PREDICTED: protein FAR1-RELATED SEQUENCE 5-like isoform X3 [Musa acuminata subsp. malaccensis]) | turquoise | 0.49888 |
| TRINITY_DN91432_c0_g1 | XP_010936712.1(PREDICTED: scarecrow-like protein 23 [Elaeis guineensis]) | turquoise | 0.4047 |
| TRINITY_DN53009_c0_g2 | XP_009417336.1(PREDICTED: cyclin-D-binding Myb-like transcription factor 1 [Musa acuminata subsp. malaccensis]) | turquoise | 0.78653 |
| TRINITY_DN55705_c1_g1 | XP_010240821.1(PREDICTED: transcription factor HY5-like [Nelumbo nucifera]) | turquoise | 0.55816 |
| TRINITY_DN60784_c3_g2 | PKU79258.1(Myb family transcription factor APL [Dendrobium catenatum]) | turquoise | 0.60133 |
| TRINITY_DN61784_c2_g1 | XP_020593729.1(B3 domain-containing protein Os11g0197600-like isoform X2 [Phalaenopsis equestris]) | turquoise | 0.57693 |
| TRINITY_DN63021_c2_g2 | PKA66027.1(Auxin response factor 17 [Apostasia shenzhenica]) | turquoise | 0.60131 |
| TRINITY_DN63742_c0_g1 | PKA54044.1(Protein FD [Apostasia shenzhenica]) | turquoise | 0.50808 |
| TRINITY_DN66228_c0_g3 | XP_008242644.1(PREDICTED: transcription factor IIIA-like [Prunus mume]) | turquoise | 0.30789 |
| TRINITY_DN46171_c2_g6 | XP_010943073.1(PREDICTED: bZIP transcription factor TRAB1 isoform X1 [Elaeis guineensis]) | turquoise | 0.48759 |
| TRINITY_DN47772_c0_g3 | XP_010922896.2(PREDICTED: mini zinc finger protein 2-like [Elaeis guineensis]) | turquoise | 0.43617 |
| TRINITY_DN56716_c0_g2 | XP_009408824.1(PREDICTED: probable ADP-ribosylation factor GTPase-activating protein AGD14 [Musa acuminata subsp. malaccensis]) | turquoise | 0.36985 |
| TRINITY_DN58102_c2_g4 | XP_010938166.1(PREDICTED: calmodulin-binding transcription activator 1 [Elaeis guineensis]) | turquoise | 0.48001 |
| TRINITY_DN65251_c3_g2 | OAY67061.1(NAC domain-containing protein 78 [Ananas comosus]) | turquoise | 0.58396 |
| TRINITY_DN65286_c4_g2 | XP_008782870.1(PREDICTED: protein FAR1-RELATED SEQUENCE 5-like isoform X1 [Phoenix dactylifera]) | turquoise | 0.7123 |
| TRINITY_DN67221_c0_g7 | ONH89763.1(hypothetical protein PRUPE_8G014900 [Prunus persica]) | turquoise | 0.5662 |
| TRINITY_DN72450_c1_g1 | XP_010930351.1(PREDICTED: protein FAR1-RELATED SEQUENCE 5-like [Elaeis guineensis]) | turquoise | 0.46314 |
| TRINITY_DN76242_c0_g1 | OAY67061.1(NAC domain-containing protein 78 [Ananas comosus]) | turquoise | 0.47043 |
| TRINITY_DN42894_c0_g1 | XP_008802063.1(PREDICTED: uncharacterized protein LOC103716014 [Phoenix dactylifera]) | turquoise | 0.86049 |
| TRINITY_DN43733_c0_g1 | XP_020275825.1(transcription factor TCP13-like [Asparagus officinalis]) | turquoise | 0.74304 |
| TRINITY_DN44301_c0_g1 | XP_010914936.1(PREDICTED: protein FAR1-RELATED SEQUENCE 5-like [Elaeis guineensis]) | turquoise | 0.84354 |
| TRINITY_DN44349_c0_g1 | OAY73141.1(Protein SHOOT GRAVITROPISM 5 [Ananas comosus]) | turquoise | 0.72579 |
| TRINITY_DN44690_c0_g1 | XP_009402416.1(PREDICTED: pathogenesis-related genes transcriptional activator PTI6-like [Musa acuminata subsp. malaccensis]) | turquoise | 0.91209 |
| TRINITY_DN44790_c0_g1 | XP_010927275.1(PREDICTED: WRKY transcription factor WRKY51-like [Elaeis guineensis]) | turquoise | 0.60218 |
| TRINITY_DN45166_c0_g1 | XP_020702645.1(transcription factor DIVARICATA-like [Dendrobium catenatum]) | turquoise | 0.70951 |
| TRINITY_DN46232_c0_g1 | XP_008781456.1(PREDICTED: histone-lysine N-methyltransferase ASHH1 isoform X1 [Phoenix dactylifera]) | turquoise | 0.82883 |
| TRINITY_DN46320_c3_g2 | XP_010932155.1(PREDICTED: squamosa promoter-binding-like protein 15 [Elaeis guineensis]) | turquoise | 0.91101 |
| TRINITY_DN46650_c0_g3 | XP_008779883.1(PREDICTED: dof zinc finger protein DOF3.5-like [Phoenix dactylifera]) | turquoise | 0.92189 |
| TRINITY_DN47007_c3_g7 | XP_018685313.1(PREDICTED: transcription factor PCL1-like isoform X2 [Musa acuminata subsp. malaccensis]) | turquoise | 0.70582 |
| TRINITY_DN47361_c1_g1 | XP_009412456.1(PREDICTED: ethylene-responsive transcription factor 4-like [Musa acuminata subsp. malaccensis]) | turquoise | 0.60419 |
| TRINITY_DN47854_c4_g10 | XP_010912370.2(PREDICTED: LOW QUALITY PROTEIN: protein NLP3 [Elaeis guineensis]) | turquoise | 0.88428 |
| TRINITY_DN47988_c7_g8 | XP_008794708.1(PREDICTED: myb family transcription factor PHL7-like isoform X2 [Phoenix dactylifera]) | turquoise | 0.88329 |
| TRINITY_DN48196_c3_g1 | XP_010245132.1(PREDICTED: L10-interacting MYB domain-containing protein-like [Nelumbo nucifera]) | turquoise | 0.80482 |
| TRINITY_DN48325_c2_g1 | XP_020686363.1(zinc finger CCCH domain-containing protein 28-like [Dendrobium catenatum]) | turquoise | 0.76836 |
| TRINITY_DN49028_c1_g4 | XP_009417256.1(PREDICTED: chitin-inducible gibberellin-responsive protein 1-like [Musa acuminata subsp. malaccensis]) | turquoise | 0.56343 |
| TRINITY_DN49505_c0_g1 | XP_019701544.1(PREDICTED: transcription factor BIM2 isoform X2 [Elaeis guineensis]) | turquoise | 0.76563 |
| TRINITY_DN49628_c0_g3 | XP_008790351.1(PREDICTED: SWI/SNF complex subunit SWI3D [Phoenix dactylifera]) | turquoise | 0.88749 |
| TRINITY_DN49778_c0_g2 | XP_010917575.1(PREDICTED: protein WRKY1 [Elaeis guineensis]) | turquoise | 0.75604 |
| TRINITY_DN50211_c3_g1 | XP_020272912.1(B3 domain-containing protein Os07g0679700-like isoform X2 [Asparagus officinalis]) | turquoise | 0.79609 |
| TRINITY_DN50641_c1_g1 | PKA60156.1(Transcription factor ASG4 [Apostasia shenzhenica]) | turquoise | 0.73633 |
| TRINITY_DN51008_c5_g1 | EAY82441.1(hypothetical protein OsI_37654 [Oryza sativa Indica Group]) | turquoise | 0.79154 |
| TRINITY_DN51929_c3_g1 | XP_020265250.1(auxin response factor 15-like isoform X1 [Asparagus officinalis]) | turquoise | 0.88867 |
| TRINITY_DN52830_c0_g2 | XP_008788487.1(PREDICTED: transcription factor HBP-1b(c38)-like [Phoenix dactylifera]) | turquoise | 0.8432 |
| TRINITY_DN53121_c5_g1 | XP_008786183.1(PREDICTED: GATA transcription factor 27-like [Phoenix dactylifera]) | turquoise | 0.84103 |
| TRINITY_DN53211_c3_g5 | XP_009394230.1(PREDICTED: protein indeterminate-domain 5, chloroplastic-like [Musa acuminata subsp. malaccensis]) | turquoise | 0.90779 |
| TRINITY_DN53443_c5_g2 | XP_010913153.1(PREDICTED: E3 ubiquitin-protein ligase makorin isoform X1 [Elaeis guineensis]) | turquoise | 0.83949 |
| TRINITY_DN53486_c1_g4 | XP_008791554.1(PREDICTED: auxin response factor 18 [Phoenix dactylifera]) | turquoise | 0.80409 |
| TRINITY_DN53713_c0_g2 | XP_010925293.1(PREDICTED: protein FAR1-RELATED SEQUENCE 6 [Elaeis guineensis]) | turquoise | 0.87214 |
| TRINITY_DN53764_c0_g1 | XP_010905494.1(PREDICTED: calmodulin-binding transcription activator 4 isoform X1 [Elaeis guineensis]) | turquoise | 0.82347 |
| TRINITY_DN54084_c0_g1 | XP_020581551.1(zinc finger CCCH domain-containing protein 53-like [Phalaenopsis equestris]) | turquoise | 0.93976 |
| TRINITY_DN54160_c5_g3 | XP_008775291.1(PREDICTED: uncharacterized protein LOC103695671 isoform X1 [Phoenix dactylifera]) | turquoise | 0.9372 |
| TRINITY_DN54394_c4_g2 | KMZ66811.1(Zinc finger CCCH domain-containing protein 63 [Zostera marina]) | turquoise | 0.87306 |
| TRINITY_DN54948_c0_g1 | XP_010919940.2(PREDICTED: transcription factor bHLH71-like isoform X2 [Elaeis guineensis]) | turquoise | 0.82225 |
| TRINITY_DN55710_c3_g2 | ONK65242.1(uncharacterized protein A4U43_C07F35130 [Asparagus officinalis]) | turquoise | 0.94079 |
| TRINITY_DN56009_c2_g2 | XP_010914036.1(PREDICTED: AP2-like ethylene-responsive transcription factor At1g79700 [Elaeis guineensis]) | turquoise | 0.76296 |
| TRINITY_DN56238_c0_g1 | XP_010906741.1(PREDICTED: zinc finger CCCH domain-containing protein 55-like isoform X2 [Elaeis guineensis]) | turquoise | 0.87461 |
| TRINITY_DN56768_c2_g2 | XP_009396365.1(PREDICTED: transcription factor MYB44-like [Musa acuminata subsp. malaccensis]) | turquoise | 0.6855 |
| TRINITY_DN56966_c2_g1 | XP_019705680.1(PREDICTED: bZIP transcription factor 16 isoform X4 [Elaeis guineensis]) | turquoise | 0.929 |
| TRINITY_DN56966_c2_g2 | XP_019705680.1(PREDICTED: bZIP transcription factor 16 isoform X4 [Elaeis guineensis]) | turquoise | 0.90172 |
| TRINITY_DN57384_c1_g3 | XP_010935427.1(PREDICTED: auxin response factor 12-like [Elaeis guineensis]) | turquoise | 0.91028 |
| TRINITY_DN57900_c5_g1 | XP_008802731.1(PREDICTED: myb-related protein B isoform X1 [Phoenix dactylifera]) | turquoise | 0.60282 |
| TRINITY_DN58023_c2_g1 | XP_020701159.1(transcription factor PCF2-like [Dendrobium catenatum]) | turquoise | 0.86335 |
| TRINITY_DN58023_c2_g4 | XP_009404879.1(PREDICTED: transcription factor TCP8-like [Musa acuminata subsp. malaccensis]) | turquoise | 0.83565 |
| TRINITY_DN58023_c2_g8 | XP_008812846.1(PREDICTED: transcription factor PCF2-like [Phoenix dactylifera]) | turquoise | 0.84583 |
| TRINITY_DN58195_c1_g3 | AHI42570.1(heat shock transcription factor A1 [Lilium longiflorum]) | turquoise | 0.86531 |
| TRINITY_DN58351_c1_g2 | PKA51327.1(Squamosa promoter-binding-like protein 17 [Apostasia shenzhenica]) | turquoise | 0.74426 |
| TRINITY_DN59349_c0_g4 |  | turquoise | 0.94915 |
| TRINITY_DN59356_c0_g1 | XP_010940314.1(PREDICTED: transcription factor ILR3 isoform X1 [Elaeis guineensis]) | turquoise | 0.76565 |
| TRINITY_DN59922_c0_g2 | XP_008793296.1(PREDICTED: NF-X1-type zinc finger protein NFXL2 [Phoenix dactylifera]) | turquoise | 0.69082 |
| TRINITY_DN60053_c0_g1 | XP_010934047.1(PREDICTED: transcription factor bHLH79 isoform X1 [Elaeis guineensis]) | turquoise | 0.87504 |
| TRINITY_DN60442_c0_g1 | XP_009390940.1(PREDICTED: pathogenesis-related genes transcriptional activator PTI6-like [Musa acuminata subsp. malaccensis]) | turquoise | 0.95586 |
| TRINITY_DN60890_c1_g1 | XP_010919976.1(PREDICTED: homeobox-leucine zipper protein ROC3-like [Elaeis guineensis]) | turquoise | 0.8054 |
| TRINITY_DN60976_c0_g2 | XP_008800636.1(PREDICTED: zinc finger CCCH domain-containing protein 18-like [Phoenix dactylifera]) | turquoise | 0.89135 |
| TRINITY_DN62481_c0_g1 | XP_018822991.1(PREDICTED: transcription factor MYB98-like [Juglans regia]) | turquoise | 0.75277 |
| TRINITY_DN63021_c2_g1 | XP_010941526.1(PREDICTED: auxin response factor 17-like [Elaeis guineensis]) | turquoise | 0.82437 |
| TRINITY_DN63073_c0_g2 | AIR77001.1(ethylene response factor, partial [Lilium longiflorum]) | turquoise | 0.88587 |
| TRINITY_DN63377_c0_g3 | XP_008775282.1(PREDICTED: uncharacterized protein LOC103695671 isoform X3 [Phoenix dactylifera]) | turquoise | 0.79176 |
| TRINITY_DN63390_c3_g1 | ONK73904.1(uncharacterized protein A4U43_C03F780 [Asparagus officinalis]) | turquoise | 0.76011 |
| TRINITY_DN63854_c4_g1 | XP_010929360.1(PREDICTED: pathogenesis-related homeodomain protein-like [Elaeis guineensis]) | turquoise | 0.66156 |
| TRINITY_DN64276_c0_g1 | XP_010912147.1(PREDICTED: transcription factor bHLH13 [Elaeis guineensis]) | turquoise | 0.88482 |
| TRINITY_DN64706_c1_g2 | XP_008793917.1(PREDICTED: auxin response factor 17-like [Phoenix dactylifera]) | turquoise | 0.93486 |
| TRINITY_DN64754_c5_g1 | XP_009388968.1(PREDICTED: transcription factor PCF6-like [Musa acuminata subsp. malaccensis]) | turquoise | 0.81924 |
| TRINITY_DN65018_c0_g1 | XP_010932454.1(PREDICTED: uncharacterized protein LOC105053113 isoform X1 [Elaeis guineensis]) | turquoise | 0.82347 |
| TRINITY_DN65034_c4_g1 | XP_010930511.1(PREDICTED: protein FAR1-RELATED SEQUENCE 11 [Elaeis guineensis]) | turquoise | 0.93885 |
| TRINITY_DN65471_c0_g1 | XP_010906525.1(PREDICTED: protein FAR1-RELATED SEQUENCE 11-like [Elaeis guineensis]) | turquoise | 0.95688 |
| TRINITY_DN65496_c3_g1 | KYP75547.1(Myb-like protein G [Cajanus cajan]) | turquoise | 0.36159 |
| TRINITY_DN65693_c2_g1 | XP_010937133.1(PREDICTED: zinc-finger homeodomain protein 9 [Elaeis guineensis]) | turquoise | 0.89496 |
| TRINITY_DN65808_c4_g1 | AID68259.1(whirly transcription factor domain containing protein [Musa acuminata AAA Group]) | turquoise | 0.92648 |
| TRINITY_DN66148_c0_g1 | XP_010912181.1(PREDICTED: auxin response factor 11 isoform X1 [Elaeis guineensis]) | turquoise | 0.41397 |
| TRINITY_DN66770_c5_g1 | XP_010912055.1(PREDICTED: LOW QUALITY PROTEIN: auxin response factor 17-like [Elaeis guineensis]) | turquoise | 0.68752 |
| TRINITY_DN66770_c5_g3 | XP_008785886.1(PREDICTED: auxin response factor 17-like [Phoenix dactylifera]) | turquoise | 0.781 |
| TRINITY_DN66892_c1_g4 | XP_010905301.1(PREDICTED: WRKY transcription factor SUSIBA2 isoform X1 [Elaeis guineensis]) | turquoise | 0.93654 |
| TRINITY_DN67436_c3_g4 | XP_010260864.1(PREDICTED: zinc finger CCCH domain-containing protein 37 isoform X1 [Nelumbo nucifera]) | turquoise | 0.90613 |
| TRINITY_DN67837_c0_g1 | XP_010929341.1(PREDICTED: transcription factor bHLH35 isoform X3 [Elaeis guineensis]) | turquoise | 0.71264 |
| TRINITY_DN67960_c0_g1 | XP_010941466.1(PREDICTED: transcription factor PIF3 isoform X2 [Elaeis guineensis]) | turquoise | 0.93498 |
| TRINITY_DN67978_c1_g3 | XP_010936276.1(PREDICTED: scarecrow-like protein 4 [Elaeis guineensis]) | turquoise | 0.87161 |
| TRINITY_DN68500_c0_g1 | XP_020584529.1(DELLA protein SLR1-like [Phalaenopsis equestris]) | turquoise | 0.91021 |
| TRINITY_DN68500_c0_g2 | XP_020682060.1(DELLA protein SLN1-like [Dendrobium catenatum]) | turquoise | 0.91896 |
| TRINITY_DN68840_c0_g2 | XP_008805601.1(PREDICTED: mediator-associated protein 1-like [Phoenix dactylifera]) | turquoise | 0.89215 |
| TRINITY_DN68857_c0_g1 | XP_010931820.1(PREDICTED: homeobox-leucine zipper protein ROC2 [Elaeis guineensis]) | turquoise | 0.80724 |
| TRINITY_DN71095_c3_g10 | XP_010909933.1(PREDICTED: heat stress transcription factor A-1-like [Elaeis guineensis]) | turquoise | 0.88749 |
| TRINITY_DN71919_c0_g6 | XP_008775348.1(PREDICTED: lysine-specific demethylase SE14 [Phoenix dactylifera]) | turquoise | 0.76443 |
| TRINITY_DN71928_c0_g1 | XP_024038296.1(PKS-NRPS hybrid synthetase CHGG_01239-like [Citrus clementina]) | turquoise | 0.88048 |
| TRINITY_DN72285_c1_g2 | XP_010249637.1(PREDICTED: transcription factor TCP7 [Nelumbo nucifera]) | turquoise | 0.7126 |
| TRINITY_DN72285_c1_g3 | XP_004975168.1(transcription factor PCF1-like [Setaria italica]) | turquoise | 0.95922 |
| TRINITY_DN72315_c1_g2 | XP_009408534.1(PREDICTED: transcription factor PCF6-like [Musa acuminata subsp. malaccensis]) | turquoise | 0.78836 |
| TRINITY_DN72443_c1_g2 | XP_010934047.1(PREDICTED: transcription factor bHLH79 isoform X1 [Elaeis guineensis]) | turquoise | 0.88693 |
| TRINITY_DN72651_c2_g1 | ATY36263.1(TGA2 [Lilium hybrid cultivar]) | turquoise | 0.86932 |
| TRINITY_DN72802_c2_g1 | XP_009394230.1(PREDICTED: protein indeterminate-domain 5, chloroplastic-like [Musa acuminata subsp. malaccensis]) | turquoise | 0.94678 |
| TRINITY_DN73119_c0_g2 | XP_020259754.1(homeobox-leucine zipper protein HOX9-like [Asparagus officinalis]) | turquoise | 0.87594 |
| TRINITY_DN73139_c0_g4 | XP_008805201.1(PREDICTED: GATA transcription factor 4-like [Phoenix dactylifera]) | turquoise | 0.79274 |
| TRINITY_DN73211_c2_g1 | XP_008794452.1(PREDICTED: homeobox-leucine zipper protein HOX32 [Phoenix dactylifera]) | turquoise | 0.87262 |
| TRINITY_DN73986_c2_g1 | XP_010940634.1(PREDICTED: splicing factor U2af small subunit B [Elaeis guineensis]) | turquoise | 0.92408 |
| TRINITY_DN74205_c0_g1 | XP_008781504.1(PREDICTED: uncharacterized protein LOC103701275 [Phoenix dactylifera]) | turquoise | 0.81878 |
| TRINITY_DN74646_c4_g3 | XP_019707167.1(PREDICTED: transcription factor TCP20-like [Elaeis guineensis]) | turquoise | 0.56527 |
| TRINITY_DN74764_c0_g1 | XP_023922000.1(zinc finger protein 346-like [Quercus suber]) | turquoise | 0.89647 |
| TRINITY_DN75176_c1_g1 | XP_010931909.1(PREDICTED: transcription factor IIIA [Elaeis guineensis]) | turquoise | 0.83501 |
| TRINITY_DN75200_c3_g1 | XP_008813491.1(PREDICTED: homeobox-DDT domain protein RLT3 [Phoenix dactylifera]) | turquoise | 0.85356 |
| TRINITY_DN75574_c1_g1 | XP_010942128.1(PREDICTED: two-component response regulator ORR22-like isoform X2 [Elaeis guineensis]) | turquoise | 0.85636 |
| TRINITY_DN75786_c1_g1 | XP_010926981.1(PREDICTED: protein LATERAL ROOT PRIMORDIUM 1-like [Elaeis guineensis]) | turquoise | 0.62019 |
| TRINITY_DN75825_c1_g2 | KMT08391.1(hypothetical protein BVRB_6g140700 [Beta vulgaris subsp. vulgaris]) | turquoise | 0.69143 |
| TRINITY_DN76660_c8_g1 | XP_010248777.1(PREDICTED: zinc finger CCCH domain-containing protein 67 isoform X1 [Nelumbo nucifera]) | turquoise | 0.80255 |
| TRINITY_DN91387_c0_g1 | ART33467.1(ethylene response factor 9 [Lilium regale]) | turquoise | 0.7779 |
| TRINITY_DN91546_c0_g1 | XP_009400292.1(PREDICTED: ethylene-responsive transcription factor ERF053-like [Musa acuminata subsp. malaccensis]) | turquoise | 0.69559 |
| TRINITY_DN60579_c1_g2 | XP_008807659.1(PREDICTED: SWI/SNF complex subunit SWI3C-like [Phoenix dactylifera]) | turquoise | 0.394 |
| TRINITY_DN60281_c4_g1 | XP_008799098.1(PREDICTED: zinc finger CCCH domain-containing protein 45-like [Phoenix dactylifera]) | yellow | 0.74793 |
| TRINITY_DN60598_c4_g3 | XP_008225697.1(PREDICTED: protein LSD1 [Prunus mume]) | yellow | 0.46754 |
| TRINITY_DN44685_c1_g1 | PKA63123.1(Transcription factor DIVARICATA [Apostasia shenzhenica]) | yellow | 0.49238 |
| TRINITY_DN46487_c1_g2 | XP_010942495.1(PREDICTED: myb family transcription factor PHL7 isoform X1 [Elaeis guineensis]) | yellow | 0.78257 |
| TRINITY_DN47127_c1_g2 | XP_021275339.1(WUSCHEL-related homeobox 13 isoform X2 [Herrania umbratica]) | yellow | 0.83655 |
| TRINITY_DN53195_c0_g2 | XP_008784021.1(PREDICTED: protein FAR1-RELATED SEQUENCE 5-like [Phoenix dactylifera]) | yellow | 0.81536 |
| TRINITY_DN58264_c0_g1 | XP_020276659.1(zinc finger protein ZAT4-like [Asparagus officinalis]) | yellow | 0.85432 |
| TRINITY_DN58371_c2_g1 | XP_010941212.1(PREDICTED: dnaJ homolog subfamily C member 2 [Elaeis guineensis]) | yellow | 0.7151 |
| TRINITY_DN62477_c0_g5 | XP_020257981.1(LOW QUALITY PROTEIN: zinc finger CCCH domain-containing protein 24-like [Asparagus officinalis]) | yellow | 0.77501 |
| TRINITY_DN64795_c0_g3 | XP_010934970.1(PREDICTED: scarecrow-like protein 27 [Elaeis guineensis]) | yellow | 0.66114 |
| TRINITY_DN66670_c0_g1 | XP_010909305.1(PREDICTED: bZIP transcription factor 39-like [Elaeis guineensis]) | yellow | 0.63959 |
| TRINITY_DN68501_c3_g3 | XP_008801132.1(PREDICTED: zinc finger protein CONSTANS-LIKE 13-like [Phoenix dactylifera]) | yellow | 0.6437 |
| TRINITY_DN68520_c2_g4 | XP_010926423.2(PREDICTED: LOW QUALITY PROTEIN: smoothelin-like [Elaeis guineensis]) | yellow | 0.54376 |
| TRINITY_DN73314_c5_g7 | XP_021610902.1(bZIP transcription factor 11-like [Manihot esculenta]) | yellow | 0.583 |
| TRINITY_DN73574_c3_g1 | XP_019705681.1(PREDICTED: trihelix transcription factor GTL1-like isoform X1 [Elaeis guineensis]) | yellow | 0.63926 |
| TRINITY_DN53802_c2_g1 | XP_008803514.1(PREDICTED: zinc finger CCCH domain-containing protein 57-like isoform X2 [Phoenix dactylifera]) | yellow | 0.64016 |
| TRINITY_DN64740_c0_g1 | XP_010921543.1(PREDICTED: LOB domain-containing protein 41 [Elaeis guineensis]) | yellow | 0.42805 |
| TRINITY_DN63637_c0_g1 | AGN52057.1(MYB-related transcription factor [Salvia miltiorrhiza]) | yellow | 0.30107 |
| TRINITY_DN64795_c0_g6 | XP_008794809.2(PREDICTED: scarecrow-like protein 27 [Phoenix dactylifera]) | yellow | 0.72565 |
| TRINITY_DN68856_c1_g3 | XP_010916003.1(PREDICTED: protein CUP-SHAPED COTYLEDON 2 [Elaeis guineensis]) | yellow | 0.47094 |
| TRINITY_DN76223_c4_g2 | XP_008810430.1(PREDICTED: transcription factor PCL1-like [Phoenix dactylifera]) | yellow | 0.52179 |
| TRINITY_DN63342_c2_g1 | ONK77375.1(uncharacterized protein A4U43_C02F5870 [Asparagus officinalis]) | yellow | 0.71465 |
| TRINITY_DN47772_c0_g6 | XP_010931461.1(PREDICTED: mini zinc finger protein 1-like [Elaeis guineensis]) | yellow | 0.86248 |
| TRINITY_DN51786_c3_g2 | XP_010921105.1(PREDICTED: NAC transcription factor NAM-B2 [Elaeis guineensis]) | yellow | 0.80852 |
| TRINITY_DN53802_c2_g2 | XP_020265527.1(zinc finger CCCH domain-containing protein 33-like isoform X1 [Asparagus officinalis]) | yellow | 0.69596 |
| TRINITY_DN66186_c1_g1 | XP_008448879.1(PREDICTED: transcription factor MYB86 [Cucumis melo]) | yellow | 0.7487 |
| TRINITY_DN75833_c12_g1 | BAB40790.1(LhMyb [Lilium hybrid division I]) | yellow | 0.69325 |
| TRINITY_DN59923_c0_g3 | XP_020251511.1(bZIP transcription factor 44-like [Asparagus officinalis]) | yellow | 0.51208 |
| TRINITY_DN43892_c0_g1 | XP_010919568.1(PREDICTED: LOW QUALITY PROTEIN: zinc finger protein ZAT4 [Elaeis guineensis]) | yellow | 0.9295 |
| TRINITY_DN44790_c0_g2 | KMZ66556.1(WRKY transcription factor 7 [Zostera marina]) | yellow | 0.68314 |
| TRINITY_DN46347_c0_g4 | XP_008783453.1(PREDICTED: scarecrow-like protein 8 [Phoenix dactylifera]) | yellow | 0.89951 |
| TRINITY_DN48198_c0_g1 | BAJ98846.1(predicted protein, partial [Hordeum vulgare subsp. vulgare]) | yellow | 0.86455 |
| TRINITY_DN48198_c0_g2 | XP_010924728.1(PREDICTED: probable WRKY transcription factor 65 isoform X2 [Elaeis guineensis]) | yellow | 0.75648 |
| TRINITY_DN48225_c0_g2 | XP_008795931.1(PREDICTED: protein RADIALIS-like 3 [Phoenix dactylifera]) | yellow | 0.55862 |
| TRINITY_DN52803_c1_g1 | XP_008785327.1(PREDICTED: protein FAR1-RELATED SEQUENCE 5-like [Phoenix dactylifera]) | yellow | 0.77997 |
| TRINITY_DN55390_c0_g1 | AIF76294.1(CONSTANS-like protein, partial [Lilium hybrid cultivar]) | yellow | 0.91624 |
| TRINITY_DN55496_c2_g2 | XP_008802484.1(PREDICTED: dof zinc finger protein PBF-like [Phoenix dactylifera]) | yellow | 0.71366 |
| TRINITY_DN58690_c0_g1 | XP_008791076.1(PREDICTED: protein spotted leaf 11 [Phoenix dactylifera]) | yellow | 0.78648 |
| TRINITY_DN59319_c0_g3 | XP_008789280.1(PREDICTED: NF-X1-type zinc finger protein NFXL1 [Phoenix dactylifera]) | yellow | 0.46713 |
| TRINITY_DN59892_c0_g4 | XP_020246876.1(dehydration-responsive element-binding protein 3-like [Asparagus officinalis]) | yellow | 0.85894 |
| TRINITY_DN64527_c1_g2 | XP_010912518.1(PREDICTED: uncharacterized zinc finger protein At4g06634 isoform X1 [Elaeis guineensis]) | yellow | 0.83291 |
| TRINITY_DN67245_c0_g2 | XP_020264895.1(B3 domain-containing protein Os01g0723500-like [Asparagus officinalis]) | yellow | 0.75207 |
| TRINITY_DN68196_c1_g1 | XP_020240915.1(uncharacterized protein LOC109819569 [Asparagus officinalis]) | yellow | 0.80896 |
| TRINITY_DN69913_c1_g6 | ART33466.1(ethylene response factor 3 [Lilium regale]) | yellow | 0.46147 |
| TRINITY_DN70269_c1_g1 | XP_009381996.1(PREDICTED: uncharacterized protein LOC103970083 [Musa acuminata subsp. malaccensis]) | yellow | 0.7477 |
| TRINITY_DN70339_c1_g1 | XP_008802569.1(PREDICTED: NAC domain-containing protein 100-like [Phoenix dactylifera]) | yellow | 0.83827 |
| TRINITY_DN70461_c2_g4 | XP_020277093.1(protein CUP-SHAPED COTYLEDON 3-like [Asparagus officinalis]) | yellow | 0.8753 |
| TRINITY_DN73172_c0_g3 | XP_008812314.1(PREDICTED: LOW QUALITY PROTEIN: zinc finger CCCH domain-containing protein ZFN-like [Phoenix dactylifera]) | yellow | 0.51241 |
| TRINITY_DN73366_c2_g1 | BAU68654.1(R2R3-MYB transcriptional factor [Lilium hybrid division I]) | yellow | 0.85311 |
| TRINITY_DN73580_c1_g1 | XP_008786269.1(PREDICTED: ethylene-responsive transcription factor ERF105-like [Phoenix dactylifera]) | yellow | 0.61601 |
| TRINITY_DN73969_c3_g1 | XP_008784959.1(PREDICTED: NAC domain-containing protein 74-like [Phoenix dactylifera]) | yellow | 0.59195 |
| TRINITY_DN76002_c5_g3 | AGO33167.1(MYB transcription factor [Narcissus tazetta var. chinensis]) | yellow | 0.663 |
| TRINITY_DN76513_c2_g1 | XP_010913776.1(PREDICTED: basic leucine zipper 9 [Elaeis guineensis]) | yellow | 0.74301 |

**Supplementary Table 6.** Gene expression of TFs identified of WGCNA

| **Gene_id** | **S0** | **S1** | **S2** | **S3** | **S4** | **S5** | **S6** | **S7** | **S8** | **S9** | **S10** |
| --- | --- | --- | --- | --- | --- | --- | --- | --- | --- | --- | --- |
| TRINITY_DN23141_c0_g1 | 7.45 | 8.80 | 13.54 | 16.49 | 11.27 | 13.94 | 7.93 | 9.80 | 18.46 | 35.10 | 39.40 |
| TRINITY_DN36237_c0_g1 | 67.49 | 62.96 | 138.71 | 87.11 | 25.81 | 27.02 | 0.77 | 0.18 | 0.22 | 0.01 | 0.07 |
| TRINITY_DN36993_c0_g1 | 17.92 | 12.41 | 10.82 | 4.31 | 2.60 | 2.34 | 1.10 | 0.60 | 0.58 | 0.50 | 1.36 |
| TRINITY_DN38127_c0_g1 | 12.31 | 14.57 | 9.49 | 9.72 | 8.26 | 9.73 | 5.47 | 5.36 | 4.29 | 4.58 | 4.34 |
| TRINITY_DN39835_c0_g1 | 2.90 | 3.28 | 1.98 | 2.48 | 3.83 | 3.29 | 2.04 | 1.60 | 5.39 | 5.02 | 4.99 |
| TRINITY_DN40189_c0_g1 | 7.82 | 7.91 | 6.57 | 8.94 | 7.32 | 8.37 | 7.99 | 6.83 | 5.58 | 5.56 | 1.64 |
| TRINITY_DN40244_c0_g1 | 13.11 | 18.77 | 10.85 | 15.20 | 9.36 | 12.16 | 10.02 | 7.57 | 6.89 | 4.75 | 0.61 |
| TRINITY_DN41860_c0_g1 | 4.06 | 5.44 | 3.45 | 0.71 | 1.83 | 1.14 | 1.99 | 1.60 | 4.48 | 25.61 | 79.65 |
| TRINITY_DN42064_c0_g1 | 43.91 | 36.50 | 78.05 | 107.46 | 68.41 | 83.53 | 7.42 | 0.71 | 0.34 | 0.30 | 0.00 |
| TRINITY_DN42110_c0_g1 | 3.62 | 3.15 | 3.30 | 11.28 | 10.22 | 8.03 | 8.28 | 12.50 | 36.45 | 32.92 | 54.31 |
| TRINITY_DN42130_c0_g1 | 135.68 | 128.15 | 51.39 | 54.72 | 17.66 | 22.01 | 20.35 | 26.81 | 20.28 | 6.26 | 4.32 |
| TRINITY_DN42741_c0_g1 | 48.93 | 53.24 | 16.86 | 13.85 | 2.83 | 3.07 | 1.80 | 0.86 | 2.70 | 1.33 | 0.00 |
| TRINITY_DN42894_c0_g1 | 31.17 | 26.03 | 21.09 | 23.58 | 12.43 | 12.52 | 10.91 | 11.73 | 11.54 | 7.41 | 2.79 |
| TRINITY_DN43128_c0_g1 | 2.86 | 2.70 | 0.93 | 3.74 | 5.38 | 3.56 | 3.51 | 7.53 | 11.07 | 1.75 | 0.24 |
| TRINITY_DN43190_c0_g1 | 12.98 | 11.83 | 4.67 | 3.05 | 1.73 | 2.32 | 1.06 | 1.72 | 4.06 | 2.08 | 3.88 |
| TRINITY_DN43391_c0_g1 | 1.56 | 1.55 | 7.65 | 3.95 | 2.51 | 2.00 | 0.09 | 0.10 | 0.46 | 11.73 | 17.14 |
| TRINITY_DN43446_c0_g1 | 24.75 | 15.64 | 9.92 | 7.70 | 8.97 | 8.94 | 9.66 | 21.81 | 18.99 | 34.05 | 26.29 |
| TRINITY_DN43733_c0_g1 | 133.75 | 141.34 | 160.20 | 99.66 | 94.73 | 105.29 | 48.33 | 39.58 | 54.84 | 36.08 | 22.93 |
| TRINITY_DN43892_c0_g1 | 1.12 | 2.36 | 1.63 | 5.62 | 10.63 | 8.35 | 9.67 | 13.80 | 17.02 | 16.61 | 1.04 |
| TRINITY_DN44011_c0_g1 | 17.09 | 13.14 | 7.29 | 4.89 | 1.28 | 0.66 | 1.45 | 1.49 | 0.77 | 0.45 | 0.35 |
| TRINITY_DN44153_c0_g1 | 10.51 | 7.38 | 5.45 | 3.66 | 1.69 | 1.25 | 1.18 | 0.41 | 1.07 | 22.63 | 0.37 |
| TRINITY_DN44301_c0_g1 | 8.95 | 6.61 | 5.83 | 7.37 | 5.20 | 4.91 | 4.34 | 4.67 | 3.32 | 2.26 | 1.91 |
| TRINITY_DN44307_c0_g1 | 7.68 | 4.96 | 3.91 | 5.34 | 3.87 | 3.34 | 1.24 | 4.72 | 11.65 | 10.32 | 2.18 |
| TRINITY_DN44348_c0_g1 | 22.57 | 25.96 | 10.29 | 6.28 | 1.11 | 1.16 | 0.05 | 0.03 | 0.11 | 0.03 | 0.01 |
| TRINITY_DN44349_c0_g1 | 20.85 | 11.33 | 3.39 | 10.96 | 6.41 | 3.92 | 4.04 | 3.67 | 3.96 | 2.21 | 0.05 |
| TRINITY_DN44570_c0_g1 | 192.55 | 127.72 | 107.74 | 69.96 | 71.49 | 43.46 | 30.66 | 26.25 | 21.26 | 23.98 | 35.70 |
| TRINITY_DN44662_c0_g1 | 1.99 | 3.47 | 1.86 | 1.22 | 1.81 | 1.78 | 1.06 | 3.11 | 4.75 | 12.06 | 46.34 |
| TRINITY_DN44685_c1_g1 | 36.63 | 50.08 | 37.55 | 34.48 | 28.98 | 18.36 | 33.97 | 69.17 | 59.73 | 86.62 | 6.98 |
| TRINITY_DN44690_c0_g1 | 42.61 | 52.34 | 94.18 | 84.95 | 55.93 | 49.56 | 32.15 | 29.14 | 16.16 | 11.86 | 0.10 |
| TRINITY_DN44790_c0_g1 | 36.40 | 31.33 | 11.05 | 13.21 | 16.60 | 15.50 | 14.95 | 14.34 | 19.32 | 21.66 | 3.86 |
| TRINITY_DN44790_c0_g2 | 3.22 | 3.64 | 1.81 | 3.48 | 5.29 | 5.14 | 5.99 | 8.73 | 12.52 | 8.80 | 3.95 |
| TRINITY_DN44901_c4_g2 | 9.26 | 7.42 | 7.04 | 11.41 | 15.43 | 15.56 | 15.25 | 16.72 | 18.77 | 16.59 | 37.46 |
| TRINITY_DN44961_c0_g1 | 0.48 | 1.63 | 0.78 | 0.37 | 0.68 | 0.50 | 0.38 | 0.36 | 5.52 | 2.82 | 122.03 |
| TRINITY_DN44964_c0_g1 | 21.09 | 16.23 | 5.60 | 6.74 | 2.16 | 1.16 | 0.46 | 0.37 | 0.44 | 0.52 | 0.10 |
| TRINITY_DN45078_c0_g1 | 5.33 | 5.22 | 5.91 | 6.18 | 6.11 | 5.99 | 6.55 | 4.83 | 9.06 | 9.59 | 8.64 |
| TRINITY_DN45166_c0_g1 | 42.00 | 32.79 | 31.03 | 30.52 | 22.54 | 19.82 | 19.54 | 22.67 | 33.43 | 29.99 | 4.65 |
| TRINITY_DN45166_c0_g2 | 20.21 | 17.88 | 14.30 | 13.82 | 8.68 | 7.36 | 4.49 | 3.52 | 5.59 | 4.47 | 2.02 |
| TRINITY_DN45177_c0_g1 | 35.74 | 28.28 | 19.48 | 21.84 | 13.59 | 11.74 | 10.52 | 10.62 | 7.87 | 6.72 | 2.81 |
| TRINITY_DN45464_c0_g1 | 5.18 | 3.90 | 1.71 | 2.71 | 2.38 | 1.79 | 1.48 | 2.18 | 1.60 | 2.28 | 2.20 |
| TRINITY_DN45464_c0_g2 | 12.65 | 8.58 | 5.09 | 7.44 | 6.19 | 4.78 | 2.87 | 2.95 | 3.02 | 3.38 | 0.93 |
| TRINITY_DN45519_c0_g2 | 5.41 | 5.76 | 4.78 | 4.84 | 4.95 | 6.24 | 4.86 | 3.93 | 5.54 | 5.25 | 3.39 |
| TRINITY_DN45611_c2_g1 | 27.24 | 28.77 | 17.53 | 20.53 | 15.20 | 18.15 | 11.47 | 7.23 | 15.95 | 9.33 | 1.60 |
| TRINITY_DN45774_c0_g1 | 3.65 | 2.97 | 3.34 | 3.23 | 4.41 | 3.26 | 3.08 | 6.38 | 2.72 | 2.08 | 6.87 |
| TRINITY_DN45791_c0_g1 | 6.96 | 5.35 | 2.70 | 1.45 | 1.00 | 0.82 | 0.58 | 1.42 | 0.58 | 0.97 | 29.47 |
| TRINITY_DN45813_c0_g1 | 43.35 | 38.85 | 22.99 | 18.68 | 5.11 | 3.97 | 2.78 | 2.28 | 1.29 | 5.07 | 0.39 |
| TRINITY_DN45950_c0_g2 | 70.35 | 68.28 | 57.10 | 20.26 | 9.25 | 6.18 | 3.69 | 4.76 | 9.73 | 14.01 | 3.40 |
| TRINITY_DN46171_c2_g6 | 11.40 | 9.38 | 9.30 | 17.64 | 18.76 | 14.94 | 16.22 | 18.98 | 11.50 | 7.58 | 8.95 |
| TRINITY_DN46232_c0_g1 | 11.82 | 9.77 | 10.90 | 11.28 | 12.51 | 11.31 | 8.69 | 12.34 | 10.33 | 5.61 | 4.86 |
| TRINITY_DN46300_c1_g1 | 7.57 | 7.85 | 1.99 | 1.04 | 1.18 | 0.67 | 0.50 | 1.14 | 3.71 | 5.40 | 0.65 |
| TRINITY_DN46300_c1_g2 | 17.17 | 11.53 | 5.36 | 10.99 | 5.54 | 4.09 | 2.04 | 4.15 | 2.69 | 2.67 | 4.38 |
| TRINITY_DN46320_c3_g2 | 25.30 | 24.79 | 20.09 | 26.58 | 29.40 | 25.37 | 25.00 | 22.63 | 20.27 | 6.22 | 3.46 |
| TRINITY_DN46320_c3_g3 | 17.40 | 24.99 | 19.62 | 22.62 | 22.36 | 29.29 | 40.49 | 14.62 | 38.91 | 8.97 | 0.16 |
| TRINITY_DN46347_c0_g4 | 12.02 | 14.25 | 30.27 | 51.68 | 47.87 | 58.41 | 56.80 | 71.26 | 72.65 | 63.27 | 14.97 |
| TRINITY_DN46487_c1_g2 | 12.18 | 11.78 | 10.77 | 15.00 | 18.55 | 19.40 | 16.65 | 18.31 | 16.53 | 17.84 | 7.89 |
| TRINITY_DN46575_c0_g1 | 2.34 | 2.06 | 1.97 | 3.07 | 3.57 | 2.79 | 1.82 | 0.80 | 4.10 | 1.63 | 0.02 |
| TRINITY_DN46621_c0_g1 | 15.00 | 8.62 | 1.83 | 2.37 | 1.73 | 1.47 | 1.01 | 0.44 | 0.49 | 0.67 | 2.32 |
| TRINITY_DN46650_c0_g3 | 74.73 | 49.77 | 47.42 | 53.75 | 34.80 | 39.13 | 22.82 | 25.87 | 11.00 | 8.83 | 2.30 |
| TRINITY_DN46650_c0_g6 | 7.78 | 7.70 | 4.61 | 7.49 | 5.51 | 3.89 | 7.75 | 8.44 | 6.94 | 6.59 | 0.99 |
| TRINITY_DN46650_c0_g8 | 5.21 | 4.47 | 1.87 | 2.40 | 1.72 | 0.85 | 1.71 | 2.46 | 1.45 | 0.18 | 2.69 |
| TRINITY_DN46650_c0_g9 | 2.74 | 2.72 | 1.80 | 2.65 | 2.66 | 2.25 | 2.76 | 3.62 | 2.64 | 2.90 | 1.22 |
| TRINITY_DN46675_c1_g1 | 24.92 | 19.42 | 13.91 | 17.66 | 32.72 | 32.53 | 18.83 | 5.48 | 14.37 | 7.98 | 0.88 |
| TRINITY_DN46675_c1_g2 | 16.81 | 15.87 | 10.46 | 12.05 | 14.21 | 13.95 | 4.55 | 2.13 | 2.97 | 1.67 | 1.23 |
| TRINITY_DN46690_c0_g1 | 7.98 | 5.74 | 5.71 | 5.82 | 5.26 | 5.07 | 5.27 | 6.44 | 4.50 | 3.52 | 4.02 |
| TRINITY_DN46762_c3_g1 | 3.61 | 3.83 | 3.78 | 4.67 | 4.70 | 4.02 | 3.98 | 3.97 | 6.99 | 6.60 | 4.91 |
| TRINITY_DN46929_c0_g10 | 4.11 | 7.04 | 2.08 | 2.63 | 1.43 | 3.09 | 1.69 | 2.65 | 2.46 | 16.00 | 0.17 |
| TRINITY_DN47007_c3_g7 | 37.66 | 28.86 | 42.83 | 43.71 | 44.61 | 29.78 | 35.18 | 60.57 | 24.82 | 22.69 | 7.24 |
| TRINITY_DN47087_c3_g2 | 170.25 | 125.47 | 95.79 | 39.79 | 2.81 | 1.83 | 1.80 | 1.54 | 0.86 | 0.64 | 0.21 |
| TRINITY_DN47087_c3_g8 | 8.80 | 19.97 | 14.01 | 14.55 | 14.73 | 47.02 | 12.19 | 4.23 | 11.65 | 30.11 | 21.33 |
| TRINITY_DN47127_c1_g2 | 7.54 | 7.05 | 4.70 | 9.95 | 13.03 | 11.14 | 8.12 | 17.15 | 14.32 | 15.83 | 0.50 |
| TRINITY_DN47127_c1_g3 | 12.37 | 8.95 | 10.28 | 12.16 | 16.49 | 14.88 | 8.05 | 5.64 | 8.70 | 2.50 | 0.53 |
| TRINITY_DN47309_c1_g3 | 9.40 | 5.85 | 7.49 | 5.23 | 6.16 | 5.51 | 7.90 | 3.76 | 5.92 | 3.98 | 0.60 |
| TRINITY_DN47309_c1_g6 | 39.22 | 34.35 | 24.30 | 30.76 | 24.97 | 22.79 | 12.72 | 8.82 | 12.37 | 6.28 | 3.98 |
| TRINITY_DN47361_c1_g1 | 7.91 | 11.19 | 5.92 | 8.57 | 5.60 | 16.25 | 3.91 | 7.81 | 1.51 | 1.92 | 1.91 |
| TRINITY_DN47361_c1_g6 | 21.70 | 25.25 | 4.72 | 5.41 | 0.66 | 6.34 | 1.26 | 2.93 | 1.86 | 2.17 | 38.01 |
| TRINITY_DN47468_c3_g2 | 12.63 | 9.40 | 6.51 | 9.86 | 4.98 | 4.16 | 2.17 | 5.20 | 4.60 | 16.45 | 28.56 |
| TRINITY_DN47534_c0_g1 | 15.78 | 12.17 | 13.64 | 16.90 | 15.62 | 11.96 | 8.25 | 6.84 | 13.84 | 3.28 | 0.35 |
| TRINITY_DN47735_c4_g1 | 3.50 | 4.20 | 5.02 | 4.75 | 4.38 | 4.15 | 1.28 | 0.46 | 1.31 | 0.89 | 0.46 |
| TRINITY_DN47772_c0_g3 | 0.79 | 2.48 | 2.54 | 5.50 | 5.36 | 14.58 | 13.10 | 7.18 | 2.99 | 2.09 | 0.18 |
| TRINITY_DN47772_c0_g6 | 0.85 | 0.63 | 18.85 | 157.19 | 329.72 | 331.22 | 419.61 | 626.08 | 148.08 | 79.53 | 1.70 |
| TRINITY_DN47854_c4_g10 | 18.63 | 16.73 | 12.54 | 14.68 | 10.71 | 10.50 | 11.78 | 11.97 | 11.92 | 9.85 | 3.02 |
| TRINITY_DN47948_c4_g1 | 30.69 | 28.02 | 24.87 | 35.37 | 47.44 | 43.76 | 37.95 | 32.73 | 50.14 | 19.22 | 9.14 |
| TRINITY_DN47961_c0_g1 | 21.24 | 16.65 | 9.12 | 6.19 | 4.05 | 3.03 | 1.63 | 2.15 | 3.23 | 5.70 | 12.67 |
| TRINITY_DN47988_c7_g8 | 12.44 | 12.60 | 10.14 | 11.17 | 17.71 | 11.20 | 8.57 | 11.16 | 10.39 | 5.96 | 1.29 |
| TRINITY_DN48142_c4_g1 | 14.14 | 12.09 | 10.74 | 11.42 | 9.43 | 7.79 | 7.91 | 13.00 | 8.06 | 12.60 | 6.97 |
| TRINITY_DN48186_c0_g1 | 33.34 | 24.33 | 9.36 | 11.06 | 5.93 | 4.04 | 3.40 | 3.29 | 3.24 | 2.79 | 4.94 |
| TRINITY_DN48196_c3_g1 | 26.80 | 22.85 | 22.26 | 30.54 | 29.23 | 28.70 | 18.53 | 17.97 | 18.31 | 20.61 | 10.23 |
| TRINITY_DN48198_c0_g1 | 0.89 | 0.73 | 1.01 | 1.12 | 2.53 | 1.48 | 2.05 | 3.04 | 4.46 | 4.64 | 0.45 |
| TRINITY_DN48198_c0_g2 | 16.72 | 17.29 | 15.03 | 14.74 | 27.13 | 26.99 | 27.30 | 49.80 | 46.61 | 51.77 | 16.62 |
| TRINITY_DN48225_c0_g2 | 1.66 | 4.41 | 1.82 | 3.91 | 7.66 | 7.47 | 9.01 | 9.87 | 17.19 | 13.77 | 10.58 |
| TRINITY_DN48225_c0_g7 | 3.37 | 3.32 | 2.84 | 9.72 | 10.53 | 7.12 | 3.30 | 3.42 | 8.42 | 2.23 | 0.00 |
| TRINITY_DN48325_c2_g1 | 14.99 | 11.97 | 11.31 | 12.70 | 16.68 | 13.39 | 8.60 | 7.58 | 9.32 | 5.41 | 6.96 |
| TRINITY_DN48347_c1_g1 | 5.02 | 4.86 | 2.34 | 2.42 | 3.60 | 3.30 | 3.53 | 1.93 | 3.68 | 10.87 | 3.67 |
| TRINITY_DN48387_c4_g3 | 2.94 | 2.51 | 1.90 | 2.33 | 3.18 | 1.91 | 0.67 | 1.02 | 1.04 | 0.59 | 2.46 |
| TRINITY_DN48522_c6_g1 | 11.39 | 10.97 | 9.52 | 12.73 | 10.45 | 10.29 | 10.37 | 9.14 | 11.62 | 7.08 | 5.57 |
| TRINITY_DN48538_c1_g1 | 17.79 | 8.50 | 2.80 | 3.10 | 2.89 | 0.90 | 0.69 | 1.10 | 2.72 | 0.10 | 5.01 |
| TRINITY_DN48636_c0_g2 | 6.55 | 5.34 | 1.97 | 2.78 | 2.92 | 1.25 | 0.54 | 1.42 | 7.89 | 9.34 | 17.00 |
| TRINITY_DN48655_c0_g1 | 5.94 | 4.04 | 2.37 | 2.90 | 3.42 | 3.70 | 2.23 | 2.35 | 3.14 | 2.25 | 4.89 |
| TRINITY_DN48655_c1_g1 | 24.26 | 16.86 | 13.02 | 9.69 | 7.56 | 5.89 | 4.37 | 6.36 | 5.88 | 5.11 | 9.18 |
| TRINITY_DN48751_c3_g1 | 7.63 | 6.03 | 3.82 | 6.44 | 12.04 | 9.63 | 7.54 | 16.25 | 46.91 | 43.13 | 100.45 |
| TRINITY_DN48751_c3_g2 | 10.81 | 11.01 | 8.35 | 7.94 | 7.40 | 7.04 | 8.57 | 11.49 | 13.37 | 10.48 | 8.78 |
| TRINITY_DN48855_c2_g1 | 38.16 | 29.46 | 23.23 | 24.70 | 24.65 | 23.97 | 16.68 | 17.96 | 12.51 | 9.71 | 16.90 |
| TRINITY_DN48865_c5_g1 | 14.34 | 11.44 | 12.43 | 14.57 | 13.74 | 11.83 | 9.50 | 10.98 | 15.58 | 11.20 | 6.43 |
| TRINITY_DN49028_c1_g4 | 1.33 | 3.15 | 2.88 | 4.83 | 4.13 | 8.30 | 3.39 | 2.70 | 1.72 | 1.25 | 1.11 |
| TRINITY_DN49043_c0_g5 | 1.47 | 1.47 | 1.01 | 1.44 | 2.99 | 2.83 | 1.94 | 4.17 | 2.08 | 1.06 | 24.29 |
| TRINITY_DN49099_c0_g1 | 54.72 | 49.11 | 46.44 | 69.21 | 64.01 | 57.85 | 92.87 | 137.60 | 67.81 | 30.70 | 15.86 |
| TRINITY_DN49113_c0_g4 | 15.87 | 15.13 | 14.22 | 6.74 | 3.86 | 4.25 | 0.63 | 0.09 | 0.30 | 0.51 | 0.01 |
| TRINITY_DN49234_c2_g2 | 26.92 | 28.24 | 25.41 | 36.26 | 23.96 | 23.96 | 14.57 | 15.89 | 4.92 | 1.58 | 17.63 |
| TRINITY_DN49263_c0_g1 | 27.08 | 22.46 | 19.45 | 20.82 | 38.11 | 29.93 | 24.48 | 38.16 | 30.91 | 13.56 | 33.35 |
| TRINITY_DN49263_c0_g2 | 6.70 | 7.19 | 5.44 | 6.65 | 6.65 | 7.59 | 6.78 | 8.83 | 7.68 | 4.30 | 2.52 |
| TRINITY_DN49389_c1_g1 | 188.49 | 202.90 | 190.56 | 219.56 | 153.89 | 187.61 | 125.44 | 83.32 | 69.61 | 49.74 | 4.66 |
| TRINITY_DN49422_c1_g2 | 2.98 | 2.63 | 2.81 | 4.96 | 7.66 | 5.75 | 3.76 | 4.50 | 8.79 | 3.30 | 0.30 |
| TRINITY_DN49427_c0_g10 | 44.56 | 41.99 | 46.11 | 48.97 | 47.82 | 43.69 | 27.52 | 24.92 | 34.82 | 11.13 | 3.63 |
| TRINITY_DN49505_c0_g1 | 9.21 | 8.53 | 6.94 | 8.90 | 9.11 | 8.21 | 6.00 | 8.20 | 6.58 | 6.24 | 5.10 |
| TRINITY_DN49628_c0_g3 | 10.35 | 9.40 | 8.58 | 9.70 | 8.06 | 7.57 | 8.38 | 6.83 | 8.04 | 6.09 | 3.76 |
| TRINITY_DN49778_c0_g2 | 27.83 | 26.31 | 21.78 | 22.94 | 20.29 | 17.54 | 19.46 | 25.89 | 15.98 | 23.70 | 7.32 |
| TRINITY_DN49855_c0_g2 | 13.31 | 10.78 | 8.39 | 10.48 | 16.07 | 19.42 | 11.79 | 2.50 | 7.61 | 3.01 | 0.13 |
| TRINITY_DN49923_c0_g1 | 20.34 | 19.77 | 17.13 | 21.86 | 15.93 | 18.41 | 16.46 | 13.72 | 14.91 | 11.04 | 4.02 |
| TRINITY_DN50057_c0_g3 | 9.83 | 11.11 | 10.70 | 13.41 | 13.43 | 12.82 | 11.10 | 12.07 | 18.32 | 12.92 | 15.95 |
| TRINITY_DN50138_c1_g6 | 17.27 | 24.49 | 19.46 | 14.63 | 23.00 | 21.42 | 14.94 | 28.09 | 95.81 | 229.40 | 296.92 |
| TRINITY_DN50211_c3_g1 | 43.88 | 34.94 | 33.03 | 30.60 | 30.39 | 20.81 | 18.46 | 31.52 | 18.71 | 14.32 | 8.52 |
| TRINITY_DN50381_c3_g1 | 16.59 | 11.73 | 4.71 | 2.70 | 1.16 | 0.91 | 1.60 | 2.16 | 1.32 | 6.67 | 11.03 |
| TRINITY_DN50434_c0_g3 | 30.10 | 30.09 | 29.78 | 29.21 | 34.71 | 23.26 | 19.72 | 47.28 | 33.92 | 32.18 | 49.94 |
| TRINITY_DN50492_c2_g2 | 4.85 | 4.90 | 3.49 | 5.37 | 5.65 | 4.94 | 3.82 | 2.42 | 4.51 | 1.96 | 7.20 |
| TRINITY_DN50577_c1_g3 | 9.20 | 4.27 | 3.29 | 4.23 | 1.66 | 1.26 | 0.73 | 0.76 | 0.58 | 0.51 | 0.27 |
| TRINITY_DN50641_c1_g1 | 59.39 | 47.54 | 35.03 | 39.29 | 72.61 | 55.05 | 29.63 | 28.21 | 27.79 | 13.51 | 17.25 |
| TRINITY_DN50736_c4_g1 | 24.15 | 16.27 | 7.50 | 4.95 | 2.59 | 1.21 | 1.37 | 2.58 | 1.36 | 0.72 | 0.51 |
| TRINITY_DN50949_c0_g1 | 99.42 | 145.17 | 69.63 | 36.02 | 97.32 | 114.48 | 113.96 | 356.60 | 221.76 | 467.98 | 149.35 |
| TRINITY_DN51008_c5_g1 | 55.03 | 43.42 | 51.05 | 50.51 | 58.54 | 37.41 | 33.53 | 52.34 | 31.41 | 22.11 | 14.07 |
| TRINITY_DN51168_c5_g1 | 6.45 | 6.94 | 7.75 | 8.68 | 11.20 | 8.98 | 7.39 | 7.88 | 7.87 | 5.53 | 4.12 |
| TRINITY_DN51278_c0_g2 | 6.71 | 5.21 | 4.46 | 6.08 | 11.31 | 7.62 | 6.84 | 9.80 | 12.58 | 9.37 | 9.85 |
| TRINITY_DN51670_c1_g5 | 57.02 | 53.40 | 39.82 | 63.13 | 54.08 | 45.52 | 67.95 | 77.55 | 78.77 | 42.37 | 33.63 |
| TRINITY_DN51786_c2_g1 | 1.65 | 2.90 | 2.59 | 1.34 | 1.25 | 0.95 | 0.54 | 0.82 | 2.51 | 4.66 | 55.48 |
| TRINITY_DN51786_c3_g1 | 22.41 | 50.31 | 28.14 | 24.72 | 27.83 | 68.30 | 39.78 | 30.31 | 66.00 | 203.74 | 238.73 |
| TRINITY_DN51786_c3_g2 | 4.84 | 8.06 | 11.85 | 17.18 | 39.38 | 42.86 | 36.62 | 41.81 | 41.10 | 16.76 | 1.57 |
| TRINITY_DN51786_c3_g4 | 3.57 | 1.46 | 0.77 | 1.06 | 1.07 | 0.94 | 0.70 | 4.17 | 6.88 | 61.07 | 599.80 |
| TRINITY_DN51869_c1_g1 | 1092.90 | 1063.96 | 1569.38 | 1224.31 | 403.71 | 394.53 | 13.72 | 4.60 | 4.03 | 1.42 | 1.51 |
| TRINITY_DN51873_c0_g1 | 17.78 | 15.26 | 9.64 | 13.87 | 12.86 | 14.03 | 11.60 | 10.49 | 16.37 | 10.21 | 7.73 |
| TRINITY_DN51929_c3_g1 | 18.77 | 20.80 | 16.60 | 27.29 | 26.73 | 23.78 | 10.56 | 5.81 | 19.38 | 2.69 | 1.04 |
| TRINITY_DN51950_c0_g1 | 9.81 | 8.71 | 7.42 | 6.32 | 12.65 | 4.71 | 1.86 | 5.89 | 6.69 | 27.15 | 10.28 |
| TRINITY_DN52006_c1_g1 | 18.63 | 15.11 | 15.87 | 16.24 | 17.77 | 13.65 | 9.02 | 15.33 | 14.45 | 12.45 | 21.96 |
| TRINITY_DN52195_c0_g1 | 52.50 | 47.78 | 46.08 | 39.42 | 37.00 | 31.31 | 25.12 | 33.99 | 40.36 | 69.33 | 79.90 |
| TRINITY_DN52314_c3_g1 | 20.00 | 13.08 | 8.45 | 22.55 | 5.72 | 3.65 | 1.16 | 0.18 | 0.30 | 0.12 | 0.05 |
| TRINITY_DN52332_c1_g5 | 61.96 | 54.09 | 26.13 | 15.03 | 7.30 | 7.33 | 1.61 | 0.60 | 1.89 | 1.40 | 0.06 |
| TRINITY_DN52387_c8_g1 | 28.10 | 30.16 | 28.56 | 28.87 | 20.65 | 20.47 | 17.67 | 16.02 | 15.37 | 11.04 | 29.52 |
| TRINITY_DN52625_c0_g2 | 4.60 | 5.03 | 5.25 | 5.37 | 8.06 | 7.90 | 6.33 | 4.48 | 3.62 | 0.54 | 0.11 |
| TRINITY_DN52768_c0_g1 | 16.45 | 22.07 | 26.71 | 23.82 | 26.14 | 23.89 | 17.00 | 32.34 | 15.58 | 16.40 | 20.09 |
| TRINITY_DN52789_c0_g3 | 1.23 | 1.79 | 2.46 | 1.78 | 1.95 | 2.20 | 0.95 | 2.43 | 2.90 | 1.57 | 1.54 |
| TRINITY_DN52803_c1_g1 | 3.56 | 3.25 | 3.14 | 4.34 | 4.23 | 4.90 | 5.55 | 5.14 | 5.80 | 6.10 | 2.78 |
| TRINITY_DN52814_c1_g1 | 47.98 | 37.39 | 32.39 | 36.07 | 24.75 | 25.35 | 20.84 | 19.46 | 16.05 | 11.41 | 17.05 |
| TRINITY_DN52830_c0_g2 | 62.88 | 52.35 | 42.55 | 42.95 | 50.59 | 43.07 | 31.41 | 36.53 | 41.39 | 41.80 | 16.91 |
| TRINITY_DN52872_c4_g1 | 26.13 | 21.10 | 12.77 | 8.82 | 6.56 | 5.09 | 5.29 | 7.12 | 6.63 | 4.80 | 4.17 |
| TRINITY_DN53009_c0_g2 | 30.13 | 27.63 | 25.51 | 35.15 | 40.30 | 43.42 | 32.17 | 27.41 | 44.50 | 26.26 | 11.31 |
| TRINITY_DN53121_c5_g1 | 13.82 | 13.61 | 10.80 | 12.49 | 10.41 | 9.30 | 8.31 | 8.14 | 13.07 | 8.77 | 3.96 |
| TRINITY_DN53142_c2_g1 | 13.12 | 12.05 | 6.71 | 11.46 | 8.01 | 8.51 | 8.62 | 9.40 | 9.22 | 8.17 | 1.33 |
| TRINITY_DN53195_c0_g2 | 5.01 | 5.08 | 4.70 | 5.94 | 7.21 | 6.66 | 10.30 | 14.28 | 7.10 | 8.07 | 2.31 |
| TRINITY_DN53211_c3_g1 | 15.00 | 9.96 | 4.31 | 6.17 | 4.86 | 2.33 | 1.36 | 0.84 | 1.50 | 1.25 | 5.06 |
| TRINITY_DN53211_c3_g5 | 24.13 | 22.71 | 28.95 | 29.59 | 19.34 | 16.51 | 12.76 | 14.90 | 8.28 | 6.67 | 0.46 |
| TRINITY_DN53443_c5_g2 | 7.52 | 8.47 | 4.12 | 6.40 | 5.24 | 4.95 | 3.24 | 2.00 | 1.89 | 1.48 | 0.25 |
| TRINITY_DN53475_c0_g2 | 40.16 | 33.35 | 23.56 | 24.53 | 25.99 | 20.67 | 11.86 | 10.96 | 13.20 | 4.44 | 7.75 |
| TRINITY_DN53486_c1_g4 | 23.37 | 23.29 | 22.24 | 24.65 | 37.42 | 24.25 | 7.60 | 8.03 | 12.97 | 4.13 | 5.89 |
| TRINITY_DN53627_c4_g1 | 131.35 | 106.95 | 81.01 | 81.12 | 60.34 | 70.58 | 53.12 | 53.99 | 37.68 | 30.06 | 18.72 |
| TRINITY_DN53654_c0_g2 | 1.72 | 3.48 | 3.59 | 2.30 | 3.71 | 2.87 | 2.45 | 2.24 | 1.06 | 11.53 | 0.76 |
| TRINITY_DN53713_c0_g2 | 11.30 | 9.76 | 9.48 | 9.87 | 10.00 | 10.19 | 8.65 | 11.54 | 8.55 | 6.24 | 4.86 |
| TRINITY_DN53764_c0_g1 | 29.48 | 24.49 | 20.30 | 21.17 | 12.45 | 15.39 | 12.24 | 8.47 | 11.26 | 12.22 | 4.19 |
| TRINITY_DN53802_c2_g1 | 4.93 | 5.79 | 5.93 | 7.34 | 9.48 | 6.09 | 8.86 | 22.42 | 9.19 | 9.09 | 4.35 |
| TRINITY_DN53802_c2_g2 | 28.56 | 34.90 | 32.24 | 49.35 | 41.77 | 45.20 | 71.42 | 80.06 | 68.04 | 66.71 | 28.04 |
| TRINITY_DN53960_c5_g1 | 265.26 | 205.36 | 96.46 | 134.94 | 91.96 | 96.93 | 81.84 | 86.04 | 133.86 | 92.73 | 289.11 |
| TRINITY_DN54084_c0_g1 | 86.35 | 76.70 | 58.61 | 58.40 | 41.57 | 49.43 | 21.99 | 17.78 | 12.97 | 4.53 | 0.30 |
| TRINITY_DN54094_c6_g1 | 6.57 | 7.26 | 4.54 | 4.40 | 1.49 | 1.67 | 0.45 | 0.37 | 0.17 | 0.19 | 3.66 |
| TRINITY_DN54094_c6_g6 | 8.07 | 7.64 | 6.40 | 12.08 | 7.02 | 7.35 | 6.66 | 2.86 | 4.28 | 2.33 | 7.26 |
| TRINITY_DN54160_c4_g1 | 15.17 | 10.38 | 9.87 | 8.29 | 9.56 | 7.21 | 4.79 | 10.81 | 5.77 | 5.93 | 7.64 |
| TRINITY_DN54160_c5_g2 | 3.50 | 2.89 | 3.01 | 3.05 | 4.35 | 3.02 | 2.33 | 6.04 | 2.51 | 2.36 | 5.94 |
| TRINITY_DN54160_c5_g3 | 15.41 | 14.86 | 13.01 | 14.75 | 13.91 | 14.58 | 11.79 | 13.05 | 13.51 | 9.98 | 5.55 |
| TRINITY_DN54228_c1_g3 | 12.11 | 11.42 | 11.16 | 10.61 | 15.27 | 11.83 | 16.18 | 12.88 | 11.47 | 8.90 | 3.75 |
| TRINITY_DN54394_c4_g2 | 6.57 | 4.69 | 4.66 | 4.04 | 5.83 | 3.60 | 2.32 | 3.47 | 2.54 | 1.27 | 0.47 |
| TRINITY_DN54625_c1_g2 | 13.56 | 13.33 | 12.47 | 13.32 | 12.52 | 13.81 | 10.58 | 10.76 | 10.24 | 7.15 | 4.81 |
| TRINITY_DN54686_c2_g2 | 5.12 | 7.36 | 5.15 | 9.07 | 12.07 | 43.36 | 13.29 | 20.40 | 25.43 | 97.80 | 48.99 |
| TRINITY_DN54716_c0_g2 | 73.77 | 68.32 | 38.51 | 50.41 | 40.09 | 38.91 | 22.69 | 20.94 | 52.74 | 286.20 | 163.32 |
| TRINITY_DN54856_c1_g1 | 7.32 | 5.98 | 6.54 | 7.25 | 7.31 | 6.12 | 5.79 | 6.63 | 7.22 | 7.46 | 17.63 |
| TRINITY_DN54873_c4_g1 | 4.71 | 4.87 | 6.34 | 5.41 | 11.64 | 10.79 | 12.44 | 9.72 | 6.54 | 1.65 | 17.50 |
| TRINITY_DN54948_c0_g1 | 8.50 | 6.64 | 6.97 | 8.66 | 11.20 | 11.58 | 3.05 | 0.40 | 1.11 | 0.66 | 0.01 |
| TRINITY_DN55033_c3_g1 | 0.86 | 0.52 | 1.47 | 1.60 | 2.95 | 2.40 | 2.03 | 4.97 | 4.16 | 3.33 | 7.40 |
| TRINITY_DN55049_c4_g2 | 17.18 | 16.19 | 12.12 | 15.95 | 7.87 | 9.79 | 3.25 | 2.05 | 1.10 | 0.48 | 2.03 |
| TRINITY_DN55390_c0_g1 | 4.79 | 6.48 | 8.44 | 27.03 | 75.64 | 99.93 | 126.43 | 90.00 | 231.33 | 91.61 | 3.77 |
| TRINITY_DN55482_c0_g1 | 6.03 | 8.46 | 4.71 | 5.88 | 2.82 | 5.20 | 1.74 | 1.01 | 1.19 | 34.88 | 4.69 |
| TRINITY_DN55482_c1_g2 | 80.71 | 90.41 | 171.43 | 74.88 | 39.22 | 53.01 | 4.87 | 2.57 | 1.69 | 0.31 | 0.00 |
| TRINITY_DN55496_c2_g2 | 3.92 | 3.41 | 9.51 | 12.09 | 23.38 | 13.07 | 16.04 | 24.39 | 16.49 | 21.97 | 10.33 |
| TRINITY_DN55705_c1_g1 | 99.19 | 80.57 | 46.44 | 60.94 | 58.50 | 53.42 | 44.12 | 57.64 | 67.64 | 45.68 | 27.62 |
| TRINITY_DN55709_c0_g1 | 190.34 | 151.86 | 96.06 | 107.61 | 76.30 | 49.07 | 55.93 | 57.63 | 73.32 | 15.86 | 5.04 |
| TRINITY_DN55710_c3_g2 | 220.22 | 150.87 | 106.21 | 110.98 | 111.35 | 76.23 | 77.76 | 42.14 | 83.21 | 15.94 | 1.53 |
| TRINITY_DN55858_c3_g1 | 44.38 | 30.47 | 24.47 | 18.38 | 7.57 | 6.27 | 1.82 | 0.87 | 0.67 | 0.15 | 0.01 |
| TRINITY_DN55923_c0_g2 | 11.59 | 10.14 | 4.58 | 4.49 | 2.50 | 2.17 | 2.17 | 2.49 | 7.27 | 15.20 | 0.48 |
| TRINITY_DN55930_c2_g2 | 22.49 | 14.12 | 10.97 | 16.92 | 8.85 | 10.88 | 6.56 | 6.40 | 14.89 | 15.27 | 18.72 |
| TRINITY_DN56007_c2_g2 | 13.58 | 11.31 | 12.11 | 13.61 | 13.52 | 10.75 | 6.93 | 6.67 | 9.16 | 7.99 | 8.11 |
| TRINITY_DN56009_c2_g2 | 5.40 | 7.30 | 12.33 | 13.02 | 9.22 | 10.49 | 0.94 | 0.71 | 3.74 | 3.88 | 0.01 |
| TRINITY_DN56107_c1_g5 | 25.12 | 17.45 | 7.83 | 15.21 | 11.34 | 13.89 | 15.23 | 7.05 | 10.84 | 4.38 | 0.67 |
| TRINITY_DN56139_c2_g2 | 93.33 | 86.87 | 34.79 | 32.41 | 21.47 | 17.19 | 17.38 | 31.39 | 29.25 | 21.74 | 60.47 |
| TRINITY_DN56238_c0_g1 | 21.86 | 17.18 | 17.74 | 19.88 | 17.40 | 16.80 | 17.61 | 16.11 | 16.98 | 12.42 | 9.17 |
| TRINITY_DN56295_c2_g1 | 19.49 | 17.06 | 10.48 | 14.19 | 4.00 | 1.65 | 0.44 | 0.33 | 0.17 | 0.07 | 0.07 |
| TRINITY_DN56491_c3_g2 | 77.03 | 57.50 | 60.87 | 49.32 | 63.24 | 47.95 | 38.82 | 96.17 | 110.63 | 85.66 | 46.46 |
| TRINITY_DN56526_c1_g3 | 4.31 | 3.41 | 3.42 | 4.71 | 2.14 | 3.71 | 3.18 | 1.17 | 2.15 | 1.25 | 0.17 |
| TRINITY_DN56646_c2_g9 | 42.47 | 46.66 | 86.48 | 37.84 | 20.19 | 21.57 | 2.22 | 1.15 | 0.33 | 0.13 | 0.23 |
| TRINITY_DN56660_c1_g1 | 9.13 | 7.37 | 8.22 | 8.76 | 9.64 | 10.35 | 13.06 | 12.17 | 11.33 | 12.33 | 5.20 |
| TRINITY_DN56716_c0_g1 | 75.77 | 53.47 | 57.75 | 43.64 | 45.48 | 33.63 | 27.37 | 31.45 | 28.64 | 19.12 | 34.18 |
| TRINITY_DN56716_c0_g2 | 34.26 | 28.48 | 24.26 | 41.69 | 39.10 | 50.48 | 57.22 | 42.72 | 64.48 | 29.41 | 26.40 |
| TRINITY_DN56768_c2_g2 | 3.17 | 5.41 | 6.43 | 6.10 | 3.81 | 10.32 | 1.87 | 0.55 | 0.12 | 0.73 | 0.00 |
| TRINITY_DN56768_c2_g5 | 20.89 | 21.63 | 9.51 | 8.78 | 5.20 | 13.31 | 25.48 | 26.27 | 26.31 | 27.74 | 71.22 |
| TRINITY_DN56909_c2_g1 | 48.65 | 43.48 | 28.23 | 18.93 | 19.42 | 16.52 | 6.31 | 7.17 | 6.45 | 4.49 | 7.21 |
| TRINITY_DN56909_c2_g2 | 6.45 | 4.39 | 2.43 | 1.54 | 2.26 | 1.70 | 2.06 | 8.08 | 12.98 | 1.58 | 1.53 |
| TRINITY_DN56909_c2_g4 | 13.98 | 12.72 | 8.97 | 8.36 | 4.50 | 3.29 | 2.37 | 5.02 | 3.59 | 4.06 | 4.81 |
| TRINITY_DN56966_c2_g1 | 57.46 | 49.39 | 44.05 | 42.25 | 45.67 | 35.94 | 25.15 | 32.24 | 25.77 | 20.23 | 5.78 |
| TRINITY_DN56966_c2_g2 | 68.45 | 58.14 | 48.93 | 59.97 | 57.72 | 51.99 | 47.84 | 65.09 | 50.47 | 40.16 | 13.84 |
| TRINITY_DN56987_c2_g1 | 2.23 | 2.71 | 2.52 | 3.41 | 4.12 | 4.02 | 4.41 | 6.02 | 2.78 | 3.33 | 6.91 |
| TRINITY_DN57072_c1_g2 | 13.88 | 5.93 | 2.64 | 3.01 | 1.60 | 0.78 | 0.77 | 2.29 | 0.65 | 0.38 | 0.09 |
| TRINITY_DN57384_c1_g3 | 188.49 | 162.57 | 91.21 | 126.81 | 78.83 | 71.97 | 73.15 | 83.64 | 40.37 | 9.58 | 0.69 |
| TRINITY_DN57395_c1_g5 | 5.57 | 6.91 | 9.24 | 10.99 | 7.32 | 5.41 | 4.67 | 5.32 | 2.26 | 3.83 | 18.27 |
| TRINITY_DN57411_c0_g1 | 8.74 | 6.50 | 8.80 | 10.86 | 9.05 | 8.83 | 9.39 | 11.79 | 7.55 | 5.59 | 6.07 |
| TRINITY_DN57490_c1_g1 | 4.27 | 4.02 | 3.72 | 3.81 | 5.56 | 3.76 | 3.15 | 5.65 | 3.74 | 6.44 | 7.73 |
| TRINITY_DN57584_c3_g2 | 18.43 | 16.21 | 17.70 | 18.85 | 23.96 | 29.84 | 28.95 | 38.65 | 31.17 | 41.95 | 54.17 |
| TRINITY_DN57617_c1_g5 | 37.33 | 34.98 | 32.69 | 33.95 | 29.28 | 28.94 | 23.66 | 22.13 | 32.40 | 37.22 | 5.23 |
| TRINITY_DN57695_c1_g2 | 45.72 | 34.35 | 34.48 | 33.40 | 31.71 | 28.20 | 24.52 | 10.37 | 6.49 | 1.17 | 58.78 |
| TRINITY_DN57900_c5_g1 | 12.74 | 11.08 | 9.39 | 10.45 | 10.12 | 9.78 | 9.30 | 13.76 | 9.44 | 8.45 | 7.33 |
| TRINITY_DN58023_c2_g1 | 23.06 | 18.58 | 17.38 | 17.41 | 16.27 | 12.63 | 10.14 | 10.42 | 7.42 | 5.57 | 1.38 |
| TRINITY_DN58023_c2_g2 | 72.38 | 85.70 | 52.53 | 64.24 | 28.06 | 20.59 | 11.78 | 6.02 | 5.47 | 2.72 | 0.00 |
| TRINITY_DN58023_c2_g4 | 53.71 | 42.68 | 16.89 | 32.10 | 20.74 | 13.67 | 9.61 | 11.22 | 4.14 | 3.78 | 0.05 |
| TRINITY_DN58023_c2_g8 | 25.80 | 20.05 | 28.09 | 18.66 | 12.10 | 13.39 | 12.49 | 9.37 | 5.35 | 2.87 | 1.30 |
| TRINITY_DN58036_c6_g2 | 30.36 | 29.19 | 31.92 | 19.29 | 20.37 | 24.45 | 13.19 | 9.62 | 14.23 | 4.26 | 84.59 |
| TRINITY_DN58102_c2_g4 | 3.11 | 2.26 | 3.12 | 3.65 | 5.24 | 4.83 | 5.29 | 1.15 | 6.68 | 2.43 | 1.42 |
| TRINITY_DN58195_c1_g1 | 7.25 | 7.08 | 7.00 | 8.63 | 7.97 | 9.82 | 9.73 | 9.87 | 10.00 | 9.57 | 8.71 |
| TRINITY_DN58195_c1_g3 | 26.46 | 19.86 | 18.06 | 22.46 | 17.14 | 16.70 | 14.64 | 13.99 | 14.66 | 12.72 | 9.56 |
| TRINITY_DN58195_c1_g4 | 8.61 | 8.32 | 7.51 | 7.93 | 7.04 | 6.46 | 4.69 | 7.17 | 11.28 | 9.95 | 16.72 |
| TRINITY_DN58218_c0_g1 | 9.38 | 7.74 | 6.95 | 9.82 | 11.61 | 10.62 | 8.54 | 6.06 | 9.67 | 8.18 | 9.85 |
| TRINITY_DN58218_c0_g4 | 27.62 | 25.35 | 22.88 | 32.73 | 21.51 | 25.31 | 24.91 | 18.73 | 21.04 | 15.56 | 2.74 |
| TRINITY_DN58239_c6_g1 | 12.81 | 11.24 | 9.89 | 11.10 | 12.07 | 9.38 | 9.47 | 14.66 | 11.41 | 10.58 | 5.59 |
| TRINITY_DN58242_c1_g1 | 14.56 | 15.20 | 16.48 | 19.45 | 19.56 | 18.35 | 15.25 | 14.92 | 14.53 | 14.21 | 4.72 |
| TRINITY_DN58264_c0_g1 | 23.59 | 26.24 | 52.51 | 104.28 | 420.42 | 162.79 | 126.66 | 211.07 | 312.56 | 176.16 | 0.51 |
| TRINITY_DN58351_c1_g1 | 17.78 | 13.13 | 7.40 | 4.93 | 6.05 | 6.46 | 2.25 | 2.00 | 3.05 | 3.83 | 1.86 |
| TRINITY_DN58351_c1_g2 | 2.39 | 3.43 | 4.09 | 3.49 | 5.23 | 5.75 | 1.21 | 0.69 | 0.16 | 0.53 | 0.00 |
| TRINITY_DN58371_c2_g1 | 8.35 | 9.34 | 10.77 | 12.79 | 12.86 | 12.47 | 17.07 | 14.81 | 12.33 | 12.74 | 5.71 |
| TRINITY_DN58423_c2_g3 | 13.07 | 12.92 | 4.05 | 2.98 | 1.17 | 0.77 | 0.58 | 0.79 | 2.13 | 0.03 | 0.00 |
| TRINITY_DN58424_c0_g3 | 4.61 | 5.64 | 7.49 | 0.63 | 1.64 | 0.39 | 2.66 | 15.28 | 9.90 | 2.87 | 8.89 |
| TRINITY_DN58424_c1_g1 | 22.02 | 19.46 | 20.42 | 23.07 | 22.84 | 16.82 | 13.82 | 16.48 | 25.98 | 13.89 | 56.21 |
| TRINITY_DN58480_c2_g4 | 1.91 | 2.00 | 2.41 | 2.43 | 2.40 | 2.44 | 1.32 | 1.55 | 1.79 | 2.56 | 0.42 |
| TRINITY_DN58494_c2_g1 | 4.13 | 4.84 | 6.56 | 1.90 | 1.95 | 1.80 | 0.79 | 0.57 | 0.79 | 2.67 | 0.01 |
| TRINITY_DN58494_c2_g8 | 1.89 | 2.65 | 7.71 | 3.82 | 10.44 | 7.47 | 3.31 | 1.49 | 6.26 | 5.71 | 0.13 |
| TRINITY_DN58499_c0_g1 | 2.46 | 1.86 | 1.23 | 2.61 | 3.47 | 2.19 | 1.58 | 2.81 | 3.97 | 1.56 | 4.85 |
| TRINITY_DN58576_c2_g2 | 20.75 | 17.03 | 12.88 | 9.79 | 8.95 | 8.43 | 6.33 | 10.27 | 11.88 | 12.45 | 50.09 |
| TRINITY_DN58690_c0_g1 | 6.93 | 9.09 | 5.07 | 13.56 | 20.42 | 17.47 | 44.83 | 38.32 | 62.85 | 37.68 | 14.46 |
| TRINITY_DN59282_c0_g2 | 41.42 | 26.85 | 33.91 | 25.21 | 27.97 | 27.13 | 50.25 | 122.09 | 18.17 | 36.51 | 36.63 |
| TRINITY_DN59319_c0_g3 | 18.84 | 17.40 | 29.85 | 37.55 | 43.55 | 47.03 | 48.85 | 53.24 | 45.71 | 31.53 | 42.93 |
| TRINITY_DN59349_c0_g4 | 10.38 | 8.71 | 8.83 | 8.96 | 6.98 | 6.74 | 4.88 | 4.91 | 3.80 | 2.45 | 0.50 |
| TRINITY_DN59349_c1_g1 | 5.92 | 4.92 | 2.47 | 5.23 | 3.22 | 2.26 | 0.36 | 1.76 | 0.70 | 0.00 | 4.45 |
| TRINITY_DN59356_c0_g1 | 26.87 | 31.64 | 20.31 | 21.56 | 19.94 | 16.22 | 15.14 | 15.10 | 18.71 | 10.37 | 9.09 |
| TRINITY_DN59551_c1_g2 | 2.70 | 2.61 | 2.13 | 3.08 | 2.91 | 3.42 | 2.84 | 1.65 | 3.86 | 2.02 | 1.44 |
| TRINITY_DN59676_c2_g2 | 6.12 | 6.87 | 7.89 | 7.22 | 7.01 | 8.41 | 4.45 | 3.02 | 2.70 | 0.65 | 8.04 |
| TRINITY_DN59706_c0_g2 | 2.77 | 2.31 | 1.46 | 2.13 | 2.75 | 2.03 | 1.77 | 2.77 | 2.64 | 1.75 | 22.59 |
| TRINITY_DN59818_c2_g1 | 8.44 | 11.19 | 18.60 | 23.39 | 22.29 | 22.44 | 15.17 | 13.22 | 19.68 | 2.73 | 0.06 |
| TRINITY_DN59829_c1_g4 | 0.78 | 1.17 | 0.94 | 2.29 | 10.11 | 11.79 | 17.95 | 24.54 | 44.05 | 61.58 | 102.18 |
| TRINITY_DN59889_c5_g1 | 38.45 | 47.21 | 45.78 | 32.45 | 14.76 | 22.39 | 18.64 | 10.17 | 16.22 | 6.61 | 10.66 |
| TRINITY_DN59892_c0_g4 | 3.10 | 7.10 | 2.17 | 10.03 | 21.69 | 9.53 | 27.62 | 34.23 | 41.22 | 18.84 | 0.04 |
| TRINITY_DN59922_c0_g2 | 6.48 | 5.53 | 5.23 | 6.78 | 7.35 | 7.14 | 5.77 | 5.28 | 6.66 | 4.02 | 4.51 |
| TRINITY_DN59923_c0_g3 | 9.66 | 10.26 | 11.83 | 10.93 | 22.07 | 10.32 | 14.35 | 61.46 | 19.56 | 8.40 | 1.01 |
| TRINITY_DN60053_c0_g1 | 140.94 | 120.43 | 144.63 | 134.34 | 135.11 | 110.24 | 83.96 | 141.89 | 64.13 | 37.86 | 9.07 |
| TRINITY_DN60136_c2_g2 | 6.01 | 5.50 | 4.84 | 7.09 | 6.11 | 6.65 | 6.64 | 4.46 | 7.23 | 4.22 | 1.67 |
| TRINITY_DN60207_c1_g2 | 27.84 | 22.08 | 21.17 | 21.91 | 29.40 | 21.34 | 15.42 | 29.89 | 21.08 | 15.61 | 41.47 |
| TRINITY_DN60281_c4_g1 | 4.72 | 3.83 | 3.15 | 3.87 | 14.73 | 9.77 | 10.32 | 19.21 | 17.05 | 17.02 | 8.08 |
| TRINITY_DN60281_c4_g2 | 9.36 | 8.07 | 7.80 | 9.73 | 7.85 | 8.13 | 6.44 | 8.93 | 8.13 | 5.74 | 2.83 |
| TRINITY_DN60299_c0_g1 | 3.03 | 2.21 | 1.91 | 1.80 | 2.94 | 2.33 | 1.74 | 2.87 | 2.07 | 1.49 | 4.27 |
| TRINITY_DN60299_c0_g2 | 15.06 | 13.63 | 11.15 | 12.26 | 13.48 | 17.29 | 14.34 | 17.04 | 17.46 | 12.50 | 41.30 |
| TRINITY_DN60442_c0_g1 | 27.80 | 23.94 | 27.89 | 28.84 | 23.34 | 21.22 | 14.70 | 10.96 | 12.50 | 9.27 | 0.74 |
| TRINITY_DN60467_c4_g1 | 360.52 | 353.36 | 269.71 | 252.66 | 102.48 | 96.03 | 11.76 | 6.86 | 23.70 | 7.88 | 2.25 |
| TRINITY_DN60522_c1_g1 | 51.82 | 59.68 | 20.37 | 34.37 | 17.66 | 11.14 | 7.24 | 10.47 | 18.32 | 7.59 | 10.86 |
| TRINITY_DN60560_c2_g1 | 33.61 | 28.07 | 16.47 | 18.46 | 6.93 | 2.29 | 0.69 | 1.56 | 0.18 | 0.43 | 0.12 |
| TRINITY_DN60579_c1_g2 | 7.62 | 7.76 | 7.53 | 10.08 | 8.36 | 8.44 | 8.88 | 9.02 | 12.91 | 10.31 | 6.35 |
| TRINITY_DN60598_c4_g3 | 8.79 | 6.26 | 4.75 | 12.69 | 17.20 | 13.42 | 13.84 | 21.24 | 21.34 | 12.10 | 17.76 |
| TRINITY_DN60612_c2_g2 | 9.73 | 8.24 | 5.43 | 3.43 | 2.60 | 1.69 | 1.43 | 1.15 | 3.50 | 4.81 | 4.41 |
| TRINITY_DN60638_c1_g2 | 27.63 | 25.72 | 13.38 | 24.42 | 18.88 | 16.02 | 15.83 | 24.59 | 31.75 | 20.75 | 19.06 |
| TRINITY_DN60721_c2_g1 | 25.68 | 33.77 | 6.09 | 19.08 | 3.94 | 4.20 | 3.20 | 4.17 | 4.53 | 3.24 | 4.85 |
| TRINITY_DN60754_c0_g1 | 7.75 | 8.54 | 6.77 | 10.08 | 8.02 | 7.21 | 5.05 | 3.71 | 4.60 | 1.82 | 54.21 |
| TRINITY_DN60784_c3_g2 | 36.65 | 37.33 | 36.39 | 43.83 | 82.91 | 60.27 | 45.29 | 44.30 | 75.53 | 41.32 | 17.38 |
| TRINITY_DN60890_c1_g1 | 9.04 | 11.49 | 8.26 | 7.89 | 3.92 | 5.38 | 2.99 | 1.35 | 1.36 | 0.51 | 0.27 |
| TRINITY_DN60908_c0_g1 | 149.17 | 163.67 | 111.13 | 108.43 | 73.92 | 78.28 | 91.87 | 156.37 | 429.19 | 560.04 | 380.76 |
| TRINITY_DN60976_c0_g2 | 10.33 | 8.91 | 8.26 | 7.43 | 5.51 | 8.00 | 6.04 | 3.28 | 3.74 | 1.85 | 0.30 |
| TRINITY_DN60997_c3_g1 | 17.53 | 17.03 | 17.56 | 22.10 | 24.17 | 22.19 | 18.43 | 18.69 | 23.12 | 19.77 | 7.15 |
| TRINITY_DN61123_c0_g1 | 6.63 | 5.57 | 3.30 | 1.66 | 1.95 | 2.39 | 1.45 | 0.66 | 0.65 | 0.62 | 0.20 |
| TRINITY_DN61268_c0_g2 | 11.09 | 9.30 | 8.18 | 10.64 | 10.96 | 9.01 | 7.71 | 8.64 | 8.53 | 5.92 | 12.75 |
| TRINITY_DN61301_c0_g2 | 20.77 | 18.73 | 18.73 | 21.91 | 23.24 | 24.52 | 22.23 | 20.63 | 103.77 | 227.06 | 40.18 |
| TRINITY_DN61420_c2_g1 | 55.19 | 48.01 | 41.11 | 52.29 | 55.48 | 51.35 | 51.14 | 69.93 | 57.02 | 35.97 | 167.91 |
| TRINITY_DN61426_c0_g1 | 7.88 | 7.28 | 6.61 | 9.12 | 7.79 | 7.44 | 6.10 | 4.03 | 5.65 | 3.22 | 2.95 |
| TRINITY_DN61595_c2_g1 | 100.26 | 116.34 | 233.33 | 150.86 | 482.84 | 350.67 | 387.56 | 433.10 | 156.17 | 68.18 | 9.72 |
| TRINITY_DN61619_c1_g8 | 18.18 | 15.37 | 14.71 | 12.50 | 15.87 | 15.31 | 14.63 | 33.03 | 31.36 | 32.06 | 27.81 |
| TRINITY_DN61784_c2_g1 | 6.50 | 7.36 | 5.99 | 7.28 | 6.46 | 6.20 | 4.91 | 3.66 | 6.33 | 5.39 | 4.33 |
| TRINITY_DN61808_c1_g1 | 39.70 | 30.40 | 32.57 | 27.55 | 27.08 | 26.54 | 21.70 | 30.02 | 25.26 | 21.06 | 21.33 |
| TRINITY_DN61808_c1_g2 | 15.12 | 12.46 | 10.69 | 10.58 | 7.61 | 5.90 | 4.18 | 8.07 | 3.17 | 4.08 | 6.64 |
| TRINITY_DN61823_c1_g1 | 8.56 | 7.63 | 6.16 | 7.57 | 4.25 | 5.39 | 2.24 | 3.40 | 7.13 | 20.56 | 2.53 |
| TRINITY_DN61860_c0_g1 | 68.20 | 51.19 | 25.15 | 22.55 | 11.18 | 8.99 | 1.86 | 0.95 | 0.89 | 1.29 | 0.78 |
| TRINITY_DN62017_c1_g2 | 33.81 | 31.07 | 34.90 | 34.07 | 32.04 | 32.39 | 25.88 | 37.19 | 31.47 | 35.47 | 97.03 |
| TRINITY_DN62034_c1_g2 | 3.39 | 4.34 | 3.71 | 3.82 | 5.14 | 4.39 | 3.40 | 3.17 | 4.31 | 3.53 | 5.14 |
| TRINITY_DN62034_c1_g3 | 7.23 | 6.88 | 3.79 | 4.65 | 4.01 | 3.91 | 1.64 | 1.29 | 2.58 | 1.88 | 10.39 |
| TRINITY_DN62078_c2_g2 | 25.76 | 32.14 | 46.88 | 22.14 | 14.00 | 15.80 | 3.21 | 0.62 | 1.15 | 0.96 | 0.21 |
| TRINITY_DN62080_c0_g1 | 16.41 | 26.88 | 13.77 | 19.86 | 21.00 | 11.82 | 27.39 | 5.24 | 8.99 | 20.76 | 10.14 |
| TRINITY_DN62080_c0_g3 | 1.70 | 1.78 | 2.77 | 3.31 | 3.64 | 3.80 | 1.82 | 2.49 | 1.41 | 1.07 | 0.71 |
| TRINITY_DN62080_c0_g5 | 28.45 | 24.79 | 36.90 | 17.48 | 18.34 | 6.66 | 4.71 | 1.21 | 0.78 | 3.33 | 0.99 |
| TRINITY_DN62099_c2_g1 | 4.14 | 3.12 | 2.47 | 4.63 | 4.53 | 2.92 | 2.39 | 5.40 | 2.54 | 3.37 | 1.77 |
| TRINITY_DN62113_c0_g1 | 100.63 | 63.94 | 51.76 | 23.53 | 15.11 | 11.04 | 5.06 | 4.06 | 3.88 | 1.88 | 44.29 |
| TRINITY_DN62316_c1_g7 | 16.07 | 13.01 | 11.53 | 13.71 | 15.50 | 12.02 | 5.81 | 6.69 | 6.24 | 2.75 | 7.76 |
| TRINITY_DN62477_c0_g3 | 1.89 | 7.91 | 2.32 | 4.68 | 1.83 | 11.38 | 4.19 | 8.22 | 17.18 | 83.68 | 9.83 |
| TRINITY_DN62477_c0_g5 | 11.81 | 9.93 | 8.86 | 11.65 | 12.50 | 12.08 | 16.21 | 13.45 | 20.88 | 19.36 | 3.08 |
| TRINITY_DN62481_c0_g1 | 18.36 | 15.17 | 14.01 | 16.29 | 42.57 | 20.41 | 10.54 | 6.20 | 11.67 | 3.07 | 4.33 |
| TRINITY_DN62882_c0_g4 | 11.95 | 10.25 | 21.75 | 11.27 | 9.44 | 10.47 | 1.47 | 0.93 | 0.09 | 0.78 | 0.89 |
| TRINITY_DN62882_c1_g1 | 4.63 | 6.10 | 2.98 | 1.82 | 1.86 | 2.20 | 1.45 | 1.27 | 2.56 | 1.32 | 0.00 |
| TRINITY_DN63021_c2_g1 | 48.73 | 36.97 | 23.48 | 25.08 | 19.69 | 13.78 | 13.78 | 13.75 | 16.77 | 8.53 | 2.59 |
| TRINITY_DN63021_c2_g2 | 44.28 | 30.19 | 23.68 | 32.79 | 33.15 | 43.06 | 49.05 | 28.50 | 102.23 | 52.01 | 3.00 |
| TRINITY_DN63073_c0_g2 | 158.46 | 146.77 | 122.93 | 140.49 | 93.67 | 104.70 | 90.17 | 68.49 | 67.95 | 60.89 | 29.74 |
| TRINITY_DN63282_c4_g1 | 27.31 | 35.36 | 48.08 | 30.02 | 14.10 | 9.27 | 2.33 | 4.64 | 4.93 | 1.14 | 24.13 |
| TRINITY_DN63282_c4_g2 | 110.36 | 94.44 | 77.39 | 91.24 | 41.40 | 38.34 | 4.51 | 2.28 | 0.86 | 0.26 | 0.03 |
| TRINITY_DN63342_c2_g1 | 1.41 | 1.53 | 1.21 | 1.29 | 2.80 | 3.22 | 2.65 | 2.95 | 4.27 | 2.51 | 0.45 |
| TRINITY_DN63368_c3_g1 | 13.61 | 11.50 | 12.50 | 10.21 | 8.18 | 8.08 | 2.30 | 1.82 | 1.30 | 0.97 | 3.73 |
| TRINITY_DN63377_c0_g3 | 15.02 | 13.80 | 11.03 | 13.14 | 13.01 | 12.29 | 9.45 | 6.78 | 9.44 | 8.18 | 5.15 |
| TRINITY_DN63390_c3_g1 | 38.24 | 33.85 | 27.11 | 34.39 | 25.62 | 32.37 | 21.04 | 12.07 | 19.29 | 17.66 | 12.51 |
| TRINITY_DN63596_c0_g1 | 25.98 | 21.98 | 3.97 | 3.02 | 0.68 | 0.86 | 0.31 | 0.16 | 0.85 | 1.36 | 0.62 |
| TRINITY_DN63637_c0_g1 | 3.32 | 3.08 | 0.36 | 1.74 | 0.59 | 1.01 | 2.13 | 1.86 | 3.31 | 25.55 | 0.06 |
| TRINITY_DN63742_c0_g1 | 2.83 | 2.86 | 4.98 | 8.00 | 15.78 | 13.28 | 20.50 | 11.19 | 12.23 | 2.56 | 0.45 |
| TRINITY_DN63854_c4_g1 | 5.56 | 4.15 | 3.46 | 3.91 | 5.43 | 3.97 | 2.63 | 2.23 | 4.15 | 1.83 | 2.49 |
| TRINITY_DN63855_c1_g1 | 0.24 | 0.29 | 0.11 | 0.14 | 0.08 | 0.15 | 0.20 | 0.06 | 7.94 | 4.75 | 204.01 |
| TRINITY_DN63875_c8_g1 | 23.46 | 25.95 | 6.09 | 4.75 | 0.61 | 1.06 | 0.25 | 0.36 | 0.62 | 0.35 | 0.62 |
| TRINITY_DN63986_c0_g1 | 1.06 | 1.23 | 0.57 | 1.16 | 2.56 | 1.20 | 1.10 | 2.22 | 4.05 | 1.69 | 7.61 |
| TRINITY_DN64276_c0_g1 | 29.73 | 27.55 | 25.16 | 27.69 | 21.71 | 23.89 | 18.02 | 11.81 | 7.76 | 4.90 | 3.10 |
| TRINITY_DN64279_c1_g7 | 6.54 | 7.67 | 6.25 | 7.85 | 10.14 | 8.96 | 7.09 | 9.99 | 6.63 | 5.72 | 2.41 |
| TRINITY_DN64343_c2_g1 | 22.37 | 17.28 | 16.66 | 21.15 | 27.66 | 26.05 | 17.61 | 19.89 | 22.14 | 11.77 | 160.42 |
| TRINITY_DN64503_c1_g1 | 58.01 | 47.92 | 7.59 | 15.45 | 8.68 | 10.86 | 13.10 | 19.68 | 30.08 | 24.18 | 12.60 |
| TRINITY_DN64527_c1_g2 | 9.12 | 8.94 | 10.03 | 14.59 | 18.01 | 16.42 | 14.36 | 19.90 | 15.74 | 14.64 | 7.76 |
| TRINITY_DN64527_c1_g3 | 3.73 | 3.72 | 3.49 | 2.80 | 3.39 | 2.85 | 1.74 | 2.33 | 2.12 | 1.47 | 3.31 |
| TRINITY_DN64528_c0_g1 | 13.63 | 6.27 | 3.87 | 3.31 | 2.23 | 1.85 | 0.32 | 0.06 | 0.03 | 0.15 | 36.48 |
| TRINITY_DN64552_c3_g4 | 10.41 | 12.19 | 12.55 | 15.11 | 12.89 | 10.44 | 8.25 | 9.53 | 6.19 | 5.04 | 9.85 |
| TRINITY_DN64602_c2_g1 | 48.46 | 63.32 | 36.86 | 26.67 | 44.57 | 28.54 | 18.23 | 41.03 | 49.59 | 79.28 | 11.84 |
| TRINITY_DN64612_c0_g1 | 36.18 | 38.90 | 36.50 | 50.33 | 42.25 | 47.64 | 51.33 | 29.00 | 34.48 | 25.95 | 9.85 |
| TRINITY_DN64706_c1_g2 | 4.20 | 3.81 | 3.33 | 4.15 | 4.28 | 3.67 | 2.48 | 2.37 | 2.23 | 1.42 | 0.30 |
| TRINITY_DN64707_c0_g1 | 19.62 | 17.85 | 15.46 | 19.75 | 16.77 | 14.45 | 17.18 | 17.70 | 25.57 | 16.77 | 83.83 |
| TRINITY_DN64740_c0_g1 | 3.47 | 2.61 | 0.27 | 7.61 | 9.01 | 3.34 | 22.40 | 28.99 | 5.84 | 21.54 | 1.45 |
| TRINITY_DN64748_c1_g2 | 13.18 | 17.15 | 13.67 | 10.51 | 9.16 | 14.83 | 11.79 | 12.61 | 17.43 | 32.95 | 327.25 |
| TRINITY_DN64754_c5_g1 | 5.57 | 8.81 | 11.80 | 13.93 | 12.09 | 13.48 | 5.38 | 2.08 | 1.59 | 0.36 | 0.44 |
| TRINITY_DN64784_c0_g1 | 52.15 | 47.84 | 45.21 | 49.19 | 69.77 | 62.23 | 67.96 | 113.35 | 80.11 | 25.50 | 0.09 |
| TRINITY_DN64795_c0_g1 | 6.48 | 6.15 | 5.63 | 5.48 | 4.07 | 2.75 | 5.20 | 6.25 | 5.64 | 2.71 | 1.23 |
| TRINITY_DN64795_c0_g3 | 23.64 | 20.00 | 13.12 | 23.80 | 22.59 | 15.26 | 19.25 | 27.42 | 55.64 | 48.93 | 9.50 |
| TRINITY_DN64795_c0_g6 | 6.75 | 4.87 | 1.59 | 5.77 | 6.61 | 4.06 | 6.38 | 10.92 | 14.81 | 23.55 | 0.55 |
| TRINITY_DN64973_c0_g1 | 429.64 | 260.59 | 239.44 | 188.75 | 123.80 | 108.42 | 67.61 | 63.84 | 59.59 | 39.86 | 13.01 |
| TRINITY_DN65018_c0_g1 | 5.13 | 5.06 | 4.96 | 4.01 | 4.45 | 3.09 | 2.48 | 3.82 | 2.94 | 2.76 | 1.07 |
| TRINITY_DN65018_c1_g1 | 7.98 | 6.58 | 4.22 | 7.44 | 12.61 | 11.84 | 10.21 | 6.18 | 13.20 | 4.59 | 1.83 |
| TRINITY_DN65034_c4_g1 | 12.35 | 11.20 | 9.04 | 11.32 | 9.30 | 9.00 | 7.64 | 8.61 | 5.12 | 3.51 | 0.81 |
| TRINITY_DN65102_c0_g1 | 5.29 | 5.83 | 4.73 | 6.26 | 3.93 | 4.29 | 2.09 | 2.47 | 0.72 | 0.23 | 0.01 |
| TRINITY_DN65159_c2_g2 | 56.10 | 51.05 | 46.17 | 61.96 | 69.68 | 65.19 | 47.00 | 45.61 | 56.04 | 28.08 | 15.39 |
| TRINITY_DN65251_c2_g1 | 74.15 | 41.51 | 31.41 | 11.45 | 14.64 | 16.01 | 1.49 | 5.39 | 15.68 | 52.63 | 742.70 |
| TRINITY_DN65251_c3_g2 | 75.06 | 73.85 | 61.43 | 92.61 | 68.48 | 92.10 | 103.77 | 48.31 | 107.25 | 78.10 | 21.92 |
| TRINITY_DN65286_c4_g2 | 8.52 | 8.64 | 8.80 | 12.51 | 11.02 | 12.62 | 13.00 | 10.95 | 11.12 | 10.32 | 4.15 |
| TRINITY_DN65386_c2_g3 | 1.34 | 1.74 | 1.83 | 2.72 | 2.68 | 2.96 | 2.57 | 0.91 | 3.02 | 1.27 | 0.30 |
| TRINITY_DN65386_c3_g2 | 20.12 | 17.96 | 15.74 | 20.21 | 16.97 | 16.13 | 14.39 | 11.22 | 15.51 | 7.93 | 8.03 |
| TRINITY_DN65399_c2_g2 | 24.90 | 18.19 | 15.96 | 17.11 | 14.90 | 13.34 | 10.91 | 11.52 | 16.72 | 9.34 | 33.81 |
| TRINITY_DN65424_c1_g1 | 12.01 | 9.34 | 8.76 | 11.76 | 13.90 | 11.36 | 10.63 | 13.78 | 13.40 | 5.61 | 5.09 |
| TRINITY_DN65471_c0_g1 | 11.08 | 9.49 | 8.85 | 11.09 | 8.82 | 9.24 | 7.62 | 6.50 | 7.68 | 5.68 | 2.80 |
| TRINITY_DN65496_c3_g1 | 23.54 | 17.64 | 15.77 | 10.75 | 18.44 | 17.38 | 9.57 | 10.23 | 19.06 | 8.39 | 11.30 |
| TRINITY_DN65554_c1_g6 | 10.15 | 5.74 | 5.61 | 12.12 | 2.36 | 2.63 | 0.02 | 0.00 | 0.01 | 0.00 | 0.00 |
| TRINITY_DN65610_c0_g3 | 12.21 | 9.99 | 4.65 | 5.25 | 3.22 | 1.80 | 1.41 | 1.33 | 0.84 | 0.54 | 0.10 |
| TRINITY_DN65610_c2_g1 | 13.89 | 14.78 | 21.75 | 27.76 | 26.67 | 34.43 | 21.91 | 6.31 | 10.46 | 3.19 | 0.68 |
| TRINITY_DN65663_c5_g1 | 4.81 | 3.87 | 3.70 | 5.03 | 5.38 | 4.81 | 5.06 | 7.06 | 4.75 | 5.49 | 3.78 |
| TRINITY_DN65663_c6_g1 | 6.32 | 5.67 | 5.28 | 6.62 | 5.51 | 6.29 | 7.00 | 5.64 | 6.57 | 6.89 | 1.64 |
| TRINITY_DN65693_c2_g1 | 45.19 | 48.09 | 65.48 | 74.39 | 52.68 | 43.95 | 18.49 | 8.03 | 8.80 | 7.91 | 0.39 |
| TRINITY_DN65732_c1_g5 | 3.58 | 3.85 | 3.46 | 4.34 | 4.69 | 4.68 | 4.11 | 4.46 | 3.49 | 2.68 | 1.67 |
| TRINITY_DN65808_c4_g1 | 29.83 | 30.29 | 26.13 | 31.80 | 24.08 | 27.87 | 13.67 | 11.64 | 11.99 | 8.00 | 3.59 |
| TRINITY_DN65829_c1_g2 | 14.75 | 15.72 | 15.40 | 15.40 | 8.93 | 10.50 | 1.01 | 0.34 | 0.50 | 0.44 | 0.13 |
| TRINITY_DN65829_c1_g3 | 6.21 | 3.30 | 1.69 | 2.54 | 2.24 | 2.07 | 0.95 | 0.93 | 1.15 | 0.78 | 1.41 |
| TRINITY_DN65846_c0_g1 | 57.56 | 45.64 | 54.18 | 44.77 | 49.74 | 39.69 | 23.00 | 20.33 | 32.36 | 21.48 | 38.52 |
| TRINITY_DN65897_c0_g1 | 8.29 | 8.23 | 3.53 | 2.06 | 0.79 | 1.22 | 1.18 | 3.65 | 3.32 | 2.56 | 34.75 |
| TRINITY_DN66148_c0_g1 | 23.65 | 22.39 | 25.04 | 41.45 | 54.40 | 40.71 | 19.86 | 29.91 | 31.40 | 11.90 | 30.94 |
| TRINITY_DN66186_c1_g1 | 10.50 | 11.16 | 13.40 | 21.15 | 20.80 | 23.25 | 17.95 | 23.62 | 18.54 | 20.50 | 10.40 |
| TRINITY_DN66228_c0_g1 | 2.14 | 1.83 | 1.40 | 1.16 | 2.66 | 1.84 | 1.30 | 2.24 | 1.80 | 2.45 | 1.87 |
| TRINITY_DN66228_c0_g3 | 4.16 | 4.55 | 3.03 | 2.49 | 5.01 | 3.46 | 2.96 | 3.16 | 4.16 | 5.60 | 2.48 |
| TRINITY_DN66374_c0_g1 | 19.13 | 16.53 | 14.27 | 17.70 | 18.90 | 18.56 | 13.11 | 8.94 | 12.47 | 8.96 | 3.03 |
| TRINITY_DN66414_c0_g5 | 12.21 | 15.28 | 11.06 | 11.09 | 10.77 | 9.76 | 16.53 | 18.53 | 11.66 | 12.58 | 38.30 |
| TRINITY_DN66474_c0_g2 | 16.31 | 14.02 | 12.66 | 12.61 | 23.11 | 15.72 | 11.14 | 15.66 | 12.29 | 9.43 | 2.14 |
| TRINITY_DN66606_c1_g1 | 12.59 | 10.95 | 8.72 | 8.92 | 20.24 | 22.37 | 19.54 | 13.53 | 9.59 | 4.11 | 3.20 |
| TRINITY_DN66670_c0_g1 | 17.49 | 18.38 | 18.45 | 25.56 | 20.79 | 20.67 | 25.37 | 25.91 | 20.10 | 22.24 | 7.86 |
| TRINITY_DN66770_c5_g1 | 4.45 | 3.57 | 3.61 | 2.94 | 3.34 | 3.07 | 1.90 | 1.70 | 1.59 | 2.32 | 0.57 |
| TRINITY_DN66770_c5_g3 | 6.32 | 7.04 | 5.48 | 5.08 | 9.38 | 5.53 | 3.75 | 4.68 | 6.75 | 2.83 | 1.03 |
| TRINITY_DN66892_c1_g4 | 53.97 | 44.01 | 42.24 | 45.61 | 39.09 | 37.77 | 31.45 | 35.52 | 27.59 | 20.37 | 13.54 |
| TRINITY_DN66940_c0_g1 | 14.59 | 15.75 | 13.38 | 13.34 | 16.17 | 16.70 | 15.80 | 21.26 | 20.30 | 12.38 | 9.33 |
| TRINITY_DN67151_c5_g1 | 124.21 | 54.02 | 43.95 | 45.25 | 64.72 | 34.14 | 10.40 | 20.36 | 34.04 | 20.11 | 298.50 |
| TRINITY_DN67197_c0_g1 | 5.83 | 17.22 | 52.74 | 115.35 | 147.52 | 159.15 | 47.28 | 25.07 | 28.10 | 1.55 | 0.09 |
| TRINITY_DN67221_c0_g11 | 8.18 | 7.69 | 6.29 | 9.39 | 7.75 | 10.18 | 10.96 | 4.51 | 11.28 | 8.26 | 1.66 |
| TRINITY_DN67221_c0_g2 | 32.41 | 28.09 | 28.13 | 30.47 | 26.91 | 28.16 | 23.51 | 22.58 | 22.79 | 18.63 | 10.20 |
| TRINITY_DN67221_c0_g3 | 6.35 | 5.29 | 4.13 | 4.30 | 5.87 | 5.92 | 4.30 | 2.48 | 4.24 | 2.65 | 0.74 |
| TRINITY_DN67221_c0_g6 | 18.00 | 16.54 | 15.38 | 19.98 | 20.37 | 21.97 | 18.16 | 14.11 | 18.02 | 12.65 | 9.04 |
| TRINITY_DN67221_c0_g7 | 3.06 | 2.92 | 2.51 | 3.98 | 3.18 | 4.13 | 4.78 | 3.31 | 5.02 | 2.85 | 0.96 |
| TRINITY_DN67245_c0_g2 | 0.24 | 0.10 | 0.05 | 1.30 | 10.94 | 9.00 | 13.27 | 27.20 | 24.76 | 39.53 | 4.59 |
| TRINITY_DN67273_c1_g1 | 160.23 | 110.77 | 78.90 | 61.17 | 27.75 | 24.94 | 16.14 | 13.52 | 15.45 | 23.77 | 59.06 |
| TRINITY_DN67436_c3_g4 | 16.95 | 13.62 | 12.13 | 14.24 | 10.57 | 12.42 | 8.13 | 6.39 | 6.13 | 2.95 | 2.43 |
| TRINITY_DN67657_c5_g4 | 16.67 | 11.78 | 2.91 | 3.62 | 1.99 | 1.11 | 1.17 | 2.31 | 1.26 | 1.06 | 2.02 |
| TRINITY_DN67665_c3_g3 | 2.80 | 2.36 | 1.95 | 2.91 | 2.93 | 2.60 | 2.42 | 3.07 | 3.30 | 2.67 | 1.37 |
| TRINITY_DN67755_c0_g1 | 46.58 | 34.55 | 11.32 | 9.52 | 2.12 | 1.47 | 0.55 | 0.29 | 0.73 | 0.09 | 0.00 |
| TRINITY_DN67837_c0_g1 | 58.76 | 45.91 | 48.68 | 21.45 | 18.33 | 36.61 | 13.11 | 0.83 | 3.77 | 12.40 | 0.07 |
| TRINITY_DN67846_c3_g1 | 21.50 | 18.18 | 15.97 | 18.38 | 16.79 | 17.61 | 16.16 | 14.17 | 24.51 | 43.77 | 20.08 |
| TRINITY_DN67854_c0_g2 | 13.63 | 10.15 | 3.09 | 5.17 | 4.32 | 2.91 | 2.83 | 2.01 | 1.77 | 0.56 | 1.28 |
| TRINITY_DN67960_c0_g1 | 33.97 | 27.27 | 22.47 | 25.92 | 23.36 | 19.07 | 12.67 | 15.50 | 10.73 | 8.46 | 0.92 |
| TRINITY_DN67978_c1_g3 | 30.92 | 27.31 | 32.50 | 26.66 | 14.88 | 14.55 | 19.46 | 11.92 | 6.53 | 3.10 | 0.47 |
| TRINITY_DN68105_c0_g2 | 11.28 | 10.76 | 10.33 | 12.13 | 12.79 | 13.86 | 9.39 | 6.05 | 13.66 | 16.15 | 14.24 |
| TRINITY_DN68196_c1_g1 | 5.68 | 5.39 | 5.03 | 7.01 | 8.94 | 7.76 | 8.82 | 9.14 | 9.76 | 8.37 | 5.71 |
| TRINITY_DN68432_c4_g1 | 7.11 | 6.62 | 6.33 | 7.30 | 6.60 | 6.82 | 5.09 | 5.75 | 4.24 | 2.53 | 7.08 |
| TRINITY_DN68445_c1_g4 | 26.44 | 21.40 | 15.66 | 13.53 | 12.17 | 13.96 | 12.73 | 18.84 | 18.30 | 46.68 | 74.56 |
| TRINITY_DN68500_c0_g1 | 200.23 | 146.77 | 133.04 | 148.41 | 128.94 | 88.71 | 61.52 | 75.00 | 57.55 | 29.52 | 8.71 |
| TRINITY_DN68500_c0_g2 | 85.10 | 61.71 | 47.54 | 46.18 | 85.09 | 54.22 | 20.34 | 15.42 | 23.40 | 8.54 | 2.21 |
| TRINITY_DN68501_c3_g3 | 10.69 | 8.22 | 3.97 | 8.80 | 12.54 | 13.05 | 11.29 | 6.70 | 31.63 | 25.09 | 0.11 |
| TRINITY_DN68520_c2_g4 | 7.90 | 8.28 | 6.43 | 9.38 | 7.08 | 7.38 | 8.20 | 7.73 | 9.33 | 10.82 | 2.03 |
| TRINITY_DN68598_c0_g2 | 3.66 | 3.61 | 3.20 | 4.16 | 2.89 | 3.69 | 2.64 | 1.32 | 2.70 | 3.30 | 0.41 |
| TRINITY_DN68762_c0_g1 | 5.28 | 5.85 | 4.14 | 7.07 | 10.46 | 9.84 | 4.14 | 0.31 | 57.81 | 52.55 | 11.35 |
| TRINITY_DN68840_c0_g2 | 30.68 | 27.62 | 23.66 | 27.50 | 17.78 | 18.12 | 20.61 | 18.28 | 18.22 | 14.29 | 1.23 |
| TRINITY_DN68856_c1_g1 | 12.26 | 15.12 | 10.99 | 9.37 | 5.70 | 5.07 | 4.82 | 6.66 | 4.21 | 19.77 | 149.07 |
| TRINITY_DN68856_c1_g3 | 3.40 | 2.45 | 2.78 | 2.57 | 2.09 | 1.06 | 1.80 | 6.54 | 12.13 | 15.03 | 0.00 |
| TRINITY_DN68857_c0_g1 | 95.50 | 99.98 | 89.98 | 71.96 | 60.76 | 80.31 | 58.66 | 44.55 | 40.00 | 24.04 | 22.01 |
| TRINITY_DN69020_c3_g1 | 39.25 | 31.85 | 26.09 | 23.14 | 26.76 | 22.73 | 20.45 | 26.16 | 17.76 | 16.64 | 39.62 |
| TRINITY_DN69064_c0_g2 | 101.13 | 61.76 | 24.59 | 20.14 | 8.75 | 11.81 | 3.44 | 1.11 | 0.55 | 0.27 | 0.00 |
| TRINITY_DN69151_c0_g1 | 5.53 | 3.44 | 1.95 | 8.03 | 4.18 | 2.31 | 2.19 | 15.74 | 4.05 | 5.08 | 0.00 |
| TRINITY_DN69295_c0_g3 | 113.94 | 95.03 | 81.18 | 70.33 | 45.66 | 50.66 | 17.86 | 11.33 | 15.20 | 8.15 | 8.06 |
| TRINITY_DN69514_c2_g1 | 48.37 | 46.32 | 39.57 | 46.27 | 38.83 | 39.30 | 24.24 | 23.19 | 23.04 | 18.03 | 18.37 |
| TRINITY_DN69538_c0_g5 | 1.90 | 14.91 | 2.84 | 1.05 | 0.02 | 0.00 | 0.07 | 0.05 | 11.85 | 413.99 | 44.42 |
| TRINITY_DN69563_c0_g2 | 9.95 | 7.32 | 5.96 | 6.30 | 5.70 | 3.90 | 4.03 | 4.52 | 3.74 | 2.98 | 4.69 |
| TRINITY_DN69602_c1_g2 | 6.30 | 6.60 | 6.06 | 4.98 | 4.58 | 3.30 | 4.03 | 3.13 | 2.16 | 1.86 | 13.25 |
| TRINITY_DN69637_c4_g1 | 12.14 | 9.56 | 12.63 | 16.69 | 20.32 | 15.28 | 17.09 | 26.07 | 12.65 | 10.99 | 2.17 |
| TRINITY_DN69670_c0_g1 | 24.39 | 20.35 | 19.42 | 18.25 | 14.60 | 11.92 | 3.07 | 4.39 | 5.56 | 5.13 | 4.48 |
| TRINITY_DN69674_c3_g1 | 1.81 | 5.89 | 3.27 | 1.55 | 2.08 | 2.76 | 3.26 | 2.03 | 2.75 | 0.26 | 0.85 |
| TRINITY_DN69913_c1_g6 | 22.45 | 23.98 | 24.14 | 30.08 | 26.36 | 29.00 | 28.11 | 40.25 | 29.88 | 43.23 | 29.31 |
| TRINITY_DN69963_c0_g1 | 111.63 | 86.81 | 86.54 | 101.97 | 96.03 | 76.62 | 81.27 | 94.06 | 120.19 | 103.09 | 21.39 |
| TRINITY_DN70039_c0_g2 | 14.82 | 13.40 | 12.71 | 13.66 | 13.32 | 15.40 | 11.89 | 10.12 | 11.91 | 10.07 | 3.72 |
| TRINITY_DN70062_c1_g3 | 39.11 | 27.34 | 20.86 | 15.88 | 16.94 | 19.16 | 15.36 | 12.53 | 19.60 | 14.75 | 27.40 |
| TRINITY_DN70069_c1_g1 | 21.64 | 18.77 | 15.86 | 20.34 | 16.19 | 19.74 | 23.77 | 21.61 | 29.21 | 45.45 | 9.35 |
| TRINITY_DN70156_c2_g3 | 11.89 | 8.55 | 8.97 | 12.71 | 10.33 | 11.47 | 10.11 | 11.12 | 8.49 | 10.92 | 4.94 |
| TRINITY_DN70245_c0_g2 | 0.87 | 4.36 | 3.58 | 3.92 | 4.46 | 3.58 | 4.39 | 9.14 | 19.64 | 52.51 | 46.61 |
| TRINITY_DN70269_c1_g1 | 1.16 | 1.76 | 1.49 | 3.96 | 2.82 | 2.38 | 2.93 | 5.72 | 12.77 | 15.83 | 0.09 |
| TRINITY_DN70320_c5_g6 | 14.65 | 12.84 | 13.48 | 16.50 | 12.10 | 13.93 | 12.80 | 10.24 | 14.29 | 9.48 | 5.19 |
| TRINITY_DN70339_c1_g1 | 45.54 | 68.70 | 92.15 | 123.04 | 228.47 | 255.08 | 252.74 | 274.91 | 578.34 | 738.77 | 24.90 |
| TRINITY_DN70461_c2_g4 | 8.63 | 7.18 | 8.39 | 19.04 | 98.22 | 68.71 | 38.92 | 102.19 | 81.00 | 74.79 | 8.20 |
| TRINITY_DN70513_c0_g1 | 10.62 | 7.15 | 2.64 | 1.65 | 1.04 | 0.64 | 0.28 | 0.57 | 0.69 | 1.03 | 28.80 |
| TRINITY_DN70513_c1_g1 | 8.87 | 8.50 | 4.75 | 4.70 | 2.25 | 1.89 | 0.65 | 0.35 | 0.89 | 0.65 | 0.04 |
| TRINITY_DN70669_c7_g1 | 1.94 | 1.94 | 1.38 | 2.53 | 3.50 | 2.19 | 2.61 | 11.01 | 5.54 | 7.14 | 5.41 |
| TRINITY_DN70684_c8_g1 | 2.43 | 1.58 | 2.95 | 2.09 | 1.59 | 1.86 | 2.30 | 3.68 | 1.87 | 1.03 | 10.56 |
| TRINITY_DN70717_c1_g2 | 8.18 | 13.46 | 9.64 | 14.09 | 12.88 | 17.72 | 13.36 | 5.25 | 7.79 | 31.72 | 12.12 |
| TRINITY_DN70858_c0_g5 | 2.76 | 4.17 | 4.97 | 5.89 | 6.31 | 6.70 | 4.79 | 3.62 | 5.70 | 3.25 | 1.94 |
| TRINITY_DN71095_c3_g10 | 15.06 | 12.39 | 11.25 | 13.32 | 9.09 | 10.38 | 9.82 | 9.02 | 7.73 | 6.07 | 4.50 |
| TRINITY_DN71156_c3_g2 | 41.77 | 35.95 | 27.64 | 19.68 | 15.98 | 13.75 | 4.09 | 5.23 | 7.77 | 8.92 | 71.76 |
| TRINITY_DN71216_c4_g3 | 6.88 | 6.30 | 6.50 | 7.34 | 6.48 | 7.28 | 5.30 | 4.47 | 6.56 | 5.13 | 2.74 |
| TRINITY_DN71279_c0_g1 | 9.81 | 10.32 | 8.72 | 10.73 | 11.59 | 10.81 | 11.62 | 9.14 | 10.71 | 9.27 | 1.92 |
| TRINITY_DN71569_c1_g1 | 25.02 | 25.33 | 19.25 | 15.41 | 15.39 | 12.05 | 13.48 | 11.57 | 25.73 | 28.44 | 6.83 |
| TRINITY_DN71703_c0_g4 | 16.21 | 14.25 | 13.28 | 17.72 | 12.65 | 16.01 | 21.30 | 8.30 | 19.56 | 12.25 | 7.48 |
| TRINITY_DN71750_c3_g4 | 5.44 | 4.81 | 4.55 | 5.48 | 5.48 | 5.37 | 5.20 | 4.48 | 7.80 | 9.21 | 7.26 |
| TRINITY_DN71755_c1_g1 | 22.37 | 21.07 | 14.30 | 12.54 | 8.55 | 5.53 | 3.49 | 4.90 | 4.75 | 10.81 | 273.14 |
| TRINITY_DN71919_c0_g6 | 12.31 | 11.47 | 9.27 | 10.01 | 11.13 | 8.50 | 7.08 | 10.33 | 7.91 | 7.63 | 5.04 |
| TRINITY_DN71928_c0_g1 | 7.53 | 6.02 | 5.72 | 6.92 | 4.98 | 4.36 | 3.23 | 2.13 | 2.28 | 0.72 | 0.62 |
| TRINITY_DN71942_c0_g1 | 39.92 | 51.48 | 19.12 | 19.78 | 9.42 | 13.29 | 5.05 | 3.92 | 8.68 | 58.48 | 82.69 |
| TRINITY_DN72224_c2_g4 | 2.03 | 1.77 | 1.98 | 1.62 | 2.41 | 1.59 | 1.17 | 1.64 | 2.82 | 2.89 | 2.37 |
| TRINITY_DN72285_c1_g2 | 27.73 | 27.77 | 9.91 | 12.47 | 7.57 | 4.51 | 11.98 | 8.16 | 4.10 | 1.85 | 0.06 |
| TRINITY_DN72285_c1_g3 | 30.73 | 26.21 | 26.46 | 33.91 | 26.49 | 25.81 | 21.07 | 16.14 | 15.46 | 8.24 | 1.32 |
| TRINITY_DN72299_c3_g3 | 5.92 | 4.98 | 4.72 | 6.08 | 6.10 | 5.97 | 4.77 | 4.65 | 4.45 | 3.39 | 2.04 |
| TRINITY_DN72304_c1_g3 | 4.25 | 4.39 | 3.31 | 4.69 | 5.05 | 4.45 | 4.71 | 4.33 | 4.39 | 3.40 | 1.59 |
| TRINITY_DN72315_c1_g2 | 126.25 | 92.17 | 207.39 | 96.85 | 85.45 | 86.19 | 37.44 | 31.21 | 15.16 | 8.75 | 10.89 |
| TRINITY_DN72316_c5_g5 | 6.45 | 6.49 | 6.12 | 7.09 | 6.63 | 5.38 | 4.10 | 4.86 | 6.81 | 6.80 | 4.53 |
| TRINITY_DN72407_c0_g1 | 7.67 | 7.93 | 2.20 | 3.35 | 2.54 | 1.97 | 3.70 | 1.02 | 8.32 | 3.29 | 154.59 |
| TRINITY_DN72442_c0_g1 | 13.03 | 13.89 | 17.75 | 22.78 | 34.29 | 44.77 | 63.09 | 30.06 | 27.80 | 6.69 | 21.12 |
| TRINITY_DN72442_c0_g2 | 17.70 | 27.04 | 10.48 | 10.55 | 4.42 | 4.53 | 4.40 | 2.47 | 6.28 | 4.11 | 61.24 |
| TRINITY_DN72443_c1_g2 | 12.08 | 8.96 | 9.37 | 9.06 | 9.72 | 8.34 | 7.35 | 8.25 | 4.18 | 2.70 | 1.89 |
| TRINITY_DN72450_c1_g1 | 3.33 | 2.53 | 2.03 | 3.43 | 5.25 | 4.42 | 7.40 | 4.28 | 10.75 | 2.87 | 0.69 |
| TRINITY_DN72651_c2_g1 | 23.10 | 20.51 | 21.60 | 19.63 | 18.69 | 15.83 | 12.12 | 15.14 | 13.74 | 10.16 | 6.82 |
| TRINITY_DN7275_c0_g1 | 12.37 | 9.65 | 2.63 | 4.88 | 6.57 | 9.78 | 5.33 | 4.36 | 3.90 | 0.25 | 0.23 |
| TRINITY_DN72801_c1_g1 | 7.08 | 5.35 | 2.08 | 1.99 | 2.26 | 1.84 | 0.86 | 1.13 | 2.85 | 2.19 | 3.32 |
| TRINITY_DN72802_c2_g1 | 27.42 | 21.04 | 15.11 | 17.95 | 17.34 | 13.61 | 10.74 | 7.89 | 6.04 | 4.86 | 0.12 |
| TRINITY_DN72802_c2_g2 | 20.54 | 22.29 | 12.67 | 18.63 | 13.96 | 15.50 | 11.77 | 7.50 | 18.46 | 11.11 | 0.88 |
| TRINITY_DN72802_c3_g1 | 25.19 | 19.70 | 21.77 | 27.73 | 31.44 | 27.38 | 19.25 | 19.87 | 25.73 | 20.59 | 8.48 |
| TRINITY_DN73070_c0_g1 | 3.89 | 3.83 | 2.12 | 1.48 | 2.46 | 2.26 | 1.39 | 1.74 | 3.50 | 5.07 | 3.71 |
| TRINITY_DN73119_c0_g2 | 10.16 | 7.19 | 4.16 | 5.14 | 6.47 | 5.68 | 4.68 | 2.85 | 4.02 | 1.98 | 0.00 |
| TRINITY_DN73139_c0_g4 | 31.75 | 40.51 | 25.41 | 21.43 | 14.96 | 13.93 | 21.37 | 8.23 | 12.19 | 2.01 | 0.64 |
| TRINITY_DN73172_c0_g3 | 49.73 | 58.30 | 50.98 | 70.52 | 98.82 | 76.61 | 129.93 | 158.94 | 172.28 | 136.54 | 165.33 |
| TRINITY_DN73211_c2_g1 | 26.00 | 18.44 | 12.29 | 13.46 | 23.31 | 16.49 | 9.52 | 10.25 | 15.25 | 9.07 | 2.53 |
| TRINITY_DN73211_c2_g2 | 25.13 | 19.44 | 10.51 | 10.62 | 14.87 | 10.58 | 3.93 | 7.86 | 4.85 | 2.66 | 2.24 |
| TRINITY_DN73314_c5_g7 | 49.28 | 49.55 | 49.92 | 57.12 | 100.06 | 55.29 | 110.72 | 277.98 | 128.24 | 60.56 | 2.29 |
| TRINITY_DN73366_c2_g1 | 7.73 | 9.99 | 34.42 | 113.23 | 273.88 | 225.18 | 197.73 | 806.05 | 358.96 | 224.66 | 53.65 |
| TRINITY_DN73480_c0_g1 | 35.77 | 37.78 | 33.88 | 27.92 | 36.11 | 39.81 | 30.23 | 22.24 | 19.38 | 25.34 | 5.48 |
| TRINITY_DN73496_c0_g3 | 6.12 | 7.27 | 4.50 | 6.72 | 4.86 | 6.24 | 3.72 | 2.45 | 4.45 | 4.11 | 0.47 |
| TRINITY_DN73574_c3_g1 | 49.64 | 53.42 | 108.60 | 150.42 | 151.48 | 161.53 | 124.70 | 99.43 | 142.91 | 95.69 | 4.94 |
| TRINITY_DN73580_c1_g1 | 2.21 | 8.52 | 3.36 | 4.41 | 4.10 | 12.29 | 20.66 | 12.15 | 31.56 | 39.23 | 2.01 |
| TRINITY_DN73679_c4_g1 | 16.02 | 13.61 | 14.07 | 14.85 | 18.48 | 16.34 | 13.57 | 26.26 | 68.58 | 139.86 | 9.13 |
| TRINITY_DN73752_c1_g1 | 84.30 | 73.89 | 36.10 | 39.96 | 33.72 | 31.98 | 22.95 | 13.30 | 49.03 | 146.07 | 107.72 |
| TRINITY_DN73900_c0_g2 | 7.06 | 7.14 | 5.28 | 8.29 | 6.79 | 5.97 | 6.32 | 4.68 | 5.78 | 5.16 | 0.95 |
| TRINITY_DN73969_c3_g1 | 7.02 | 7.99 | 6.55 | 6.73 | 9.72 | 7.65 | 10.99 | 13.05 | 12.04 | 11.28 | 9.08 |
| TRINITY_DN73986_c2_g1 | 229.66 | 195.21 | 170.47 | 180.10 | 218.69 | 212.38 | 144.38 | 115.38 | 131.87 | 105.01 | 48.20 |
| TRINITY_DN74058_c0_g2 | 131.47 | 115.75 | 108.84 | 182.72 | 162.62 | 174.25 | 186.69 | 112.81 | 178.75 | 70.92 | 7.72 |
| TRINITY_DN74067_c0_g1 | 33.82 | 25.41 | 30.66 | 25.50 | 29.50 | 20.11 | 21.36 | 36.16 | 35.70 | 36.50 | 9.71 |
| TRINITY_DN74104_c3_g1 | 7.92 | 6.43 | 5.80 | 5.63 | 6.80 | 5.77 | 5.47 | 6.48 | 13.51 | 20.07 | 5.61 |
| TRINITY_DN74189_c0_g6 | 40.84 | 31.33 | 22.50 | 13.54 | 5.63 | 4.82 | 4.02 | 5.61 | 3.86 | 4.33 | 0.97 |
| TRINITY_DN74193_c2_g3 | 8.34 | 7.67 | 5.40 | 6.55 | 4.37 | 3.64 | 5.77 | 5.52 | 6.69 | 8.51 | 8.09 |
| TRINITY_DN74205_c0_g1 | 29.97 | 22.85 | 23.92 | 28.89 | 20.94 | 18.36 | 17.19 | 17.24 | 21.79 | 16.43 | 9.60 |
| TRINITY_DN74508_c0_g4 | 5.77 | 5.26 | 2.37 | 5.22 | 3.02 | 3.89 | 5.08 | 2.64 | 4.67 | 3.71 | 0.57 |
| TRINITY_DN74646_c4_g3 | 27.48 | 24.52 | 23.28 | 17.51 | 14.99 | 12.17 | 16.95 | 25.27 | 17.01 | 22.24 | 7.30 |
| TRINITY_DN74764_c0_g1 | 13.59 | 10.82 | 13.37 | 16.41 | 16.79 | 12.66 | 9.21 | 11.16 | 8.81 | 5.04 | 2.41 |
| TRINITY_DN74819_c1_g1 | 1.79 | 2.09 | 2.53 | 2.76 | 2.29 | 2.28 | 1.40 | 1.43 | 2.22 | 2.01 | 2.99 |
| TRINITY_DN74819_c1_g4 | 5.68 | 4.11 | 5.02 | 4.30 | 7.29 | 4.31 | 2.29 | 3.75 | 3.17 | 1.80 | 8.34 |
| TRINITY_DN74973_c3_g1 | 58.76 | 56.75 | 66.38 | 63.32 | 60.93 | 77.48 | 68.16 | 33.01 | 25.09 | 6.24 | 0.34 |
| TRINITY_DN75176_c1_g1 | 30.98 | 27.13 | 24.69 | 18.41 | 33.53 | 22.65 | 9.89 | 9.74 | 11.88 | 8.87 | 4.97 |
| TRINITY_DN75200_c3_g1 | 6.59 | 8.56 | 7.03 | 8.06 | 7.01 | 5.76 | 4.39 | 4.38 | 3.45 | 3.87 | 0.76 |
| TRINITY_DN75304_c4_g1 | 17.33 | 15.24 | 13.63 | 13.65 | 28.00 | 17.42 | 9.60 | 14.16 | 14.40 | 9.23 | 22.22 |
| TRINITY_DN75423_c3_g1 | 4.16 | 6.70 | 3.79 | 5.08 | 3.70 | 5.30 | 4.37 | 6.66 | 4.50 | 14.48 | 1.15 |
| TRINITY_DN75474_c2_g1 | 49.50 | 62.82 | 35.78 | 52.02 | 27.04 | 19.05 | 35.88 | 30.63 | 39.93 | 48.50 | 57.42 |
| TRINITY_DN75484_c1_g1 | 6.16 | 11.40 | 8.61 | 5.31 | 5.79 | 6.97 | 4.58 | 4.96 | 8.22 | 7.71 | 18.40 |
| TRINITY_DN75513_c1_g3 | 28.14 | 26.86 | 24.00 | 29.16 | 22.46 | 32.29 | 22.30 | 20.48 | 31.14 | 27.83 | 5.88 |
| TRINITY_DN75569_c1_g2 | 16.65 | 15.37 | 11.48 | 16.14 | 21.85 | 20.78 | 20.57 | 14.57 | 18.72 | 10.38 | 4.07 |
| TRINITY_DN75574_c1_g1 | 26.07 | 21.53 | 18.43 | 20.53 | 29.08 | 23.82 | 14.51 | 9.96 | 15.08 | 9.25 | 8.33 |
| TRINITY_DN75786_c1_g1 | 4.25 | 3.23 | 8.70 | 2.14 | 3.63 | 2.45 | 2.20 | 0.87 | 3.35 | 0.79 | 0.31 |
| TRINITY_DN75825_c1_g2 | 8.14 | 8.69 | 7.50 | 8.85 | 12.06 | 9.45 | 4.64 | 5.85 | 5.63 | 3.69 | 3.97 |
| TRINITY_DN75832_c2_g3 | 6.31 | 6.16 | 8.03 | 5.64 | 7.81 | 7.37 | 6.95 | 7.21 | 8.20 | 6.69 | 4.72 |
| TRINITY_DN75833_c12_g1 | 35.63 | 260.97 | 813.67 | 978.26 | 1466.91 | 1376.66 | 1416.71 | 2182.49 | 1370.39 | 937.13 | 159.25 |
| TRINITY_DN75902_c3_g2 | 8.57 | 6.61 | 6.63 | 5.30 | 14.11 | 9.88 | 7.10 | 10.03 | 8.64 | 5.62 | 9.14 |
| TRINITY_DN76002_c5_g3 | 6.99 | 3.40 | 3.05 | 27.76 | 277.82 | 282.64 | 435.09 | 971.73 | 676.18 | 636.37 | 284.79 |
| TRINITY_DN76223_c4_g2 | 8.22 | 8.27 | 13.28 | 13.67 | 9.67 | 6.44 | 10.49 | 21.93 | 34.04 | 101.13 | 7.75 |
| TRINITY_DN76242_c0_g1 | 21.17 | 24.09 | 17.36 | 36.56 | 22.89 | 29.60 | 35.75 | 19.40 | 38.48 | 29.91 | 6.22 |
| TRINITY_DN76366_c3_g2 | 3.64 | 2.85 | 3.08 | 4.56 | 3.98 | 5.19 | 5.13 | 1.75 | 4.75 | 3.01 | 0.15 |
| TRINITY_DN76513_c2_g1 | 108.24 | 60.34 | 49.41 | 129.90 | 287.45 | 172.46 | 189.68 | 873.35 | 369.64 | 325.74 | 34.18 |
| TRINITY_DN76660_c8_g1 | 12.18 | 12.72 | 10.74 | 9.70 | 7.22 | 6.33 | 7.20 | 8.19 | 4.04 | 5.18 | 0.44 |
| TRINITY_DN91387_c0_g1 | 11.64 | 16.10 | 8.46 | 10.91 | 6.20 | 10.31 | 4.15 | 6.95 | 5.67 | 2.92 | 0.50 |
| TRINITY_DN91432_c0_g1 | 7.12 | 4.93 | 3.80 | 4.20 | 1.78 | 1.62 | 2.02 | 2.76 | 2.76 | 11.88 | 0.12 |
| TRINITY_DN91546_c0_g1 | 10.54 | 11.19 | 7.33 | 7.05 | 5.63 | 7.36 | 1.18 | 4.28 | 0.77 | 2.59 | 0.20 |

**Supplementary Table 7.** Annotation of unigenes riched in ‘Plant hormone signal transduction’ pathway

| **gene id** | **NR description** |
| --- | --- |
| TRINITY_DN58400_c1_g2 | PREDICTED: jasmonic acid-amido synthetase JAR1-like isoform X1 [Phoenix dactylifera] |
| TRINITY_DN75921_c2_g3 | PREDICTED: LOW QUALITY PROTEIN: BRI1 kinase inhibitor 1-like [Phoenix dactylifera] |
| TRINITY_DN40667_c0_g1 | PREDICTED: auxin-responsive protein SAUR32-like [Musa acuminata subsp. malaccensis] |
| TRINITY_DN38238_c0_g1 | PREDICTED: auxin-induced protein 6B-like [Elaeis guineensis] |
| TRINITY_DN71936_c1_g2 | PREDICTED: probable indole-3-acetic acid-amido synthetase GH3.8 isoform X2 [Musa acuminata subsp. malaccensis] |
| TRINITY_DN53475_c0_g2 | PREDICTED: auxin response factor 7-like [Phoenix dactylifera] |
| TRINITY_DN49188_c7_g2 | PREDICTED: transcription factor TGA4 isoform X1 [Elaeis guineensis] |
| TRINITY_DN71936_c1_g1 | PREDICTED: probable indole-3-acetic acid-amido synthetase GH3.8 [Elaeis guineensis] |
| TRINITY_DN45020_c0_g1 | scarecrow-like protein 3 [Populus trichocarpa] |
| TRINITY_DN55566_c4_g1 | PREDICTED: coronatine-insensitive protein homolog 1a-like [Elaeis guineensis] |
| TRINITY_DN58109_c0_g1 | probable xyloglucan endotransglucosylase/hydrolase protein 23 [Medicago truncatula] |
| TRINITY_DN75314_c5_g2 | PREDICTED: transcription factor MYC2-like [Musa acuminata subsp. malaccensis] |
| TRINITY_DN46373_c1_g2 | PREDICTED: auxin-induced protein 15A [Phoenix dactylifera] |
| TRINITY_DN48344_c2_g1 | PREDICTED: transcription factor TGA2.2-like isoform X3 [Elaeis guineensis] |
| TRINITY_DN74078_c0_g1 | PREDICTED: systemin receptor SR160-like [Elaeis guineensis] |
| TRINITY_DN74078_c0_g3 | PREDICTED: systemin receptor SR160-like [Musa acuminata subsp. malaccensis] |
| TRINITY_DN74973_c3_g1 | DELLA protein GAI, partial [Lilium formosanum x Lilium longiflorum] |
| TRINITY_DN47309_c1_g3 | PREDICTED: two-component response regulator ORR22-like isoform X1 [Elaeis guineensis] |
| TRINITY_DN66148_c0_g1 | PREDICTED: auxin response factor 11 isoform X1 [Elaeis guineensis] |
| TRINITY_DN57802_c0_g1 | hypothetical protein GQ55_7G065700 [Panicum hallii var. hallii] |
| TRINITY_DN65601_c1_g1 | PREDICTED: two-component response regulator ORR6-like [Musa acuminata subsp. malaccensis] |
| TRINITY_DN28807_c0_g2 | PREDICTED: two-component response regulator ARR1-like isoform X1 [Phoenix dactylifera] |
| TRINITY_DN69256_c0_g2 | PREDICTED: gibberellin receptor GID1C-like [Elaeis guineensis] |
| TRINITY_DN42350_c0_g1 | PREDICTED: protein TIFY 6b-like isoform X3 [Phoenix dactylifera] |
| TRINITY_DN56792_c1_g4 | PREDICTED: transcription factor PIF4-like isoform X2 [Elaeis guineensis] |
| TRINITY_DN67207_c2_g1 | probable tocopherol O-methyltransferase, chloroplastic [Asparagus officinalis] |
| TRINITY_DN53995_c0_g1 | probable protein phosphatase 2C 30 [Brachypodium distachyon] |
| TRINITY_DN63043_c2_g1 | PREDICTED: protein BREAST CANCER SUSCEPTIBILITY 1 homolog isoform X2 [Elaeis guineensis] |
| TRINITY_DN58634_c3_g4 | PREDICTED: auxin-induced protein 6B-like [Musa acuminata subsp. malaccensis] |
| TRINITY_DN44440_c0_g1 | PREDICTED: auxin-responsive protein SAUR36 [Elaeis guineensis] |
| TRINITY_DN52863_c1_g1 | PREDICTED: probable protein phosphatase 2C 37 [Musa acuminata subsp. malaccensis] |
| TRINITY_DN70055_c5_g1 | PYL1 [Zoysia japonica] |
| TRINITY_DN68285_c3_g1 | PREDICTED: regulatory protein NPR6-like isoform X1 [Phoenix dactylifera] |
| TRINITY_DN70919_c4_g1 | PREDICTED: auxin-responsive protein IAA17 [Elaeis guineensis] |
| TRINITY_DN39254_c0_g1 | PREDICTED: auxin-responsive protein SAUR32-like [Musa acuminata subsp. malaccensis] |
| TRINITY_DN44964_c0_g1 | PREDICTED: transcription factor PIF4-like isoform X2 [Phoenix dactylifera] |
| TRINITY_DN51102_c2_g1 | non-expressor of pathogenesis-related 1 [Lilium hybrid division VII] |
| TRINITY_DN41773_c0_g1 | PREDICTED: ethylene-responsive transcription factor 1B [Theobroma cacao] |
| TRINITY_DN13429_c0_g1 | PREDICTED: auxin-responsive protein SAUR71-like [Elaeis guineensis] |
| TRINITY_DN70319_c0_g1 | PREDICTED: probable indole-3-acetic acid-amido synthetase GH3.1 [Phoenix dactylifera] |
| TRINITY_DN54283_c0_g2 | hypothetical protein AQUCO_01400608v1 [Aquilegia coerulea] |
| TRINITY_DN70319_c0_g4 | PREDICTED: probable indole-3-acetic acid-amido synthetase GH3.8 [Elaeis guineensis] |
| TRINITY_DN43837_c0_g1 | PREDICTED: uncharacterized protein LOC103716962 [Phoenix dactylifera] |
| TRINITY_DN65081_c0_g1 | PREDICTED: auxin-responsive protein IAA10-like [Phoenix dactylifera] |
| TRINITY_DN56834_c9_g1 | PREDICTED: auxin-responsive protein SAUR32-like [Phoenix dactylifera] |
| TRINITY_DN71356_c1_g2 | PREDICTED: auxin-induced protein 15A-like [Nicotiana tabacum] |
| TRINITY_DN58411_c0_g2 | PREDICTED: probable xyloglucan endotransglucosylase/hydrolase protein 23 [Beta vulgaris subsp. vulgaris] |
| TRINITY_DN68500_c0_g1 | DELLA protein SLR1-like [Phalaenopsis equestris] |
| TRINITY_DN68500_c0_g2 | DELLA protein SLN1-like [Dendrobium catenatum] |
| TRINITY_DN44763_c0_g2 | PREDICTED: auxin-responsive protein IAA30 [Elaeis guineensis] |
| TRINITY_DN46172_c0_g1 | Auxin-responsive protein IAA8 [Ananas comosus] |
| TRINITY_DN23674_c0_g1 | auxin-responsive protein SAUR32 [Durio zibethinus] |
| TRINITY_DN58411_c0_g4 | probable xyloglucan endotransglucosylase/hydrolase protein 23 [Chenopodium quinoa] |
| TRINITY_DN67960_c0_g1 | PREDICTED: transcription factor PIF3 isoform X2 [Elaeis guineensis] |
| TRINITY_DN76534_c1_g1 | probable indole-3-acetic acid-amido synthetase GH3.1 [Olea europaea var. sylvestris] |
| TRINITY_DN45462_c0_g1 | PREDICTED: auxin-induced protein 22D-like [Phoenix dactylifera] |
| TRINITY_DN85365_c0_g1 | PREDICTED: probable protein phosphatase 2C 8 [Elaeis guineensis] |
| TRINITY_DN69518_c4_g5 | PREDICTED: abscisic acid receptor PYL12-like [Nelumbo nucifera] |
| TRINITY_DN64057_c1_g1 | transcription factor MYC2 [Lilium regale] |
| TRINITY_DN70343_c1_g1 | ethylene receptor [Lilium formosanum x Lilium longiflorum] |
| TRINITY_DN66040_c0_g2 | PREDICTED: two-component response regulator ORR6-like [Elaeis guineensis] |
| TRINITY_DN46418_c2_g4 | two-component response regulator ORR5-like [Aegilops tauschii subsp. tauschii] |
| TRINITY_DN62282_c2_g2 | transcription factor MYC2 [Lilium regale] |
| TRINITY_DN76534_c5_g4 | PREDICTED: probable indole-3-acetic acid-amido synthetase GH3.8 [Elaeis guineensis] |
| TRINITY_DN51993_c2_g1 | PREDICTED: auxin-responsive protein SAUR32-like [Elaeis guineensis] |
| TRINITY_DN43151_c0_g1 | PREDICTED: histidine-containing phosphotransfer protein 2-like [Musa acuminata subsp. malaccensis] |
| TRINITY_DN57853_c0_g6 | probable serine/threonine-protein kinase At4g35230 [Phalaenopsis equestris] |
| TRINITY_DN46416_c1_g1 | PREDICTED: auxin transporter-like protein 2 [Elaeis guineensis] |
| TRINITY_DN54499_c0_g1 | auxin transporter-like protein 2 [Morus notabilis] |
| TRINITY_DN43666_c0_g1 | PREDICTED: auxin-responsive protein SAUR71-like [Phoenix dactylifera] |
| TRINITY_DN47098_c5_g3 | PREDICTED: probable histidine kinase 5 [Phoenix dactylifera] |
| TRINITY_DN66922_c1_g7 | PREDICTED: protein TIFY 9-like [Musa acuminata subsp. malaccensis] |
| TRINITY_DN36748_c0_g1 | PREDICTED: indole-3-acetic acid-induced protein ARG7 [Phoenix dactylifera] |
| TRINITY_DN58931_c4_g1 | PREDICTED: protein TRANSPORT INHIBITOR RESPONSE 1-like [Phoenix dactylifera] |
| TRINITY_DN36987_c0_g1 | PREDICTED: pathogenesis-related protein 1 [Daucus carota subsp. sativus] |
| TRINITY_DN73628_c3_g2 | transcription factor MYC2 [Lilium regale] |
| TRINITY_DN56670_c1_g1 | PREDICTED: probable histidine kinase 3 isoform X1 [Elaeis guineensis] |
| TRINITY_DN65800_c3_g11 | abscisic acid receptor PYL8 [Prunus persica] |
| TRINITY_DN39172_c0_g1 | PREDICTED: transcription factor HBP-1b(c38)-like isoform X1 [Phoenix dactylifera] |
| TRINITY_DN59979_c1_g1 | PREDICTED: auxin-responsive protein SAUR32-like [Phoenix dactylifera] |
| TRINITY_DN59400_c5_g1 | PREDICTED: auxin-responsive protein IAA6-like isoform X2 [Phoenix dactylifera] |
| TRINITY_DN67812_c4_g9 | putative transcription factor bHLH family [Rosa chinensis] |
| TRINITY_DN38097_c0_g1 | PREDICTED: auxin-responsive protein SAUR24-like [Phoenix dactylifera] |
| TRINITY_DN46274_c0_g1 | PREDICTED: abscisic acid receptor PYL4-like [Musa acuminata subsp. malaccensis] |
| TRINITY_DN55661_c0_g1 | PREDICTED: ethylene receptor 3 [Elaeis guineensis] |
| TRINITY_DN63637_c1_g7 | protein TIFY 10a-like [Dendrobium catenatum] |
| TRINITY_DN54354_c0_g1 | PREDICTED: auxin-induced protein 6B-like [Elaeis guineensis] |
| TRINITY_DN69963_c0_g1 | PREDICTED: ETHYLENE INSENSITIVE 3-like 1 protein [Musa acuminata subsp. malaccensis] |
| TRINITY_DN64972_c3_g1 | PREDICTED: transcription factor MYC2-like [Vigna angularis] |
| TRINITY_DN76054_c3_g1 | PREDICTED: probable protein phosphatase 2C 6 [Elaeis guineensis] |
| TRINITY_DN51929_c3_g1 | auxin response factor 15-like isoform X1 [Asparagus officinalis] |
| TRINITY_DN52982_c4_g2 | uncharacterized protein LOC109840677 [Asparagus officinalis] |
| TRINITY_DN76146_c2_g3 | PYL1 [Zoysia japonica] |
| TRINITY_DN63637_c1_g6 | protein TIFY 10A-like [Asparagus officinalis] |
| TRINITY_DN62954_c0_g3 | transcription factor MYC2 [Lilium regale] |
| TRINITY_DN61123_c0_g1 | PREDICTED: transcription factor PIF4 isoform X7 [Elaeis guineensis] |
| TRINITY_DN63637_c1_g3 | PREDICTED: protein TIFY 10a-like [Nelumbo nucifera] |
| TRINITY_DN63637_c1_g2 | pnFL-2 [Zea mays] |
| TRINITY_DN57433_c2_g2 | PREDICTED: auxin transporter-like protein 3 [Elaeis guineensis] |
| TRINITY_DN58400_c1_g1 | PREDICTED: jasmonic acid-amido synthetase JAR1-like [Musa acuminata subsp. malaccensis] |
| TRINITY_DN91443_c0_g1 | Abscisic acid receptor PYL6 [Apostasia shenzhenica] |
| TRINITY_DN46172_c1_g1 | PREDICTED: auxin-responsive protein IAA30 [Elaeis guineensis] |
| TRINITY_DN46172_c1_g2 | PREDICTED: auxin-induced protein 22D [Elaeis guineensis] |
| TRINITY_DN46172_c1_g3 | PREDICTED: auxin-induced protein 22D-like [Phoenix dactylifera] |
| TRINITY_DN76064_c5_g3 | PREDICTED: auxin-responsive protein SAUR71-like [Musa acuminata subsp. malaccensis] |
| TRINITY_DN57115_c0_g1 | EIN3-binding F-box protein 1 [Ananas comosus] |
| TRINITY_DN73280_c3_g1 | PREDICTED: ABSCISIC ACID-INSENSITIVE 5-like protein 7 [Phoenix dactylifera] |
| TRINITY_DN45936_c2_g1 | hypothetical protein VITISV_036640 [Vitis vinifera] |
| TRINITY_DN54256_c0_g2 | PREDICTED: auxin response factor 11 [Elaeis guineensis] |
| TRINITY_DN63885_c8_g1 | transcription factor MYC2 [Lilium regale] |
| TRINITY_DN71283_c1_g3 | PREDICTED: protein TRANSPORT INHIBITOR RESPONSE 1-like [Phoenix dactylifera] |
| TRINITY_DN71283_c1_g2 | PREDICTED: protein TRANSPORT INHIBITOR RESPONSE 1-like [Phoenix dactylifera] |
| TRINITY_DN75209_c1_g6 | PREDICTED: probable xyloglucan endotransglucosylase/hydrolase protein 23 [Elaeis guineensis] |
| TRINITY_DN75209_c1_g5 | unnamed protein product, partial [Vitis vinifera] |
| TRINITY_DN33643_c0_g1 | bZIP transcription factor TGA10 isoform X2 [Asparagus officinalis] |
| TRINITY_DN69214_c2_g5 | PREDICTED: F-box protein GID2-like isoform X1 [Phoenix dactylifera] |
| TRINITY_DN69214_c2_g4 | F-box protein GID2 [Apostasia shenzhenica] |
| TRINITY_DN45936_c2_g5 | Glycoside hydrolase [Macleaya cordata] |
| TRINITY_DN52830_c0_g2 | PREDICTED: transcription factor HBP-1b(c38)-like [Phoenix dactylifera] |
| TRINITY_DN54675_c0_g1 | hypothetical protein POPTR_001G306300v3 [Populus trichocarpa] |
| TRINITY_DN54675_c0_g2 | PREDICTED: auxin-induced protein 6B-like [Musa acuminata subsp. malaccensis] |
| TRINITY_DN54675_c0_g3 | hypothetical protein POPTR_001G306300v3 [Populus trichocarpa] |
| TRINITY_DN74535_c0_g1 | PREDICTED: ethylene receptor 2-like [Elaeis guineensis] |
| TRINITY_DN40999_c0_g1 | PREDICTED: auxin-responsive protein SAUR32-like [Musa acuminata subsp. malaccensis] |
| TRINITY_DN62852_c4_g2 | PREDICTED: mitogen-activated protein kinase 1 [Elaeis guineensis] |
| TRINITY_DN43446_c0_g1 | auxin response factor 11-like [Ananas comosus] |
| TRINITY_DN68800_c1_g1 | PREDICTED: ABSCISIC ACID-INSENSITIVE 5-like protein 2 [Phoenix dactylifera] |
| TRINITY_DN67083_c2_g1 | two-component response regulator ORR1-like [Asparagus officinalis] |
| TRINITY_DN45173_c5_g1 | auxin-responsive protein SAUR50 [Populus trichocarpa] |
| TRINITY_DN50341_c2_g3 | auxin-induced protein TGSAUR12 [Tulipa gesneriana] |
| TRINITY_DN50341_c2_g1 | auxin-induced protein TGSAUR12 [Tulipa gesneriana] |
| TRINITY_DN47790_c1_g1 | unknown [Lotus japonicus] |
| TRINITY_DN68595_c1_g2 | PREDICTED: abscisic acid receptor PYL8-like [Musa acuminata subsp. malaccensis] |
| TRINITY_DN73741_c5_g3 | transcription factor MYC2 [Lilium regale] |
| TRINITY_DN51361_c0_g1 | protein TIFY 10A-like [Asparagus officinalis] |
| TRINITY_DN64793_c1_g4 | transcription factor MYC2 [Lilium regale] |
| TRINITY_DN51361_c0_g2 | protein TIFY 10A-like [Asparagus officinalis] |
| TRINITY_DN61748_c0_g1 | PREDICTED: pseudo histidine-containing phosphotransfer protein 2-like isoform X2 [Elaeis guineensis] |
| TRINITY_DN48142_c4_g4 | PREDICTED: ABSCISIC ACID-INSENSITIVE 5-like protein 2 [Musa acuminata subsp. malaccensis] |
| TRINITY_DN75993_c1_g1 | PREDICTED: transcription factor bHLH3-like [Nicotiana tabacum] |
| TRINITY_DN71161_c2_g1 | PREDICTED: mitogen-activated protein kinase homolog MMK1 [Nelumbo nucifera] |
| TRINITY_DN44282_c0_g1 | Auxin-responsive protein IAA31 [Dendrobium catenatum] |
| TRINITY_DN62324_c2_g1 | PREDICTED: histidine-containing phosphotransfer protein 1 isoform X1 [Lupinus angustifolius] |
| TRINITY_DN62954_c0_g4 | transcription factor MYC2 [Lilium regale] |
| TRINITY_DN58084_c0_g1 | PREDICTED: abscisic acid receptor PYL8-like [Elaeis guineensis] |
| TRINITY_DN53383_c3_g1 | PREDICTED: probable protein phosphatase 2C 8 [Oryza brachyantha] |
| TRINITY_DN53383_c3_g5 | probable protein phosphatase 2C 51 [Populus trichocarpa] |
| TRINITY_DN45344_c1_g1 | PREDICTED: pseudo histidine-containing phosphotransfer protein 6 [Cucumis melo] |
| TRINITY_DN45344_c1_g2 | PREDICTED: pseudo histidine-containing phosphotransfer protein 6 isoform X1 [Phoenix dactylifera] |
| TRINITY_DN63491_c0_g1 | auxin-induced protein TGSAUR21 [Tulipa gesneriana] |
| TRINITY_DN65524_c2_g2 | PREDICTED: probable protein phosphatase 2C 6 [Elaeis guineensis] |
| TRINITY_DN52631_c4_g5 | putative small auxin-up RNA [Rosa chinensis] |
| TRINITY_DN71283_c0_g2 | PREDICTED: protein TRANSPORT INHIBITOR RESPONSE 1-like [Phoenix dactylifera] |
| TRINITY_DN53250_c1_g1 | transcription factor PIF3-like [Asparagus officinalis] |
| TRINITY_DN51828_c1_g4 | PREDICTED: F-box protein GID2-like isoform X1 [Phoenix dactylifera] |
| TRINITY_DN51828_c1_g6 | PREDICTED: F-box protein GID2 [Elaeis guineensis] |
| TRINITY_DN51828_c1_g1 | PREDICTED: F-box protein GID2 [Elaeis guineensis] |
| TRINITY_DN82298_c0_g1 | PREDICTED: indole-3-acetic acid-induced protein ARG7-like [Elaeis guineensis] |
| TRINITY_DN64671_c2_g2 | PREDICTED: protein TIFY 10a [Elaeis guineensis] |
| TRINITY_DN48923_c6_g3 | PREDICTED: beta-1,4-mannosyl-glycoprotein 4-beta-N-acetylglucosaminyltransferase [Musa acuminata subsp. malaccensis] |
| TRINITY_DN52830_c0_g1 | PREDICTED: transcription factor TGA2.2-like isoform X2 [Elaeis guineensis] |
| TRINITY_DN62613_c0_g5 | PREDICTED: indole-3-acetic acid-amido synthetase GH3.17-like [Elaeis guineensis] |
| TRINITY_DN17397_c0_g1 | auxin-induced protein 15A-like [Ananas comosus] |
| TRINITY_DN63777_c0_g1 | shaggy-related protein kinase eta-like isoform X1 [Asparagus officinalis] |
| TRINITY_DN52345_c1_g3 | PREDICTED: F-box protein GID2-like [Musa acuminata subsp. malaccensis] |
| TRINITY_DN57840_c0_g3 | Protein TIFY 10A [Apostasia shenzhenica] |
| TRINITY_DN57840_c0_g2 | protein TIFY 10A-like [Asparagus officinalis] |
| TRINITY_DN60433_c4_g8 | transcription factor MYC2 [Lilium regale] |
| TRINITY_DN57840_c0_g6 | protein TIFY 10A-like [Asparagus officinalis] |
| TRINITY_DN60522_c1_g1 | PREDICTED: transcription factor PIF4-like isoform X2 [Elaeis guineensis] |
| TRINITY_DN38529_c1_g1 | PREDICTED: auxin-responsive protein SAUR36-like [Gossypium arboreum] |
| TRINITY_DN52174_c1_g1 | PREDICTED: probable serine/threonine-protein kinase At5g41260 isoform X1 [Elaeis guineensis] |
| TRINITY_DN62417_c0_g2 | PREDICTED: histidine-containing phosphotransfer protein 2 isoform X1 [Elaeis guineensis] |
| TRINITY_DN52064_c3_g1 | PREDICTED: probable serine/threonine-protein kinase At5g41260 [Elaeis guineensis] |
| TRINITY_DN65048_c4_g2 | PREDICTED: probable indole-3-acetic acid-amido synthetase GH3.8 [Elaeis guineensis] |
| TRINITY_DN75768_c0_g2 | unknown [Zea mays] |
| TRINITY_DN53568_c4_g1 | auxin-induced protein TGSAUR21 [Tulipa gesneriana] |
| TRINITY_DN53568_c4_g2 | auxin-induced protein TGSAUR22 [Tulipa gesneriana] |
| TRINITY_DN60612_c2_g5 | PREDICTED: two-component response regulator ORR21 [Elaeis guineensis] |
| TRINITY_DN68595_c1_g1 | PREDICTED: abscisic acid receptor PYL8-like [Elaeis guineensis] |
| TRINITY_DN75209_c1_g8 | hypothetical protein PAHAL_D00457 [Panicum hallii] |
| TRINITY_DN40192_c0_g1 | PREDICTED: abscisic acid receptor PYL2-like [Elaeis guineensis] |
| TRINITY_DN45662_c0_g1 | Auxin-responsive protein IAA31 [Dendrobium catenatum] |
| TRINITY_DN74533_c3_g2 | abscisic acid receptor PYL4 [Citrus clementina] |
| TRINITY_DN32412_c0_g1 | PREDICTED: probable protein phosphatase 2C 24 [Elaeis guineensis] |
| TRINITY_DN47790_c2_g1 | auxin-responsive protein SAUR50-like [Brassica napus] |
| TRINITY_DN60612_c2_g2 | PREDICTED: two-component response regulator ORR21-like isoform X1 [Phoenix dactylifera] |
| TRINITY_DN38712_c0_g1 | PREDICTED: auxin-responsive protein SAUR50-like [Elaeis guineensis] |
| TRINITY_DN54283_c1_g1 | two-component response regulator ORR9-like [Phalaenopsis equestris] |
| TRINITY_DN67273_c1_g1 | PREDICTED: DELLA protein SLR1 isoform X1 [Elaeis guineensis] |
| TRINITY_DN54283_c1_g3 | two-component response regulator ORR9-like [Dendrobium catenatum] |
| TRINITY_DN57433_c2_g5 | Aa_trans domain-containing protein, partial [Cephalotus follicularis] |
| TRINITY_DN68285_c2_g3 | PREDICTED: BTB/POZ domain and ankyrin repeat-containing protein NPR5-like isoform X1 [Elaeis guineensis] |
| TRINITY_DN52427_c0_g1 | PREDICTED: auxin-induced protein 6B-like [Juglans regia] |
| TRINITY_DN66997_c3_g5 | transcription factor MYC2 [Lilium regale] |
| TRINITY_DN52427_c0_g2 | PREDICTED: auxin-induced protein 6B-like [Juglans regia] |
| TRINITY_DN69050_c0_g1 | PREDICTED: ETHYLENE INSENSITIVE 3-like 1 protein [Elaeis guineensis] |
| TRINITY_DN71834_c1_g2 | PREDICTED: ethylene receptor 2 [Phoenix dactylifera] |
| TRINITY_DN49569_c5_g2 | PREDICTED: auxin-induced protein 15A [Elaeis guineensis] |
| TRINITY_DN49569_c5_g5 | PREDICTED: auxin-induced protein 15A [Elaeis guineensis] |
| TRINITY_DN49569_c5_g4 | PREDICTED: auxin-induced protein X10A [Phoenix dactylifera] |
| TRINITY_DN57945_c2_g2 | auxin-induced protein TGSAUR21 [Tulipa gesneriana] |
| TRINITY_DN68646_c6_g4 | transcription factor MYC2 [Lilium regale] |
| TRINITY_DN64697_c0_g2 | probable xyloglucan endotransglucosylase/hydrolase protein 23 [Quercus suber] |
| TRINITY_DN64697_c0_g1 | xyloglucantransglusylase/hydrolase 1 [Gladiolus grandiflorus] |
| TRINITY_DN53597_c3_g4 | protein TIFY 3 isoform X2 [Zea mays] |
| TRINITY_DN41491_c0_g1 | PREDICTED: two-component response regulator ORR5-like [Phoenix dactylifera] |
| TRINITY_DN55022_c1_g2 | PREDICTED: probable xyloglucan endotransglucosylase/hydrolase protein 23, partial [Phoenix dactylifera] |
| TRINITY_DN55022_c1_g1 | PREDICTED: probable xyloglucan endotransglucosylase/hydrolase protein 23, partial [Phoenix dactylifera] |
| TRINITY_DN48680_c0_g1 | auxin-responsive protein SAUR32 [Amborella trichopoda] |
| TRINITY_DN58411_c0_g1 | hypothetical protein OsJ_22495 [Oryza sativa Japonica Group] |
| TRINITY_DN61671_c1_g1 | PREDICTED: auxin-responsive protein SAUR32-like [Musa acuminata subsp. malaccensis] |
| TRINITY_DN58411_c0_g3 | PREDICTED: probable xyloglucan endotransglucosylase/hydrolase protein 23, partial [Phoenix dactylifera] |
| TRINITY_DN48680_c0_g5 | PREDICTED: auxin-responsive protein SAUR32-like [Musa acuminata subsp. malaccensis] |
| TRINITY_DN55227_c0_g1 | PREDICTED: auxin-responsive protein IAA1-like [Phoenix dactylifera] |
| TRINITY_DN64859_c8_g1 | Protein phosphatase 2C [Cynara cardunculus var. scolymus] |
| TRINITY_DN57667_c0_g4 | Protein TIFY 10A [Dendrobium catenatum] |
| TRINITY_DN49569_c5_g1 | PREDICTED: auxin-induced protein 15A [Elaeis guineensis] |
| TRINITY_DN37744_c0_g1 | PREDICTED: auxin-responsive protein SAUR32-like [Phoenix dactylifera] |
| TRINITY_DN57667_c0_g3 | PREDICTED: protein TIFY 10a-like [Musa acuminata subsp. malaccensis] |
| TRINITY_DN42974_c0_g2 | auxin-responsive protein SAUR50 [Sorghum bicolor] |
| TRINITY_DN59544_c1_g3 | transcription factor MYC2 [Lilium regale] |
| TRINITY_DN56670_c1_g2 | PREDICTED: probable histidine kinase 3 isoform X1 [Elaeis guineensis] |
| TRINITY_DN48680_c0_g3 | PREDICTED: auxin-responsive protein SAUR32-like [Phoenix dactylifera] |
| TRINITY_DN74368_c0_g1 | PREDICTED: DELLA protein RGL1-like [Phoenix dactylifera] |
| TRINITY_DN51727_c1_g2 | PREDICTED: bZIP transcription factor TRAB1 isoform X1 [Elaeis guineensis] |
| TRINITY_DN71084_c0_g1 | PREDICTED: LOW QUALITY PROTEIN: transcription factor MYC2-like [Elaeis guineensis] |
| TRINITY_DN45611_c2_g1 | PREDICTED: auxin response factor 15-like isoform X3 [Elaeis guineensis] |
| TRINITY_DN50186_c1_g3 | PREDICTED: auxin-responsive protein SAUR32-like [Elaeis guineensis] |
| TRINITY_DN62244_c0_g4 | PREDICTED: probable histidine kinase 4 [Elaeis guineensis] |
| TRINITY_DN67812_c4_g7 | transcription factor MYC2 [Lilium regale] |
| TRINITY_DN57912_c0_g3 | ethylene insensitive 2 [Zea mays] |
| TRINITY_DN57912_c0_g2 | protein ETHYLENE-INSENSITIVE 2-like isoform X6 [Ananas comosus] |
| TRINITY_DN57912_c0_g1 | PREDICTED: ethylene-insensitive protein 2-like [Lupinus angustifolius] |
| TRINITY_DN48003_c1_g2 | PREDICTED: serine/threonine-protein kinase SAPK3-like [Phoenix dactylifera] |
| TRINITY_DN48680_c0_g2 | PREDICTED: auxin-responsive protein SAUR32-like [Phoenix dactylifera] |
| TRINITY_DN48680_c0_g4 | PREDICTED: auxin-responsive protein SAUR32-like [Phoenix dactylifera] |
| TRINITY_DN65089_c0_g2 | auxin-induced protein TGSAUR21 [Tulipa gesneriana] |
| TRINITY_DN65089_c0_g1 | unknown [Lotus japonicus] |
| TRINITY_DN73841_c0_g4 | PREDICTED: probable serine/threonine-protein kinase At4g35230 [Musa acuminata subsp. malaccensis] |
| TRINITY_DN65089_c0_g4 | auxin-induced protein TGSAUR22 [Tulipa gesneriana] |
| TRINITY_DN63491_c0_g4 | auxin-responsive protein SAUR50-like [Phalaenopsis equestris] |
| TRINITY_DN63491_c0_g5 | PREDICTED: auxin-induced protein 15A-like [Nicotiana tabacum] |
| TRINITY_DN63491_c0_g2 | auxin-induced protein TGSAUR21 [Tulipa gesneriana] |
| TRINITY_DN63491_c0_g3 | unknown [Lotus japonicus] |
| TRINITY_DN42647_c0_g1 | PREDICTED: auxin-responsive protein SAUR71-like [Elaeis guineensis] |

**Supplementary Table 8.** The primers were used in this study

| **Name** | **Sequence (5'-3')** |
| --- | --- |
| LiMYB305-F | ATGGACAGGAAAGTAGGGACTAATG |
| LiMYB305-R | CTAGTATTGCATAGCCCACAGTTCC |
| qLiMYB305-F | GGCCAAACCTCCATGCTTTC |
| qLiMYB305-R | GGTCGTTCGAAGTCTGGCCT |
| LiActin-F | TGTGCTTTCCCTCTACGCCAGT |
| LiActin-R | TCCCTCACGATTTCCCGCTCT |
| pBWA(V)HS-LiMYB305-F | CAGTGGTCTCACAACATGGACAGGAAAGTAGGGAC |
| pBWA(V)HS-LiMYB305-R | CAGTGGTCTCATACAGTATTGCATAGCCCACAGTT |
| TRV2-*LiMYB305*-F | GTCCAGTCCTGGCCTGACAGGAAAGTAGGGACTAATGAAGATCTTG |
| TRV2-*LiMYB305*-R | GACCACAAGTGGCCACGACGAACATCAGGCCTCAG |
| q*LiLiS* -F | TCGCCTTGTTGAGATCATCCTCTTC |
| q*LiLiS* -R | TGTCTCCACGGAGCTGCTGTT |
| q*LiOcS*-F | TCCGCCAATTACCACCCAACCA |
| q*LiOcS*-R | GCTGAGCCACTGGATCGTCTGA |
| q*LiMyS*-F | GACTTGTTGGAGCCATGTCTTAGAGA |
| q*LiMyS*-R | AACTCAACTCGTCGCCAAGAAGT |
